# Asymmetric Iron‐Catalyzed Vicinal C(sp^3^)─H Diamination of Carboxylic Acids

**DOI:** 10.1002/anie.4041858

**Published:** 2026-06-07

**Authors:** Bing Zhou, Yuan Zheng, Xiulan Xie, Marcel Hemming, Sergei I. Ivlev, Eric Meggers

**Affiliations:** ^1^ Fachbereich Chemie Philipps‐Universität Marburg Marburg Germany

**Keywords:** 1,2‐diamines, amination, asymmetric catalysis, C─H activation, carboxylic acids, iron

## Abstract

The direct construction of chiral 1,2‐diamines through the stereoselective functionalization of two vicinal C(sp^3^)─H bonds remains a significant synthetic challenge. We report an iron‐catalyzed, carboxylate‐directed asymmetric vicinal α,β‐C(sp^3^) ─H diamination of readily available carboxylic acids that furnishes chiral α,β‐diamino acids in a single pot with high regio‐, diastereo‐, and enantioselectivity (up to >20:1 dr and >99% ee). Using a chiral tetradentate N4‐ligated Fe(II) catalyst and BocNHOMs as the aminating reagent, a broad range of aromatic and aliphatic substrates undergo two sequential, nitrene‐mediated C─H aminations under mild conditions. Mechanistic studies indicate stepwise amination via iron−nitrenoid species, where the initial site of hydrogen‐atom transfer (β for aromatic substrates, α for aliphatic ones) dictates the order of functionalization. The method tolerates diverse functional groups and operates enantioconvergently for selected racemic substrates. This sustainable iron‐catalyzed strategy enables direct conversion of abundant carboxylic acids into versatile chiral α,β‐diamino building blocks amenable to peptide synthesis and heterocycle formation.

## Graphical abstract

An iron‐catalyzed vicinal α,β‐C(sp^3^)─H diamination of readily available carboxylic acids furnishes chiral α,β‐diamino acids in a single pot with high regio‐, diastereo‐, and enantioselectivity (up to >20:1 dr and >99% ee).

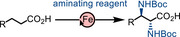

## 1 Introduction

Chiral 1,2‐diamines are ubiquitous structural motifs found in bioactive compounds, including natural products and pharmaceuticals. They are also widely employed in organocatalysts, as ligands for metal catalysts, as components of chiral auxiliaries, and as highly valuable chiral synthetic building blocks. Unsurprisingly, numerous strategies have been developed to access nonracemic vicinal diamines in a catalytic asymmetric fashion [1]. These approaches include C─H bond functionalizations [1, 2] (e.g., reduction of diimines), C─C bond‐forming reactions [1, 3, 4, 5] (e.g., Mannich reactions), and single C─N bond‐forming transformations [1, 6] (e.g., amination of enamines or allylamines).

**FIGURE 1:**
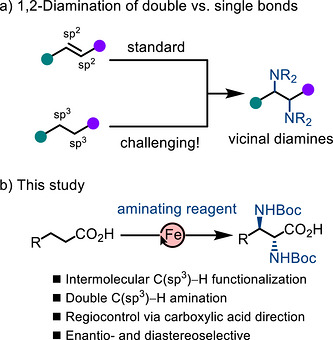
Background and this study. (a) Well‐established 1,2‐diaminations of alkenes versus challenging 1,2‐diaminations of alkanes. (b) This study: Fe‐catalyzed asymmetric 1,2‐aminations of carboxylic acids.

From the standpoint of synthetic efficiency and step economy, reactions that simultaneously introduce two C─N bonds are particularly attractive (Figure 1a). In this regard, significant progress has been achieved in the catalytic, enantioselective diamination of alkenes, where two sp^2^‐hybridized carbons are transformed into two C(sp^3^)─N bonds [7, 8]. However, this strategy requires the pre‐installation of a C═C double bond. A more direct and appealing approach would be the catalytic asymmetric conversion of two vicinal C(sp^3^)─H bonds into C(sp^3^)─N bonds. Yet, the simultaneous control of regioselectivity, diastereoselectivity, and enantioselectivity renders this transformation especially challenging. Some progress has been achieved in this area. The Shi group developed a Pd‐catalyzed diamination of terminal olefins at the allylic and homoallylic positions [9, 10], while Lambert reported a trisaminocyclopropenium ion‐catalyzed electrophotocatalytic diamination at benzylic and homobenzylic sites [11]. Both strategies operate through an intermediate dehydrogenation to alkenes. More recently, Dauban introduced a directed catalytic enantioselective 1,2‐diamination employing a chiral dirhodium(II) catalyst [12]. This approach, however, generates only a single stereocenter and requires two distinct steps.

Herein, we present our progress toward the catalytic asymmetric diamination of vicinal C(sp^3^)─H bonds. In a single‐pot transformation, abundant carboxylic acids are converted into highly valuable chiral α,β‐diamino acids with excellent levels of regio‐, diastereo‐, and enantioselectivity under iron catalysis (Figure 1b). This method provides an economical and convenient strategy for transforming abundant and inexpensive starting materials into high‐value chiral building blocks. By utilizing iron catalysis to achieve two stereocontrolled C(sp^3^)─H functionalizations, it also addresses key principles of sustainable chemistry.

## 2 Results and Discussion

### 2.1 Initial Experiments

Our group recently developed an iron‐catalyzed, carboxylic acid‐directed α‐amination of carboxylic acids [13, 14], and we also demonstrated the related β‐amination in cases with especially activated β‐C─H bonds [15]. We were wondering whether these nitrene‐mediated C(sp^3^)─H aminations could be a basis to achieve more challenging α,β‐diaminations. To explore this, we selected phenylpropanoic acid (**1a**) as a model substrate because it contains an activated benzylic C(sp^3^)─H bond in β‐position, rendering it a promising starting point (Table 1). After extensive catalyst and condition screening, we identified a successful protocol: the reaction of carboxylic acid **1a** with an excess of the aminating reagent *tert*‐butyl ((methylsulfonyl)oxy)carbamate (BocNHOMs, 10 equivalents) in the presence of piperidine (12 equivalents) in CHCl_3_ at –15°C for 64 h using the chiral iron catalyst (*S*)‐**Fe1** provided the α,β‐diamino acid (2*R*,3*R*)‐**2a** in 68% isolated yield, with 95% ee and 12:1 dr (entry 1). The corresponding monoaminated products, α‐amino acid **2b** and β‐amino acid **2c**, were detected only in trace amounts.

**TABLE 1:** Initial experiments and optimization.

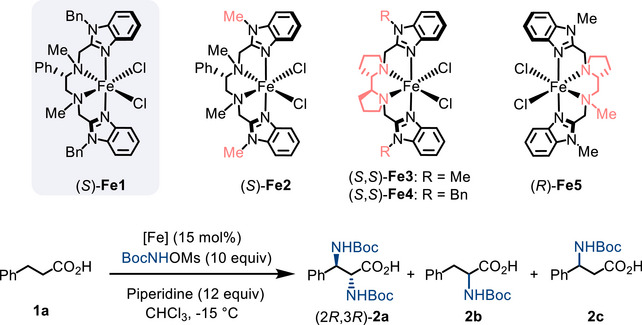								
Entry	Catalyst	Conditions^a^	Conv. [%]	α‐mono (2b)	β‐mono (2c)	α,β‐diamination (2a)		
				Yield [%]^b^	Yield [%]^b^	Yield [%]^b^	ee [%]^c^	Dr^d^
1	(*S*)‐**Fe1**	Standard	100	<5	<5	68	95	12
2	(*S*)‐**Fe2**	Standard	89	7	19	38	68	6
3	(*S*,*S*)‐**Fe3**	Standard	82	15	47	19	87	2
4	(*S*,*S*)‐**Fe4**	Standard	83	21	54	7	n.d.^e^	n.d.^e^
5	(*R*)‐**Fe5**	Standard	86	11	39	12	82^f^	2
6	(*S*)‐**Fe1**	base Et_3_N	24	5	14	<5	n.d.^e^	n.d.^e^
7	(*S*)‐**Fe1**	base TMP	85	9	49	0	—	—
8	(*S*)‐**Fe1**	base K_2_CO_3_	82	0	29	0	—	—

^a^ Standard reaction conditions: **1a** (0.1 mmol), Fe catalyst (15.0 mol%), BocNHOMs (1.0 mmol), and piperidine (1.2 mmol) in CHCl_3_ (1.0 mL) were stirred at –15°C for 64 h under an atmosphere of N_2_.

^b^ Isolated yields.

^c^ Determined by HPLC on a chiral stationary phase.

^d^ Determined by ^1^H NMR.

^e^ Not determined.

^f^ Mirror‐imaged (2*S*,3*S*)‐**2a** as the main product.

Importantly, the method proved highly sensitive to structural changes in the chiral iron catalyst. The overall catalyst scaffold features an iron(II) center coordinated by a tetradentate N4‐ligand composed of two benzimidazole moieties tethered through a chiral bisamine backbone. Two labile chloride ligands complement the coordination sphere. Replacing the benzylic substituents at the benzimidazole moieties with methyl groups (**Fe2**) led to a reduced diamination yield (38%), reduced enantioselectivity (68% ee), and reduced diastereoselectivity (6:1 dr) (entry 2). Using instead our previously reported standard α‐amination catalyst with a chiral 2,2’‐bipyrrolidine backbone (**Fe3**, previously dubbed **FeBIM**) [13, 14] further decreased the diamination yield to 19% (entry 3), while substituting methyl with benzyl groups at the benzimidazole moieties (**Fe4**) lowered the yield to only 7% (entry 4). Similarly, catalyst **Fe5** [15], which we recently reported for the efficient β‐amination of 3‐indolepropionic acids, provided **2a** in just 12% yield, with β‐amination predominating (entry 5). The choice of base was also critical: replacing piperidine with Et_3_N, TMP, or K_2_CO_3_ resulted in inferior outcomes (entries 6–8).

**FIGURE 2:**
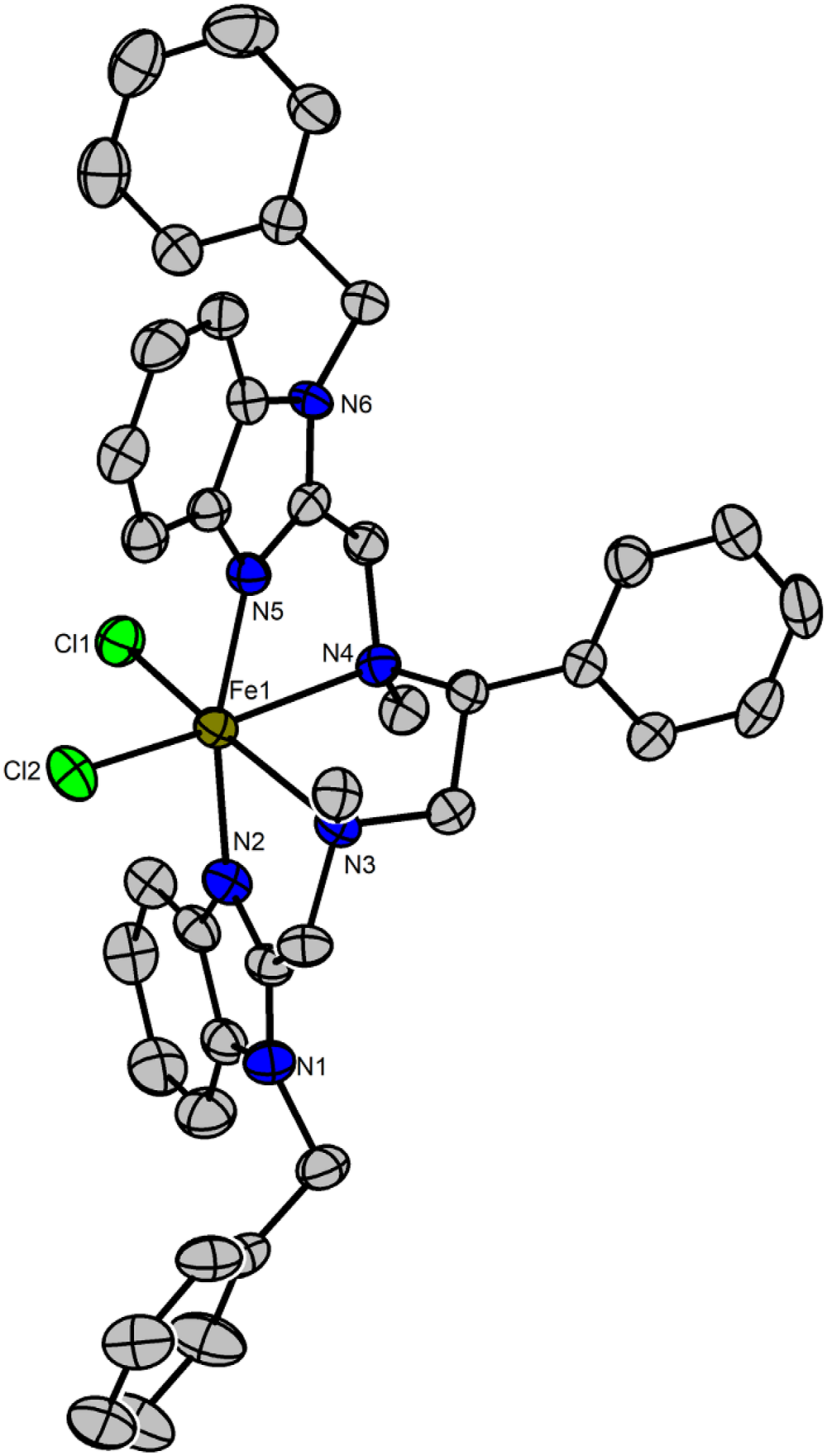
Crystal structure of (*R*)‐**Fe1** derivative with iron in the oxidation state III. The chloride counterion, a CH_2_Cl_2_ solvent molecule, and the hydrogen atoms are not shown. Displacement ellipsoids are shown at 50% probability level at 230 K.

To summarize this part, we identified an iron catalyst (**Fe1**) capable of stereoselectively aminating two C(sp^3^)─H bonds. Figure 2 displays an x‐ray crystal structure of a related Fe(III) complex obtained by reacting the tetradentate ligand with FeCl_3_ hydrate [16]. It reveals a *C*_2_‐symmetric complex with *cis*‐α coordination of the tetradentate ligand. Notably, a single stereogenic carbon in the ligand backbone dictates the configuration of the iron center (Δ).

### 2.2 Substrate Scope

**FIGURE 3:**
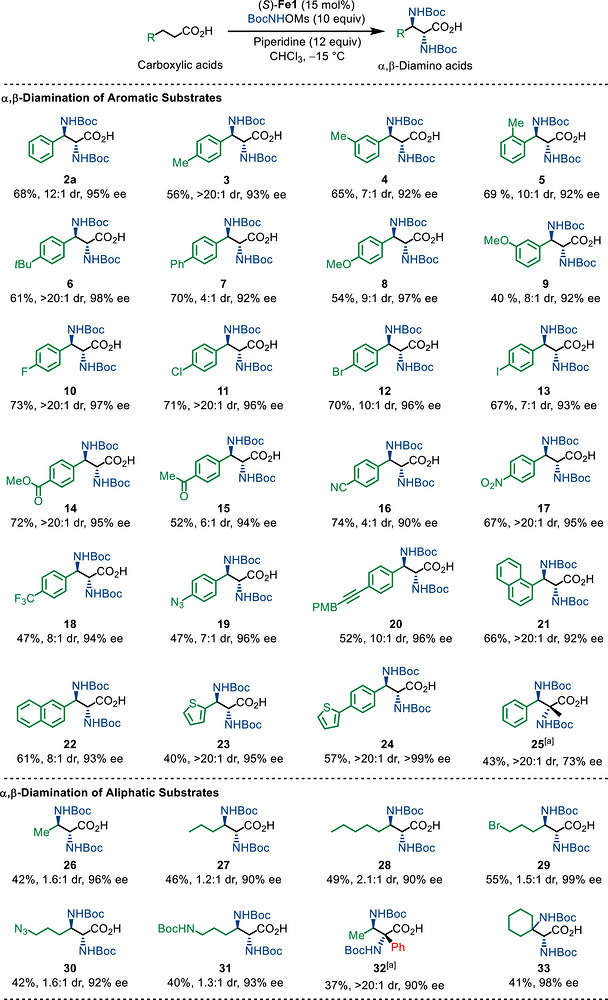
Substrate scope. Reaction conditions: Carboxylic acids (0.1 mmol), (*S*)‐**Fe1** (15 mol%), BocNHOMs (1.0 mmol), and piperidine (1.2 mmol) were stirred in CHCl_3_ (1.0 mL) at –15°C for 64 h under an atmosphere of nitrogen. Enantiomeric excess values were determined by HPLC analysis on a chiral stationary phase and are shown for the major diastereomers. Diastereomeric ratios were determined by ^1^H NMR. Shown are isolated yields. PMB = *p*‐methoxybenzene. ^[a]^Reaction performed with racemic carboxylic acids.

Next, we explored the substrate scope, beginning with modified 3‐phenylpropionic acids (Figure 3). Introduction of a methyl substituent at the *para*‐, *meta*‐, or *ortho*‐position of the phenyl ring provided diamino acids **3**–**5** in 56%–69% yield, with diastereomeric ratios of 7:1 to >20:1 and 92%–93% ee. A *para*‐*tert*‐butyl substituent delivered diamino acid **6** in 61% yield, with excellent diastereoselectivity (>20:1 dr) and very high enantioselectivity (98% ee). A broad range of electron‐donating and electron‐withdrawing substituents including phenyl, methoxy, fluoro, chloro, bromo, iodo, methoxycarbonyl, acetyl, cyano, nitro, trifluoromethyl, azido, and acetylide were well tolerated, affording diamino acids **7**–**20** in 40%–73% yield, with dr values ranging from 4:1 to >20:1 and ee values of 90%–97%. Extension to larger aromatic systems showed that 3‐(1‐naphthyl)propionic acid afforded the diamino acid **21** in 66% yield, >20:1 dr, and 92% ee, whereas the regioisomer 3‐(2‐naphthyl)propionic acid afforded **22** in 61% yield, 8:1 dr, and 93% ee. For heteroaromatic substrates, 3‐(2‐thienyl)propionic acid produced diamino acid **23** in 40% yield, >20:1 dr, and 95% ee, while the 3‐(3‐thienyl) isomer delivered **24** in 57% yield with nearly perfect stereoselectivity (>20:1 dr, >99% ee). Interestingly, racemic 2‐methyl‐3‐phenylpropionic acid, which contains both a tertiary C─H bond and a stereocenter at the 2‐position, underwent an enantioconvergent transformation to provide diamino acid **25** in 43% yield, >20:1 dr, and a modest 73% ee.

We next examined the more challenging diamination of aliphatic carboxylic acids, where the unactivated β‐position makes functionalization less favorable [17]. Under our standard conditions, butyric acid was converted to diamino acid **26** in 42% yield, 1.6:1 dr, and 96% ee. Extending the chain length, hexanoic acid afforded diamino acid **27** in 46% yield with 1.2:1 dr and 90% ee (77% ee for the minor diastereomer), while caprylic acid (octanoic acid) delivered **28** in 49% yield, 2.1:1 dr, and 90% ee. Functional group tolerance was demonstrated with substrates bearing a bromide, an azido group, or a Boc‐protected amine, which provided diamino acids **29**–**31** in 40%–55% yield, with dr values of 1.3:1 to 1.6:1 and ee values of 92%–99%. In an enantioconvergent process, racemic 2‐phenylbutyric acid gave diamino acid **32** in 37% yield, with excellent diastereoselectivity (>20:1 dr) and 90% ee. Finally, cyclohexylacetic acid, featuring a tertiary β‐C─H bond, was transformed into diamino acid **33** in 41% yield with 98% ee.

To summarize the substrate scope, this diamination protocol exhibits broad versatility across a range of arylpropionic and unactivated aliphatic carboxylic acids [18]. The method demonstrates high functional group tolerance and excellent enantioselectivity, albeit with varying degrees of diastereocontrol. Notably, the method also enables enantioconvergent pathways for racemic branched substrates. The moderate yields observed in specific cases can be largely ascribed to the competitive formation of monoaminated side products (see Table 1), sometimes in combination with incomplete conversion (e.g. 94% conversion for **9** and 82% conversion for **18**).

### 2.3 Mechanistic Investigations

We recently established that the iron‐catalyzed amination of carboxylic acids with BocNHOMs proceeds via a carboxylate‐directed, iron‐catalyzed C(sp^3^)─H nitrene insertion [13, 14]. This transformation thus merges nitrene‐mediated C(sp^3^)─H amination with site‐selective C(sp^3^)─H functionalization. The key intermediate is an iron‐nitrenoid species coordinated to the deprotonated carboxylic acid substrate. This species undergoes a regioselective hydrogen‐atom transfer (HAT) from a C(sp^3^)─H bond to the nitrenoid nitrogen, followed by C─N bond formation. The site of HAT dictates the product outcome: a 1,5‐HAT delivers N‐Boc‐protected α‐amino acids, whereas a 1,6‐HAT affords N‐Boc‐protected β‐amino acids.

**FIGURE 4:**
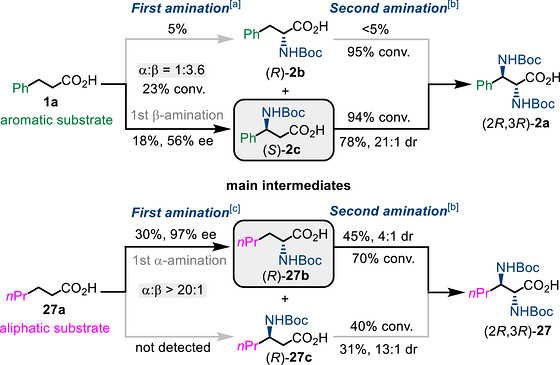
Stepwise amination experiments with catalyst (*S*)‐**Fe1**. The second amination step was always performed with enantiopure monoamino acids. Standard reaction conditions were applied (Table 1, entry 1) but with reduced reaction times as indicated: ^[a]^12 min, ^[b]^16 h, and ^[c]^6 h.

In our discovered diamination reaction, apparently two consecutive aminations occur, driven by the excess of the aminating reagent. To suppress diamination and detect monoamination intermediates [19, 20, 21], we subjected the aromatic substrate **1a** to the standard conditions but with a shortened reaction time of only 12 min, resulting in partial conversion (23%) (Figure 4). Under these conditions, only trace amounts of diamination were observed, while the β‐amino acid **2c** was the main product (18% yield, 56% ee) alongside minor formation of the α‐amino acid **2b** (5% yield). This α‐amino acid **2b** is only a sluggish substrate for the second amination step. However, exposure of enantiopure β‐amino acid (*S*)‐**2c** (99% ee) to the standard conditions for a reduced reaction time of 16 h enabled efficient α‐amination, affording the diamino acid (2*R*,3*R*)‐**2a** with a 21:1 dr. Thus, these findings reveal that, for carboxylic acids bearing an aromatic substituent at the β‐position, the initial amination occurs preferentially at the β‐site, followed by α‐amination.

We next investigated the stepwise amination of an aliphatic carboxylic acid. Applying the standard conditions to **27a** for a shortened reaction time of 6 h selectively afforded the α‐amino acid (*R*)‐**27b** (97% ee), with no detectable formation of the β‐amino acid (*R*)‐**27c** [22]. Subsequent exposure of enantiopure α‐amino acid (*R*)‐**27b** (99% ee) to the standard conditions for a reduced reaction time of 16 h furnished the diamino acid (2*R*,3*R*)‐**27** in 45% yield (70% conversion) with a 4:1 dr. In contrast, enantiopure β‐amino acid (*R*)‐**27c** proved to be a more sluggish substrate, reaching only 40% conversion under the same conditions. These results demonstrate that aliphatic carboxylic acids undergo initial amination at the α‐position with high enantioselectivity, followed by β‐amination with modest diastereoselectivity. For both aliphatic and aromatic substrates, the α‐amination proceeds with significantly higher stereoselectivity than the β‐amination.

**FIGURE 5:**
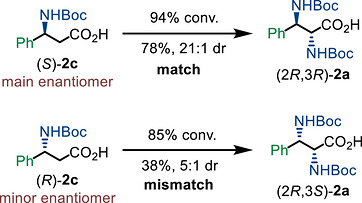
Match versus mismatch for the second amination step with catalyst (*S*)‐**Fe1**. Enantiopure β‐amino acids were used under standard reaction conditions (Table 1, entry 1) with reduced reaction times (16 h).

To evaluate potential match/mismatch effects, we examined how the stereochemistry established in the first amination influences the second amination step. We observed that the minor β‐amino acid enantiomer intermediate (*R*)‐**2c** reacted more slowly, with lower yield, and with lower diastereoselectivity than the major enantiomer (*S*)‐**2c** in the subsequent α‐amination (Figure 5). Notably, both enantiomers exhibit the same preference for formation of the *R*‐configuration at the α‐position, which accounts for the configuration of the major and minor diastereomer of the diamination product. Overall, these findings reveal that the major enantiomer produced in the first step is the matched intermediate, an aspect that is instrumental for achieving satisfactory overall yields and high stereoselectivity of the dual amination process [23].

Finally, magnetic susceptibility measurements reveal that the new catalyst (*S*)‐**Fe1** exists in a high‐spin state, whereas the previously reported standard catalyst for α‐monoaminations, (*S*,*S*)‐**Fe4**, exhibits a mixed‐spin state (50:50 high/low spin). The influence of the metal spin state on the catalytic activity and reaction pathways of iron complexes is well‐documented in the literature [24, 25, 26, 27, 28, 29].

### 2.4 Follow‐up Chemistry

The methodology disclosed herein facilitates the direct transformation of abundant carboxylic acids into high‐value chiral α,β‐diamino acids in a catalytic asymmetric fashion. Beyond their inherent value, these chiral α,β‐diamino acids serve as highly versatile building blocks [30]. Representative examples for follow‐up chemistry are provided in the Supporting Information. For example, chiral α,β‐diamino acids can be seamlessly integrated into peptide synthesis or cyclized in a single step to afford chiral 2‐imidazolidinones and 2‐imidazolines. Furthermore, reduction of the carboxyl group provides streamlined access to chiral 2,3‐diamino alcohols.

## 3 Conclusions

In summary, we have developed an iron‐catalyzed, carboxylic acid‐directed asymmetric vicinal C(sp^3^)─H diamination that converts simple and abundant carboxylic acids into highly valuable chiral α,β‐diamino acids in a single operation. This transformation forges two C(sp^3^)─N bonds with excellent levels of regio‐, diastereo‐, and enantioselectivity under mild conditions, without the need for prefunctionalization or preinstalled unsaturation. The method relies on a specifically tailored chiral N4 iron catalyst, in which a single stereogenic element in the ligand backbone governs both the metal‐centered chirality and the stereochemical outcome of two consecutive C─H amination events.

A broad substrate scope was demonstrated, encompassing a wide variety of substituted aromatic, heteroaromatic, and purely aliphatic carboxylic acids, with good functional group tolerance and consistently high enantioselectivities. Notably, enantioconvergent transformations of racemic substrates were observed in selected cases. Mechanistic investigations revealed that the diamination proceeds through two sequential, carboxylate‐directed nitrene‐mediated C(sp^3^)─H aminations, with the order of α‐ and β‐functionalization depending on substrate class. For aromatic systems, β‐amination precedes α‐amination, whereas aliphatic substrates undergo initial α‐amination followed by β‐functionalization. The stereochemical outcome of the second amination is strongly influenced by the configuration of the monoaminated intermediate, providing insight into the origin of diastereoselectivity.

This work demonstrates that iron catalysis can be harnessed to achieve two stereocontrolled C(sp^3^)─H functionalizations in a single process, offering a step‐economical and sustainable approach to complex chiral building blocks from inexpensive feedstock chemicals. Given the versatility of the resulting α,β‐diamino acids in peptide synthesis, heterocycle formation, and further derivatization, this methodology opens new opportunities for the streamlined synthesis of structurally and stereochemically rich nitrogen‐containing molecules. Although this study focused on synthetic methodology, future work will explore potential correlations between ligand structure, spin state, and the underlying mechanism.

## Conflicts of Interest

The authors declare no conflicts of interest.

## Supporting information




**Supporting File**: anie72955‐sup‐0001‐SuppMat.pdf.

**Supporting File**: anie72955‐sup‐0002‐Data.zip.

## Data Availability

The data that supports the findings of this study are available in the Supporting Information of this article.
