# Supplementary material for: Asymmetric Iron‐Catalyzed Vicinal C(sp3)─H Diamination of Carboxylic Acids
Source: Angew Chem Int Ed Engl. 2026 Jun 7;65(32):e4041858. doi: 10.1002/anie.4041858 (PMC13427101; doi:10.1002/anie.4041858)
Supplement: Supplementary file 1 — Supporting File: anie72955‐sup‐0001‐SuppMat.pdf. [file ANIE-65-e4041858-s001.pdf]

SUPPORTING INFORMATION

Asymmetric Iron-Catalyzed Vicinal C(sp^3)–H Diamination of Carboxylic Acids

Bing Zhou,[§] Yuan Zheng,[§] Xiulan Xie, Marcel Hemming, Sergei I. Ivlev, and Eric Meggers*

Fachbereich Chemie, Philipps-Universität Marburg, Hans-Meerwein-Strasse 4, 35043 Marburg, Germany

[§]These authors contributed equally.

*Email: meggers@chemie.uni-marburg.de

Table of Contents

1. General Information S3

2. Catalysts Synthesis and Characterization S4

2.1 Catalyst characterizations S7

2.2 Determination of effective magnetic moments in solution using Evans method S12

3. Synthesis of Substrates S19

4. Iron Catalyzed Asymmetric C(sp^3)–H Diamination of Carboxylic Acids S21

4.1 Diamination reaction by using chiral Fe(III) catalyst S38

4.2 Ligand recovering experiment S38

5. Mechanistic Experiments S40

5.1 Stepwise amination experiments with catalyst (*S*)-**Fe1**: 1st step S40

5.2 Stepwise amination experiments with catalyst (*S*)-**Fe1**: 2nd step S44

5.3 Decomposition experiment S46

5.4 Determination of *syn*/*anti*-diastereoselectivity and absolute configuration S47

6. Follow-Up Chemistry S51

6.1 Esterification S51

6.2 Synthesis of urea S52

6.3 Reduction of carboxylic acid S53

6.4 Synthesis of imidazoline S53

6.5 Synthesis of dipeptide S54

6.6 Synthesis of tripeptide S55

7. Single Crystal X-Ray Diffraction S57

8. Chiral HPLC analysis S61

9. NMR Spectra S96

Supplementary References S139

1. General Information

All catalytic reactions were carried out in Schlenk tubes from Synthware (10 mL) under an atmosphere of nitrogen with magnetic stirring. The mixture was degassed via two freeze-pump-thaw cycles. Solvents were distilled under nitrogen from calcium hydride (CH_3CN, CH_2Cl_2, $CHCl_3$), sodium/benzophenone (THF), Mg/I_2 (MeOH). All other reagents were commercially available and used without further purification. The catalysts (*S*,*S*)-**Fe3**[1] and (*R*)-**Fe5**[2] were prepared according to published procedures. Flash column chromatography was performed with silica gel 60 M from Macherey-Nagel (irregular shaped, 230-400 mesh, pH 6.8, pore volume: 0.81 mL × g^{-1}, mean pore size: 66 Å, specific surface: 492 m^2 × g^{-1}, particle size distribution: 0.5% < 25 µm and 1.7% > 71 µm, water content: 1.6%). ^1H NMR and proton decoupled ^{13}C NMR spectra were recorded on Bruker Avance 300 (300 MHz), Bruker AM (500 MHz) or Bruker AM (600 MHz) spectrometers at ambient temperature. NMR standards were used as follows: ^1H NMR spectroscopy: δ = 7.26 ppm ($CDCl_3$), 3.31 ppm (CD_3OD), and 2.05 ppm ($(CD_3)_2CO$). ^{13}C NMR spectroscopy: δ = 77.16 ppm ($CDCl_3$), 49 ppm (CD_3OD), 29.84, and 206.26 ppm ($(CD_3)_2CO$). High-resolution mass spectra were recorded on a Bruker En Apex Ultra 7.0 TFT-MS instrument using ESI/EI/FD/APCI techniques. Chiral HPLC chromatography was performed on Agilent 1200, Agilent 1260 with Daicel columns (Chiralpak IG, IC, IA, AD-H, OD-H, and IB-N5. all with particle size of 5 µm and column size of 4.6 × 250 mm). Voltammetric experiments were conducted with a computer-controlled Eco Chemie Autolab PGSTAT204 potentiostat in a Metrohm electrochemical cell. Optical rotations were measured on a Krüss P8000-T polarimeter with $[\alpha]_D^{22}$ values reported in degrees with concentrations reported in g/100 mL. IR spectra were recorded on a Brucker IFS 200 spectrometer. The location of the absorption bands is given in wavenumber $\tilde{\nu}$ in cm^{-1}. UV-Visible absorption spectroscopy was performed with a Cary 8454 UV-Vis spectrophotometer from Agilent with ChemStation software. Low temperature control was achieved using a cryostat from Unisoku Scientific Instruments. Quartz cells with cell paths of 1 cm were used.

2. Catalysts Synthesis and Characterization

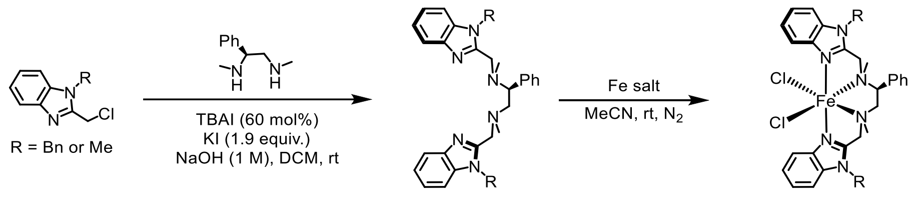

S1 **Ligand S2** (*S*)-**Fe cat.**

Step 1: To a solution of (*S*)-N^1, N^2-dimethyl-1-phenylethane-1,2-diamine[3] (1.0 equiv.), TBAI (60 mol%), KI (1.9 equiv.) and **S1**[1,4] (2.2 equiv.) in DCM (0.1 M) at room temperature was added NaOH (aqueous solution, 1 M). The reaction mixture was stirred at room temperature for 40 hours. After completion, the reaction mixture was diluted with sodium hydroxide (aqueous solution, 1 M, 4.0 equiv.) and was extracted with DCM for three times. The combined organic layer was dried over Na_2SO_4. After filtration, the solvent was evaporated under reduced pressure, and the residue was purified by column chromatography eluting with MeOH/DCM = 1/20 with 0.1% $NH_3•H_2O$ to afford the desired ligand **S2**.

Step 2: To a stirred solution of the ligand **S2** (1.1 equiv.) in MeCN (0.15 M) under N_2 atmosphere was added $FeCl_2•4H_2O$ (1.0 equiv. for Fe(II) complex) or $FeCl_3•6H_2O$ (1.0 equiv. for Fe(III) complex) in one portion. The reaction mixture was stirred at room temperature overnight before being diluted with diethyl ether (10 mL). The slurry was transferred into a centrifuge tube, and the solid material was separated by centrifugation and was washed with a mixed solvent of MeCN/Et_2O (v/v = 1/2.5) for three times (3×35 mL in total). The solid was dried under nitrogen flow to afford the desired iron complex.

(*S*)-N^1,N^2-bis((1-benzyl-1H-benzo[*d*]imidazol-2-yl)methyl)-N^1,N^2-dimethyl-1-phenylethane-1,2-diamine (S2-Bn)

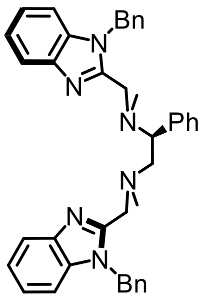

The title compound was obtained as a white gum in 63% yield (chromatography on silica gel, eluent: MeOH/DCM = 1/20 with 0.1% NH_3•H_2O). **^{1}H NMR** (300 MHz, $CDCl_3$) δ 7.83 – 7.69 (m, 2H), 7.32 – 7.11 (m, 12H), 7.11 – 6.99 (m, 3H), 6.96 – 6.85 (m, 4H), 6.82 – 6.73 (m, 2H), 5.37 (d, *J* = 16.5 Hz, 22.6 Hz, 2H), 4.79 (d, *J* = 16.5 Hz, 31.8 Hz, 2H), 3.80 – 3.53 (m, 5H), 3.13 – 2.72 (m, 2H), 2.24 (s, 3H), 2.06 (s, 3H). **^{13}C NMR** (75 MHz, $CDCl_3$) δ 152.1, 151.4, 142.5, 142.4, 138.3, 136.6, 136.2, 136.0, 128.83, 128.78, 128.1, 127.6, 127.5, 127.4, 126.31, 126.29, 122.9, 122.8, 122.1, 119.8, 110.0, 109.9, 66.4, 59.3, 56.2, 52.8, 47.1, 46.3, 43.4, 38.7. **HRMS** (+ESI) Exact mass calculated for $[C_{40}H_{41}N_6]^+$ $[(M + H)^+]$: 605.3387, found: 605.3376. $[\alpha]_D^{25} = -6.3^{\circ}$ (c 1.0, $CHCl_3$).

(*S*)-N^1,N^2-dimethyl-N^1,N^2-bis((1-methyl-1H-benzo[*d*]imidazol-2-yl)methyl)-1-phenylethane-1,2-diamine (S2-Me)

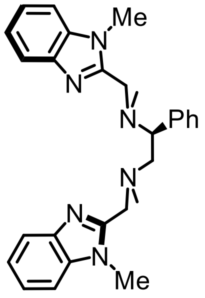

The title compound was obtained as a white gum in 80% yield (chromatography on silica gel, eluent: MeOH/DCM = 1/20 with 0.1% NH_3•H_2O). **^{1}H NMR** (300 MHz, $CDCl_3$) δ 7.71 – 7.57 (m, 2H), 7.23 – 7.11 (m, 6H), 7.09 – 6.99 (m, 3H), 6.94 – 6.88 (m, 2H), 3.64 (s, 2H), 3.58 (s, 6H), 3.11 – 2.98 (m, 4H), 2.80 (dd, *J* = 12.6, 4.9 Hz, 1H), 2.22 (s, 3H), 1.99 (s, 3H). **^{13}C NMR** (75 MHz, $CDCl_3$) δ 151.9, 151.3, 141.9, 141.7, 138.3, 136.2, 136.1, 128.7, 127.9, 127.2, 122.44, 122.37, 121.8, 121.7, 119.4, 109.1, 109.0, 66.1, 58.7, 55.6, 52.2, 43.2, 38.5, 29.9, 29.2. **HRMS** (+ESI) Exact mass calculated for $[C_{28}H_{32}N_6Na]^+$ $[(M + Na)^+]$: 475.2581, found: 475.2571. $[\alpha]_D^{25} = -4.1^{\circ}$ (c 0.5, $CHCl_3$).

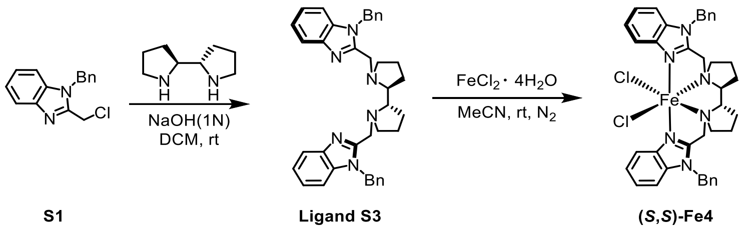

Step 1: To a solution of (2*S*,2'*S*)-2,2'-bipyrrolidine (1.0 equiv.) and **S1** (2.2 equiv.) in DCM (0.1 M) at room temperature was added NaOH (aqueous solution, 1 M). The reaction mixture was stirred at room temperature for 16 hours. After completion, the reaction mixture was diluted with sodium hydroxide (aqueous solution, 1.0 M, 4.0 equiv.) and was extracted with DCM for three times. The combined organic layer was dried over Na_2SO_4. After filtration, the solvent was evaporated under reduced pressure, and the residue was purified by column chromatography eluting with MeOH/DCM = 1/20 with 0.1% NH_3•H_2O to afford the desired ligand **S3**.

Step 2: To a stirred solution of the ligand **S3** (1.1 equiv.) in MeCN (0.15 M) under N_2 atmosphere was added $FeCl_2$•$4H_2O$ (1.0 equiv.) in one portion. The reaction mixture was stirred at room temperature overnight before being diluted with diethyl ether (10 mL). The slurry was transferred into a centrifuge tube, and the solid material was separated by centrifugation and was washed with a mixed solvent of MeCN/Et_2O (v/v = 1/2.5) for three times (3×35 mL in total). The solid was dried under nitrogen flow to afford the desired iron complex.

(*2S,2'S*)-1,1'-Bis((1-benzyl-1*H*-benzo[*d*]imidazol-2-yl)methyl)-2,2'-bipyrrolidine (S3)

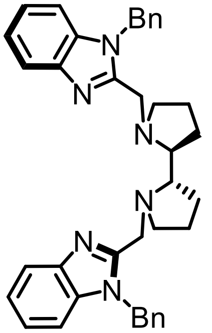

The title compound was obtained as a white gum in 85% yield (chromatography on silica gel, eluent: MeOH/DCM = 1/20 with 0.1% NH_3•H_2O). **^{1}H NMR** (300 MHz, $CDCl_3$) δ 8.40 (dd, *J* = 7.4, 1.3 Hz, 2H), 7.93 – 7.76 (m, 12H), 7.66 (dd, *J* = 7.1, 2.4 Hz, 4H), 6.22 – 6.03 (m, 4H), 4.78 (d, *J* = 13.4 Hz, 2H), 4.23 (d, *J* = 13.3 Hz, 2H), 3.48 (t, *J* = 7.8 Hz, 2H), 3.24 (t, *J* = 6.1 Hz, 2H), 2.93 (td, *J* = 9.7, 5.3 Hz, 2H), 2.37 – 2.05 (m, 8H). **^{13}C NMR** (75 MHz, $CDCl_3$) δ 152.3, 142.4, 136.3, 135.8, 128.8, 127.6, 126.1, 122.7, 122.0, 119.7, 109.8, 65.2, 55.5, 52.6, 46.9, 26.1, 24.0. **HRMS** (+ESI)

Exact mass calculated for $[C_{38}H_{41}N_6]^+$ $[(M + H)^+]$: 581.3387, found: 518.3376. $[\alpha]_D^{25}$ = +13.5° (c 1.0, $CHCl_3$).

2.1 Catalyst characterizations

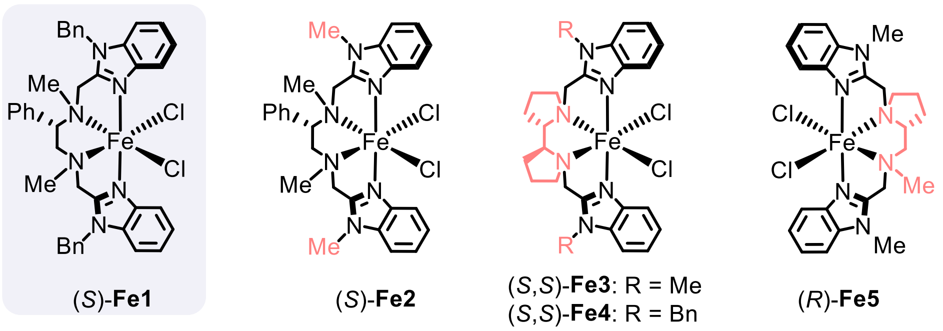

(*S*)-**Fe1** (*S*)-**Fe2** (*S,S*)-**Fe3**: R = Me (*S,S*)-**Fe4**: R = Bn (*R*)-**Fe5**

(*S,S*)-**Fe3** and (*R*)-**Fe5** were reported in previous works of our group[1,2]. (*S*)-**Fe1**, (*S*)-**Fe2**, and (*S,S*)-**Fe4** were newly synthesized and fully characterized in this work.

(*S*)-**Fe1**(II)

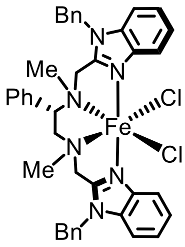

(*S*)-**Fe1**(II)

After the reaction of the ligand with iron salt ($FeCl_2$•$4H_2O$), the (*S*)-**Fe1**(II) was obtained as a yellow solid in 92% yield. **HRMS** (+ESI) Exact mass calculated for $[C_{40}H_{40}ClFeN_6]^+$ $[(M - Cl)^+]$: 695.2341, found: 695.2333. **IR** (ATR, $\tilde{\nu}$, cm^{-1}) 3091, 3023, 2939, 2285, 1641, 1502, 1478, 1440, 1386, 1354, 1033, 1006, 904, 863, 756, 719, 653, 432. **UV–vis** (CH_3OH) λmax (nm) (ε, M^{-1} cm^{-1}) 284.

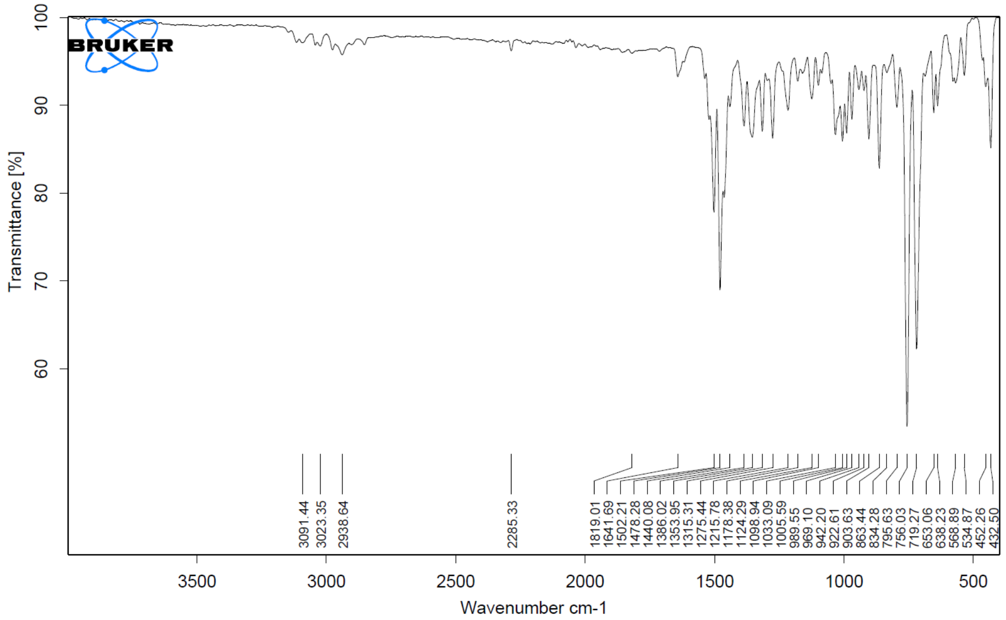

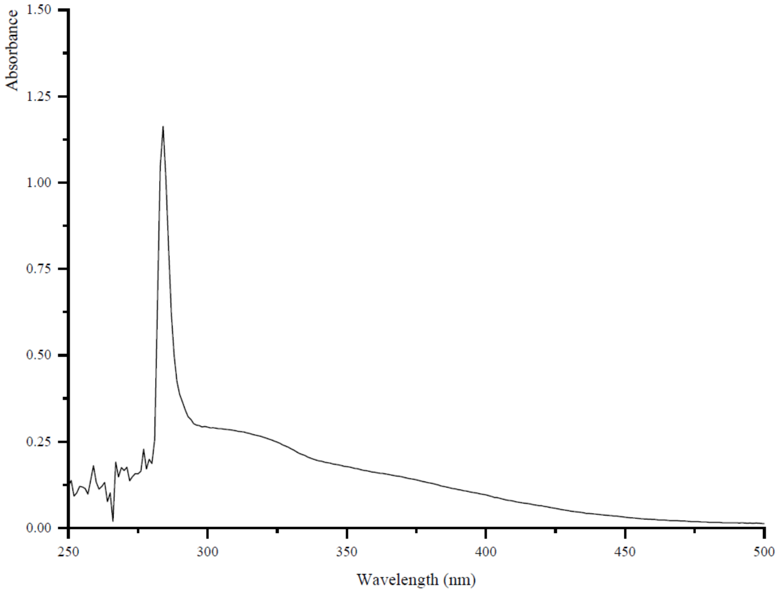

(*S*)-**Fe1**(III)

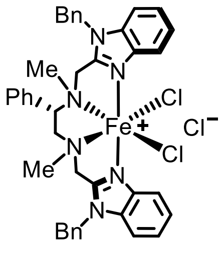

(*S*)-**Fe1**(III)

After the reaction of the ligand with iron salt ($FeCl_3$•$6H_2O$), the (*S*)-**Fe1**(III) was obtained as a yellow solid in 87% yield. **HRMS** (+ESI) Exact mass calculated for $[C_{40}H_{40}Cl_2FeN_6]^+$ $[(M - Cl)^+]$: 730.2030, found: 730.2021. **IR** (ATR, $\tilde{\nu}$, cm^{-1}) 3385, 3082, 2934, 1546, 1454, 1388, 1339, 1269, 1185, 1105, 1032, 971, 878, 753, 716, 617, 528. **UV–vis** (CH_3OH) λmax (nm) (ε, M^{-1} cm^{-1}) 283.

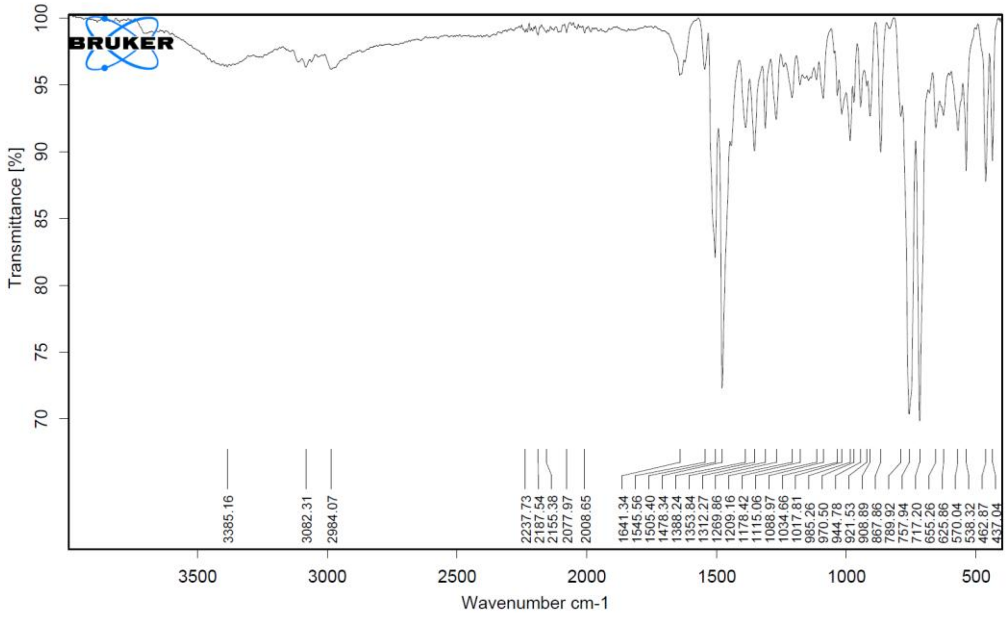

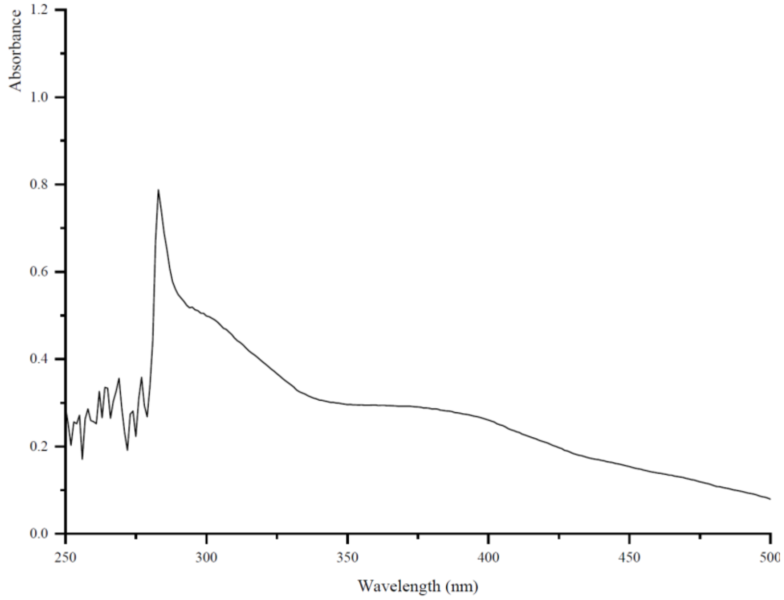

(*S*)-**Fe2**

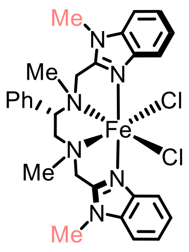

(*S*)-**Fe2**

After the reaction of the ligand with iron salt ($FeCl_2$•$4H_2O$), the (*S*)-**Fe2** was obtained as a yellow solid in 88% yield. **HRMS** (+ESI) Exact mass calculated for $[C_{28}H_{32}ClFeN_6]^+$ $[(M - Cl)^+]$: 543.1715, found: 543.1714. **IR** (ATR, $\tilde{\nu}$, cm^{-1}) 3114, 3076, 2953, 1609, 1547, 1516, 1467, 1388, 1316, 1243, 1187, 1109, 1027, 983, 861, 779, 716. **UV–vis** (CH_3OH) λmax (nm) (ε, M^{-1} cm^{-1}) 284.

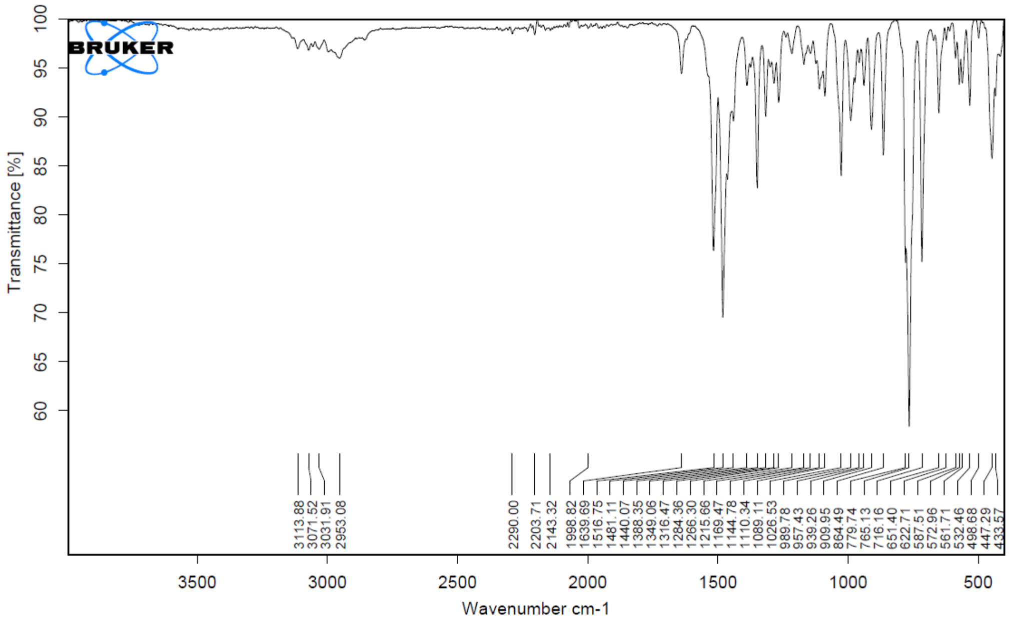

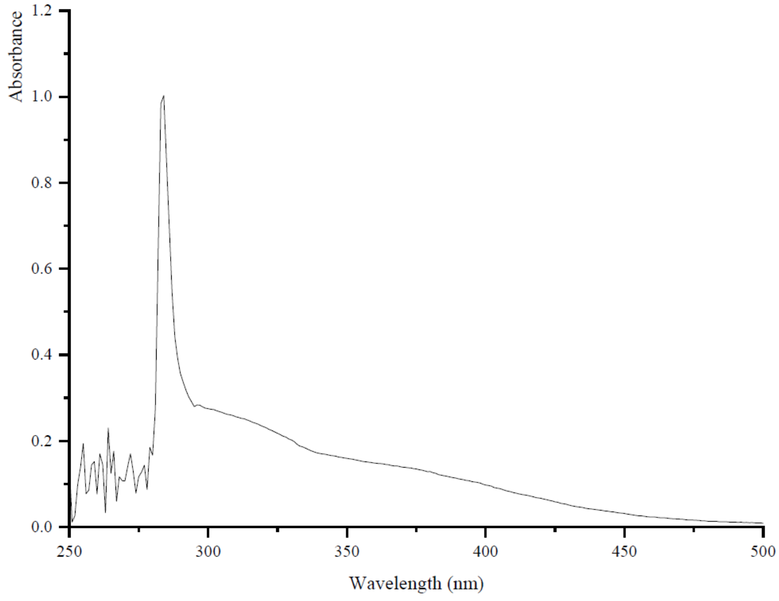

(*S*,*S*)-**Fe4**

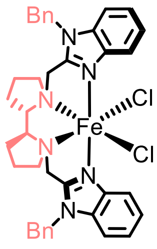

(*S*,*S*)-**Fe4**

After the reaction of the ligand with iron salt ($FeCl_2$•$4H_2O$), the (*S*,*S*)-**Fe4** was obtained as a yellow solid in 83% yield. **HRMS** (+ESI) Exact mass calculated for $[C_{38}H_{40}ClFeN_6]^+$ $[(M - Cl)^+]$: 671.2341, found: 671.2333. **IR** (ATR, $\tilde{\nu}$, cm^{-1}) 3437, 3113, 3006, 1639, 1541, 1510, 1473, 1389, 1314, 1240, 1158, 1087, 1017, 963, 864, 754, 711, 594, 532. **UV–vis** (CH_3OH) λmax (nm) (ε, M^{-1} cm^{-1}) 283.

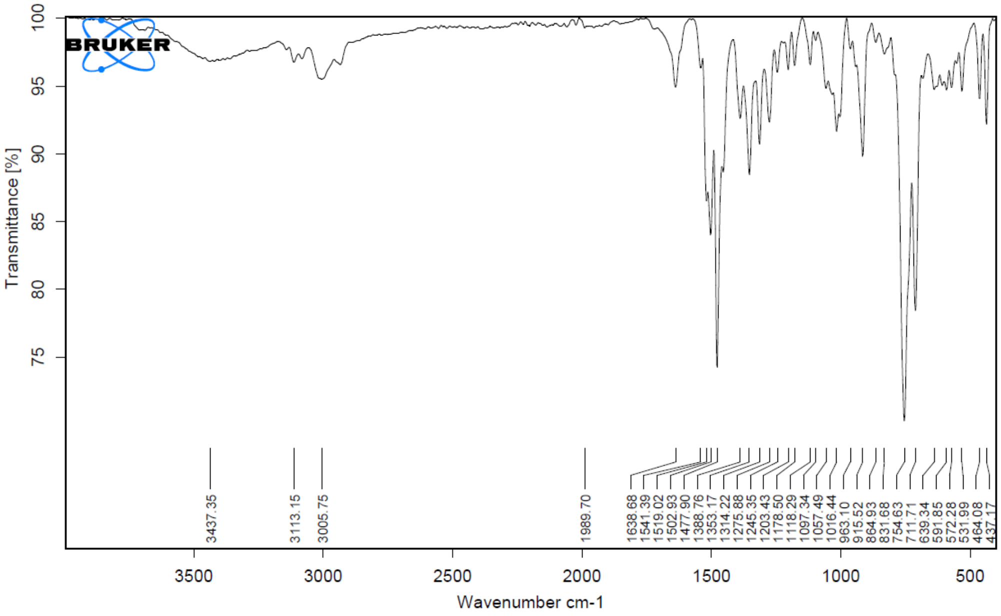

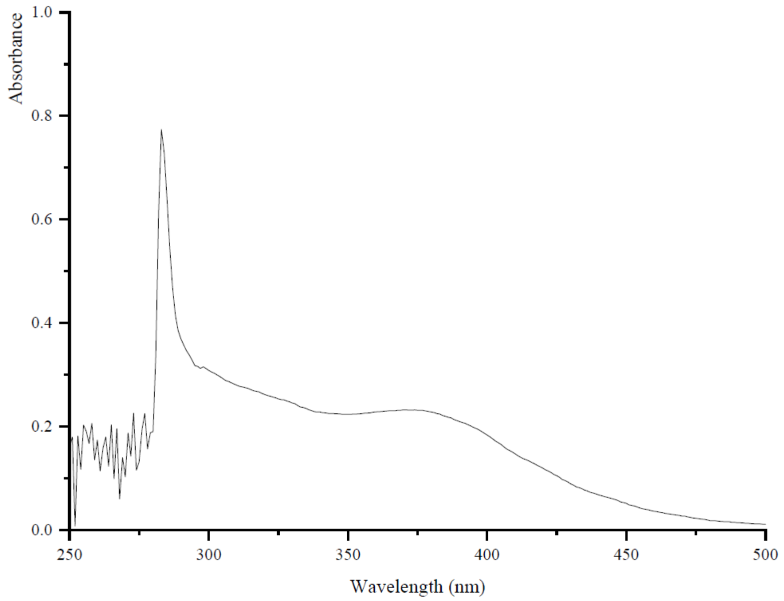

2.2 Determination of effective magnetic moments in solution using Evans method

Theory. Magnetic susceptibilities were traditionally measured by a Gouy balance and nowadays often by using a magnetometer SQUID. It was proposed by Evans[5] to determine it by measuring the change in chemical shift of an indicator compound caused by introducing a known concentration of the paramagnetic compound into the solution. It was found by Dickinson[6] that the change in the chemical shift of the ^{1}H signals of inert reference molecules in solution caused by paramagnetic substances could be accurately related to the magnetic susceptibility of the paramagnetic substances. The shift was termed bulk magnetic susceptibility shift (BMS) and can be expressed as follows:[7]

$$\Delta_{\chi} = \frac{4\pi cs}{T}\left(\frac{\mu_{eff}}{2.84}\right)^2 \times 10^3 \qquad (1)$$

In equation (1), c is the concentration of the paramagnetic solute in mol/l, s is the shape factor which equals 1/3, -1/6 and 0 for a cylinder parallel to the external magnetic field, a cylinder perpendicular to the external magnetic field and a sphere, respectively, T is the absolute temperature and μ_{eff} is the effective magnetic moment of the paramagnetic ion given in Bohr magneton μ_B. The BMS shift $\Delta\chi$ is given in ppm, the same as for the normal chemical shift. In the state-of-the-art NMR spectrometers, sample tubes are parallel to the external magnetic field, equation (1) becomes:

$$\Delta_{\chi} = \frac{4\pi c}{3T}\left(\frac{\mu_{eff}}{2.84}\right)^2 \times 10^3 \qquad (2)$$

The primary parameter to describe the paramagnetism of a substance is the paramagnetic susceptibility χ, which varies with temperature according to the Curie Law:[8]

$$\chi = \frac{C}{T} \qquad (3)$$

Where C is the Curie constant and can be expressed as:

$$C = \frac{Ng^2\mu_B^2 S(S+1)}{3k} \qquad (4)$$

The constants in equation (4) are as follows:

N: Avogadro's number and N = 6.02 × 10^{23} mol^{-1};

g: Landé constant of electron;

μ_B: Bohr magneton and μ_B = 9.27 × 10^{-24} JT^{-1}

S: the total spin quantum number of the unpaired electrons in the paramagnetic ion;

K: Boltzmann constant and k = 1.38 × 10^{-23} JK^{-1}.

The magnitude of χ is an inconvenient number, chemists often report the effective magnetic moment μ_{eff}, which is defined as:

$$\chi_M = \frac{N\mu_{eff}^2}{3kT} \qquad (5)$$

A combination of equations (3) – (5) leads to:

$$\mu_{eff} = \sqrt{g^2 S(S+1)}\mu_B = 2\sqrt{S(S+1)}\mu_B = \sqrt{n(n+2)}\mu_B \qquad (6)$$

A combination of equations (2) and (6) and setting the temperature of 298 K give us the following:

$$\mu_{eff}^2 = n(n+2) = \frac{0.57\Delta_\chi}{c} \qquad (7)$$

In equation (7) n is the number of unpaired electrons, Δχ the observed BMS shift in ppm, and c the concentration of the paramagnetic substance in mol/l. Therefore, with equation (7), by measuring Δχ the effective magnetic moment and number of unpaired electrons can be calculated. Therefore, spin state of a paramagnetic substance can be determined.

Experimental. Samples for NMR measurements were 5–15 mg of the substance dissolved in 0.5 mL of methanol-d_4 containing 0.5 % (v/v) dichloromethane and filled into 5 mm NMR tubes (Wilmad). The ^{1}H NMR spectra were taken on a Bruker Avance-III HD 500 MHz spectrometer installed with a 5 mm inverse TBI probe. For the Evans experiment the 5 mm NMR sample tubes (Wilmad) were added with a 3 mm coaxial insert filled with the same solvent (from a stock solution of CD_3OD + 0.5 % CH_2Cl_2). The filling amount of the coaxial insert was adjusted to be of similar height as that of the outer tube. The amount of the paramagnetic substance used was adjusted to be within sample saturation and the final volumes were carefully recorded. In this way, sample concentrations were calculated with the least experimental error. ^{1}H spectra were recorded at 298 K with spectral width of 16 ppm, relaxation delay of 3 s and 16 transients. Temperature was calibrated with a Bruker standard of 4 % methanol in methanol-d_4. Chemical shifts were referenced with the rest signal of methanol-d_4 within the coaxial insert.

Results and Discussion. Our initial purpose was to characterize the oxidation state of the iron catalyst **Fe1**. The Evans method is one popular means of characterizing properties of paramagnetic complexes in solution. Nevertheless, sources such as accuracy in concentration of the paramagnetic solute and any paramagnetic impurities may lead to relatively high error of this method.[9] This situation may be more severe when air-sensitive paramagnetic complexes are concerned.[9,10] Taking advantage of the inert property of our iron complexes, strict inert atmosphere was not necessary and the sample handling was very straightforward. Thus, Evans measurements were done on all of the catalysts presented in this paper for a better comparison. The measurement conditions, the BMS shifts used for the calculation, and the determined effective magnetic moment and the number of unpaired electrons are summarized in **Table S1**. Thus, the largest effective magnetic moment of 5.4 μ_B was observed for (*S*)-**Fe1**(III), which indicates a 70 % high-spin state for an Fe(III) in its d^5 electron configuration. The observed effective magnetic moment for the rest of the complexes consistent with an oxidation state of Fe(II) in high-spin state of d^6 electron configuration. The only exception was (*S,S*)-**Fe4**, whereby an oxidation state of Fe(II) with an almost 50/50 mixed high-spin/low-spin state was observed.

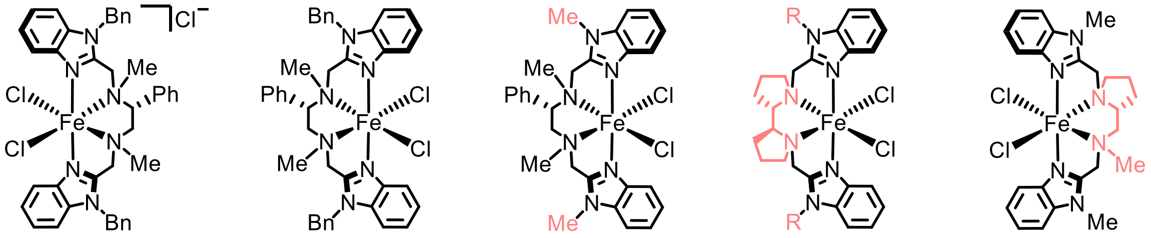

(R)-**Fe1**(III) | (S)-**Fe1**(II) | (S)-**Fe2**(II) | (S,S)-**Fe3**(II): R = Me; (S,S)-**Fe4**(II): R = Bn | (R)-**Fe5**(II)

Table S1. Results of Evans measurement at 298 K

Catalyst	Δχ (ppm)				C (mM)	μ_{eff} (μ_B)	n
	CH_2Cl_2	CD_3OH	CD_2HOD	ave			
(R)-**Fe1**(III)	2.014	2.024	2.058	2.032	39.53	5.41	4.5
(S)-**Fe1**(II)	1.154	1.190	1.186	1.177	25.81	5.10	4.0
(S)-**Fe2**(II)	0.598	0.618	0.613	0.610	14.26	4.94	4.0
(S,S)-**Fe3**(II)	0.527	0.534	0.542	0.537	15.38	4.46	3.6
(S,S)-**Fe4**(II)	0.506	0.528	0.525	0.520	18.92	3.96	3.0
(R)-**Fe5**(II)	0.210	0.227	0.222	0.220	5.21	4.90	4.0

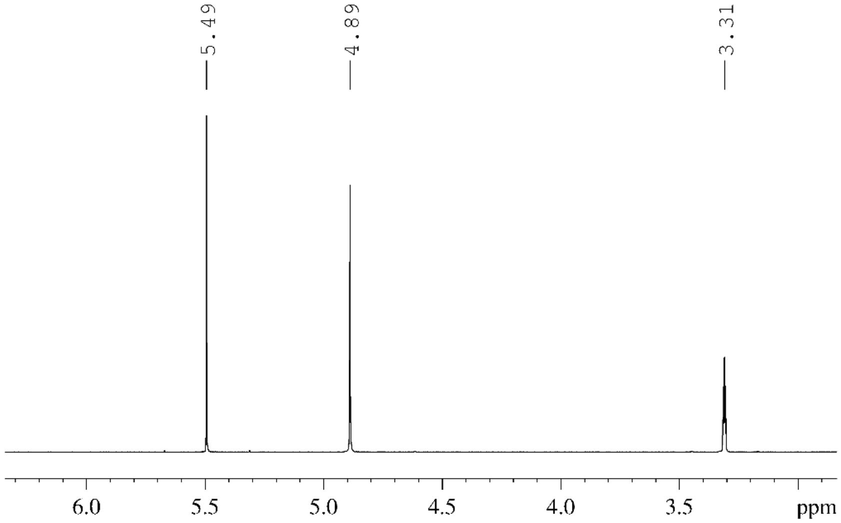

Figure S1. Evans experiment at 298 K: ^{1}H spectrum (500.13 MHz) of solvent CD_3OD with 0.5 % CH_2Cl_2 in coaxial insert capillary, as a control spectrum for reference.

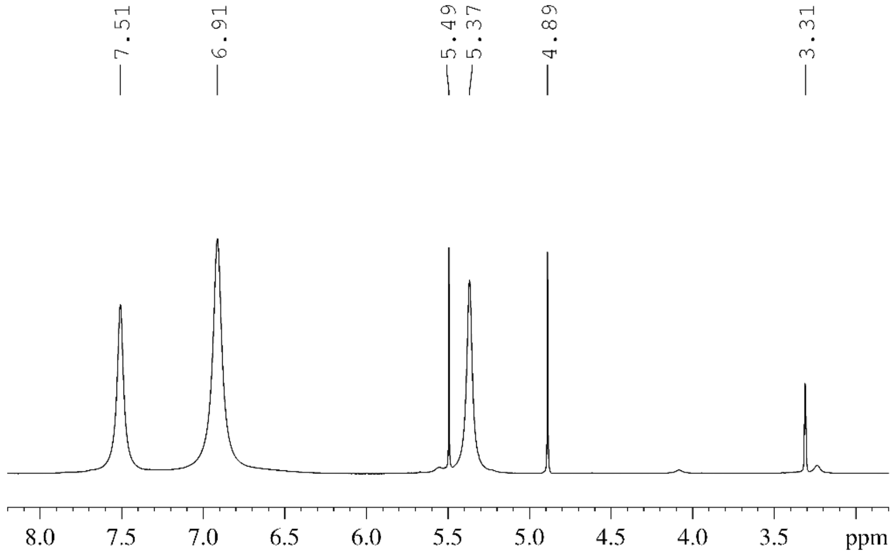

Figure S2. Evans experiment at 298 K: ^{1}H spectrum (500.13 MHz) of 39.5 mM (*R*)-**Fe1**(III) in CD_3OD with 0.5 % CH_2Cl_2. The sharp signals are due to solvents in the coaxial insert capillary.

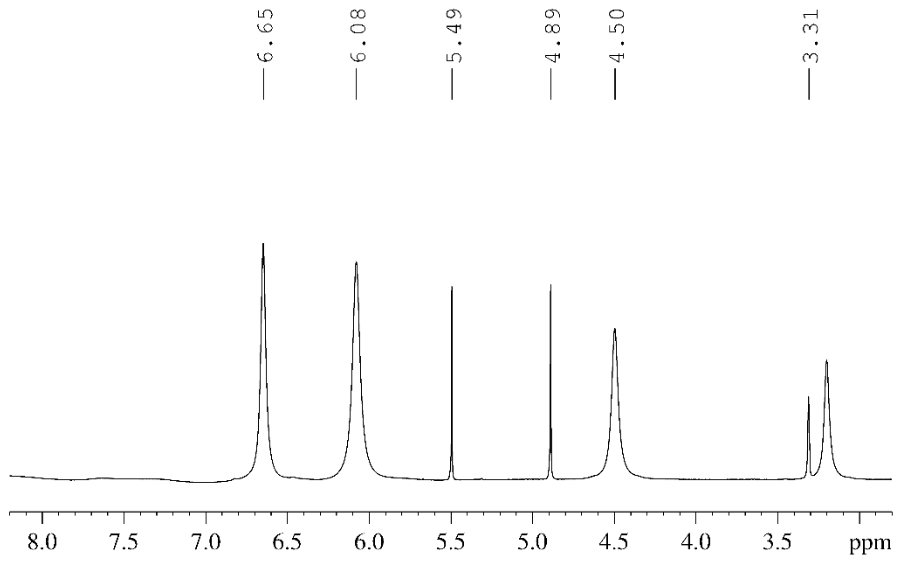

Figure S3. Evans experiment at 298 K: ^{1}H spectrum (500.13 MHz) of 25.8 mM (*S*)-**Fe1**(II) in CD_3OD with 0.5 % CH_2Cl_2. The sharp signals are due to solvents in the coaxial insert capillary.

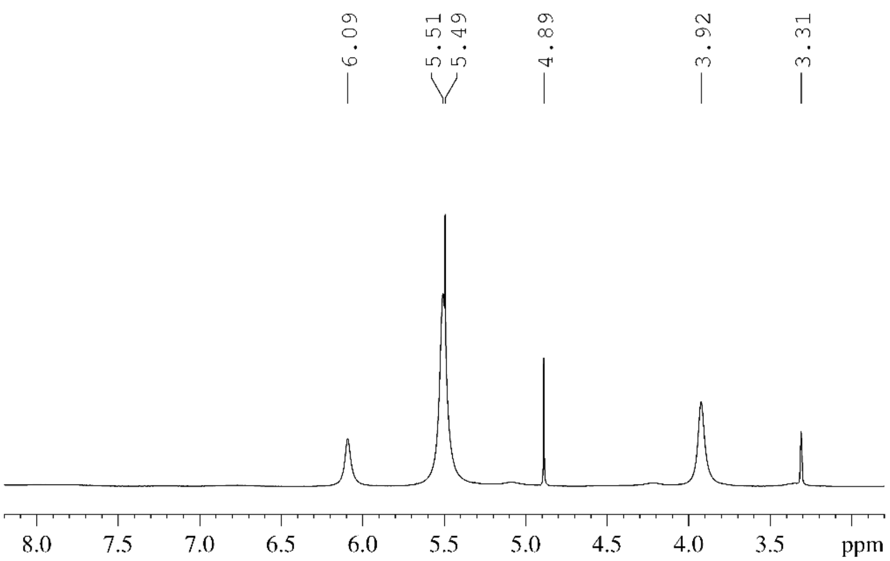

Figure S4. Evans experiment at 298 K: ^{1}H spectrum (500.13 MHz) of 14.3 mM (*S*)-**Fe2**(II) in CD_3OD with 0.5 % CH_2Cl_2. The sharp signals are due to solvents in the coaxial insert capillary.

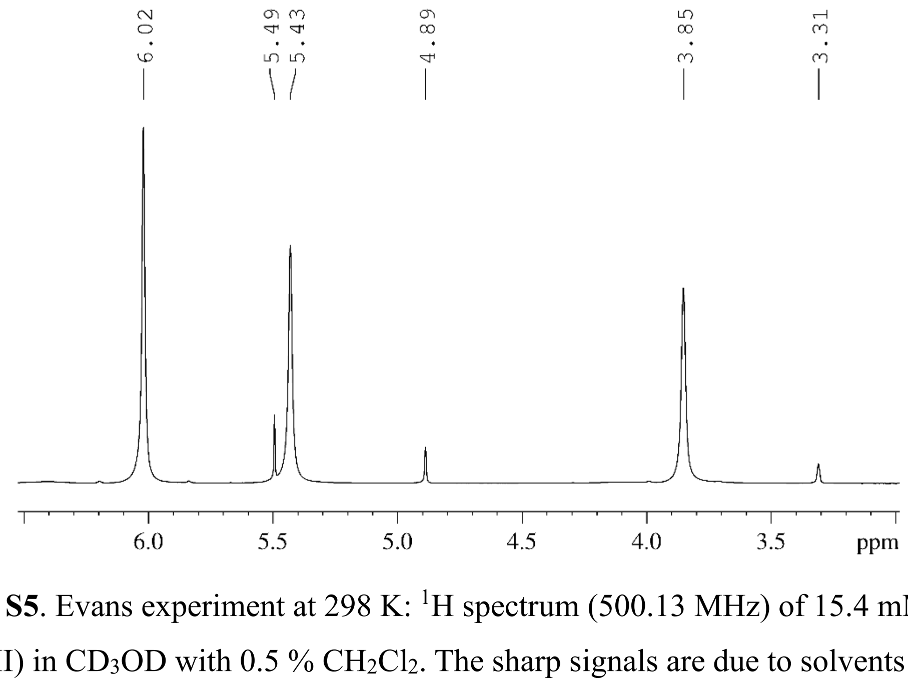

Figure S5. Evans experiment at 298 K: ^{1}H spectrum (500.13 MHz) of 15.4 mM (*S,S*)-**Fe3**(II) in CD_3OD with 0.5 % CH_2Cl_2. The sharp signals are due to solvents in the coaxial insert capillary.

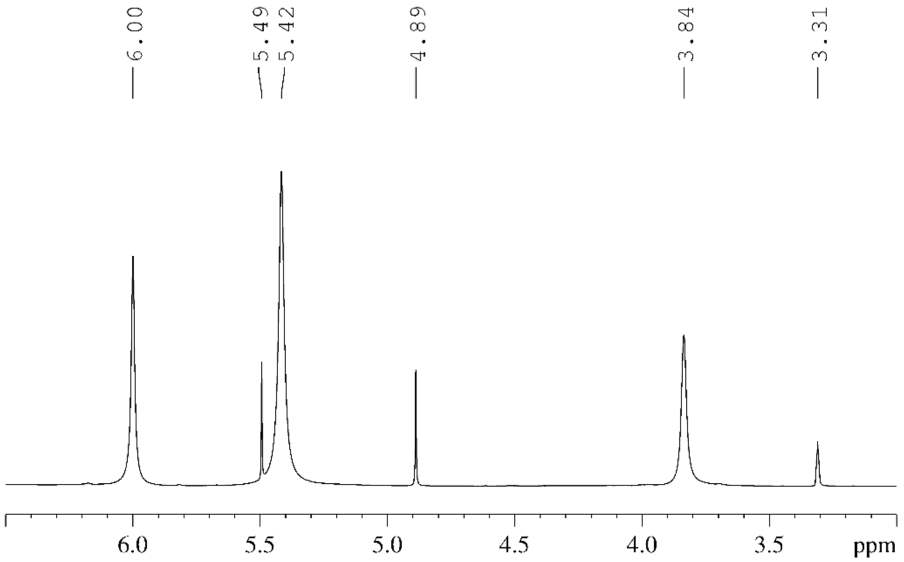

Figure S6. Evans experiment at 298 K: ^{1}H spectrum (500.13 MHz) of 18.9 mM (*S,S*)-**Fe4**(II) in CD_3OD with 0.5 % CH_2Cl_2. The sharp signals are due to solvents in the coaxial insert capillary.

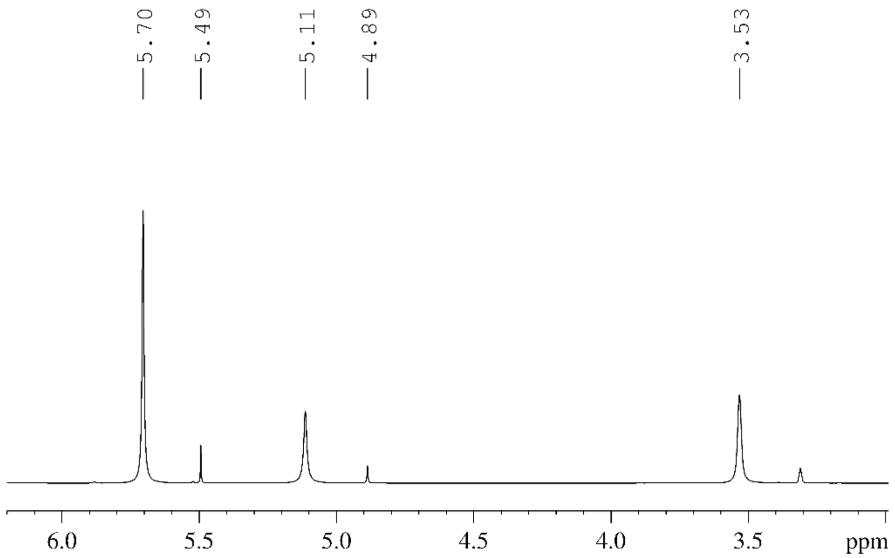

Figure S7. Evans experiment at 298 K: ^{1}H spectrum (500.13 MHz) of 5.2 mM (*R*)-**Fe5**(II) in CD_3OD with 0.5 % CH_2Cl_2. The sharp signals are due to solvents in the coaxial insert capillary.

3. Synthesis of Substrates

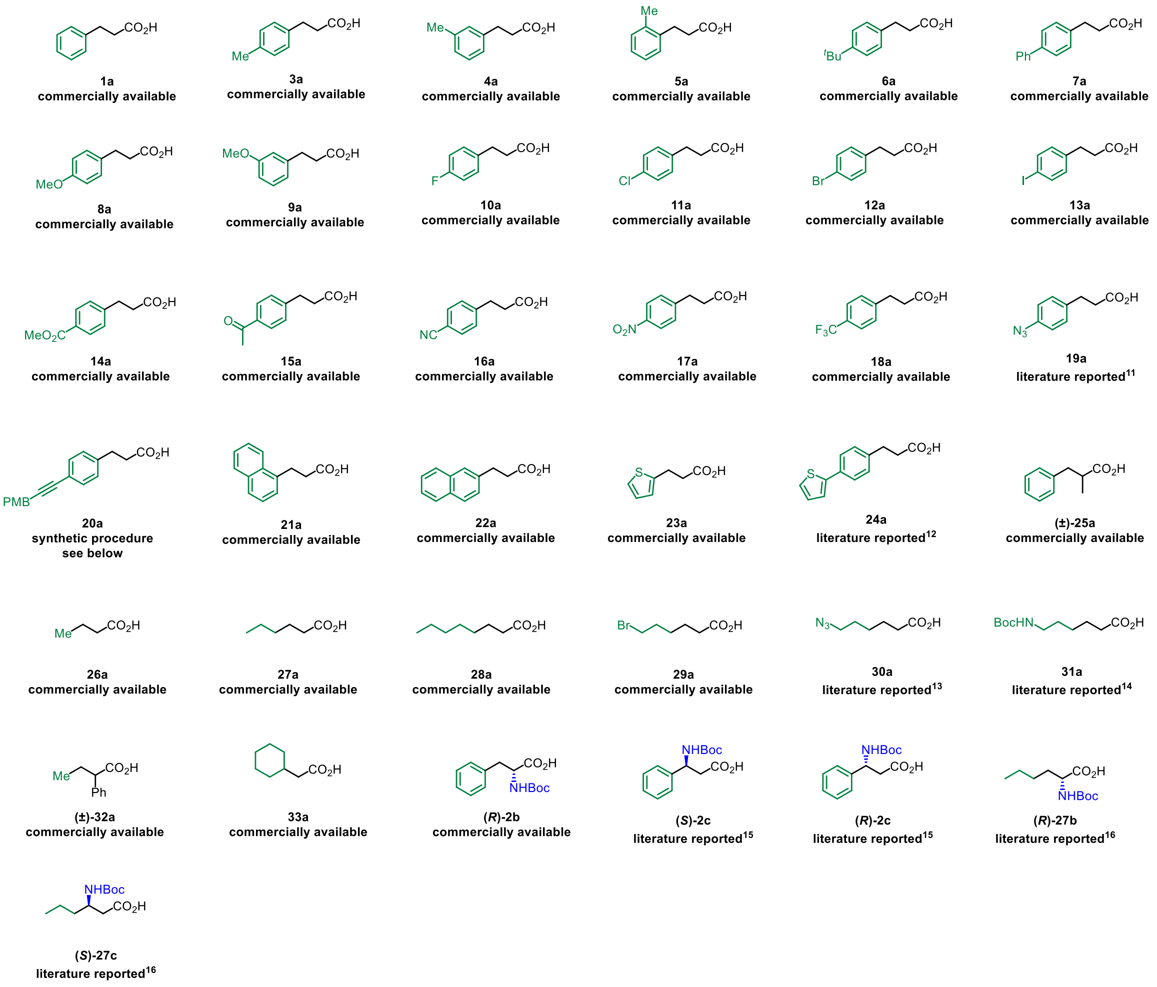

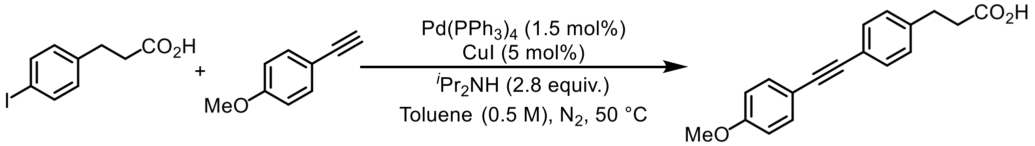

To a 25 mL flask containing 3-(4-iodophenyl)propanoic acid (1.38 g, 5 mmol, 1.0 equiv.), $Pd(PPh_3)_4$ (87 mg, 0.075 mmol, 1.5 mol%) and CuI (48 mg, 0.25 mmol, 5 mol%) was added toluene (10 mL, 0.5 M), ${}^{i}Pr_2NH$ (2.0 mL, 14 mmol, 2.8 equiv.) and 1-ethynyl-4-methoxybenzene (780 μL, 6.0 mmol, 1.2 equiv.) under N_2 atmosphere. The mixture was heated to 50 °C overnight. After cooling to room temperature, the reaction was quenched with 1.0 M NaOH solution (40 mL). The aqueous phase was washed three times with EA

(3×25 mL), then acidified to pH = 1 with HCl (conc.). The aqueous phase was extracted three times with EA (3×30 mL), and the combined organic phase was dried over anhydrous Na_2SO_4, concentrated under vacuum. The residue was purified by flash column chromatography eluenting with EtOAc/n-hexane (1:5 to 1:2) to afford 3-(4-(3-(4-methoxyphenyl)prop-1-yn-1-yl)phenyl)propanoic acid **20a** as white foam (912.5 mg, 62% yield). **^{1}H NMR** (300 MHz, CD_3OD) δ 7.50 – 7.38 (m, 4H), 7.32 – 7.22 (m, 2H), 6.99 – 6.89 (m, 2H), 3.84 (d, J = 1.5 Hz, 3H), 2.96 (t, J = 7.6 Hz, 2H), 2.64 (t, J = 7.6 Hz, 2H). **^{13}C NMR** (75 MHz, CD_3OD) δ 176.53, 161.24, 142.44, 133.92, 132.43, 129.52, 122.81, 116.75, 115.14, 89.80, 88.72, 55.79, 36.45, 31.87. **HRMS** (+ESI) Exact mass calculated for [$C_{19}H_{18}NaO_3$:$^+$ [(M + Na)$^+$]: 317.1148, found: 317.1156.

4. Iron Catalyzed Asymmetric C(sp^3)–H Diamination of Carboxylic Acids

General procedure: To a dry Schlenk tube was added the carboxylic acid (0.1 mmol, 1.0 equiv.), BocNHOMs[17] (10.0 equiv.) and (*S*)-**Fe1** (15 mol%). The tube was evacuated and backfilled with N_2 for five times. $CHCl_3$ (1.0 mL, 0.1 M) was added via syringe while the Schlenk tube was kept in liquid nitrogen. Then piperidine (120 µL, 12.0 equiv.) was added and the tube was sealed. The reaction mixture was further degassed twice via freeze-pump-thaw. Then the reaction mixture was stirred at –15 ºC for 64 h. After completion, the reaction mixture was diluted with 30 mL Et_2O and was washed with aqueous $NaHSO_4$ (1 M) twice (2×10 mL) to remove the majority of iron complex from the Et_2O layer. The combined aqueous layer was extracted with Et_2O (2×30 mL). The combined organic layer was dried over Na_2SO_4. After filtration, the solvent was evaporated under reduced pressure and the residue was purified by column chromatography on silica gel using the indicated solvent as the eluent. Note: Thin-layer chromatography was conducted in a mixed solvent of $MeOH/CH_2Cl_2$ = 1/4 with 0.2% NH_3•H_2O, in which the *in situ* formed ammonium salt of *N*-Boc-protected diamino acid is always less polar than the ammonium salt of the starting carboxylic acid.

(2*R*,3*R*)-2,3-bis((*tert*-butoxycarbonyl)amino)-3-phenylpropanoic acid (2a)

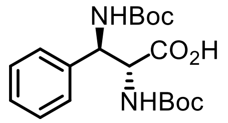

Catalyzed by 15 mol% (*S*)-**Fe1**, the diamination of **1a** (15.1 mg, 0.1 mmol) gave **2a** as a white foam (chromatography on silica gel, eluent: EtOAc/*n*-hexane = 1/6 with 0.2% HOAc, 25.8 mg, 68% yield) with 95% e.e. Diastereoselectivity was determined by ^{1}H NMR analysis of the isolated mixture as 12:1. [DAICEL CHIRALPAK IG column reverse phase, Agilent HPLC 1260, MeCN/Water (0.1% TFA) = 30/70 (v/v), 1.0 mL/min, 23 ºC, 210 nm; t_1 = 24.6 min, t_2 = 85.5 min]: $[\alpha]_D^{25} = -31.6^{\circ}$ (c 0.5, MeOH, 95% e.e.); **^{1}H NMR** (300 MHz, CD_3OD) δ 7.38 – 7.21 (m, 5H), 5.26 (d, *J* = 3.9 Hz, 0.09H, minor), 5.02 (d, *J* = 4.6 Hz, 1.08 H, major), 4.51 (d, *J* = 6.9 Hz, 1H), 1.45 – 1.34 (m, 18H). **^{13}C NMR** (75 MHz, CD_3OD) δ 173.4, 157.6, 157.4, 140.1, 129.3, 128.7, 128.5, 80.8, 80.6, 58.8, 57.5, 28.7, 28.6. **HRMS** (+ESI)

Exact mass calculated for $[C_{19}H_{28}N_2O_6Na]^+$ $[(M + Na)^+]$: 403.1840, found: 403.1830.

(2*R*,3*R*)-2,3-bis((*tert*-butoxycarbonyl)amino)-3-(*p*-tolyl)propanoic acid (3)

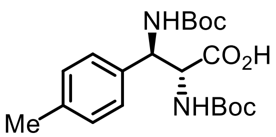

Catalyzed by 15 mol% (*S*)-**Fe1**, the diamination of **3a** (16.4 mg, 0.1 mmol) gave **3** as a white foam (chromatography on silica gel, eluent: EtOAc/*n*-hexane = 1/6 with 0.2% HOAc, 22.1 mg, 56% yield) with 93% e.e. Diastereoselectivity was determined by ^{1}H NMR analysis of the isolated mixture as >20:1. [DAICEL CHIRALPAK AD-H column, Agilent HPLC 1260, *i*PrOH/*n*-hexane = 5/95 (v/v) with 0.1% TFA, 1.0 mL/min, 25 ºC, 210 nm; t_1 = 25.7 min, t_2 = 32.9 min]: $[\alpha]_D^{25}$ = +31.5° (*c* 1.0, MeOH, 93% e.e.); **^{1}H NMR** (300 MHz, CD_3OD) δ 7.34 – 6.99 (m, 4H), 4.98 (d, *J* = 7.0 Hz, 1H), 4.50 (d, *J* = 7.1 Hz, 1H), 2.31 (s, 3H), 1.47 – 1.26 (m, 18H). **^{13}C NMR** (75 MHz, CD_3OD) δ 173.5, 157.6, 157.4, 138.5, 137.0, 129.9, 128.3, 80.8, 80.5, 59.0, 57.2, 28.7, 28.6, 21.1. **HRMS** (+ESI) Exact mass calculated for $[C_{20}H_{30}N_2O_6Na]^+$ $[(M + Na)^+]$: 417.1996, found: 417.1986.

(2*R*,3*R*)-2,3-bis((*tert*-butoxycarbonyl)amino)-3-(m-tolyl)propanoic acid (4)

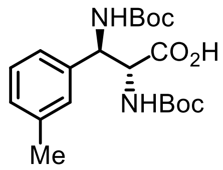

Catalyzed by 15 mol% (*S*)-**Fe1**, the diamination of **4a** (16.4 mg, 0.1 mmol) gave **4** as a white foam (chromatography on silica gel, eluent: EtOAc/*n*-hexane = 1/6 with 0.2% HOAc, 25.8 mg, 65% yield) with 92% e.e. Diastereoselectivity was determined by ^{1}H NMR analysis of the isolated mixture as 7:1. [DAICEL CHIRALPAK IG column reverse phase, Agilent HPLC 1260, MeCN/Water (0.1% TFA) = 23/77 (v/v) with 0.1% TFA, 1.0 mL/min, 20 ºC, 210 nm; t_1 = 124.7 min, t_2 = 227.4 min]: $[\alpha]_D^{25}$ = -11.5° (*c* 1.0, MeOH, 92% e.e.); **^{1}H NMR** (300 MHz, CD_3OD) δ 7.29 – 7.00 (m, 4H), 5.23 (d, *J* = 4.7 Hz, 0.14H, minor), 4.98 (d, *J* = 7.3 Hz, 0.94H, major), 4.62 – 4.36 (m, 1H), 2.33 (s, 3H), 1.46 – 1.31 (m, 18H). **^{13}C NMR** (75 MHz, CD_3OD) δ 173.5, 157.6, 157.4, 140.2, 139.0, 129.3, 129.2, 129.11 (major), 128.44 (minor), 125.47 (major), 124.87 (minor), 80.8, 80.6, 59.60 (minor), 58.90 (major), 57.44 (major), 57.08 (minor), 28.7, 28.6, 21.54 (minor), 21.51 (major). **HRMS** (+ESI) Exact mass calculated for $[C_{20}H_{30}N_2O_6Na]^+$ $[(M + Na)^+]$: 417.1996, found: 417.1989.

(2*R*,3*R*)-2,3-bis((*tert*-butoxycarbonyl)amino)-3-(o-tolyl)propanoic acid (5)

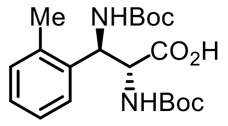

Catalyzed by 15 mol% (*S*)-**Fe1**, the diamination of **5a** (20.6 mg, 0.1 mmol) gave **5** as a white foam (chromatography on silica gel, eluent: EtOAc/*n*-hexane = 1/6 with 0.2% HOAc, 27.3 mg, 69% yield) with 92% e.e. Diastereoselectivity was determined by ^{1}H NMR analysis of the isolated mixture as 10:1. [DAICEL CHIRALPAK IB-N5 column reverse phase, Agilent HPLC 1260, MeCN/Water (0.1% TFA) = 25/75 (v/v), 1.0 mL/min, 25 ºC, 210 nm; t_1 = 89.3 min, t_2 = 99.7 min]: $[\alpha]_D^{25}$ = -10.5° (*c* 1.0, MeOH, 92% e.e.); **^{1}H NMR** (300 MHz, CD_3OD) δ 7.39 – 7.25 (m, 1H), 7.14 (d, *J* = 2.8 Hz, 3H), 5.51 (d, *J* = 4.3 Hz, 0.1H, minor), 5.25 (d, *J* = 7.9 Hz, 1H, major), 4.63 – 4.40 (m, 1H), 2.44 (s, 3H), 1.44 – 1.31 (m, 18H). **^{13}C NMR** (75 MHz, CD_3OD) δ 173.7, 157.3, 138.4, 137.5, 131.4, 128.5, 127.7, 127.0, 80.7, 80.5, 57.9, 53.4, 28.7, 28.6, 19.6. **HRMS** (+ESI) Exact mass calculated for $[C_{20}H_{30}N_2O_6Na]^+$ $[(M + Na)^+]$: 417.1996, found: 417.1998.

(2*R*,3*R*)-2,3-bis((*tert*-butoxycarbonyl)amino)-3-(4-(*tert*-butyl)phenyl)propanoic acid (6)

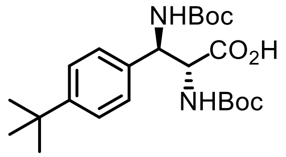

Catalyzed by 15 mol% (*S*)-**Fe1**, the diamination of **6a** (20.6 mg, 0.1 mmol) gave **6** as a white foam (chromatography on silica gel, eluent: EtOAc/*n*-hexane = 1/6 with 0.2% HOAc, 26.6 mg, 61% yield) with 98% e.e. Diastereoselectivity was determined by ^{1}H NMR analysis of the isolated mixture as >20:1. [DAICEL CHIRALPAK IA column, Agilent HPLC 1260, *i*PrOH/*n*-hexane = 5/95 (v/v) with 0.1% TFA, 0.8 mL/min, 25 ºC, 210 nm; t_1 = 27.7 min, t_2 = 31.6 min]: $[\alpha]_D^{25}$ = -13.6° (*c* 1.0, MeOH, 98% e.e.); **^{1}H NMR** (300 MHz, CD_3OD) δ 7.35 (d, *J* = 8.2 Hz, 2H), 7.24 (d, *J* = 7.9 Hz, 2H), 4.99 (d, *J* = 7.2 Hz, 1H), 4.57 – 4.34 (m, 1H), 1.46 – 1.31 (m, 18H), 1.30 (s, 9H). **^{13}C NMR** (75 MHz, CD_3OD) δ 173.1 (d, *J* = 44.25 Hz, 1C), 157.7, 151.5 (d, *J* = 15.75 Hz, 1C), 137.5, 128.2, 127.5, 126.2, 80.7, 80.6, 59.3 (d, *J* = 47.25 Hz, 1C), 57.1 (d, *J* = 30.75 Hz, 1C), 35.3, 31.8, 28.7, 28.6. **HRMS** (+ESI) Exact mass calculated for $[C_{23}H_{36}N_2O_6Na]^+$ $[(M + Na)^+]$: 459.2466, found: 459.2449.

(2*R*,3*R*)-3-([1,1'-biphenyl]-4-yl)-2,3-bis((*tert*-butoxycarbonyl)amino)propanoic acid (7)

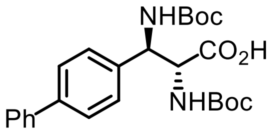

Catalyzed by 15 mol% (*S*)-**Fe1**, the diamination of **7a** (22.6 mg, 0.1 mmol) gave **7** as a white foam (chromatography on silica gel, eluent: EtOAc/*n*-hexane = 1/6 with 0.2% HOAc, 32.0 mg, 70% yield) with 92% e.e. Diastereoselectivity was determined by ^{1}H NMR analysis of the isolated mixture as 4:1. [DAICEL CHIRALPAK IA column, Agilent HPLC 1260, *i*PrOH/*n*-hexane = 10/90 (v/v) with 0.1% TFA, 1.0 mL/min, 25 ºC, 210 nm; t_1 = 14.4 min, t_2 = 19.0 min]: $[\alpha]_D^{25}$ = -32.9° (*c* 1.0, MeOH, 92% e.e.); **^{1}H NMR** (300 MHz, CD_3OD) δ 7.63 – 7.52 (m, 4H), 7.48 – 7.36 (m, 4H), 7.36 – 7.27 (m, 1H), 5.33 (d, *J* = 4.7 Hz, 0.21H, minor), 5.07 (d, *J* = 7.4 Hz, 0.86H, major), 4.68 – 4.43 (m, 1H), 1.48 – 1.32 (m, 18H). **^{13}C NMR** (75 MHz, CD_3OD) δ 173.4, 157.6, 157.4, 142.0, 141.9, 139.2, 129.8, 129.0, 128.3, 127.92, 127.88, 80.8, 80.6, 58.9, 57.4, 28.7, 28.6. **HRMS** (+ESI) Exact mass calculated for $[C_{25}H_{32}N_2O_6Na]^+$ $[(M + Na)^+]$: 479.2153, found: 479.2141.

(2*R*,3*R*)-2,3-bis((*tert*-butoxycarbonyl)amino)-3-(4-methoxyphenyl)propanoic acid (8)

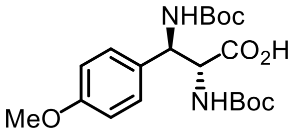

Catalyzed by 15 mol% (*S*)-**Fe1**, the diamination of **8a** (18.0 mg, 0.1 mmol) gave **8** as a white foam (chromatography on silica gel, eluent: EtOAc/*n*-hexane = 1/4 with 0.2% HOAc, 22.1 mg, 54% yield) with 97% e.e. Diastereoselectivity was determined by ^{1}H NMR analysis of the isolated mixture as 9:1. [DAICEL CHIRALPAK IA column, Agilent HPLC 1260, *i*PrOH/*n*-hexane = 10/90 (v/v) with 0.1% TFA, 1.0 mL/min, 20 ºC, 210 nm; t_1 = 15.9 min, t_2 = 20.4 min]: $[\alpha]_D^{25}$ = +26.4° (*c* 1.0, MeOH, 97% e.e.); **^{1}H NMR** (300 MHz, CD_3OD) δ 7.24 (d, *J* = 8.0 Hz, 2H), 6.86 (d, *J* = 8.4 Hz, 2H), 5.19 (d, *J* = 4.9 Hz, 0.14H, minor), 4.96 (d, *J* = 5.9 Hz, 0.94H, major), 4.57 – 4.34 (m, 1H), 3.77 (s, 2.88H major), 3.64 (s, 0.33H, minor), 1.45 – 1.28 (m, 18H). **^{13}C NMR** (75 MHz, CD_3OD) δ 173.6, 160.7, 157.6, 157.3, 132.0, 129.6, 129.0, 114.7, 80.8, 80.5, 59.0, 57.0, 55.7, 28.7, 28.6. **HRMS** (+ESI) Exact mass calculated for $[C_{20}H_{30}N_2O_7Na]^+$ $[(M + Na)^+]$: 433.1945, found: 433.1938.

(*2R,3R*)-2,3-bis((*tert*-butoxycarbonyl)amino)-3-(3-methoxyphenyl)propanoic acid (9)

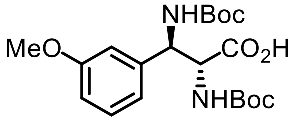

Catalyzed by 15 mol% (*S*)-**Fe1**, the diamination of **9a** (18.0 mg, 0.1 mmol) gave **9** as a white foam (94% conversion based on crude NMR analysis) (chromatography on silica gel, eluent: EtOAc/*n*-hexane = 1/5 with 0.2% HOAc, 16.4 mg, 40% yield) with 92% e.e. Diastereoselectivity was determined by ^{1}H NMR analysis of the isolated mixture as 8:1. [DAICEL CHIRALPAK IA column, Agilent HPLC 1260, ^{i}PrOH/*n*-hexane = 10.0/90.0 (v/v) with 0.1% TFA, 0.5 mL/min, 25 ºC, 210 nm; t_1 = 18.9 min, t_2 = 22.6 min]: $[\alpha]_D^{25}$ = +46.5° (c 0.5, MeOH, 92% e.e.). **^{1}H NMR** (500 MHz, CD_3OD) δ 7.24 (td, *J* = 8.0, 5.0 Hz, 1H), 6.95 – 6.87 (m, 2H), 6.86 – 6.81 (m, 1H), 5.26 (d, *J* = 4.7 Hz, 0.13H, minor), 5.00 (d, *J* = 7.4 Hz, 0.98H, major), 4.60 – 4.49 (m, 1H), 3.81 (s, 3H), 1.46 – 1.36 (m, 18H). **^{13}C NMR** (126 MHz, CD_3OD) δ 173.5, 161.2 (major), 161.1 (minor), 157.6, 157.5, 141.7, 130.4, 120.7, 114.4, 114.0, 80.8, 80.6, 58.9, 57.5, 55.7, 28.8, 28.6. **HRMS** (+ESI) Exact mass calculated for [$C_{20}H_{30}N_2O_7Na^+$ [(M + Na)$^+$]: 433.1945, found: 433.1938.

(2*R*,3*R*)-2,3-bis((*tert*-butoxycarbonyl)amino)-3-(4-fluorophenyl)propanoic acid (10)

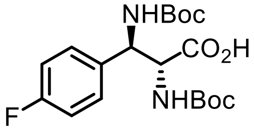

Catalyzed by 15 mol% (*S*)-**Fe1**, the diamination of **10a** (16.8 mg, 0.1 mmol) gave **10** as a white foam (chromatography on silica gel, eluent: EtOAc/*n*-hexane = 1/6 with 0.2% HOAc, 29.1 mg, 73% yield) with 97% e.e. Diastereoselectivity was determined by ^{1}H NMR analysis of the isolated mixture as >20:1. [DAICEL CHIRALPAK IA column, Agilent HPLC 1260, *i*PrOH/*n*-hexane = 10/90 (v/v) with 0.1% TFA, 1.0 mL/min, 25 ºC, 210 nm; t_1 = 15.0 min, t_2 = 17.0 min]: $[\alpha]_D^{25}$ = +13.3° (*c* 1.0, MeOH, 97% e.e.); **^{1}H NMR** (300 MHz, CD_3OD) *δ* 7.45 – 7.25 (m, 2H), 7.03 (t, *J* = 8.6 Hz, 2H), 4.98 (d, *J* = 7.3 Hz, 1H), 4.51 (d, *J* = 7.4 Hz, 1H), 1.47 – 1.24 (m, 18H). **^{13}C NMR** (75 MHz, CD_3OD) *δ* 173.3, 163.7 (d, *J* = 244.6 Hz), 157.5, 157.3, 136.4, 130.5 (d, *J* = 8.0 Hz), 115.9 (d, *J* = 21.7 Hz), 80.8, 80.6, 58.8, 56.9, 28.7, 28.6. **^{19}F NMR** (282 MHz, CD_3OD) *δ* -117.26. **HRMS** (+ESI) Exact mass calculated for [$C_{19}H_{27}FN_2O_6Na$]$^+$ [(M + Na)$^+$]: 421.1745, found: 421.1732.

(2*R*,3*R*)-2,3-bis((*tert*-butoxycarbonyl)amino)-3-(4-chlorophenyl)propanoic acid (11)

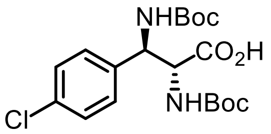

Catalyzed by 15 mol% (*S*)-**Fe1**, the diamination of **11a** (18.5 mg, 0.1 mmol) gave **11** as a white foam (chromatography on silica gel, eluent: EtOAc/*n*-hexane = 1/6 with 0.2% HOAc, 29.4 mg, 71% yield) with 96% e.e. Diastereoselectivity was determined by ^{1}H NMR analysis of the isolated mixture as >20:1. [DAICEL CHIRALPAK IA column, Agilent HPLC 1260, *i*PrOH/*n*-hexane = 10/90 (v/v) with 0.1% TFA, 1.0 mL/min, 25 ºC, 210 nm; t_1 = 10.4 min, t_2 = 12.7 min]: $[\alpha]_D^{25}$ = -34.3° (*c* 1.0, MeOH, 96% e.e.); **^{1}H NMR** (300 MHz, CD_3OD) δ 7.31 (s, 4H), 4.97 (d, *J* = 7.7 Hz, 1H), 4.51 (d, *J* = 7.8 Hz, 1H), 1.51 – 1.21 (m, 18H). **^{13}C NMR** (75 MHz, CD_3OD) δ 173.2, 157.5, 157.3, 139.2, 134.5, 130.2, 129.3, 80.8, 80.7, 58.6, 57.1, 28.7, 28.6. **HRMS** (+ESI) Exact mass calculated for $[C_{19}H_{27}ClN_2O_6Na]^+$ [(M + Na)$^+$]: 437.1441, found: 437.1450.

(2*R*,3*R*)-3-(4-bromophenyl)-2,3-bis((*tert*-butoxycarbonyl)amino)propanoic acid (12)

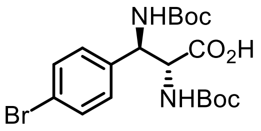

Catalyzed by 15 mol% (*S*)-**Fe1**, the diamination of **12a** (22.9 mg, 0.1 mmol) gave **12** as a white foam (chromatography on silica gel, eluent: EtOAc/*n*-hexane = 1/6 with 0.2% HOAc, 32.3 mg, 70% yield) with 96% e.e. Diastereoselectivity was determined by ^{1}H NMR analysis of the isolated mixture as 10:1. [DAICEL CHIRALPAK IA column, Agilent HPLC 1260, *i*PrOH/*n*-hexane = 10/90 (v/v) with 0.1% TFA, 1.0 mL/min, 25 ºC, 210 nm; t_1 = 11.0 min, t_2 = 13.9 min]: $[\alpha]_D^{25}$ = -13.5° (*c* 0.5, MeOH, 96% e.e.); **^{1}H NMR** (300 MHz, CD_3OD) δ 7.46 (d, *J* = 8.0 Hz, 2H), 7.26 (d, *J* = 8.3 Hz, 2H), 5.25 – 5.22 (m, 0.09H, minor), 5.04 – 4.90 (m, 0.93H, major), 4.96 (d, *J* = 7.7 Hz, 1H), 4.50 (d, *J* = 7.7 Hz, 1H), 1.49 – 1.17 (m, 18H). **^{13}C NMR** (75 MHz, CD_3OD) δ 173.2, 157.5, 157.3, 139.7, 132.3, 130.6, 122.5, 80.8, 80.7, 58.6, 57.1, 28.7, 28.6. **HRMS** (+ESI) Exact mass calculated for $[C_{19}H_{27}BrN_2O_6Na]^+$ [(M + Na)$^+$]: 481.0945, found: 481.0945.

(2*R*,3*R*)-2,3-bis((*tert*-butoxycarbonyl)amino)-3-(4-iodophenyl)propanoic acid (13)

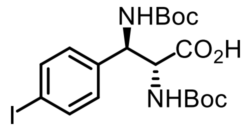

Catalyzed by 15 mol% (*S*)-**Fe1**, the diamination of **13a** (27.6 mg, 0.1 mmol) gave **13** as a white foam (chromatography on silica gel, eluent: EtOAc/*n*-hexane = 1/6 with 0.2% HOAc, 33.9 mg, 67% yield) with 93% e.e. Diastereoselectivity was determined by ^{1}H NMR analysis of the isolated mixture as 7:1. [DAICEL CHIRALPAK IA column, Agilent HPLC 1260, *i*PrOH/*n*-hexane = 10/90 (v/v) with 0.1% TFA, 1.0 mL/min, 25 ºC, 210 nm; t_1 = 12.5 min, t_2 = 18.3 min]: $[\alpha]_D^{25}$ = -6.5° (*c* 1.0, MeOH, 93% e.e.); **^{1}H NMR** (300 MHz, CD_3OD) δ 7.65 (d, *J* = 7.9 Hz, 2H), 7.12 (d, *J* = 8.1 Hz, 2H), 5.25 – 5.19 (m, 0.14H, minor), 5.02 – 4.90 (m, 0.96H, major), 4.59 – 4.44 (m, 1H), 1.43 – 1.32 (m, 18H). **^{13}C NMR** (75 MHz, CD_3OD) δ 173.2, 157.5, 157.3, 140.2, 138.4, 130.7 (major), 130.0 (minor), 93.7, 80.9, 80.7, 58.6, 57.3, 28.7, 28.6. **HRMS** (+ESI) Exact mass calculated for $[C_{19}H_{27}IN_2O_6Na]^+$ $[(M + Na)^+]$: 529.0806, found: 529.0789.

(2*R*,3*R*)-2,3-bis((*tert*-butoxycarbonyl)amino)-3-(4-(methoxycarbonyl)phenyl)propanoic acid (14)

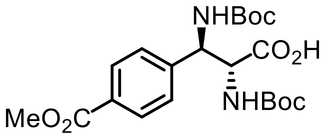

Catalyzed by 15 mol% (*S*)-**Fe1**, the diamination of **14a** (20.8 mg, 0.1 mmol) gave **14** as a light-yellow foam (chromatography on silica gel, eluent: EtOAc/*n*-hexane = 1/3 with 0.2% HOAc, 31.5 mg, 72% yield) with 95% e.e. Diastereoselectivity was determined by ^{1}H NMR analysis of the isolated mixture as >20:1. [DAICEL CHIRALPAK IG column, Agilent HPLC 1260, *i*PrOH/*n*-hexane = 10/90 (v/v) with 0.1% TFA, 1.0 mL/min, 25 ºC, 210 nm; t_1 = 35.4 min, t_2 = 58.9 min]: $[\alpha]_D^{25}$ = +38.2° (*c* 1.0, MeOH, 95% e.e.); **^{1}H NMR** (300 MHz, CD_3OD) δ 7.96 (d, *J* = 8.0 Hz, 2H), 7.46 (d, *J* = 8.0 Hz, 2H), 5.06 (d, *J* = 7.7 Hz, 1H), 4.62 – 4.41 (m, 1H), 3.89 (s, 3H), 1.47 – 1.32 (m, 18H). **^{13}C NMR** (75 MHz, CD_3OD) δ 173.1, 168.3, 157.4, 145.9, 130.6, 130.4, 128.8, 80.84, 80.75, 58.6, 57.5, 52.6, 28.7, 28.5. **HRMS** (+ESI) Exact mass calculated for $[C_{21}H_{30}N_2O_8Na]^+$ $[(M + Na)^+]$: 461.1894, found: 461.1886.

(2*R*,3*R*)-3-(4-acetylphenyl)-2,3-bis((*tert*-butoxycarbonyl)amino)propanoic acid (15)

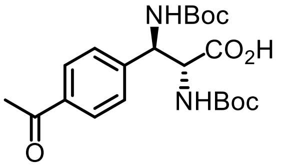

Catalyzed by 15 mol% (*S*)-**Fe1**, the diamination of **15a** (19.2 mg, 0.1 mmol) gave **15** as a white foam (chromatography on silica gel, eluent: EtOAc/*n*-hexane = 1/5 with 0.2% HOAc, 22.1 mg, 52% yield) with 94% e.e. Diastereoselectivity was determined by ^{1}H NMR analysis of the isolated mixture as 6:1. [DAICEL CHIRALPAK IA column, Agilent HPLC 1260, *i*PrOH/*n*-hexane = 10/90 (v/v) with 0.1% TFA, 1.0 mL/min, 25 ºC, 210 nm; t_1 = 24.1 min, t_2 = 26.0 min]: $[\alpha]_D^{25}$ = +64.6° (*c* 1.0, MeOH, 94% e.e.); **^{1}H NMR** (300 MHz, CD_3OD) δ 7.95 (d, *J* = 7.9 Hz, 2H), 7.48 (d, *J* = 8.1 Hz, 2H), 5.35 (d, *J* = 4.5 Hz, 0.15H, minor), 5.07 (d, *J* = 7.6 Hz, 0.83H, major), 4.68 – 4.39 (m, 1H), 2.58 (s, 3H), 1.45 – 1.28 (m, 18H). **^{13}C NMR** (75 MHz, CD_3OD) δ 200.1, 173.0, 157.5, 157.3, 146.0, 137.7, 131.6 (minor), 129.4 (major), 128.9 (major), 128.2 (minor), 80.9, 80.7, 58.6, 57.4, 28.7, 28.5, 26.7. **HRMS** (+ESI) Exact mass calculated for $[C_{21}H_{30}N_2O_7Na]^+$ $[(M + Na)^+]$: 445.1945, found: 445.1929.

(2*R*,3*R*)-2,3-bis((*tert*-butoxycarbonyl)amino)-3-(4-cyanophenyl)propanoic acid (16)

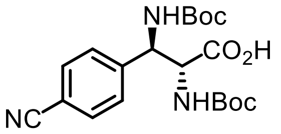

Catalyzed by 15 mol% (*S*)-**Fe1**, the diamination of **16a** (17.5 mg, 0.1 mmol) gave **16** as a white foam (chromatography on silica gel, eluent: EtOAc/*n*-hexane = 1/4 with 0.2% HOAc, 30.1 mg, 74% yield) with 90% e.e. Diastereoselectivity was determined by ^{1}H NMR analysis of the isolated mixture as 4:1. [DAICEL CHIRALPAK IA column, Agilent HPLC 1260, *i*PrOH/*n*-hexane = 10/90 (v/v) with 0.1% TFA, 1.0 mL/min, 25 ºC, 210 nm; t_1 = 29.0 min, t_2 = 191.3 min]: $[\alpha]_D^{25}$ = -26.3° (*c* 1.0, MeOH, 90% e.e.); **^{1}H NMR** (300 MHz, CD_3OD) δ 7.78 – 7.58 (m, 2H), 7.58 – 7.45 (m, 2H), 5.35 (d, *J* = 4.4 Hz, 0.19H, minor), 5.05 (d, *J* = 7.9 Hz, 0.72H, major), 4.70 – 4.31 (m, 1H), 1.46 – 1.29 (m, 18H). **^{13}C NMR** (75 MHz, CD_3OD) δ 172.9, 157.5, 157.3, 146.3, 133.1 (major), 132.2 (minor), 129.7 (major), 129.0 (minor), 119.6, 112.4 (major), 112.2 (minor), 80.9, 80.8, 58.5 (major), 58.3 (minor), 57.5 (major), 57.3 (minor), 28.7, 28.5. **HRMS** (+ESI) Exact mass calculated for $[C_{20}H_{27}N_3O_6Na]^+$ $[(M + Na)^+]$: 428.1792, found: 428.1783.

(*2R,3R*)-2,3-Bis((*tert*-butoxycarbonyl)amino)-3-(4-nitrophenyl)propanoic acid (17)

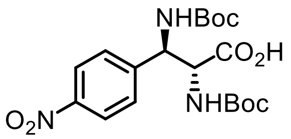

Catalyzed by 15 mol% **(*S*)-Fe1**, the diamination of **17a** (19.5 mg, 0.1 mmol) gave **17** as a white foam (chromatography on silica gel, eluent: EtOAc/*n*-hexane = 1/5 with 0.2% HOAc, 29.2 mg, 67% yield) with 95% e.e Diastereoselectivity was determined by ^{1}H NMR analysis of the isolated mixture as >20:1. [DAICEL CHIRALPAK IA column, Agilent HPLC 1260, ^{i}PrOH/*n*-hexane = 10.0/90.0 (v/v) with 0.1% TFA, 1.0 mL/min, 25 ºC, 210 nm; t_1 = 25.8 min, t_2 = 42.4 min]: $[\alpha]_D^{25}$ = −40.9° (c 0.5, MeOH, 95% e.e.). **^{1}H NMR** (500 MHz, CD_3OD) δ 8.19 (d, *J* = 8.3 Hz, 2H), 7.59 (d, *J* = 8.4 Hz, 2H), 5.10 (d, *J* = 7.9 Hz, 1H), 4.54 (d, *J* = 8.0 Hz, 1H), 1.44 – 1.30 (m, 18H). **^{13}C NMR** (126 MHz, CD_3OD) δ 172.9, 157.5, 157.3, 148.8, 148.2, 129.9, 124.2, 80.9, 80.8, 58.6, 57.4, 28.7, 28.5. **HRMS** (+ESI) Exact mass calculated for $[C_{19}H_{27}N_3O_8Na]^+$ $[(M + Na)^+]$: 448.1690, found: 448.1677.

(*2R,3R*)-2,3-Bis((*tert*-butoxycarbonyl)amino)-3-(4-(trifluoromethyl)phenyl)propanoic acid (18)

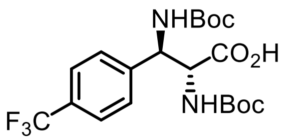

Catalyzed by 15 mol% **(*S*)-Fe1**, the diamination of **18a** (21.8 mg, 0.1 mmol) gave **18** as a white foam (82% conversion based on crude NMR analysis) (chromatography on silica gel, eluent: EtOAc/*n*-hexane = 1/5 with 0.2% HOAc, 21.1 mg, 47% yield) with 94% e.e. Diastereoselectivity was determined by ^{1}H NMR analysis of the isolated mixture as 8:1. [DAICEL CHIRALPAK IA column, Agilent HPLC 1260, ^{i}PrOH/*n*-hexane = 10.0/90.0 (v/v) with 0.1% TFA, 1.0 mL/min, 25 ºC, 210 nm; t_1 = 8.9 min, t_2 = 15.2 min]: $[\alpha]_D^{25}$ = +45.3° (c 1.0, MeOH, 94% e.e.). **^{1}H NMR** (500 MHz, CD_3OD) δ 7.61 (d, *J* = 8.1 Hz, 2H), 7.53 (d, *J* = 8.1 Hz, 2H), 5.37 (d, *J* = 4.5 Hz, 0.12H, minor), 5.05 (d, *J* = 8.1 Hz, 0.9H, major), 4.66 – 4.47 (m, 1H), 1.42 – 1.30 (m, 18H). **^{13}C NMR** (126 MHz, CD_3OD) δ 173.1, 157.44, 157.38, 145.0, 130.8 (q, *J* = 32.2 Hz), 129.4, 128.5, 126.1 (d, *J* = 3.9 Hz), 125.7 (q, *J* = 271.0 Hz), 80.8, 80.7, 58.6, 57.4, 28.7, 28.5. **^{19}F NMR** (282 MHz, CD_3OD) δ -64.04. **HRMS** (+ESI) Exact mass calculated for $[C_{20}H_{27}F_3N_2O_6Na]^+$ $[(M + Na)^+]$: 471.1713, found: 471.1706.

(2*R*,3*R*)-3-(4-azidophenyl)-2,3-bis((*tert*-butoxycarbonyl)amino)propanoic acid (19)

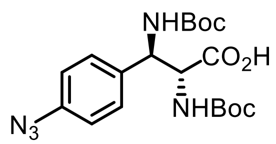

Catalyzed by 15 mol% (*S*)-**Fe1**, the diamination of **19a** (19.1 mg, 0.1 mmol) gave **19** as a white foam (chromatography on silica gel, eluent: EtOAc/*n*-hexane = 1/4 with 0.2% HOAc, 19.6 mg, 47% yield) with 96% e.e. Diastereoselectivity was determined by ^{1}H NMR analysis of the isolated mixture as 7:1. [DAICEL CHIRALPAK IA column, Agilent HPLC 1260, *i*PrOH/*n*-hexane = 10/90 (v/v) with 0.1% TFA, 1.0 mL/min, 40 ºC, 210 nm; t_1 = 12.4 min, t_2 = 14.9 min]: $[\alpha]_D^{25}$ = -22.5° (*c* 1.0, MeOH, 96% e.e.); **^{1}H NMR** (300 MHz, CD_3OD) δ 7.36 (d, *J* = 8.1 Hz, 2H), 7.01 (d, *J* = 8.0 Hz, 2H), 5.24 (d, *J* = 4.8 Hz, 0.13H, minor), 4.99 (d, *J* = 7.5 Hz, 0.93H, major), 4.61 – 4.35 (m, 1H), 1.45 – 1.30 (m, 18H). **^{13}C NMR** (75 MHz, CD_3OD) δ 173.4, 157.6, 157.3, 140.8, 137.3, 130.2 (major), 129.5 (minor), 122.3 (minor), 119.8 (major), 80.8, 80.6, 58.8, 57.1, 28.7, 28.6. **HRMS** (+ESI) Exact mass calculated for $[C_{19}H_{27}N_5O_6Na]^+$ $[(M + Na)^+]$: 444.1854, found: 444.1868.

(*2R,3R*)-2,3-Bis((*tert*-butoxycarbonyl)amino)-3-(4-((4-methoxyphenyl)ethynyl)phenyl)propanoic acid (20)

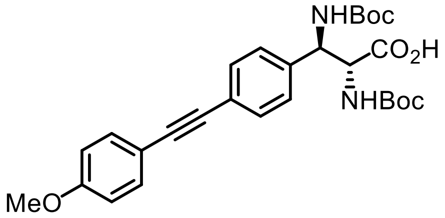

Catalyzed by 15 mol% (*S*)-**Fe1**, the diamination of **20a** (29.4 mg, 0.1 mmol) gave **20** as a white foam (chromatography on silica gel, eluent: EtOAc/*n*-hexane = 1/5 with 0.2% HOAc, 27.8 mg, 52% yield, 10:1 d.r.) with 96% e.e. Diastereoselectivity was determined by ^{1}H NMR analysis of the isolated mixture as 10:1. [DAICEL CHIRALPAK IG column, Agilent HPLC 1260, ^{i}PrOH/*n*-hexane = 10.0/90.0 (v/v) with 0.1% TFA, 0.6 mL/min, 25 ºC, 210 nm; t_1 = 60.2 min, t_2 = 68.2 min]: $[\alpha]_D^{25}$ = +73.0° (c 1.0, MeOH, 96% e.e.). **^{1}H NMR** (500 MHz, CD_3OD) δ 7.45 – 7.42 (m, 4H), 7.33 (d, *J* = 8.0 Hz, 2H), 6.94 – 6.91 (m, 2H), 5.28 (d, *J* = 4.6 Hz, 0.1H, minor), 5.02 (d, *J* = 7.6 Hz, 1H, major), 4.61 – 4.47 (m, 1H), 3.81 (s, 3H), 1.44 – 1.35 (m, 18H). **^{13}C NMR** (126 MHz, CD_3OD) δ 173.5, 161.5, 157.7, 157.5, 140.4, 134.2, 132.3, 128.9, 128.1, 124.4, 116.7, 115.3, 90.6, 88.7, 81.0, 80.8, 58.9, 57.6, 55.9, 28.9, 28.7. **HRMS** (+ESI) Exact mass calculated for $[C_{28}H_{34}N_2O_7Na]^+$ $[(M + Na)^+]$: 533.2258, found: 533.2244.

(2*R*,3*R*)-2,3-bis((*tert*-butoxycarbonyl)amino)-3-(naphthalen-1-yl)propanoic acid (21)

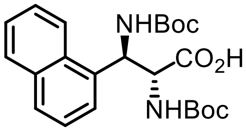

Catalyzed by 15 mol% (*S*)-**Fe1**, the diamination of **21a** (20.0 mg, 0.1 mmol) gave **21** as a white powder (chromatography on silica gel, eluent: EtOAc/*n*-hexane = 1/6 with 0.2% HOAc, 28.2 mg, 66% yield) with 92% e.e. Diastereoselectivity was determined by ^{1}H NMR analysis of the isolated mixture as >20:1. [DAICEL CHIRALPAK IG column, Agilent HPLC 1260, *i*PrOH/*n*-hexane = 3/97 (v/v) with 0.1% TFA, 1.0 mL/min, 25 ºC, 210 nm; t_1 = 114.2 min, t_2 = 136.7 min]: $[\alpha]_D^{25}$ = -8.6° (*c* 1.0, MeOH, 92% e.e.); **^{1}H NMR** (300 MHz, CD_3OD) δ 8.39 (d, *J* = 8.6 Hz, 1H), 7.84 (dd, *J* = 23.6, 8.1 Hz, 2H), 7.67 – 7.38 (m, 4H), 5.91 (d, *J* = 6.7 Hz, 1H), 4.80 – 4.60 (m, 1H), 1.47 – 1.27 (m, 18H). **^{13}C NMR** (75 MHz, CD_3OD) δ 173.4, 157.5, 157.4, 136.1, 135.3, 132.7, 129.8, 129.3, 127.4, 126.7, 126.2, 125.3, 124.3, 80.73, 80.67, 58.2, 52.9, 28.7, 28.6. **HRMS** (+ESI) Exact mass calculated for $[C_{23}H_{30}N_2O_6Na]^+$ $[(M + Na)^+]$: 453.1996, found: 453.1983.

(2*R*,3*R*)-2,3-bis((*tert*-butoxycarbonyl)amino)-3-(naphthalen-2-yl)propanoic acid (22)

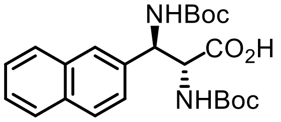

Catalyzed by 15 mol% (*S*)-**Fe1**, the diamination of **22a** (20.0 mg, 0.1 mmol) gave **22** as a white powder (chromatography on silica gel, eluent: EtOAc/*n*-hexane = 1/6 with 0.2% HOAc, 26.2 mg, 61% yield) with 93% e.e. Diastereoselectivity was determined by ^{1}H NMR analysis of the isolated mixture as 8:1. [DAICEL CHIRALPAK IG column, Agilent HPLC 1260, *i*PrOH/*n*-hexane = 10/90 (v/v) with 0.1% TFA, 0.8 mL/min, 25 ºC, 210 nm; t_1 = 30.0 min, t_2 = 38.4 min]: $[\alpha]_D^{25}$ = -21.7° (*c* 1.0, MeOH, 93% e.e.); **^{1}H NMR** (300 MHz, CD_3OD) δ 7.87 – 7.68 (m, 4H), 7.63 – 7.37 (m, 3H), 5.45 (d, *J* = 4.6 Hz, 0.11H, minor), 5.18 (d, *J* = 7.8 Hz, 0.84H, major), 4.74 – 4.44 (m, 1H), 1.44 – 1.22 (m, 18H). **^{13}C NMR** (75 MHz, CD_3OD) δ 173.5, 157.53, 157.45, 137.6, 134.5 (d, *J* = 13.5 Hz, 1C), 132.3, 129.01, 128.96, 128.6, 127.6, 127.1, 127.0, 126.2, 80.8, 80.6, 58.9, 57.7, 28.7, 28.5. **HRMS** (+ESI) Exact mass calculated for $[C_{23}H_{30}N_2O_6Na]^+$ $[(M + Na)^+]$: 453.1996, found: 453.1991.

(2*R*,3*S*)-2,3-bis((*tert*-butoxycarbonyl)amino)-3-(thiophen-2-yl)propanoic acid (23)

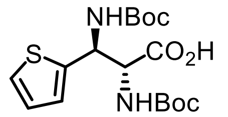

Catalyzed by 15 mol% (*S*)-**Fe1**, the diamination of **23a** (15.6 mg, 0.1 mmol) gave **23** as a white foam (chromatography on silica gel, eluent: EtOAc/*n*-hexane = 1/6 with 0.2% HOAc, 15.3 mg, 40% yield) with 95% e.e. Diastereoselectivity was determined by ^{1}H NMR analysis of the isolated mixture as >20:1. [DAICEL CHIRALPAK IA column, Agilent HPLC 1260, *i*PrOH/*n*-hexane = 10/90 (v/v) with 0.1% TFA, 1.0 mL/min, 25 ºC, 210 nm; t_1 = 9.4 min, t_2 = 10.1 min]: $[\alpha]_D^{25}$ = -13.1° (*c* 0.5, MeOH, 95% e.e.); **^{1}H NMR** (300 MHz, CD_3OD) δ 7.29 (d, *J* = 5.1 Hz, 1H), 7.05 – 6.92 (m, 2H), 5.34 (d, *J* = 6.3 Hz, 1H), 4.65 – 4.45 (m, 1H), 1.51 – 1.35 (m, 18H). **^{13}C NMR** (75 MHz, CD_3OD) δ 173.1, 157.8, 157.3, 143.1, 127.7, 126.4, 125.7, 81.0, 80.7, 59.2, 53.6, 28.7, 28.6. **HRMS** (+ESI) Exact mass calculated for $[C_{17}H_{26}N_2O_6SNa]^+$ $[(M + Na)^+]$: 409.1404, found: 409.1392.

(*2R,3R*)-2,3-bis((*tert*-butoxycarbonyl)amino)-3-(4-(thiophen-2-yl)phenyl)propanoic acid (24)

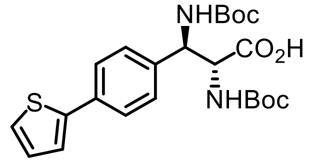

Catalyzed by 15 mol% (*S*)-**Fe1** the diamination of **24a** (23.2 mg, 0.1 mmol) gave **24** as a light-yellow foam (chromatography on silica gel, eluent: EtOAc/*n*-hexane = 1/5 with 0.2% HOAc, 26.4 mg, 57% yield) with 99% e.e. Diastereoselectivity was determined by ^{1}H NMR analysis of the isolated mixture as >20:1. [DAICEL CHIRALPAK IA column, Agilent HPLC 1260, ^{i}PrOH/*n*-hexane = 10.0/90.0 (v/v) with 0.1% TFA, 1.0 mL/min, 25 ºC, 210 nm; t_1 = 19.4 min, t_2 = 25.6 min]: $[\alpha]_D^{25}$ = +100.2° (c 0.5, MeOH, 99% e.e.). **^{1}H NMR** (500 MHz, CD_3OD) δ 7.65 – 7.57 (m, 3H), 7.46 (dd, *J* = 5.0, 2.9 Hz, 1H), 7.44 (dd, *J* = 5.1, 1.4 Hz, 1H), 7.36 (d, *J* = 8.0 Hz, 2H), 5.03 (d, *J* = 7.3 Hz, 1H), 4.55 (d, *J* = 7.4 Hz, 1H), 1.45 – 1.33 (m, 18H). **^{13}C NMR** (126 MHz, CD_3OD) δ 173.5, 157.7, 157.4, 143.2, 138.2, 136.6, 129.0, 127.3, 127.2, 127.1, 121.3, 80.8, 80.6, 58.9, 57.4, 28.7, 28.6. **HRMS** (+ESI) Exact mass calculated for $[C_{23}H_{30}N_2O_6SNa]^+$ $[(M + Na)^+]$: 485.1717, found: 485.1717.

(2*R*,3*R*)-2,3-bis((*tert*-butoxycarbonyl)amino)-2-phenylbutanoic acid (25)

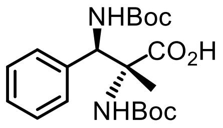

Catalyzed by 15 mol% (*S*)-**Fe1**, the diamination of **25a** (16.4 mg, 0.1 mmol) gave **25** as a white foam (chromatography on silica gel, eluent: EtOAc/*n*-hexane = 1/6 with 0.2% HOAc, 16.8 mg, 43% yield) with 73% e.e. Diastereoselectivity was determined by ^{1}H NMR analysis of the isolated mixture as >20:1. [DAICEL CHIRALPAK OD-H column, Agilent HPLC 1260, *i*PrOH/*n*-hexane = 5/95 (v/v) with 0.1% TFA, 1.0 mL/min, 25 ºC, 210 nm; t_1 = 4.9 min, t_2 = 6.9 min]: $[\alpha]_D^{25}$ = -5.1° (*c* 1.0, MeOH, 73% e.e.); **^{1}H NMR** (300 MHz, CD_3OD) δ 7.37 – 7.20 (m, 5H), 5.18 – 5.04 (m, 1H), 1.55 – 1.33 (m, 21H). **^{13}C NMR** (75 MHz, CD_3OD) δ 175.6, 157.7, 157.6, 139.9, 129.5, 128.9, 128.7, 80.6, 80.5, 64.0, 61.2, 28.7. **HRMS** (+ESI) Exact mass calculated for $[C_{20}H_{30}N_2O_6Na]^+$ $[(M + Na)^+]$: 417.1996, found: 417.1993.

(2*R*,3*R*)-2,3-bis((*tert*-butoxycarbonyl)amino)butanoic acid (26)

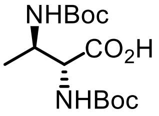

Catalyzed by 15 mol% (*S*)-**Fe1**, the diamination of **26a** (8.8 mg, 0.1 mmol) gave **26** as a colorless oil (chromatography on silica gel, eluent: EtOAc/*n*-hexane = 1/8 with 0.2% HOAc, 13.5 mg, 42% yield) with 96% e.e. Diastereoselectivity was determined by ^{1}H NMR analysis of the isolated mixture as 1.6:1. [DAICEL CHIRALPAK AD-H column, Agilent HPLC 1260, *i*PrOH/*n*-hexane = 5/95 (v/v) with 0.1% TFA, 0.7 mL/min, 25 ºC, 210 nm; t_1 = 19.8 min, t_2 = 37.7 min]: $[\alpha]_D^{25}$ = +17.0° (*c* 1, MeOH, 96% e.e.); **^{1}H NMR** (500 MHz, CD_3OD) δ 4.41 (d, *J* = 4.5 Hz, 0.48H, minor), 4.15 (d, *J* = 2.0 Hz, 0.79H, major), 4.10 – 3.95 (m, 1H), 1.46 – 1.42 (m, 18H), 1.16 (d, *J* = 6.4 Hz, 1.29H, minor), 1.09 (d, *J* = 6.9 Hz, 2.07H, major). **^{13}C NMR** (126 MHz, CD_3OD) δ 174.2 (minor), 174.0 (major), 158.1, 157.7 (major), 157.6 (minor), 80.7 (major), 80.6 (minor), 80.3 (major), 80.2 (minor), 59.5, 58.4, 28.8, 28.70, 28.66, 18.4 (minor), 16.1 (major). **HRMS** (+ESI) Exact mass calculated for $[C_{14}H_{26}N_2O_6Na]^+$ $[(M + Na)^+]$: 341.1683, found: 314.1676.

(*2R,3R*)-2,3-bis((*tert*-butoxycarbonyl)amino)hexanoic acid (27)

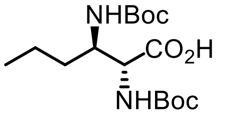

Catalyzed by 15 mol% **(*S*)-Fe1**, the diamination of **27a** (11.6 mg, 0.1 mmol) gave **27** as a colorless oil (chromatography on silica gel, eluent: EtOAc/*n*-hexane = 1/8 with 0.2% HOAc, 15.9 mg, 46% yield) with 90% e.e. Diastereoselectivity was determined by ^{1}H NMR analysis of the isolated mixture as 1.2:1. [DAICEL CHIRALPAK AD-H column, Agilent HPLC 1260, ^{i}PrOH/*n*-hexane = 5.0/95.0 (v/v) with 0.1% TFA, 1.0 mL/min, 25 ºC, 210 nm; t_1 = 12.8 min, t_2 = 23.3 min]: $[\alpha]_D^{25}$ = −14.16° (c 0.5, MeOH, 90% e.e.). The ee value of minor diastereomer could also be obtained (77% e.e.) using larger amount of product (60.0 mg) which isolated from large scale reaction (0.5 mml). [DAICEL CHIRALPAK AD-H column, Agilent HPLC 1260, ^{i}PrOH/*n*-hexane = 5.0/95.0 (v/v) with 0.1% TFA, 1.0 mL/min, 25 ºC, 210 nm; t_1 = 9.0 min, t_2 = 14.9 min] **^{1}H NMR** (600 MHz, CD_3OD) δ 4.40 – 4.30 (m, 0.40H, minor), 4.26 – 4.17 (m, 0.57H, major), 4.13 – 4.02 (m, 0.66H, major), 3.95 – 3.86 (m, 0.46H, minor), 1.49 – 1.29 (m, 22H), 0.97 – 0.89 (m, 3H). **^{13}C NMR** (151 MHz, CD_3OD) δ 174.5, 158.2 (minor), 158.1 (major), 158.0, 81.5 (minor), 80.7 (major), 80.6 (minor), 80.2 (major), 58.5 (minor), 58.4 (major), 53.1 (major), 52.9 (minor), 35.5, 33.4, 28.8 (minor), 28.70, 28.67 (major), 26.6 20.4 (major), 20.3 (minor), 14.1 (major), 14.0 (minor). **HRMS** (+ESI) Exact mass calculated for $[C_{16}H_{30}N_2O_6Na]^+$ $[(M + Na)^+]$: 369.1996, found: 369.1987.

(2*R*,3*R*)-2,3-bis((*tert*-butoxycarbonyl)amino)octanoic acid (28)

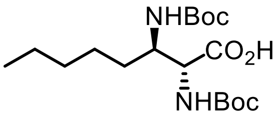

Catalyzed by 15 mol% (*S*)-**Fe1**, the diamination of **28a** (14.4 mg, 0.1 mmol) gave **28** as a white gum (chromatography on silica gel, eluent: EtOAc/*n*-hexane = 1/8 with 0.2% HOAc, 18.3 mg, 49% yield) with 90% e.e. Diastereoselectivity was determined by ^{1}H NMR analysis of the isolated mixture as 2.1:1. [DAICEL CHIRALPAK AD-H column, Agilent HPLC 1260, *i*PrOH/*n*-hexane = 5/95 (v/v) with 0.1% TFA, 1.0 mL/min, 25 ºC, 210 nm; t_1 = 18.2 min, t_2 = 24.1 min]: $[\alpha]_D^{25}$ = -148.6° (*c* 0.25, MeOH, 90% e.e.); **^{1}H NMR** (300 MHz, CD_3OD) *δ* 4.38 – 4.29 (m, 0.26H, minor), 4.26 – 4.14 (m, 0.56H, major), 4.13 – 3.80 (m, 1H), 1.55 – 1.37 (m, 18H), 1.37 – 1.22 (m, 6H), 0.97 – 0.81 (m, 3H). **^{13}C NMR** (126 MHz, CD_3OD) *δ* 174.6, 158.1, 158.0, 80.7, 80.1, 58.6 (minor), 58.4 (major), 53.3 (minor), 53.2 (major),

33.3, 32.61 (major), 32.56 (minor), 28.8, 28.71, 28.67, 26.9 (minor), 26.8 (major), 23.6, 14.4 (minor), 14.3 (major). **HRMS** (+ESI) Exact mass calculated for $[C_{18}H_{34}N_2O_6Na]^+$ $[(M + Na)^+]$: 397.2309, found: 397.2299.

(*2R,3R*)-6-bromo-2,3-bis((*tert*-butoxycarbonyl)amino)hexanoic acid (29)

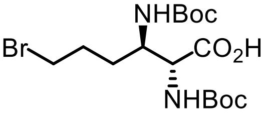

Catalyzed by 15 mol% **(*S*)-Fe1**, the diamination of **29a** (19.5 mg, 0.1 mmol) gave **29** as a light-yellow gum (chromatography on silica gel, eluent: EtOAc/*n*-hexane = 1/6 with 0.2% HOAc, 23.4 mg, 55% yield) with 99% e.e. Diastereoselectivity was determined by ^{1}H NMR analysis of the isolated mixture as 1.5:1. [DAICEL CHIRALPAK AD-H column, Agilent HPLC 1260, ^{i}PrOH/*n*-hexane = 5.0/95.0 (v/v) with 0.1% TFA, 1.0 mL/min, 25 ºC, 210 nm; t_1 = 37.2 min, t_2 = 43.2 min]: $[\alpha]_D^{25}$ = +17.8° (c 1.0, MeOH, 99% e.e.). **^{1}H NMR** (600 MHz, CD_3OD) δ 4.36 (d, *J* = 5.4 Hz, 0.37H, minor), 4.23 (d, *J* = 4.1 Hz, 0.62H, major), 4.14 – 4.03 (m, 0.65H, major), 3.99 – 3.90 (m, 0.40H, minor), 3.50 – 3.42 (m, 2H), 1.96 – 1.82 (m, 2H), 1.72 – 1.53 (m, 2H), 1.47 – 1.41 (m, 18H). **^{13}C NMR** (151 MHz, CD_3OD) δ 174.0 (major), 173.8 (minor), 158.2 (major), 158.1 (minor), 158.0, 80.8 (major), 80.7 (minor), 80.3, 58.3, 52.6 (minor), 52.6 (major), 33.8 (major), 33.7 (minor), 32.1, 30.7 (minor), 30.6 (major), 30.0, 28.76 (major), 28.71 (minor), 28.68, 26.6. **HRMS** (+ESI) Exact mass calculated for $[C_{16}H_{29}BrN_2O_6Na]^+$ $[(M + Na)^+]$: 447.1101, found: 447.1107.

(*2R,3R*)-6-azido-2,3-bis((*tert*-butoxycarbonyl)amino)hexanoic acid (30)

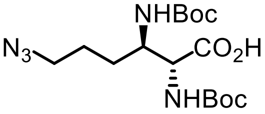

Catalyzed by 15 mol% (*S*)-**Fe1**, the diamination of **30a** (15.7 mg, 0.1 mmol) gave **30** as a white gum (chromatography on silica gel, eluent: EtOAc/*n*-hexane = 1/6 with 0.2% HOAc, 16.3 mg, 42% yield) with 92% e.e. Diastereoselectivity was determined by ^{1}H NMR analysis of the isolated mixture as 1.6:1. [DAICEL CHIRALPAK AD-H column, Agilent HPLC 1260, ^{i}PrOH/*n*-hexane = 15.0/85.0 (v/v) with 0.1% TFA, 1.0 mL/min, 25 ºC, 254 nm; t_1 = 8.0 min, t_2 = 9.1 min]: $[\alpha]_D^{25}$ = +65.3° (c 0.25, MeOH, 92% e.e.). **^{1}H NMR** (600 MHz, CD_3OD) δ 4.35 (d, *J* = 5.4 Hz, 0.38H, minor), 4.23 (d, *J* = 4.1 Hz, 0.64H, major), 4.13 – 4.02 (m, 0.62H, major), 3.93 (dt, *J* = 9.4, 4.3 Hz, 0.40H, minor), 3.36 – 3.31 (m, 2H), 1.70 – 1.51 (m, 4H), 1.46 – 1.41 (m, 18H). **^{13}C NMR** (151 MHz, CD_3OD) δ 172.7 (major), 172.5 (minor), 156.8 (major), 156.7 (minor),

156.6, 79.4 (major), 79.3 (minor), 78.9, 57.0 (minor), 56.9 (major), 51.52 (minor), 51.48 (major), 50.71 (major), 50.66 (minor), 29.4 (minor), 29.2 (major), 27.36 (minor), 27.29, 27.26 (major), 25.3. **HRMS** (+ESI) Exact mass calculated for $[C_{16}H_{29}N_5O_6Na]^+$ [(M + Na)$^+$]: 410.2010, found: 410.2004.

(2*R*,3*R*)-2,3,6-tris((*tert*-butoxycarbonyl)amino)hexanoic acid (31)

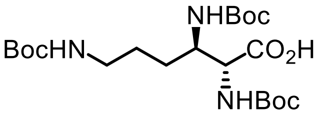

Catalyzed by 15 mol% (*S*)-**Fe1**, the diamination of **31a** (23.1 mg, 0.1 mmol) gave **31** as a light-yellow solid (chromatography on silica gel, eluent: EtOAc/*n*-hexane = 1/6 with 0.2% HOAc, 18.5 mg, 40% yield) with 93% e.e. Diastereoselectivity was determined by ^{1}H NMR analysis of the isolated mixture as 1.3:1. [DAICEL CHIRALPAK IG column, Agilent HPLC 1260, ^{i}PrOH/*n*-hexane = 15.0/85.0 (v/v) with 0.1% TFA, 1.0 mL/min, 25 ºC, 254 nm; t_1 = 11.2 min, t_2 = 13.6 min]: $[\alpha]_D^{25}$ = +122.9° (c 0.25, MeOH, 93% e.e.). **^{1}H NMR** (500 MHz, CD_3OD) δ 4.36 (d, *J* = 5.2 Hz, 0.48H, minor), 4.22 (d, *J* = 4.1 Hz, 0.50H, minor), 4.11 – 4.01 (m, 0.64H, major), 3.96 – 3.84 (m, 0.62H, major). 3.58 – 3.50 (m, 1H), 3.10 – 2.97 (m, 3H), 2.25 (td, *J* = 6.7, 3.9 Hz, 1H), 1.82 – 1.75 (m, 1H), 1.44 – 1.43 (m, 27H). **^{13}C NMR** (126 MHz, CD_3OD) δ 172.8 (major), 172.6 (minor), 157.20 (major), 157.15 (minor), 156.8(major), 156.6 (minor), 79.3 (major), 79.2 (minor), 78.8 (major), 78.50 (minor), 78.47 (minor), 78.43 (major), 57.0 (minor), 56.8 (major), 51.8 (major), 51.7 (minor), 39.7 (minor), 39.6 (major), 27.39 (major), 27.37 (minor), 27.32 (minor), 27.29 (major), 27.26 (minor), 27.24 (major), 26.9, 22.4. **HRMS** (+ESI) Exact mass calculated for $[C_{21}H_{39}N_3O_8Na]^+$ [(M + Na)$^+$]: 484.2629, found: 484.2617.

(2*R*,3*R*)-2,3-bis((*tert*-butoxycarbonyl)amino)-2-phenylbutanoic acid (32)

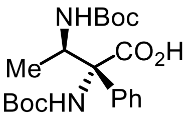

Catalyzed by 15 mol% (*S*)-**Fe1**, the diamination of **32a** (16.4 mg, 0.1 mmol) gave **32** as a white foam (chromatography on silica gel, eluent: EtOAc/*n*-hexane = 1/6 with 0.2% HOAc, 14.5 mg, 37% yield) with 90% e.e. Diastereoselectivity was determined by ^{1}H NMR analysis of the isolated mixture as >20:1. [DAICEL CHIRALPAK IA column, Agilent HPLC 1260, *i*PrOH/*n*-hexane = 10/90 (v/v) with 0.1% TFA, 1.0 mL/min, 25 ºC, 210 nm; t_1 = 9.7 min, t_2 = 10.6 min]: $[\alpha]_D^{25}$

= +16.1° (c 0.5, MeOH, 90% e.e.); ^{1}**H NMR** (300 MHz, CD_3OD) δ 7.56 (d, J = 6.9 Hz, 2H), 7.42 – 7.17 (m, 3H), 4.70 – 4.55 (m, 1H), 1.41 (s, 18H), 1.08 (d, J = 6.8 Hz, 3H). ^{13}**C NMR** (126 MHz, CD_3OD) δ 174.1, 157.9, 157.1, 138.9, 128.8, 128.7, 128.5, 80.8, 80.4, 70.4, 52.6, 28.7, 17.0. **HRMS** (+ESI) Exact mass calculated for $[C_{20}H_{30}N_2O_6Na]^+$ [(M + Na)$^+$]: 417.1996, found: 417.1987.

(*R*)-2-((*tert*-butoxycarbonyl)amino)-2-(1-((*tert*-butoxycarbonyl)amino)cyclohexyl)acetic acid (33)

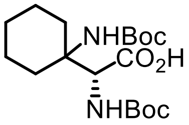

Catalyzed by 15 mol% (*S*)-**Fe1**, the diamination of **33** (14.2 mg, 0.1 mmol) gave **33** as a white gum (chromatography on silica gel, eluent: EtOAc/*n*-hexane = 1/6 with 0.2% HOAc, 15.3 mg, 41% yield) with 98% e.e. [DAICEL CHIRALPAK IA column, Agilent HPLC 1260, *i*PrOH/*n*-hexane = 10/90 (v/v) with 0.1% TFA, 1.0 mL/min, 25 ºC, 210 nm; t_1 = 12.3 min, t_2 = 14.3 min]: $[\alpha]_D^{25}$ = +19.0° (c 1.0, MeOH, 98% e.e.); ^{1}**H NMR** (500 MHz, CD_3OD) δ 4.57 (s, 1H), 1.98 – 1.88 (m, 2H), 1.76 – 1.68 (m, 2H), 1.43 (d, J = 8.8 Hz, 18H), 1.32 – 1.20 (m, 6H). ^{13}**C NMR** (126 MHz, CD_3OD) δ 176.4, 158.3, 157.7, 80.3, 79.7, 55.0, 50.9, 45.2, 35.1, 28.9, 28.8, 26.9, 26.6, 26.4. **HRMS** (+ESI) Exact mass calculated for $[C_{18}H_{32}N_2O_6Na]^+$ [(M + Na)$^+$]: 395.2153, found: 395.2148.

4.1 Diamination reaction by using chiral Fe(III) catalyst

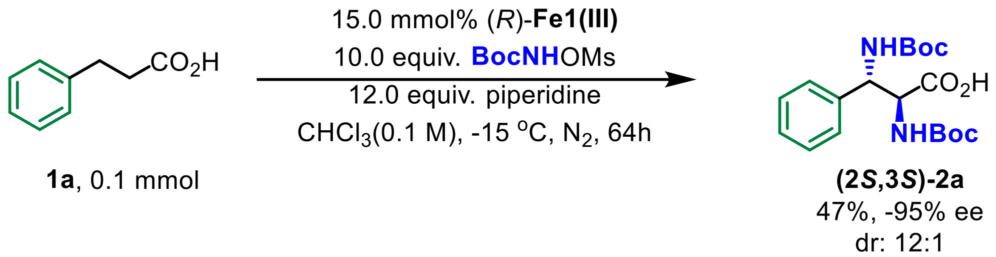

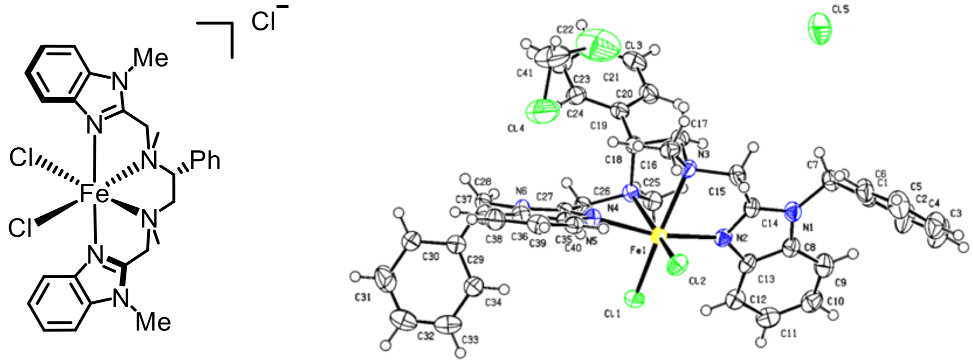

Following the **general procedure** for the iron-catalyzed diaminations of carboxylic acids, the reaction of carboxylic acid **1a** (0.1 mmol, 1.0 equiv.), BocNHOMs (10.0 equiv.), (*R*)-**Fe1**(III) (15 mol%) and piperidine (120 µL, 12.0 equiv.) in $CHCl_3$ (1.0 mL, 0.1 M) was stirred at –15 ºC for 64 hours under N_2. After work-up, the solvent was evaporated under reduced pressure. The residue was purified by column chromatography on silica gel using the previously mentioned solvent as the eluent. The *N*-Boc-protected di-amino acid (2*S*,3*S*)-**2a** was obtained in 47%, and with -95% ee.

4.2 Ligand recovering experiment

The reaction was set up following the **general procedure** but on a 0.3 mmol scale of acid **1a**. After completion, the reaction mixture was diluted with 40 mL Et_2O and was washed with aqueous $NaHSO_4$ (1 M) twice (2×30 mL). The combined aqueous layer was extracted with Et_2O (2×30 mL). The combined organic layer was dried over Na_2SO_4. After filtration,

the solvent was evaporated under reduced pressure and the residue was purified by column chromatography on silica gel to give the product **2a** in 66% yield (75.3 mg, 12:1 dr with 95% ee). The combined aqueous layer was then basified with 1.0 M NaOH solution to pH = 10 ~ 11. It was extracted with Et_2O (3×40 mL) afterwards. The second combined organic layer was dried over Na_2SO_4. After filtration, the solvent was evaporated under reduced pressure and the residue was purified by column chromatography on silica gel with EA/MeOH (20:1 to 3:1) to provide the chiral ligand **S2-Bn** in 77% recovery yield (20.9 mg).

5. Mechanistic Experiments

5.1 Stepwise amination experiments with catalyst (*S*)-Fe1: 1st step

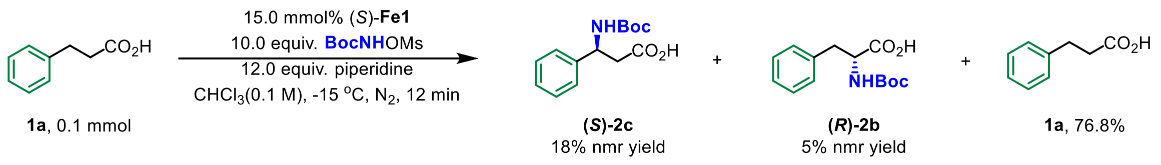

Following the **general procedure** for the iron-catalyzed diaminations of carboxylic acids, the reaction of carboxylic acid **1a** (0.1 mmol, 1.0 equiv.), BocNHOMs (10.0 equiv.), (*S*)-**Fe1** (15 mol%) and piperidine (120 µL, 12.0 equiv.) in $CHCl_3$ (1.0 mL, 0.1 M) was stirred at –15 ºC for 12 min under N_2. After work-up, the solvent was evaporated under reduced pressure. The crude sample was analyzed by ^{1}H NMR, the β-amino acid (*S*)-**2c** was detected in 18% NMR yield, the α-amino acid (*R*)-**2b** was detected in 5% NMR yield, and the starting material **1a** was intact in 76.8 %. The ee value of **2c** was determined by chiral HPLC as 56%. [DAICEL CHIRALPAK IG column, Agilent HPLC 1260, *i*PrOH/*n*-hexane = 10/90 (v/v) with 0.1% TFA, 1.0 mL/min, 25 ºC, 210 nm; t_1 = 15.8 min, t_2 = 36.4 min.]

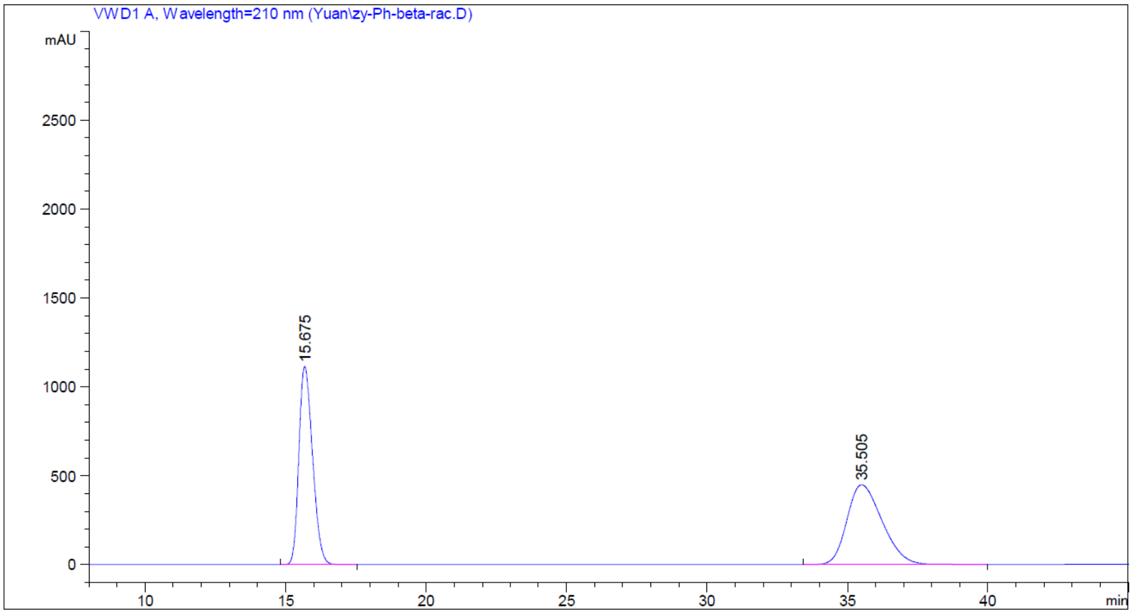

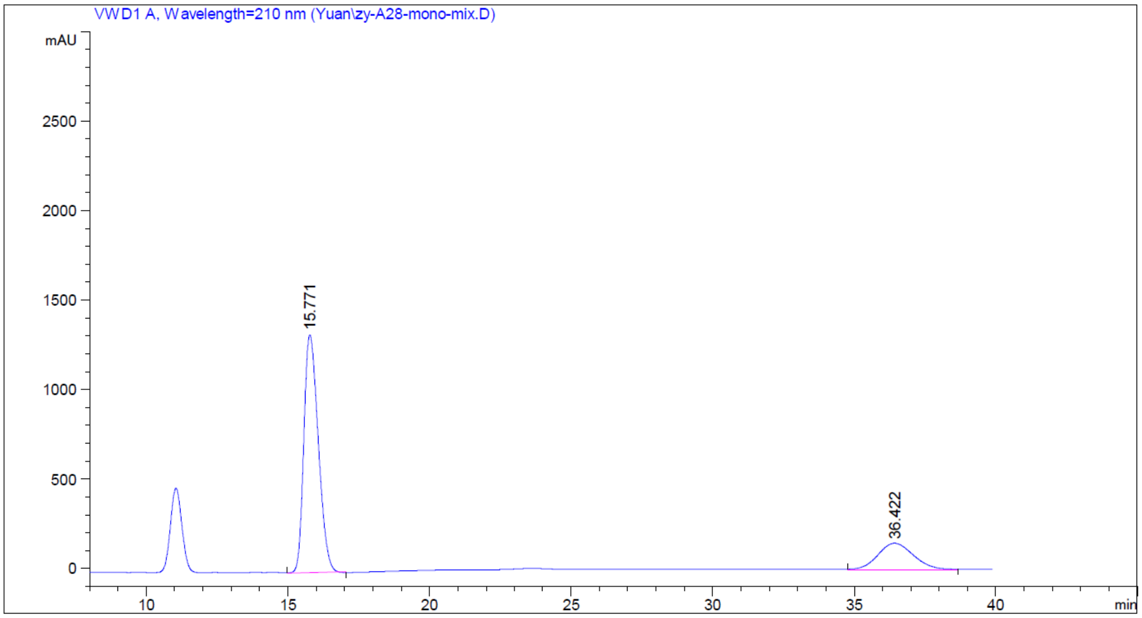

Peak #	RetTime [min]	Type	Width [min]	Area [mAU*s]	Height [mAU]	Area %
1	15.771	MM R	0.5900	4.70719e4	1329.70422	78.1171
2	36.422	MM R	1.4805	1.31862e4	148.44821	21.8829

Figure S8. HPLC traces of rac-**2c** and enantioenriched-**2c**.

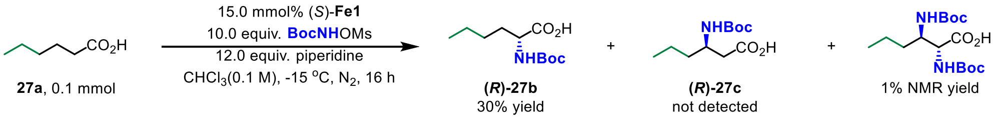

Following the **general procedure** for the iron-catalyzed diaminations of carboxylic acids, the reaction of carboxylic acid **27a** (0.1 mmol, 1.0 equiv.), BocNHOMs (10.0 equiv.), (*S*)-**Fe1** (15 mol%) and piperidine (120 µL, 12.0 equiv.) in $CHCl_3$ (1.0 mL, 0.1 M) was stirred at –15 ºC for 6 hours under N_2. After work-up, the solvent was evaporated under reduced pressure. The residue was purified by column chromatography on silica gel using the previous mentioned solvent as the eluent. The α-amino acid (*R*)-**27b** was isolated in 30%, the β-amino acid (*R*)-**27c** was not detected in NMR, and the diamination product was detected in 1% NMR yield. The conversion was not able to be determined since the starting material hexanoic acid can volatilize during concentration. The ee value of (*R*)-**27b** was

determined by chiral HPLC as 97%. [DAICEL CHIRALPAK IA column, Agilent HPLC 1260, ^{i}PrOH/*n*-hexane = 5.0/95.0 (v/v) with 0.1% TFA, 1.0 mL/min, 25 ºC, 210 nm; t_1 = 13.2 min, t_2 = 23.0 min]

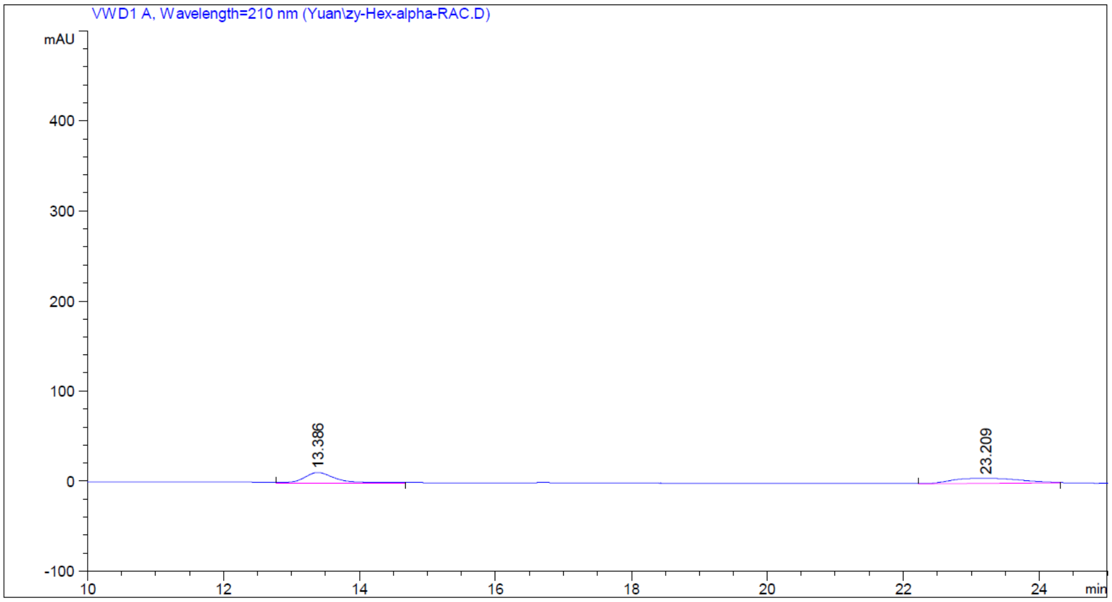

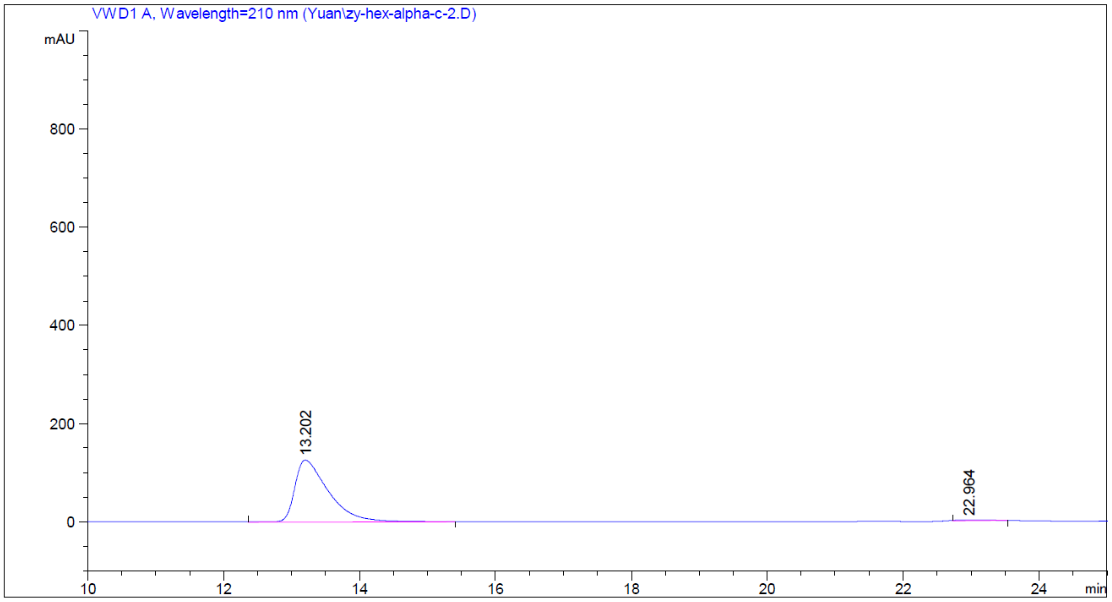

Peak #	RetTime [min]	Type	Width [min]	Area [mAU*s]	Height [mAU]	Area %
1	13.202	MM R	0.5767	4378.80371	126.54984	98.3296
2	22.971	MM	0.6587	74.38799	1.88210	1.6704

Figure S9. HPLC traces of rac-**27b** and enantioenriched-**27b**.

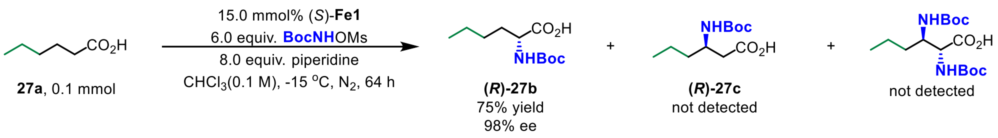

Following the **general procedure** for the iron-catalyzed diaminations of carboxylic acids, the reaction of carboxylic acid **27a** (0.1 mmol, 1.0 equiv.), BocNHOMs (6.0 equiv.), (*S*)-**Fe1** (15 mol%) and piperidine (80 μL, 8.0 equiv.) in $CHCl_3$ (1.0 mL, 0.1 M) was stirred at –15 ºC for 64 hours under N_2. After work-up, the solvent was evaporated under reduced pressure. The residue was purified by column chromatography on silica gel using the previous mentioned solvent as the eluent. The α-amino acid (*R*)-**27b** was isolated in 75% yield as white foam (17.3 mg, 98% ee). No β-aminated or diaminated product was isolated. **^{1}H NMR** (300 MHz, MeOD) δ 4.13 – 3.87 (m, 1H), 1.88 – 1.72 (m, 1H), 1.70 – 1.53 (m, 1H), 1.44 (s, 9H), 1.43 – 1.27 (m, 4H), 0.93 (t, *J* = 4.6 Hz, 3H).

5.2 Stepwise amination experiments with catalyst (*S*)-Fe1: 2nd step

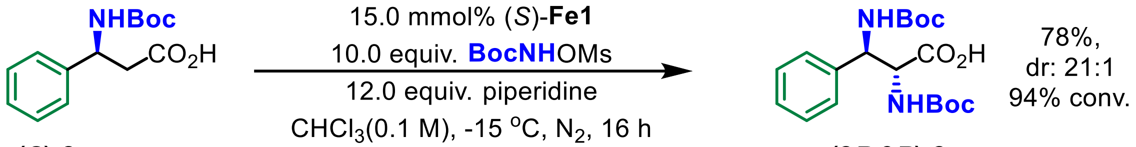

(*S*)-2c, 0.1 mmol **(2*R*,3*R*)-2a**

Following the **general procedure** for the iron-catalyzed diaminations of carboxylic acids, the reaction of β-amino acid (*S*)-**2c** (0.1 mmol, 1.0 equiv.), BocNHOMs (10.0 equiv.), (*S*)-**Fe1** (15 mol%) and piperidine (120 µL, 12.0 equiv.) in $CHCl_3$ (1.0 mL, 0.1 M) was stirred at –15 ºC for 16 hours under N_2. After work-up, the solvent was evaporated under reduced pressure. The residue was purified by column chromatography on silica gel using the previous mentioned solvent as the eluent. The *N*-Boc-protected α,β-di-amino acid (2*R*,3*R*)-**2a** was detected and isolated in 78% yield (94% conv.) with 21:1 dr.

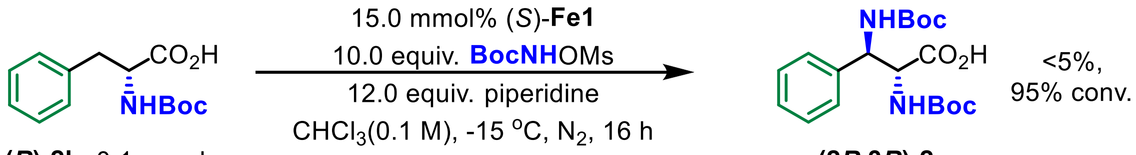

(*R*)-2b, 0.1 mmol **(2*R*,3*R*)-2a**

Following the **general procedure** for the iron-catalyzed diaminations of carboxylic acids, the reaction of β-amino acid (*R*)-**2b** (0.1 mmol, 1.0 equiv.), BocNHOMs (10.0 equiv.), (*S*)-**Fe1** (15 mol%) and piperidine (120 µL, 12.0 equiv.) in $CHCl_3$ (1.0 mL, 0.1 M) was stirred at –15 ºC for 16 hours under N_2. After work-up, the solvent was evaporated under reduced pressure. The *N*-Boc-protected α,β-di-amino acid (2*R*,3*R*)-**2a** was detected in less than 5% yield (95% conv.).

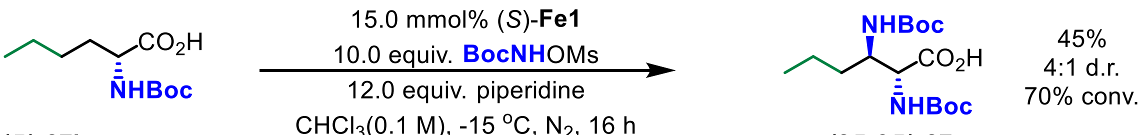

(*R*)-27b, 0.1 mmol **(2*R*,3*R*)-27**

Following the **general procedure** for the iron-catalyzed diaminations of carboxylic acids, the reaction of β-amino acid (*R*)-**27b** (0.1 mmol, 1.0 equiv.), BocNHOMs (10.0 equiv.),

(S)-**Fe1** (15 mol%) and piperidine (120 µL, 12.0 equiv.) in $CHCl_3$ (1.0 mL, 0.1 M) was stirred at –15 ºC for 16 hours under N_2. After work-up, the solvent was evaporated under reduced pressure. The residue was purified by column chromatography on silica gel using the previous mentioned solvent as the eluent. The *N*-Boc-protected α,β-di-amino acid (2*R*,3*R*)-**27** was detected and isolated in 45% yield (70% conv.) with 4:1 dr.

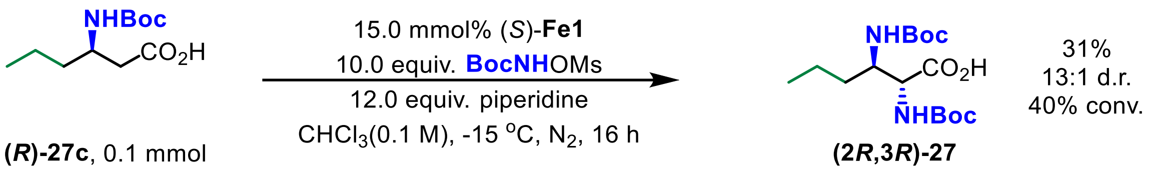

Following the **general procedure** for the iron-catalyzed diaminations of carboxylic acids, the reaction of β-amino acid (R)-**27c** (0.1 mmol, 1.0 equiv.), BocNHOMs (10.0 equiv.), (S)-**Fe1** (15 mol%) and piperidine (120 µL, 12.0 equiv.) in $CHCl_3$ (1.0 mL, 0.1 M) was stirred at –15 ºC for 16 hours under N_2. After work-up, the solvent was evaporated under reduced pressure. The residue was purified by column chromatography on silica gel using the previous mentioned solvent as the eluent. The *N*-Boc-protected α,β-di-amino acid (2*R*,3*R*)-**27** was detected and isolated in 31% yield (40% conv.) with 13:1 dr.

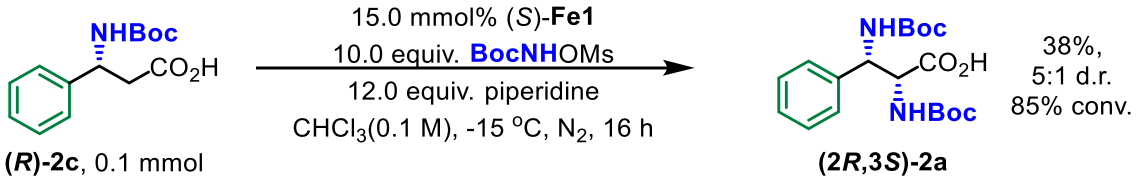

Following the **general procedure** for the iron-catalyzed diaminations of carboxylic acids, the reaction of β-amino acid (R)-**2c** (0.1 mmol, 1.0 equiv.), BocNHOMs (10.0 equiv.), (S)-**Fe1** (15 mol%) and piperidine (120 µL, 12.0 equiv.) in $CHCl_3$ (1.0 mL, 0.1 M) was stirred at –15 ºC for 16 hours under N_2. After work-up, the solvent was evaporated under reduced pressure. The residue was purified by column chromatography on silica gel using the previous mentioned solvent as the eluent. The *N*-Boc-protected α,β-di-amino acid (2*R*,3*S*)-**2a** was detected and isolated in 38% yield (85% conv.) with 5:1 dr.

5.3 Decomposition experiment

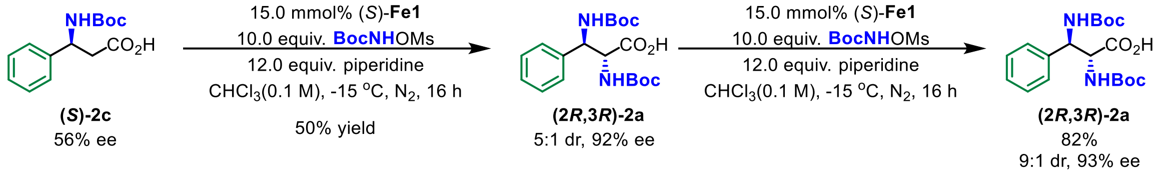

The diamino acid (2*R*,3*R*)-**2a** with relatively low dr and ee value (5:1 dr, 92% ee) was prepared from (*S*)-**2c** (56% ee) following the **general procedure** and reacted at –15 ºC for 16 hours under N_2. The diamino acid (2*R*,3*R*)-**2a** (5:1 dr, 92% ee, 1.0 equiv.) together with BocNHOMs (10.0 equiv.), (*S*)-**Fe1** (15 mol%) and piperidine (12.0 equiv.) in $CHCl_3$ (1.0 mL, 0.1 M) were stirred at –15 ºC for 16 hours under N_2. After work-up, the solvent was evaporated under reduced pressure. The residue was purified by column chromatography on silica gel using the previous mentioned solvent as the eluent. The obtained (2*R*,3*R*)-**2a** was isolated in 82% yield with 9:1 dr and 93% ee.

5.4 Determination of *syn*/*anti*-diastereoselectivity and absolute configuration

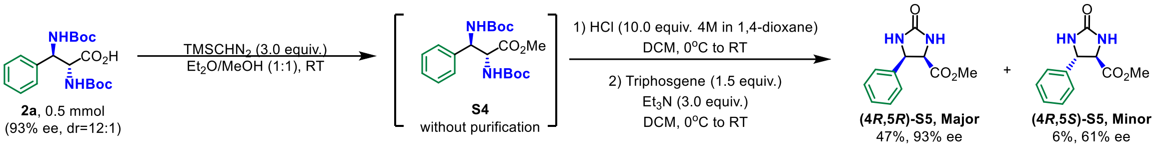

To a solution of α,β-diamino acid **2a** (0.5 mmol) in dry MeOH/Et_2O (5 mL /7.5 mL) was added $TMSCHN_2$ (0.6 mol/L in hexane, 2.5 mL, 1.5 mmol, 3.0 equiv.) at 0 °C. Then, the reaction mixture was warmed to room temperature and stirred for 2 h. After completion, the reaction mixture was concentrated under reduced pressure to afford the *N*-Boc-protected α,β-di-amino ester **S4** without purification. To a solution of the *N*-Boc-protected α,β-di-amino ester in dry DCM (5.0 mL) was added HCl solution (1.25 mL, 10.0 equiv., 4 M in 1,4-dioxane) at 0 °C and stirred for 2 h at room temperature. After removal of the solvent under reduced pressure, the residue was redissolved in fresh DCM (3x20 ml) and re-evaporated. To this residue, dry DCM (20 ml) was added, and the mixture was cooled to 0 °C. To this stirred solution Et_3N (0.21 ml, 1.5 mmol, 3.0 equiv.) was added dropwise, and then triphosgene (222.5 mg, 0.75 mmol, 1.5 equiv.) in DCM (5 ml). The reaction mixture was then stirred for 1 hour at 0 °C, and for 2 hours at room temperature. After removal of the solvent under reduced pressure, the residue was treated with EtOAc (4x40 ml), the solvent was evaporated under reduced pressure. The residue was purified by column chromatography eluenting with DCM/MeOH + 0.1% ammonia = 100/1 to afford the desired product **S5** in 47% (10.3 mg), 93% ee (major); 6% (1.3 mg), 61% ee (minor).

Methyl (4*R*,5*R*)-2-oxo-5-phenylimidazolidine-4-carboxylate (S5, Major)

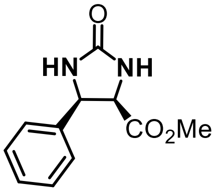

^{1}H NMR (300 MHz, CD_3OD) δ 7.33 (s, 5H), 5.22 (d, J = 9.7 Hz, 1H), 4.65 (d, J = 9.7 Hz, 1H), 3.16 (s, 3H). Enantiomeric excess was determined by HPLC analysis on a chiral stationary phase, e.e. = 93%. [HPLC: Chiralpak ADH column, 250 x 4.6 mm, Agilent HPLC 1260, *i*PrOH/*n*-hexane = 80/20 (v/v), 1.0 mL/min, 25 ºC, 210 nm; t_1 = 8.8 min, t_2 = 10.6 min]: $[\alpha]_D^{25}$ = -90.61° (*c* 0.5, MeOH, 93% e.e.). NMR and spectroscopic data of **S5** are in

agreement with the reported literature.[18] Comparison of the optical rotation leads to an assignment that the configuration of **S5 (Major)** is 4*R*, 5*R*.[18] Therefore, the configuration of product **2a** (major) is *cis*-2*R*, 3*R*.

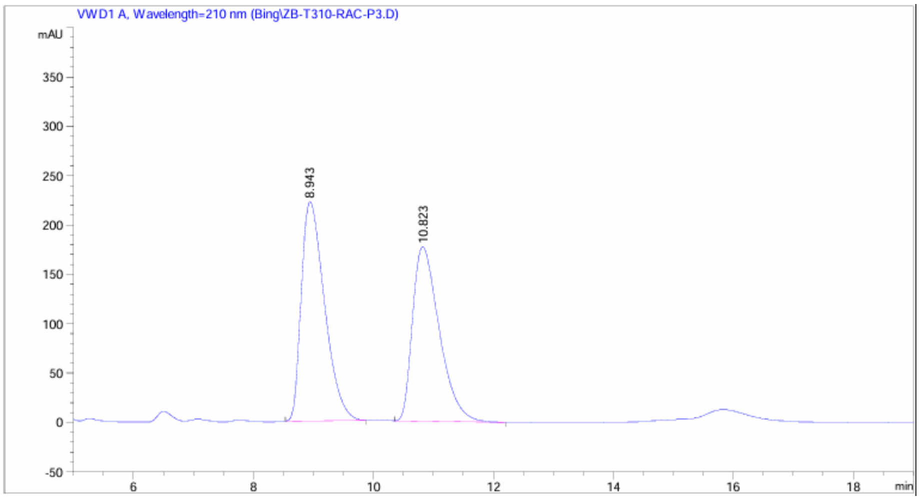

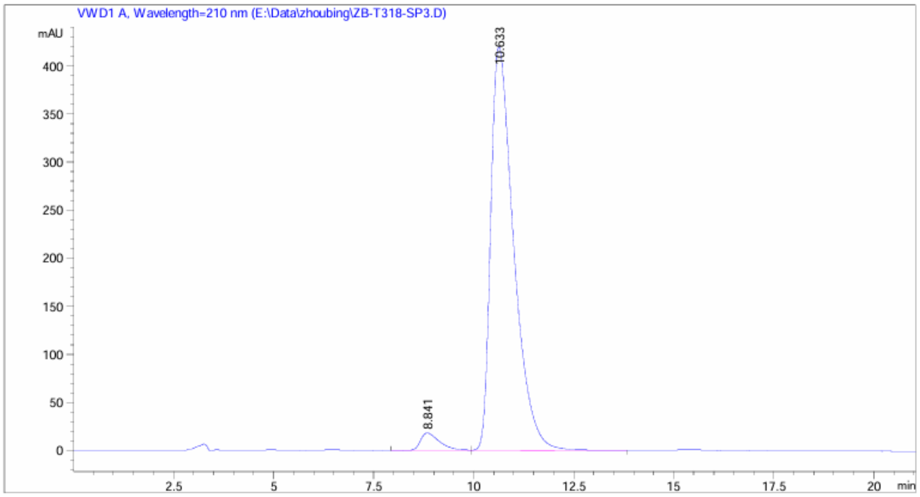

Peak #	RetTime [min]	Type	Width [min]	Area [mAU*s]	Height [mAU]	Area %
1	8.841	BV	0.4990	601.17804	18.12383	3.4627
2	10.633	VB	0.6063	1.67605e4	419.37582	96.5373

Figure S10. HPLC traces of *rac*-**S5** (Major) and enantioenriched-**S5** (Major).

Methyl (4*R*,5*S*)-2-oxo-5-phenylimidazolidine-4-carboxylate (S5, Minor)

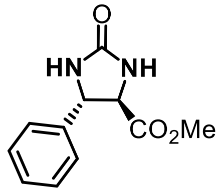

[11]**H NMR** (300 MHz, CD_3OD) δ 7.45 – 7.27 (m, 5H), 4.88 (d, J = 4.6 Hz, 1H), 4.11 (d, J = 4.6 Hz, 1H), 3.82 (s, 3H). Enantiomeric excess was determined by HPLC analysis on a chiral stationary phase, e.e. = 61%. [HPLC: Chiralpak ADH column, 250 x 4.6 mm, Agilent HPLC 1260, *i*PrOH/*n*-hexane = 80/20 (v/v), 1.0 mL/min, 25 ºC, 210 nm; t_1 = 9.2 min, t_2 = 15.7 min]: $[\alpha]_D^{25}$ = -37.9° (*c* 0.5, MeOH, 61% e.e.). NMR and spectroscopic data of **S5** are in agreement with the reported literature.[19] Comparison of the optical rotation leads to an assignment that the configuration of **S5 (Minor)** is 4*R*, 5*S*.[19] Therefore, the configuration of product **2a** (minor) is *trans*-2*R*, 3*S*.

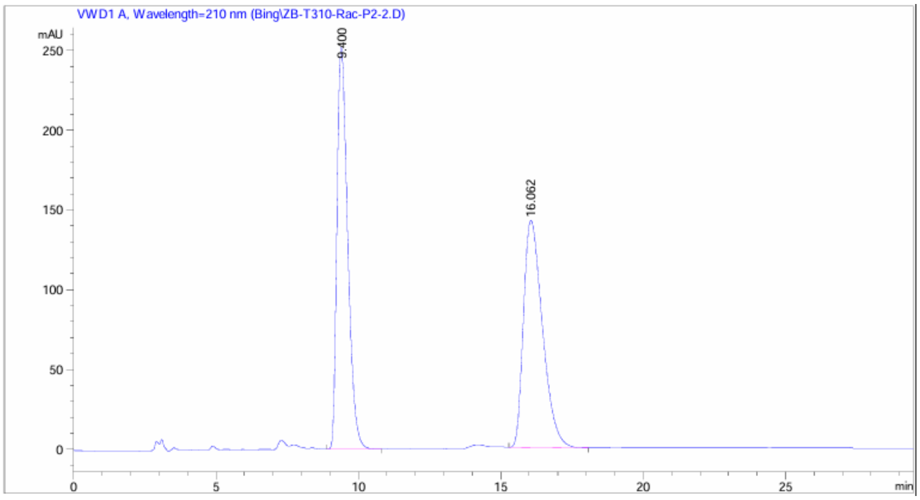

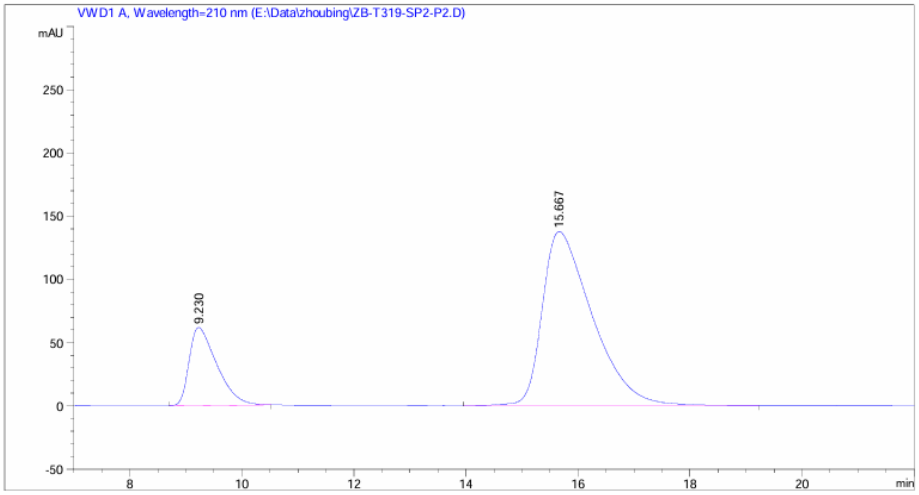

Peak #	RetTime [min]	Type	Width [min]	Area [mAU*s]	Height [mAU]	Area %
1	9.230	MM R	0.5718	2117.88208	61.73007	19.7056
2	15.667	MM R	1.0427	8629.73926	137.93852	80.2944

Figure S11. HPLC traces of *rac*-**S5** (Minor) and enantioenriched-**S5** (Minor).

6. Follow-Up Chemistry

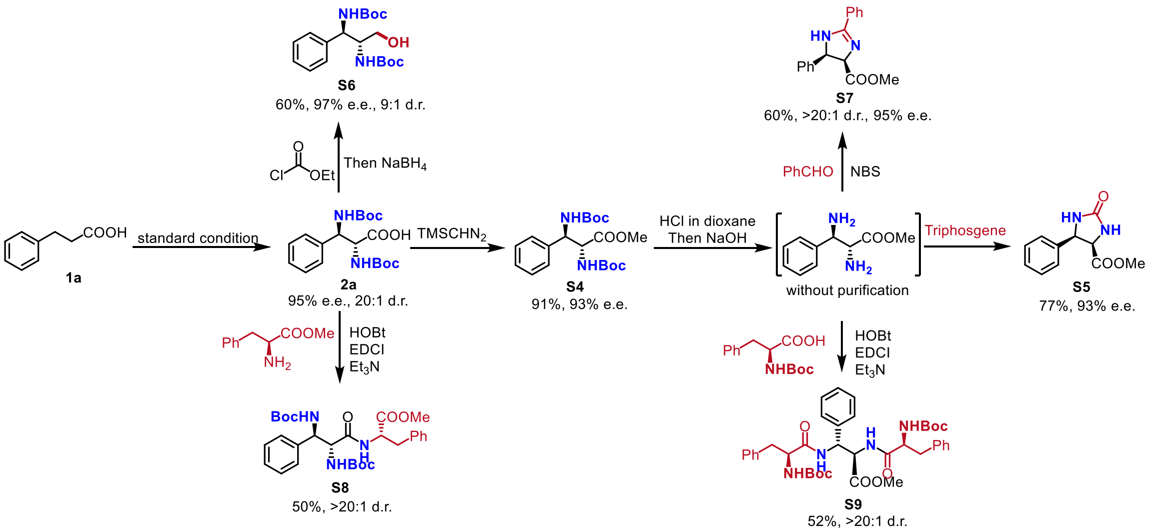

6.1 Esterification reaction

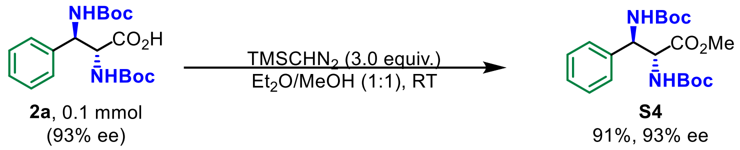

To a solution of α,β-di-amino acid **2a** (38.0 mg, 0.1 mmol) in dry MeOH/Et_2O (1 mL /2.5 mL) was added $TMSCHN_2$ (0.6 mol/L in hexane, 0.5 mL, 0.3 mmol, 3.0 equiv.) at 0 °C. Then, the reaction mixture was warmed to room temperature and stirred for 2 h. After completion, the reaction mixture was concentrated under reduced pressure and the residue was purified by column chromatography eluenting with *n*-hexane/EtOAc = 6/1 to afford the desired product **S4** as white powder (35.7 mg, 91%) with 93% e.e. [HPLC: Chiralpak IA column, 250 x 4.6 mm, Agilent HPLC 1260, *i*PrOH/*n*-hexane = 5/95 (v/v), 1.0 mL/min, 25 ºC, 210 nm; t_1 = 18.2 min, t_2 = 29.2 min.]: $[\alpha]_D^{25}$ = −36.4° (c 0.5, MeOH, 93% e.e.). **^{1}H NMR** (500 MHz, Acetone-*d*$_6$) δ 7.42 (d, *J* = 7.7 Hz, 2H), 7.33 (t, *J* = 7.5 Hz, 2H), 7.28 (d, *J* = 7.3 Hz, 1H), 6.65 (d, *J* = 9.1 Hz, 1H), 6.04 (d, *J* = 9.0 Hz, 1H), 5.12 (t, *J* = 8.5 Hz, 1H), 4.66 (t, *J* = 8.2 Hz, 1H), 3.67 (s, 3H), 1.38 (s, 9H), 1.32 (s, 9H). **^{13}C NMR** (126 MHz, Acetone-*d*$_6$) δ 171.7, 156.1, 155.7, 140.0, 129.1, 128.4, 128.3, 79.7, 79.2, 58.7, 56.9, 52.4, 28.5, 28.4. **HRMS** (+ESI) Exact mass calculated for $[C_{20}H_{30}N_2O_6Na]^+$ $[(M + Na)^+]$: 417.1996, found: 417.1985.

6.2 Synthesis of urea

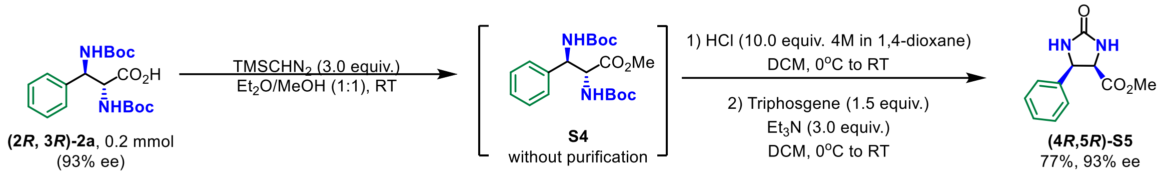

To a solution of α,β-di-amino acid (2*R*,3*R*)-**2a** (76.0 mg, 0.2 mmol) in dry MeOH/Et_2O (1 mL /2.5 mL) was added $TMSCHN_2$ (0.6 mol/L in hexane, 1.0 mL, 0.6 mmol, 3.0 equiv.) at 0 °C. Then, the reaction mixture was warmed to room temperature and stirred for 2 h. After completion, the reaction mixture was concentrated under reduced pressure to afford the *N*-Boc-protected α,β-di-amino ester without purification. To a solution of the *N*-Boc-protected α,β-di-amino ester in dry DCM (2.0 mL) was added HCl solution (0.5 mL, 10.0 equiv., 4 M in 1,4-dioxane) at 0 °C and stirred for 2 h at room temperature. After removal of the solvent under reduced pressure, the residue was redissolved in fresh DCM (3x10 ml) and re-evaporated. To this residue, dry DCM (8 ml) was added, and the mixture was cooled to 0 °C. To this stirred solution Et_3N (84 μL, 0.6 mmol, 3.0 equiv.) was added dropwise, and then triphosgene (89.0 mg, 0.3 mmol, 1.5 equiv.) in DCM (2 mL). The reaction mixture was then stirred for 1 hour at 0 °C, and for 2 hours at room temperature. After removal of the solvent under reduced pressure, the residue was treated with EtOAc (4x10 ml), the solvent was evaporated under reduced pressure. The residue was purified by column chromatography eluenting with DCM/MeOH + 0.1% ammonia = 100/1 to afford the desired product (4*R*,5*R*)-**S5** in 77% (17.0 mg), 93% e.e. Characterization data could be found in **Section 5.4** which matched previous reports[18,19].

6.3 Reduction of carboxylic acid

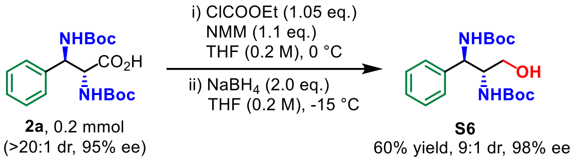

Ethyl chloroformate (28 μL, 0.22 mmol, 1.1 equiv.) was added dropwise to a stirred THF (1.0 mL, 0.2 M) solution of **2a** (0.2 mmol, 1.0 equiv.) and 4-methylmorpholine (NMM) (24 μL, 0.21 mmol, 1.05 equiv.) at 0 °C. The mixture was allowed to stir at 0 °C for another 1 hour, then filtered through a short plug of Celite. The filtrate was cooled to -15 °C, and $NaBH_4$ (16.0 mg, 0.4 mmol, 2.0 equiv.) was added. After stirring at -15 °C for 16 h, saturated NH_4Cl (aq.) was added to quench excess $NaBH_4$. The mixture was extracted with EA (50 mL), and the organic phase was washed with NH_4Cl (sat.) and brine, dried over anhydrous Na_2SO_4, and concentrated under reduced pressure. The residue was purified by flash column chromatography eluenting with MeOH/DCM (1:100) to afford di-*tert*-butyl ((1*R*,2*R*)-3-hydroxy-1-phenylpropane-1,2-diyl)dicarbamate **S6** as white foam (44.0 mg, 60% yield, 9:1 d.r.) with 98% e.e. [DAICEL CHIRALPAK IC column, Agilent HPLC 1260, ^{i}PrOH/*n*-hexane = 9.0/91.0 (v/v), 1.0 mL/min, 25 ºC, 210 nm; t_1 = 12.5 min, t_2 = 37.2 min]: $[\alpha]_D^{25}$ = -56.8° (c 0.5, MeOH, 98% e.e.). **^{1}H NMR** (300 MHz, $CDCl_3$) δ 7.36 – 7.27 (m, 5H), 5.40 – 5.24 (m, 1H), 5.04 (d, *J* = 9.6 Hz, 1H), 4.71 (t, *J* = 8.6 Hz, 1H), 3.92 (t, *J* = 9.3 Hz, 1H), 3.85 – 3.69 (m, 1H), 3.69 – 3.54 (m, 1H), 3.54 – 3.32 (m, 1H), 1.47 – 1.26 (m, 18H). **^{13}C NMR** (76 MHz, $CDCl_3$) δ 156.64, 155.26, 139.11, 129.01, 128.82, 127.99, 127.54, 127.24, 80.67, 79.50, 62.37, 62.28, 56.14, 55.35, 28.45, 28.33. **HRMS** (–ESI) Exact mass calculated for $[C_{19}H_{29}N_2O_5^-$: $[(M - H)^-]$: 365.2082, found: 365.2084.

6.4 Synthesis of imidazoline

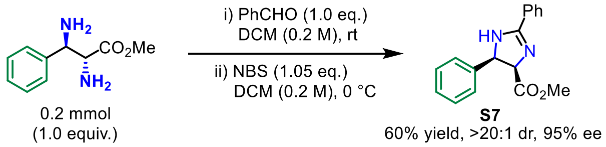

The diamine ester (prepared following the procedure described in section **6.2** using 4.0 M

HCl dioxane solution as reagent, the diamine was directly used after basification with saturated Na_2CO_3 solution) (0.2 mmol) and benzaldehyde (17 µL, 0.2 mmol, 1.0 equiv.) were mixed in DCM (1.0 mL, 0.2 M) and stirred at room temperature for 2.0 h. Then the reaction was cooled to 0 °C and *N*-bromosuccinimide (38.3 mg, 0.21 mmol) was added slowly. The reaction was allowed to warm to room temperature over 1.5 h and quenched with $Na_2S_2O_3$ (sat.) and 5% KOH (aq.). The aqueous phase was extracted with DCM (3x), dried over anhydrous Na_2SO_4, and concentrated under reduced pressure. The residue was purified by flash column chromatography eluenting with MeOH/DCM/Et_3N (1:18:1) to afford methyl (4*R*,5*R*)-2,5-diphenyl-4,5-dihydro-1*H*-imidazole-4-carboxylate **S7** as yellow gum (33.6 mg, 60% yield, >20:1 d.r.) with 95% e.e. [DAICEL CHIRALPAK IA column, Agilent HPLC 1260, ^{i}PrOH/*n*-hexane = 10.0/90.0 (v/v) with 0.1% DEA, 1.0 mL/min, 25 °C, 210 nm; t_1 = 22.4 min, t_2 = 34.3 min]: $[\alpha]_D^{25} = +45.6^{\circ}$ (c 0.25, MeOH, 95% e.e.). **^{1}H NMR** (300 MHz, CD_3OD) δ 7.97 – 7.85 (m, 2H), 7.60 – 7.47 (m, 3H), 7.43 – 7.29 (m, 6H), 5.28 (d, *J* = 7.1 Hz, 1H), 4.48 (d, *J* = 7.0 Hz, 1H), 3.83 (s, 3H). **^{13}C NMR** (126 MHz, CD_3OD) δ 174.12, 166.87, 143.98, 132.90, 129.94, 129.92, 129.79, 129.05, 128.88, 127.42, 72.65, 69.48, 53.08. **HRMS** (+ESI) Exact mass calculated for $[C_{17}H_{17}N_2O_2^+$: $[(M + H)^+]$: 281.1285, found: 281.1278.

6.5 Synthesis of dipeptide

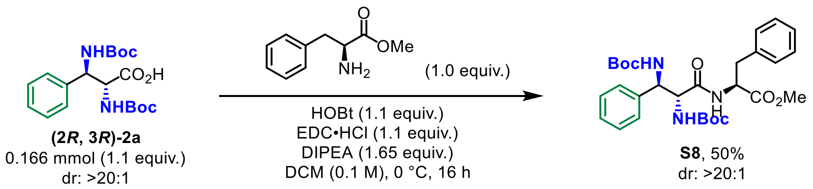

To a 10 mL flask containing 2.0 mL DCM with a stir bar was added amino acid (2*R*,3*R*)-**2a** (63.2 mg, 0.166 mmol, 1.1 equiv.), EDC·HCl (31.8 mg, 0.166 mmol, 1.1 equiv.), hydroxybenzotriazole (HOBt) (22.4 mg, 0.166 mmol, 1.1 equiv.) and DIPEA (44 µL, 0.249 mmol, 1.65 equiv.) at 0 °C. After stirring at this temperature for 10 minutes, *L*-phenylalanine methyl ester (32.5 mg, 0.151 mmol) was added in one portion and the mixture was stirred at 0 °C for 16 h. The reaction was warmed to room temperature and suspended in EA (20 mL). The organic phase was washed sequentially with 5% citric acid

solution, saturated $NaHCO_3$ solution and brine, dried over anhydrous Na_2SO_4, and concentrated under vacuum. The residue was purified by flash column chromatography eluenting with EtOAc/n-hexane (1:8 to 1:2) to afford dipeptide **S8** as white powder (40.9 mg, 50% yield). $[\alpha]_D^{25} = +24.7^\circ$ (c 1.0, $CHCl_3$, >20:1 d.r.). **^{1}H NMR** (300 MHz, $CDCl_3$) δ 7.40 – 7.16 (m, 9H), 6.78 (dd, *J* = 6.6, 2.8 Hz, 2H), 6.44 (s, 1H), 5.45 (d, *J* = 8.3 Hz, 1H), 5.20 – 5.05 (m, 1H), 4.72 (q, *J* = 6.3 Hz, 1H), 4.48 (dd, *J* = 8.0, 3.8 Hz, 1H), 3.67 (s, 3H), 2.97 (dd, *J* = 13.8, 6.0 Hz, 1H), 2.80 (dd, *J* = 13.9, 5.9 Hz, 1H), 1.47 (s, 18H). **^{13}C NMR** (75 MHz, $CDCl_3$) δ 171.0, 155.8, 138.4, 135.3, 129.0, 128.62, 128.58, 127.7, 127.1, 126.4, 80.6, 79.9, 57.4, 56.5, 53.0, 52.3, 37.6, 28.3, 28.2. **HRMS** (+ESI) Exact mass calculated for $[C_{29}H_{39}N_3O_7Na]^+$ $[(M + Na)^+]$: 564.2680, found: 564.2670.

6.6 Synthesis of tripeptide

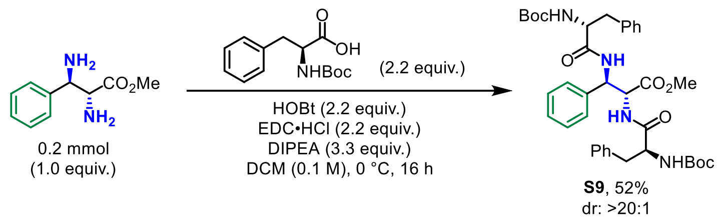

To a 10 mL flask containing 3.0 mL DCM with a stir bar was added *N*-Boc-*L*-phenylalanine (150.0 mg, 0.565 mmol, 2.2 equiv.), EDC·HCl (108.1 mg, 0.565 mmol, 2.2 equiv.), hydroxybenzotriazole (HOBt) (76.4 mg, 0.565 mmol, 2.2 equiv.) and DIPEA (150 μL, 0.848 mmol, 3.3 equiv.) at 0 °C. After stirring at this temperature for 10 minutes, the crude diamine (0.2 mmol) dissolved in 3 mL DCM was wadded in one portion and the mixture was stirred at 0 °C for 16 h. The reaction was warmed to room temperature and suspended in EA (30 mL). The organic phase was washed sequentially with 5% citric acid solution, saturated $NaHCO_3$ solution and brine, dried over anhydrous Na_2SO_4, and concentrated under vacuum. The residue was purified by flash column chromatography eluenting with EtOAc/n-hexane (1:5 to 2:3) to afford tripeptide **S9** as white powder (92.1 mg, 52% yield for two steps). $[\alpha]_D^{25} = -35.5^\circ$ (c 1.0, $CHCl_3$, >20:1 d.r.). **^{1}H NMR** (300 MHz, $CDCl_3$) δ 7.27 – 7.03 (m, 15H), 6.84 – 6.80 (m, 1H), 5.50 – 5.33 (m, 1H), 5.08 (s, 1H), 4.99 – 4.90 (m, 1H), 4.90 – 4.78 (m, 1H), 4.41 – 4.19 (m, 2H), 3.60 – 3.43 (m, 3H), 3.20 – 2.75 (m,

5H), 1.36 – 1.25 (m, 18H). **^{13}C NMR** (75 MHz, $CDCl_3$) δ 172.1, 170.8, 169.7, 155.3, 137.0, 136.4, 136.1, 129.3, 129.2, 128.7, 128.6, 128.5, 128.1, 127.0, 126.7, 126.5, 80.3, 80.2, 56.1, 55.8, 55.6, 55.2, 52.5, 38.0, 29.6, 28.3, 28.2. **HRMS** (+ESI) Exact mass calculated for $[C_{38}H_{48}N_4O_8\ Na]^+$ $[(M + Na)^+]$: 711.3364, found: 711.3343.

7. Single Crystal X-Ray Diffraction

Single crystals of (*R*)-**Fe1**(III) were obtained by slow diffusion from a solution of (*R*)-**Fe1**(III) (15 mg) in DCM layered with *n*-pentane at –20 ºC for 5 days.

A suitable crystal of $C_{40}H_{40}C_{l2}FeN_6(Cl) \cdot CH_2Cl_2$, was selected under inert oil and mounted using a MiTeGen loop. Intensity data of the crystal were recorded with a D8 Venture diffractometer (Bruker AXS). The instrument was operated with Mo-Kα radiation (0.71073 Å, microfocus source) and equipped with a PHOTON III C14 detector. Evaluation, integration and reduction of the diffraction data was carried out using the Bruker APEX 5 software suite.[20] Multi-scan and numerical absorption corrections were applied using the SADABS program.[21,22] The structure was solved using dual-space methods (SHELXT-2018/2) and refined against F^2 (SHELXL-2019/1 using ShelXle interface).[23-25] All non-hydrogen atoms were refined with anisotropic displacement parameters. The hydrogen atoms were refined using the “riding model” approach with isotropic displacement parameters 1.2 times (1.5 times for terminal methyl groups) of that of the preceding carbon atom. The SQUEEZE algorithm in the PLATON software was applied to eliminate the residual electron density in the solvent accessible voids.[26,27] CCDC 2434956 contains the supplementary crystallographic data for this paper. These data can be obtained free of charge from The Cambridge Crystallographic Data Centre via www.ccdc.cam.ac.uk/structures.

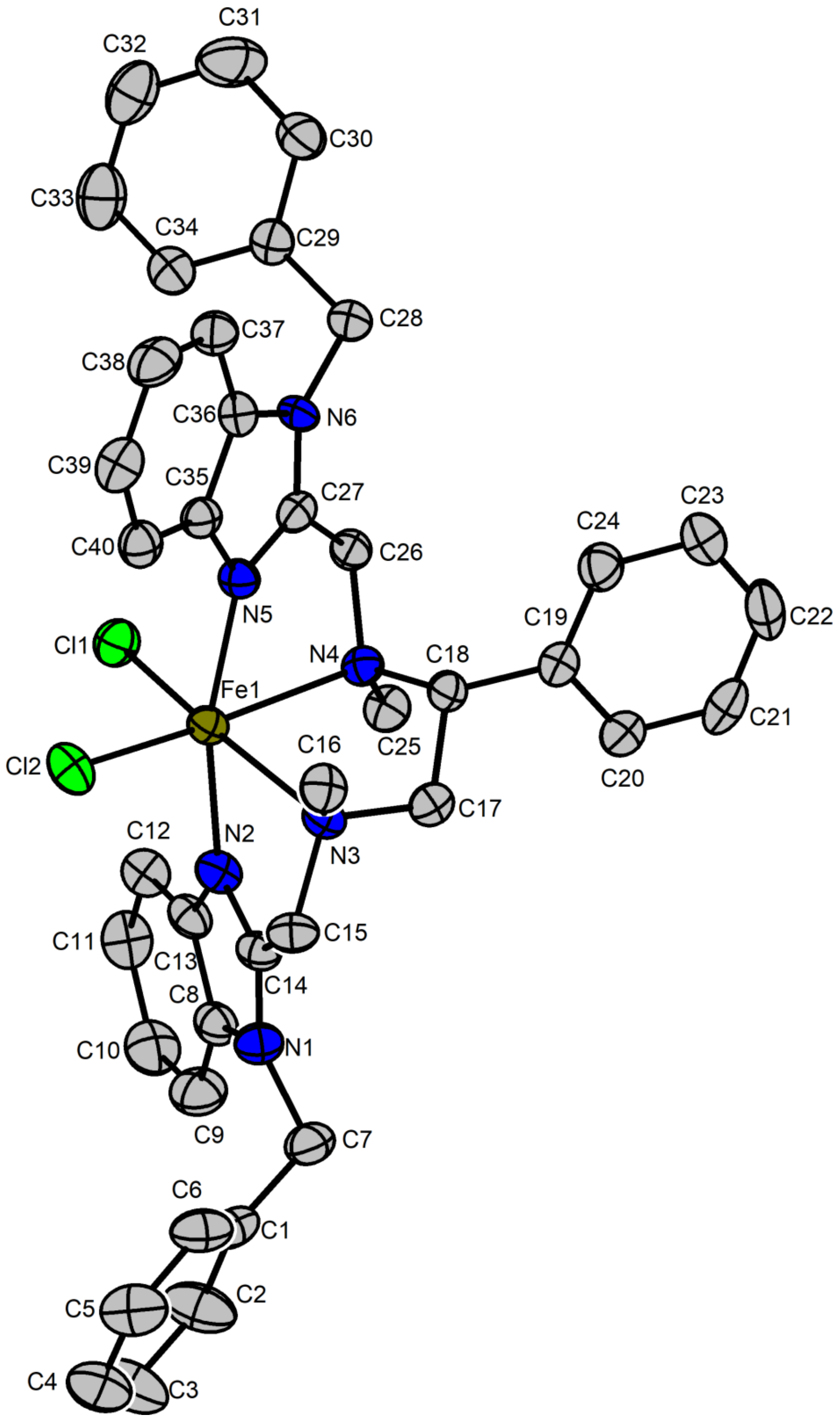

Figure S12. Crystal structure of (*R*)-**Fe1**(III). * The Cl^{-} anion, the DCM solvent molecule and the hydrogen atoms are not shown. Displacement ellipsoids are shown at 50 % probability level at 230 K.

Table S2. Selected crystallographic data and details of the structure determination for $C_{40}H_{40}Cl_2FeN_6(Cl) \cdot CH_2Cl_2$.

Identification code	(*R*)-**Fe1**(III)
Empirical formula	$C_{41}H_{42}Cl_5FeN_6$
Molar mass / g·mol^{-1}	851.90
Space group (No.)	$P2_12_12_1$ (19)
a / Å	8.2684(3)
b / Å	16.2633(7)
c / Å	31.5322(13)
V / Å^3	4240.2(3)
Z	4
$\rho_{calc.}$ / g·cm^{-3}	1.334
μ / mm^{-1}	0.707
Color	red
Crystal habitus	block
Crystal size / mm^3	0.091 x 0.064 x 0.063
T / K	230
λ / Å	0.71073 (Mo-K$_\alpha$)
θ range / °	2.307 to 25.065
Range of Miller indices	$-9 \leq h \leq 9$
	$-18 \leq k \leq 19$
	$-37 \leq l \leq 37$
Absorption correction	multi-scan and numerical
$T_{\min}$, $T_{\max}$	0.9158, 1.0000
R_{int}, R_σ	0.0363, 0.0278
Completeness of the data set	0.998
No. of measured reflections	46397
No. of independent reflections	7498
No. of parameters	481

No. of restraints	0
S (all data)	1.134
$R(F)$ ($I \geq 2\sigma(I)$, all data)	0.0456, 0.0508
$wR(F^2)$ ($I \geq 2\sigma(I)$, all data)	0.0975, 0.0996
Extinction coefficient	0.0010(3)
Flack parameter x	0.020(5)
$\Delta\rho_{max}$, $\Delta\rho_{min}$ / e·Å^{-3}	0.363, –0.412

8. Chiral HPLC analysis

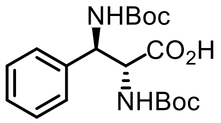

[CHIRALPAK IG reverse phase, 23 ºC, MeCN/Water (0.1% TFA) = 30/70, 1.0 mL/min, 210 nm]

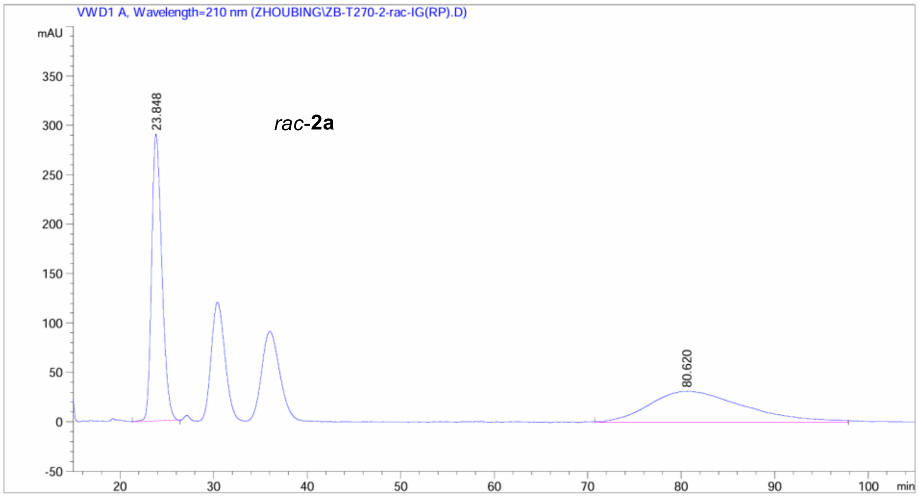

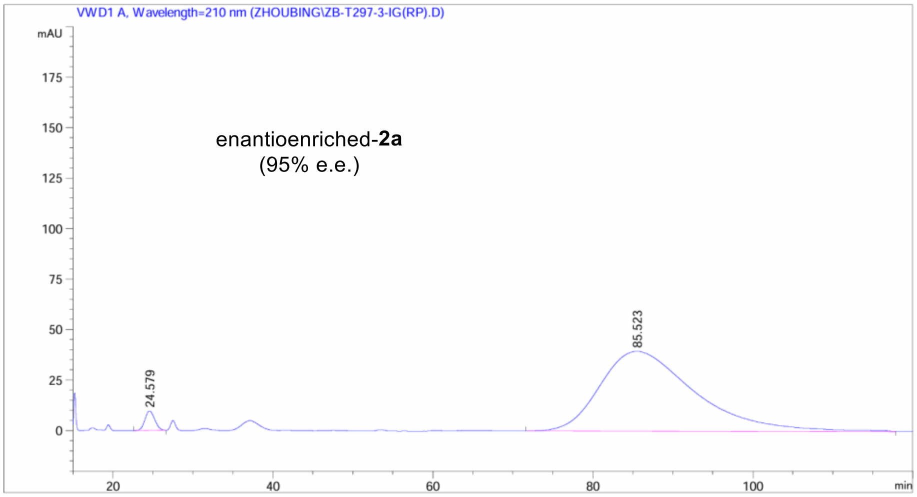

Peak #	RetTime [min]	Type	Width [min]	Area [mAU*s]	Height [mAU]	Area %
1	24.579	BB	1.2097	814.74670	9.61730	2.4441
2	85.523	MM R	13.7211	3.25202e4	39.50162	97.5559

Figure S13. HPLC traces of *rac*-**2a** and enantioenriched-**2a**.

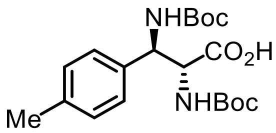

[CHIRALPAK AD-H, 25 ºC, *i*PrOH/*n*-hexane = 5/95 (v/v) + 0.1% TFA, 1.0 mL/min, 210 nm]

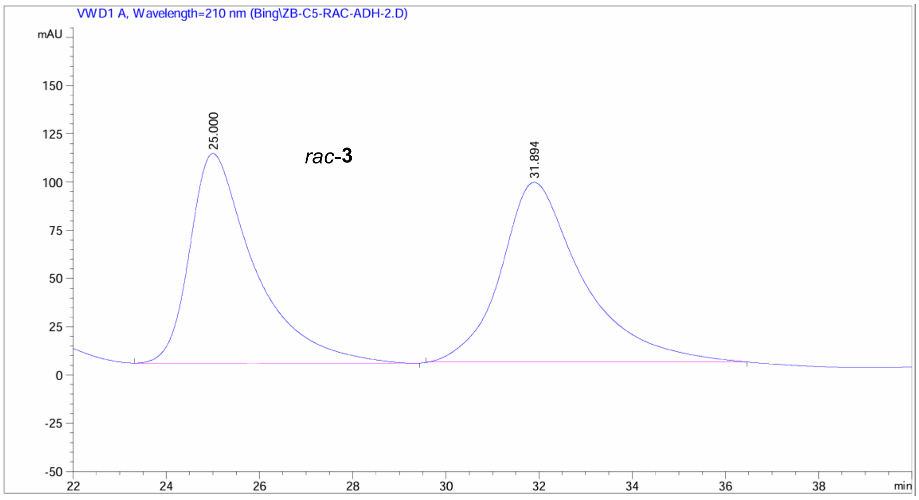

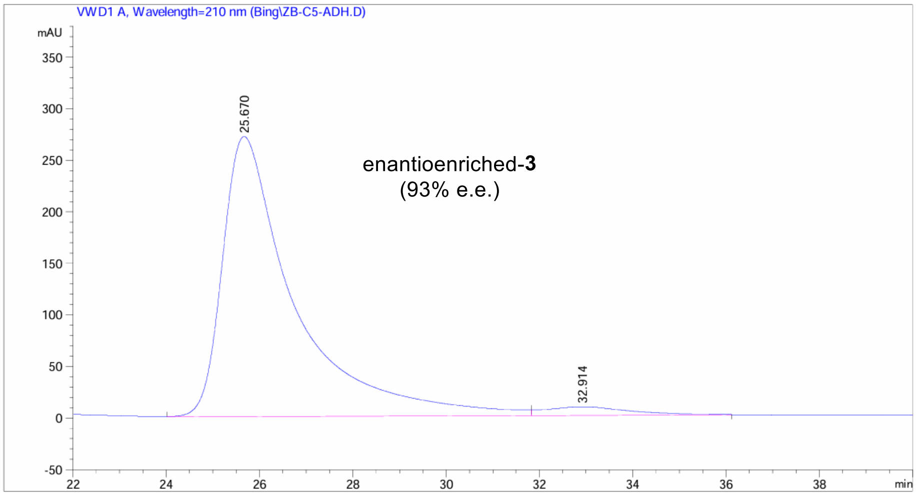

Peak #	RetTime [min]	Type	Width [min]	Area [mAU*s]	Height [mAU]	Area %
1	25.670	MF R	1.7906	2.91611e4	271.42624	96.5385
2	32.914	FM R	2.0876	1045.60181	8.34786	3.4615

Figure S14. HPLC traces of *rac*-**3** and enantioenriched-**3**.

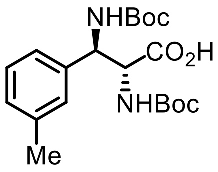

[CHIRALPAK IG reverse phase, 20 ºC, MeCN/Water (0.1% TFA) = 23/77, 1.0 mL/min, 210 nm]

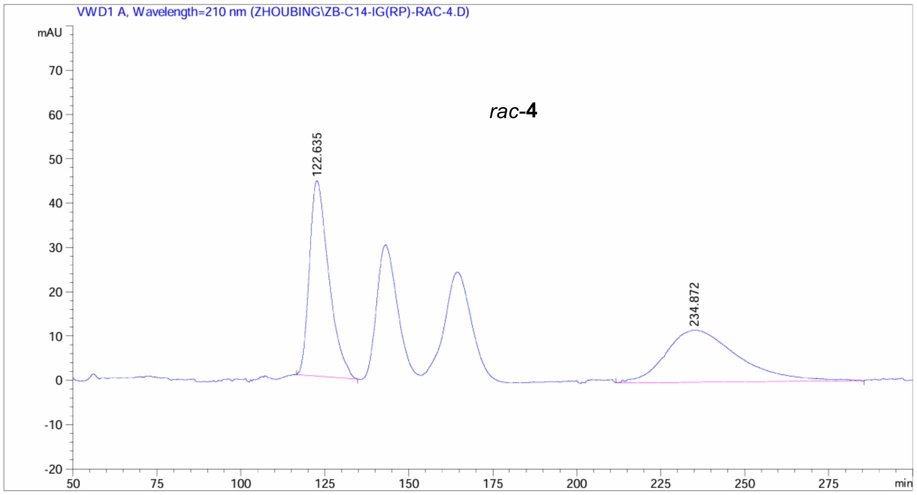

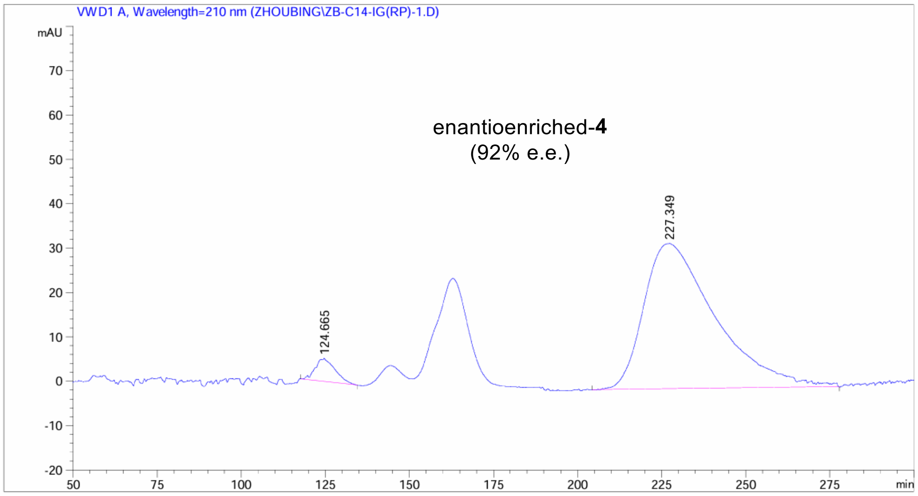

Peak #	RetTime [min]	Type	Width [min]	Area [mAU*s]	Height [mAU]	Area %
1	124.665	MM R	6.7491	2105.97998	5.20067	4.1878
2	227.349	MM R	24.5254	4.81820e4	32.74293	95.8122

Figure S15. HPLC traces of *rac*-**4** and enantioenriched-**4**.

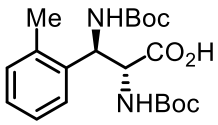

[CHIRALPAK IB-N5 reverse phase, 25 ºC, MeCN/Water (0.1% TFA) = 25/75, 1.0 mL/min, 210 nm]

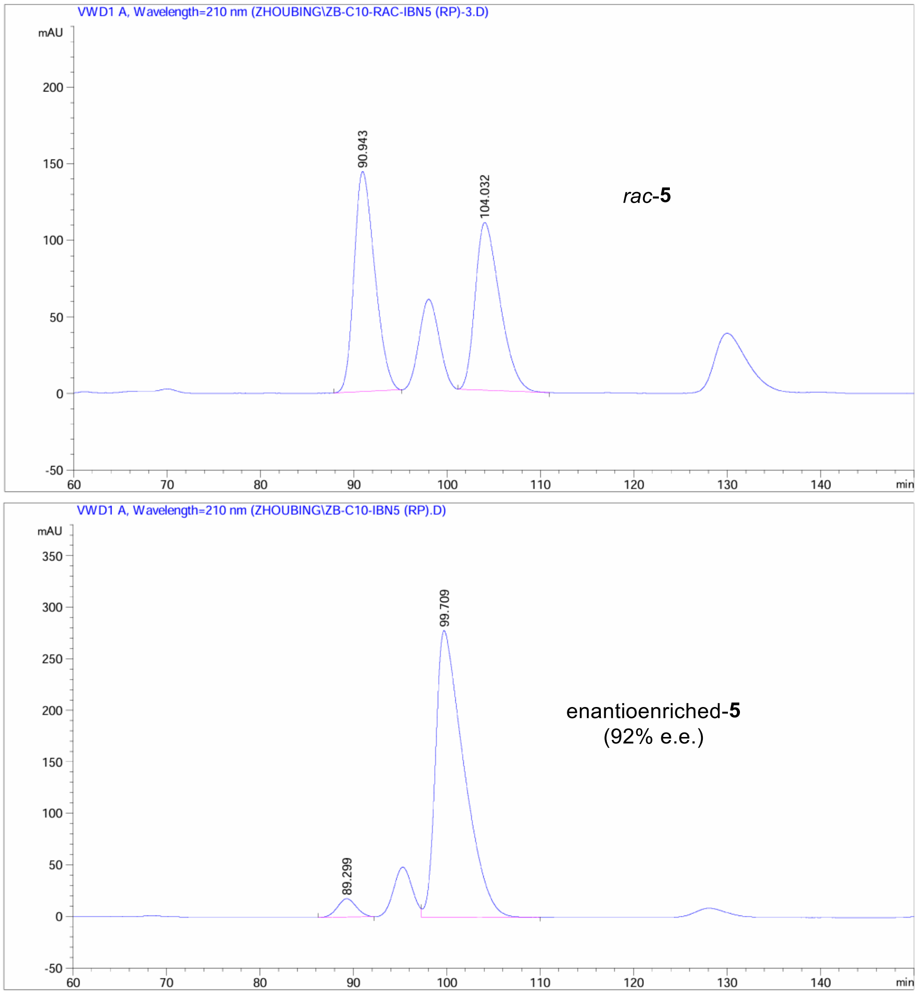

Peak #	RetTime [min]	Type	Width [min]	Area [mAU*s]	Height [mAU]	Area %
1	89.299	BB	1.6964	2482.75610	17.71314	4.0631
2	99.709	FM R	3.5172	5.86223e4	277.78494	95.9369

Figure S16. HPLC traces of *rac*-**5** and enantioenriched-**5**.

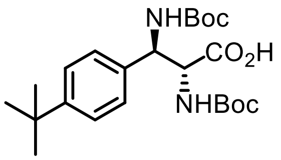

[CHIRALPAK IA, 25 ºC, *i*PrOH/*n*-hexane = 5/95 (v/v) + 0.1% TFA, 0.8 mL/min, 210 nm]

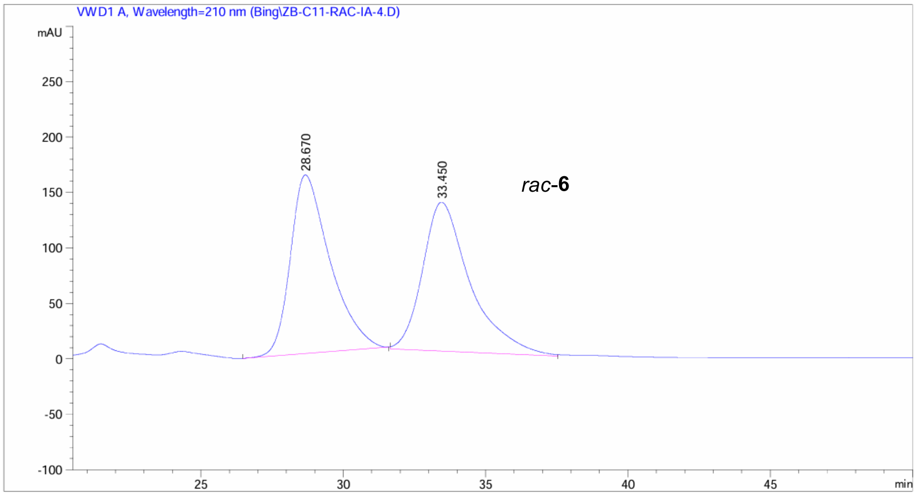

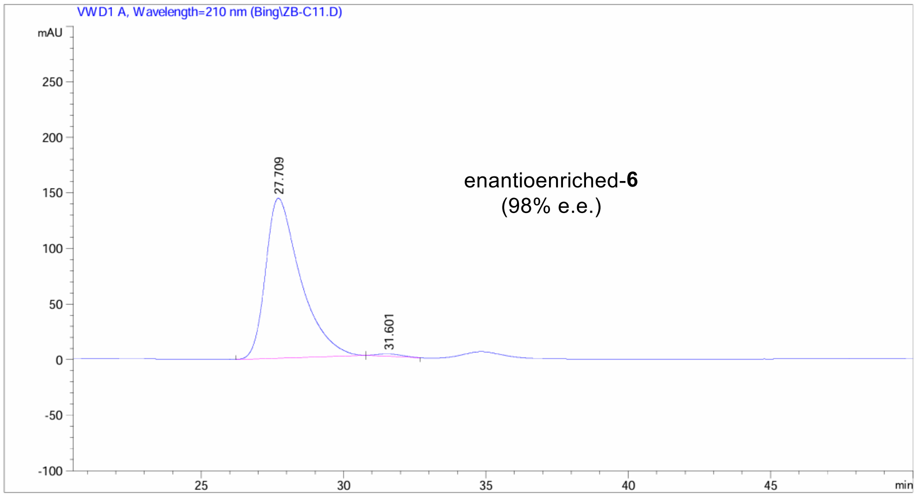

Peak #	RetTime [min]	Type	Width [min]	Area [mAU*s]	Height [mAU]	Area %
1	27.709	MM R	1.4233	1.22821e4	143.81758	98.9503
2	31.601	MM R	0.9859	130.29205	2.20248	1.0497

Figure S17. HPLC traces of *rac*-**6** and enantioenriched-**6**.

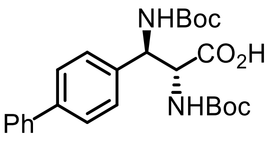

[CHIRALPAK IA, 25 ºC, *i*PrOH/*n*-hexane = 10/90 (v/v) + 0.1% TFA, 1.0 mL/min, 210 nm]

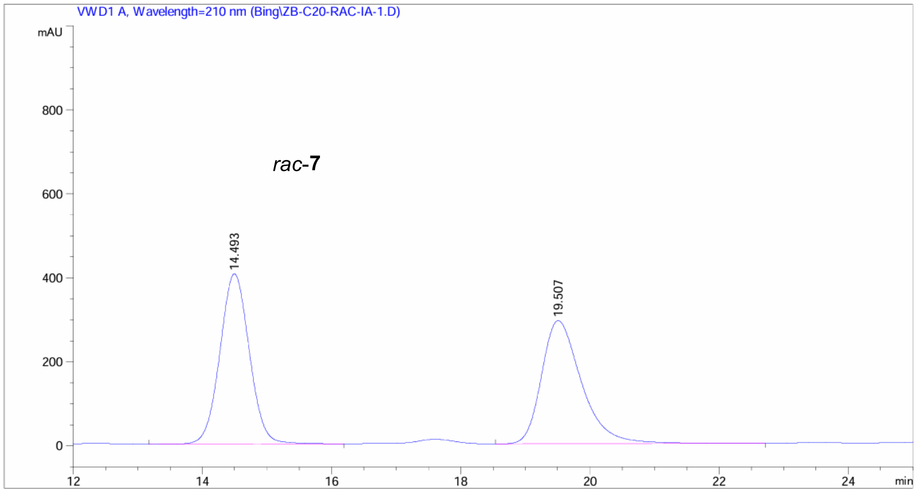

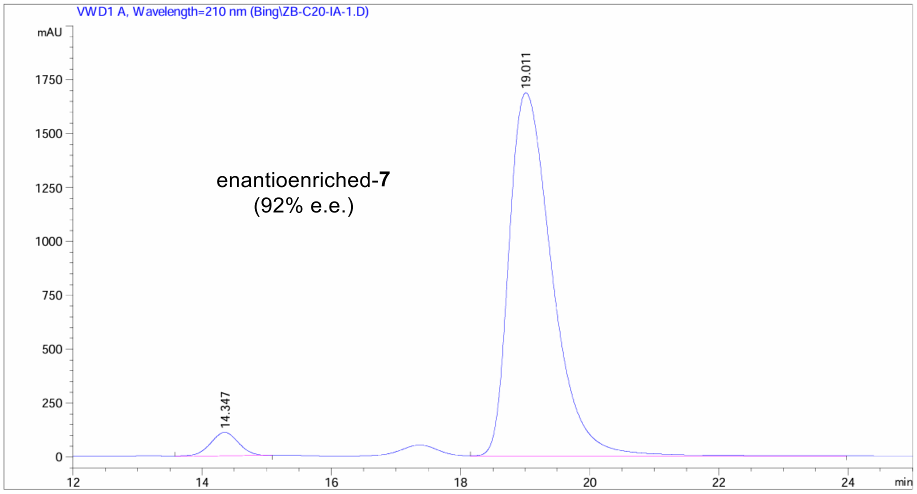

Peak #	RetTime [min]	Type	Width [min]	Area [mAU*s]	Height [mAU]	Area %
1	14.347	MM R	0.5022	3271.80640	108.59093	4.0249
2	19.011	MF R	0.7712	7.80179e4	1686.12097	95.9751

Figure S18. HPLC traces of *rac*-**7** and enantioenriched-**7**.

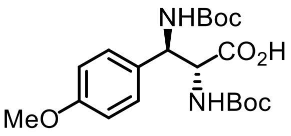

[CHIRALPAK IA, 20 ºC, *i*PrOH/*n*-hexane = 10/90 (v/v) + 0.1% TFA, 1.0 mL/min, 210 nm]

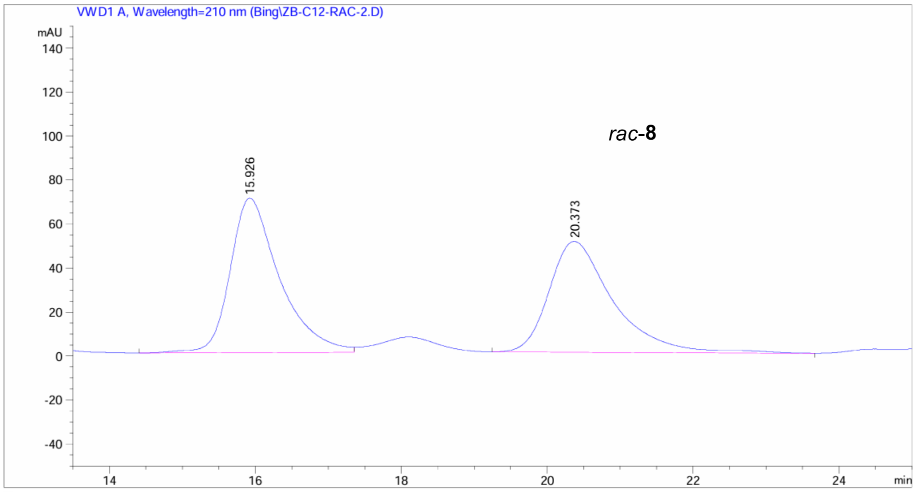

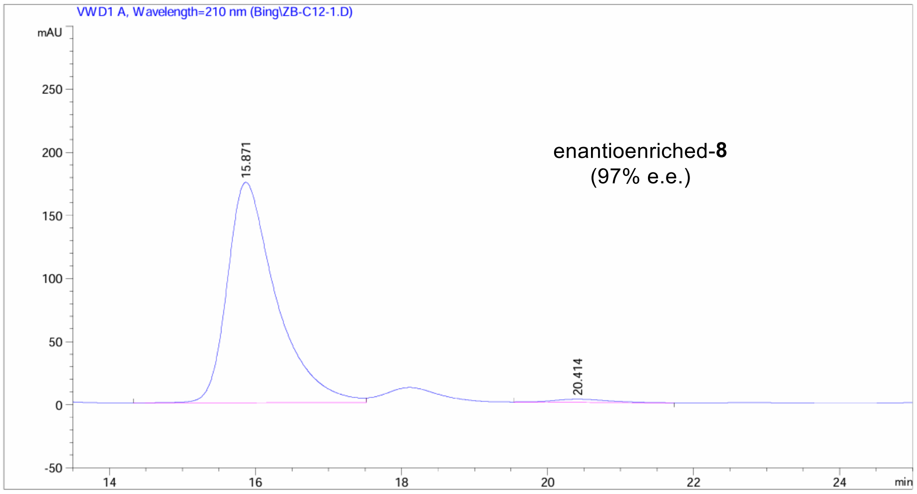

Peak #	RetTime [min]	Type	Width [min]	Area [mAU*s]	Height [mAU]	Area %
1	15.871	MF R	0.7735	8106.41748	174.68005	98.4740
2	20.414	BB	0.6363	125.62210	2.44300	1.5260

Figure S19. HPLC traces of *rac*-**8** and enantioenriched-**8**.

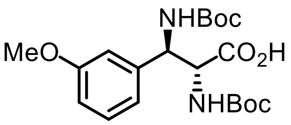

[CHIRALPAK IA, 25 ºC, *i*PrOH/*n*-hexane = 10/90 (v/v) + 0.1% TFA, 0.5 mL/min, 210 nm]

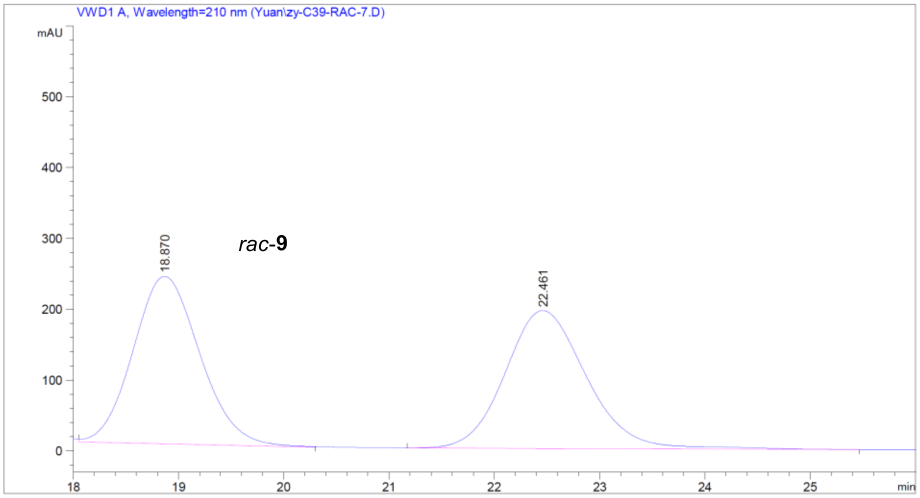

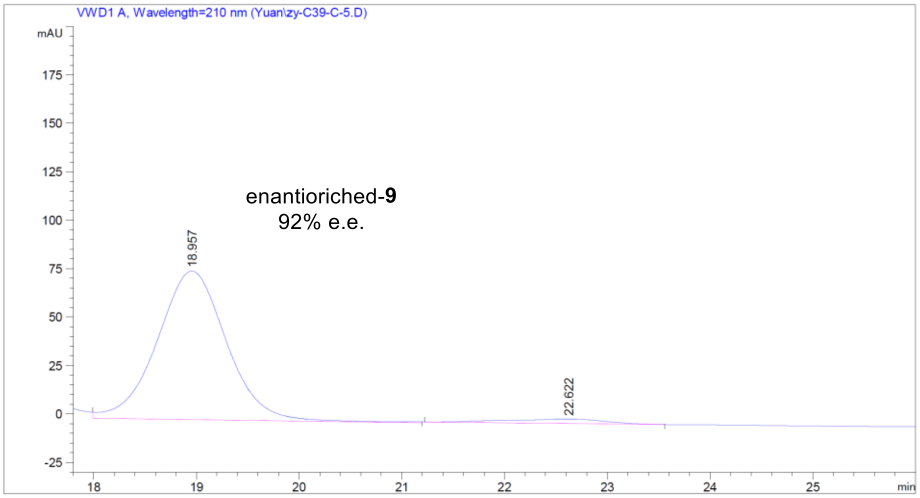

Peak #	RetTime [min]	Type	Width [min]	Area [mAU*s]	Height [mAU]	Area %
1	18.957	FM R	0.7811	3589.51440	76.59077	95.9594
2	22.622	MM R	1.0699	151.14638	2.35458	4.0406

Figure S20. HPLC traces of *rac*-**9** and enantioenriched-**9**.

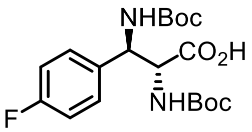

[CHIRALPAK IA, 25 ºC, *i*PrOH/*n*-hexane = 10/90 (v/v) + 0.1% TFA, 1.0 mL/min, 210 nm]

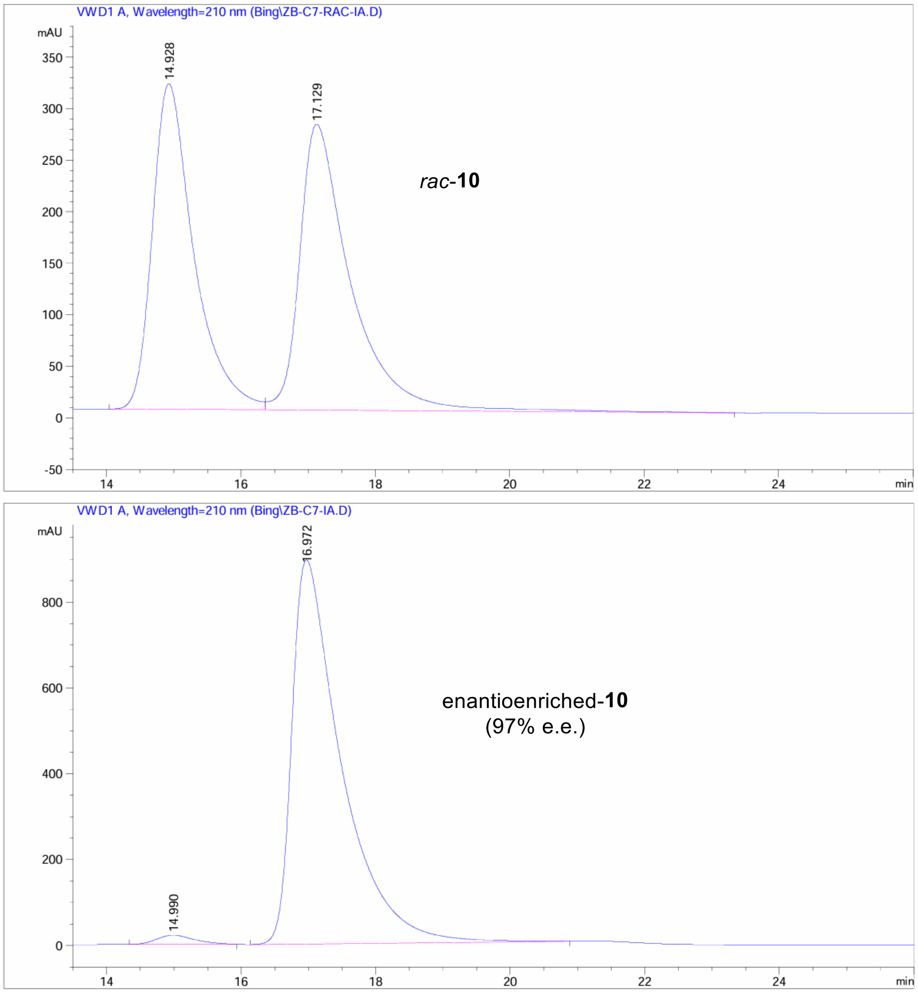

Peak #	RetTime [min]	Type	Width [min]	Area [mAU*s]	Height [mAU]	Area %
1	14.990	MM R	0.6517	835.01471	21.35357	1.7962
2	16.972	MF R	0.8503	4.56525e4	894.82214	98.2038

Figure S21. HPLC traces of *rac*-**10** and enantioenriched-**10**.

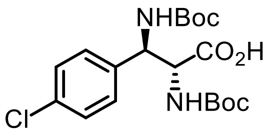

[CHIRALPAK IA, 25 ºC, *i*PrOH/*n*-hexane = 10/90 (v/v) + 0.1% TFA, 1.0 mL/min, 210 nm]

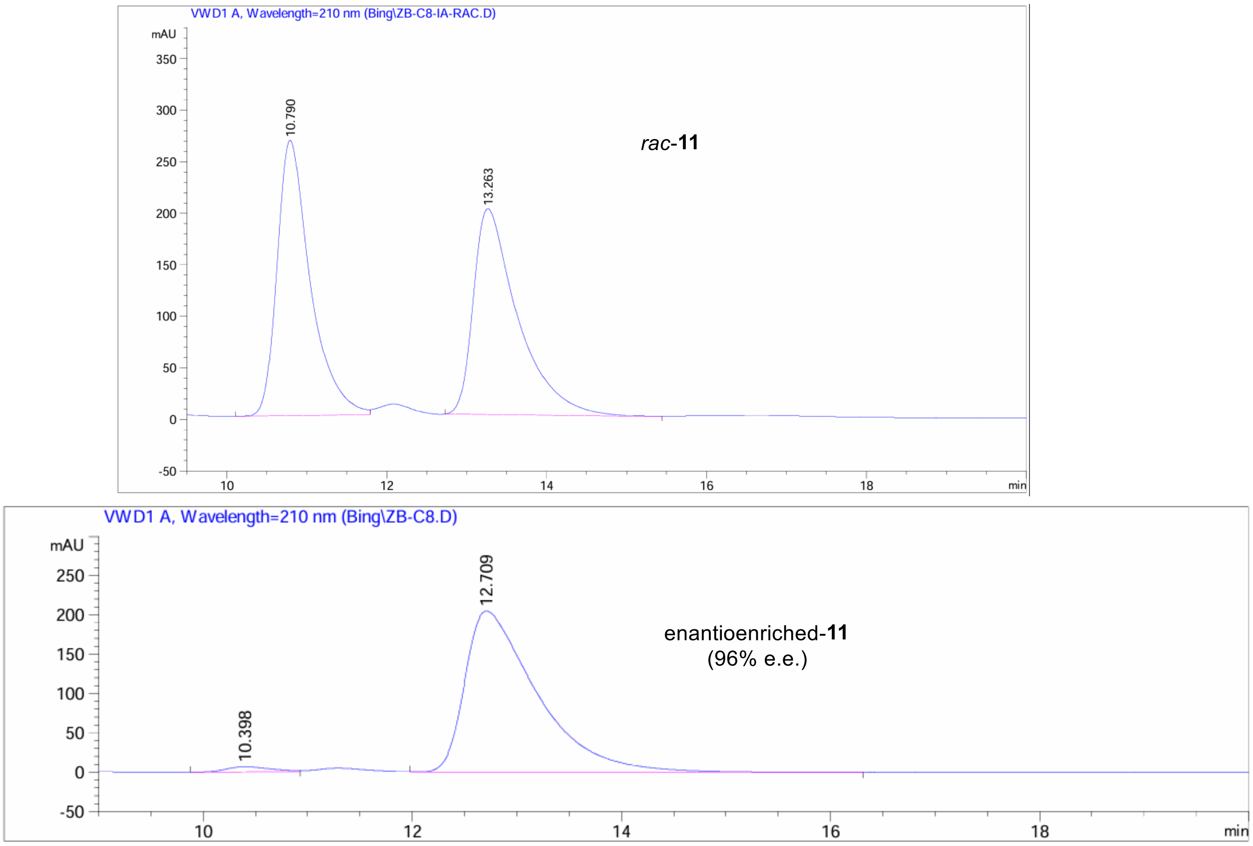

Peak #	RetTime [min]	Type	Width [min]	Area [mAU*s]	Height [mAU]	Area %
1	10.398	MV R	0.5056	203.94728	6.72292	2.0188
2	12.709	FM R	0.8036	9898.61426	205.30125	97.9812

Figure S22. HPLC traces of *rac*-**11** and enantioenriched-**11**.

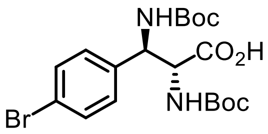

[CHIRALPAK IA, 25 ºC, *i*PrOH/*n*-hexane = 10/90 (v/v) + 0.1% TFA, 1.0 mL/min, 210 nm]

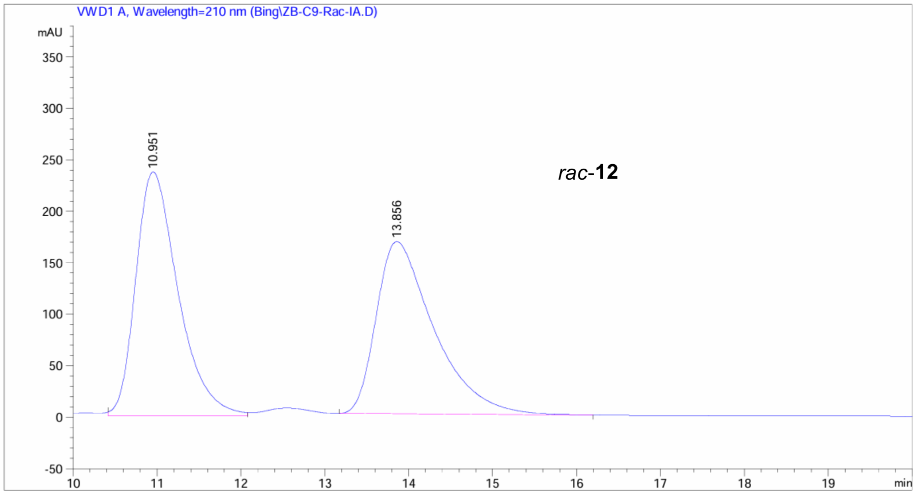

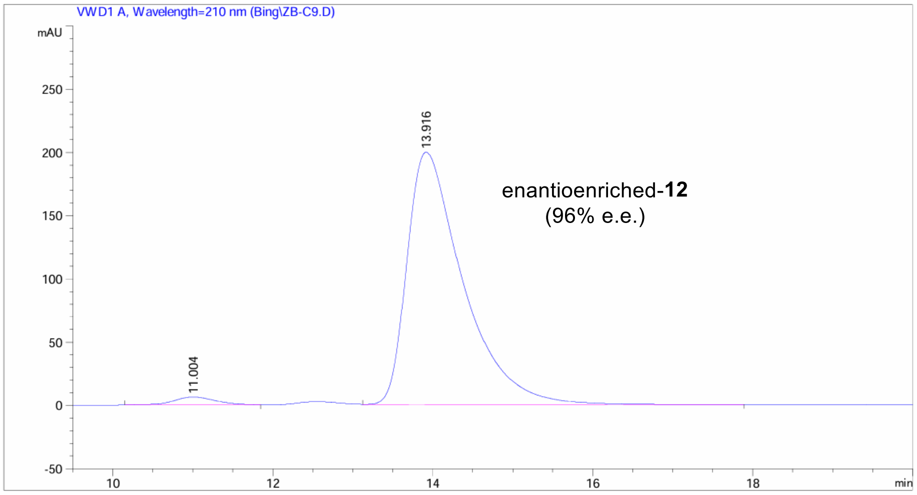

Peak #	RetTime [min]	Type	Width [min]	Area [mAU*s]	Height [mAU]	Area %
1	11.004	MM R	0.6080	223.60452	6.12980	2.2395
2	13.916	FM R	0.8142	9760.76465	199.79088	97.7605

Figure S23. HPLC traces of *rac*-**12** and enantioenriched-**12**.

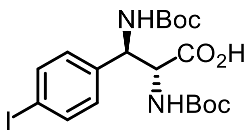

[CHIRALPAK IA, 25 ºC, *i*PrOH/*n*-hexane = 10/90 (v/v) + 0.1% TFA, 1.0 mL/min, 210 nm]

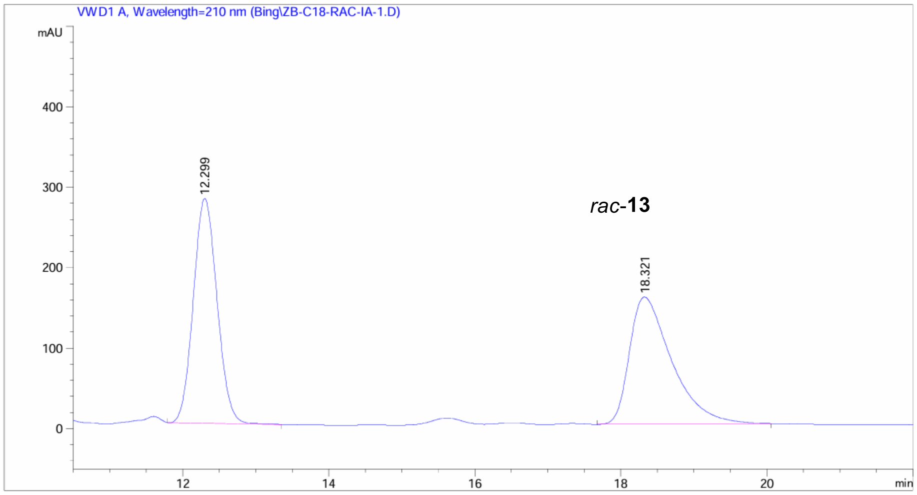

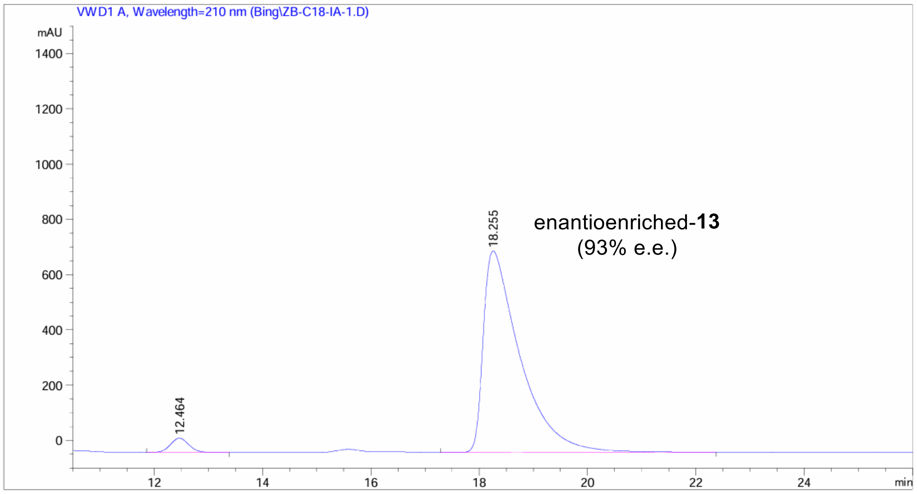

Peak #	RetTime [min]	Type	Width [min]	Area [mAU*s]	Height [mAU]	Area %
1	12.464	MM R	0.3958	1227.89893	51.70531	3.4336
2	18.255	MM R	0.7887	3.45332e4	729.71606	96.5664

Figure S24. HPLC traces of *rac*-**13** and enantioenriched-**13**.

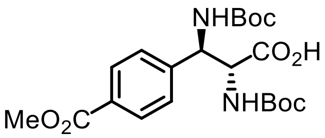

[CHIRALPAK IG, 25 ºC, *i*PrOH/*n*-hexane = 10/90 (v/v) + 0.1% TFA, 1.0 mL/min, 210 nm]

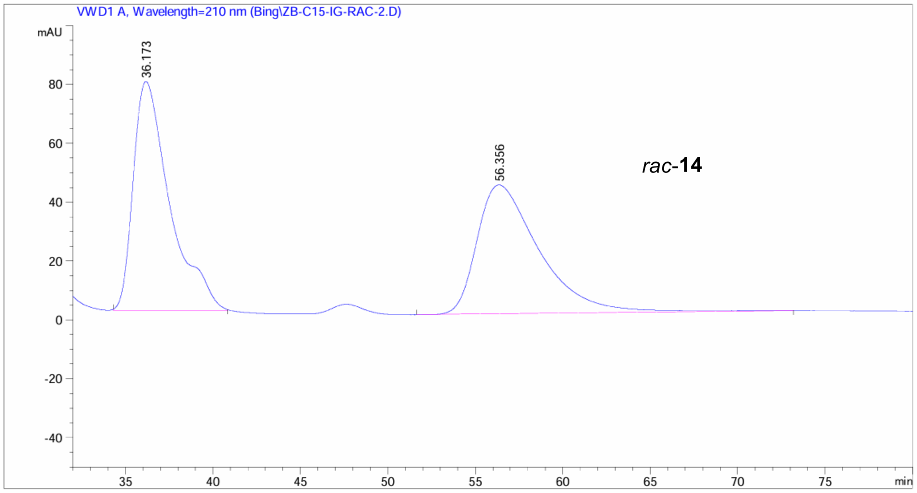

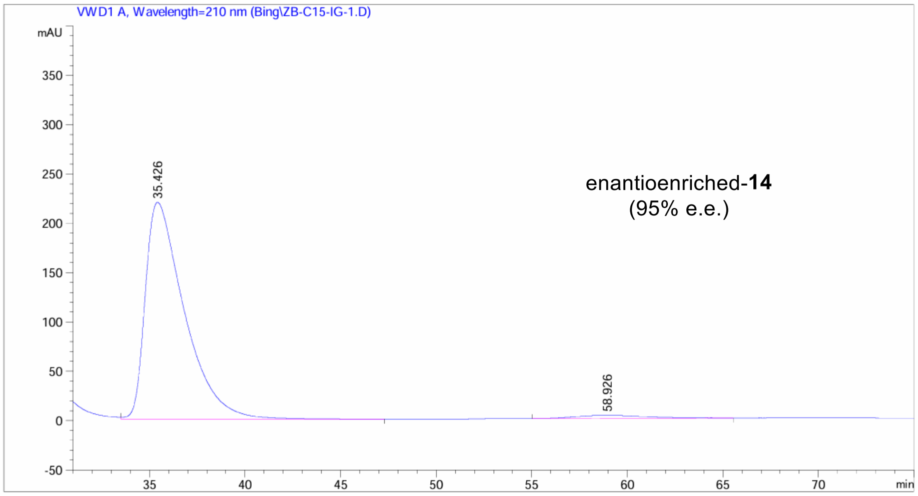

Peak #	RetTime [min]	Type	Width [min]	Area [mAU*s]	Height [mAU]	Area %
1	35.426	VB	2.0942	3.13859e4	220.15997	97.5519
2	58.926	BB	2.9830	787.64111	3.17981	2.4481

Figure S25. HPLC traces of *rac*-**14** and enantioenriched-**14**.

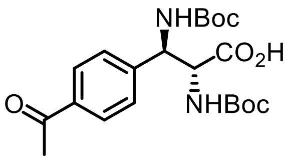

[CHIRALPAK IA, 25 ºC, *i*PrOH/*n*-hexane = 10/90 (v/v) + 0.1% TFA, 1.0 mL/min, 210 nm]

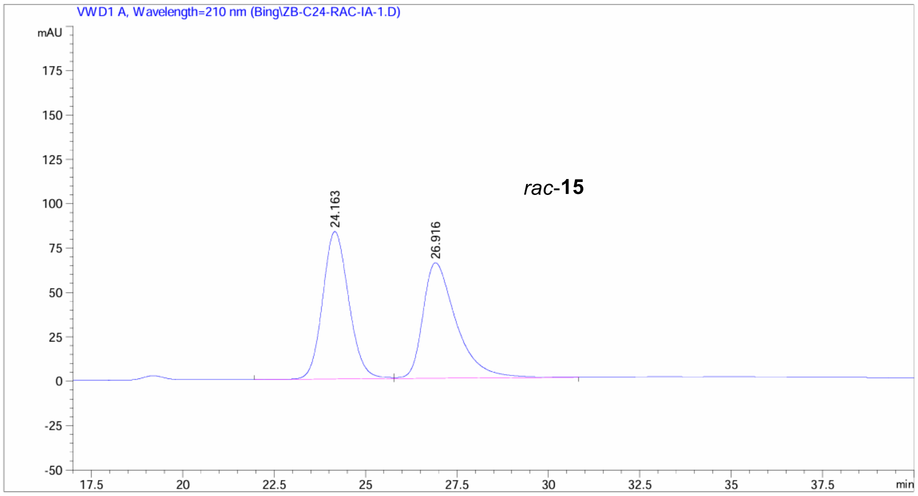

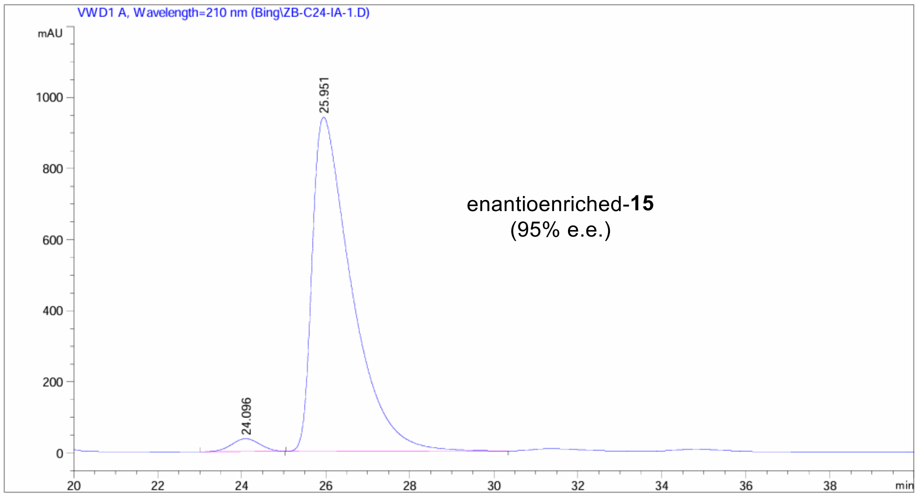

Peak #	RetTime [min]	Type	Width [min]	Area [mAU*s]	Height [mAU]	Area %
1	24.096	MM R	0.7820	1700.08582	36.23463	2.7080
2	25.951	MM R	1.0823	6.10806e4	940.56567	97.2920

Figure S26. HPLC traces of *rac*-**15** and enantioenriched-**15**.

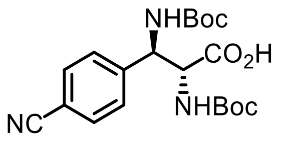

[CHIRALPAK IA, 25 ºC, *i*PrOH/*n*-hexane = 10/90 (v/v) + 0.1% TFA, 1.0 mL/min, 210 nm]

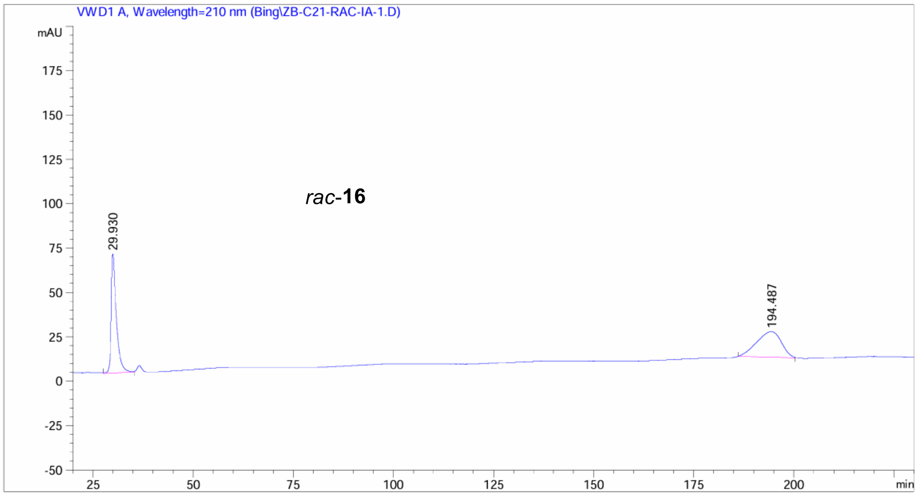

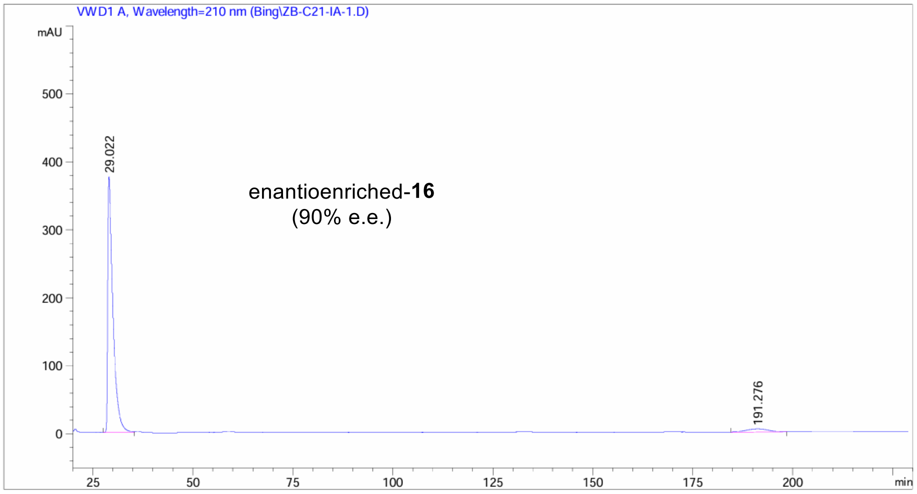

Peak #	RetTime [min]	Type	Width [min]	Area [mAU*s]	Height [mAU]	Area %
1	29.022	MF R	1.5063	3.39993e4	376.19812	95.0371
2	191.276	MM R	6.5579	1775.46545	4.51229	4.9629

Figure S27. HPLC traces of *rac*-**16** and enantioenriched-**16**.

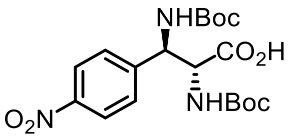

[CHIRALPAK IA, 25 ºC, *i*PrOH/*n*-hexane = 10/90 (v/v) + 0.1% TFA, 1.0 mL/min, 210 nm]

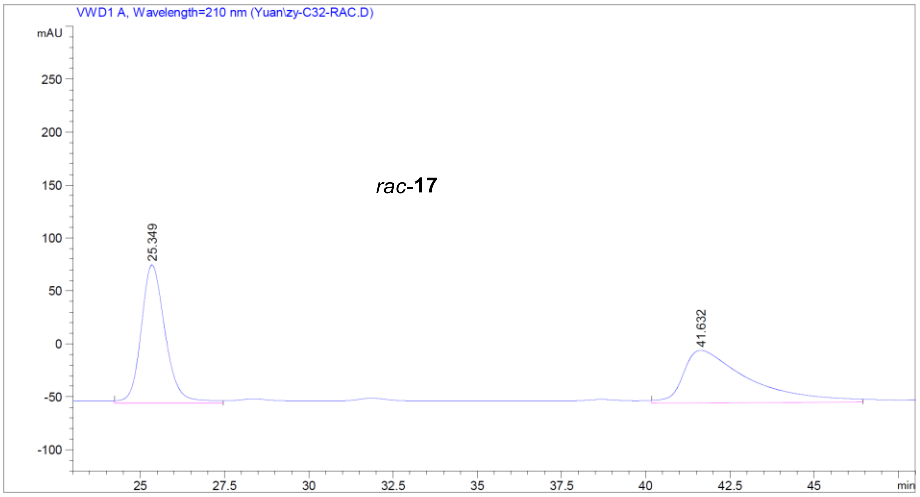

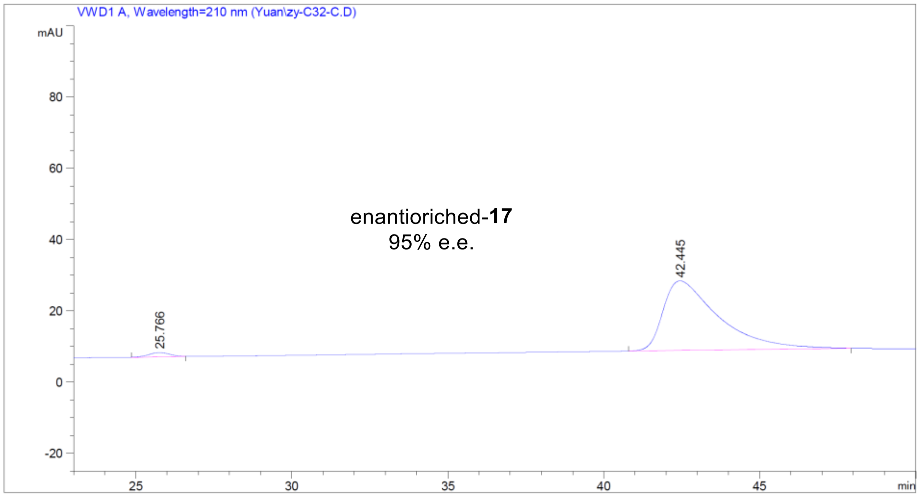

Peak #	RetTime [min]	Type	Width [min]	Area [mAU*s]	Height [mAU]	Area %
1	25.766	MM R	0.7814	56.93713	1.21442	2.3052
2	42.445	BB	1.6737	2413.01782	19.52006	97.6948

Figure S28. HPLC traces of *rac*-**17** and enantioenriched-**17**.

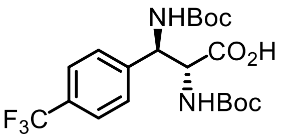

[CHIRALPAK IA, 25 ºC, *i*PrOH/*n*-hexane = 10/90 (v/v) + 0.1% TFA, 1.0 mL/min, 210 nm]

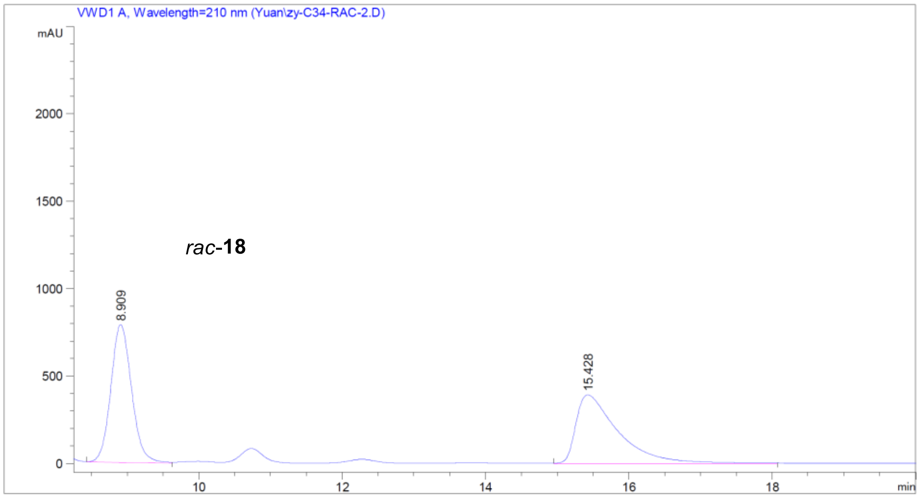

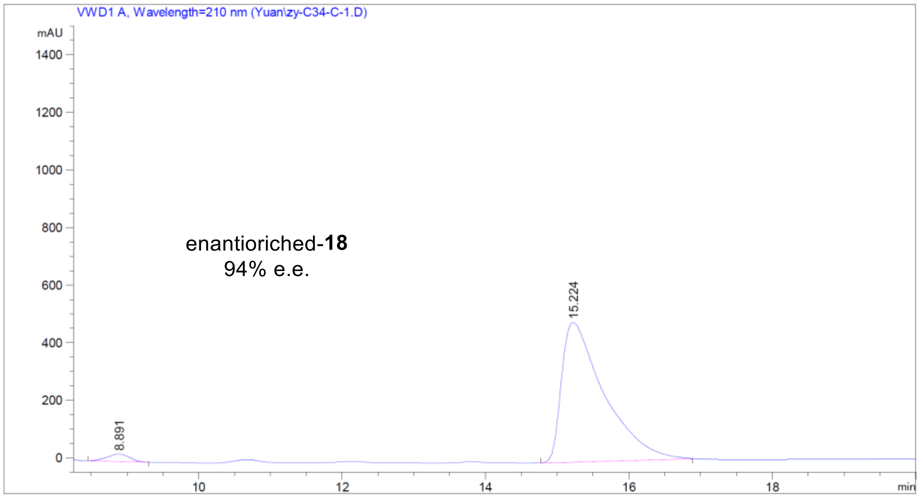

Peak #	RetTime [min]	Type	Width [min]	Area [mAU*s]	Height [mAU]	Area %
1	8.891	MM R	0.3654	560.36255	25.55763	2.7219
2	15.224	MM R	0.6879	2.00266e4	485.18304	97.2781

Figure S29. HPLC traces of *rac*-**18** and enantioenriched-**18**.

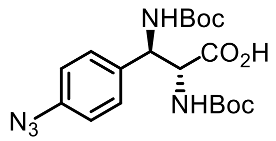

[CHIRALPAK IA, 40 ºC, *i*PrOH/*n*-hexane = 10/90 (v/v) + 0.1% TFA, 1.0 mL/min, 210 nm]

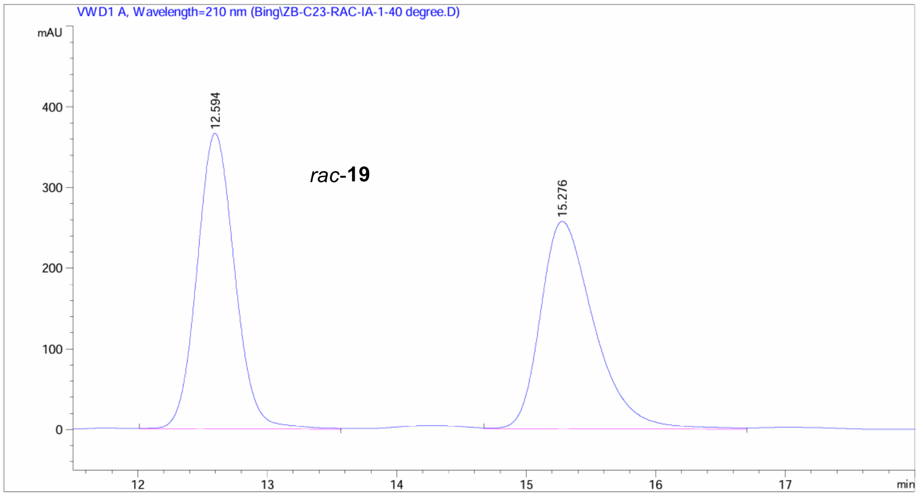

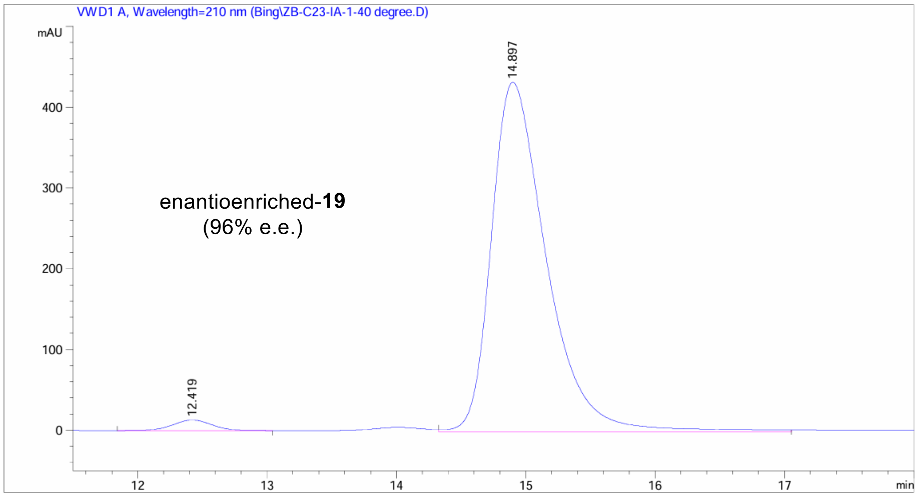

Peak #	RetTime [min]	Type	Width [min]	Area [mAU*s]	Height [mAU]	Area %
1	12.419	MM R	0.3572	280.74579	13.10075	2.1319
2	14.897	MM R	0.4964	1.28880e4	432.75171	97.8681

Figure S30. HPLC traces of *rac*-**19** and enantioenriched-**19**.

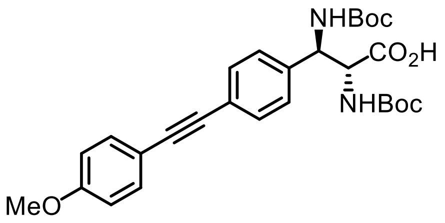

[CHIRALPAK IG, 25 ºC, *i*PrOH/*n*-hexane = 10/90 (v/v) + 0.1% TFA, 1.0 mL/min, 210 nm]

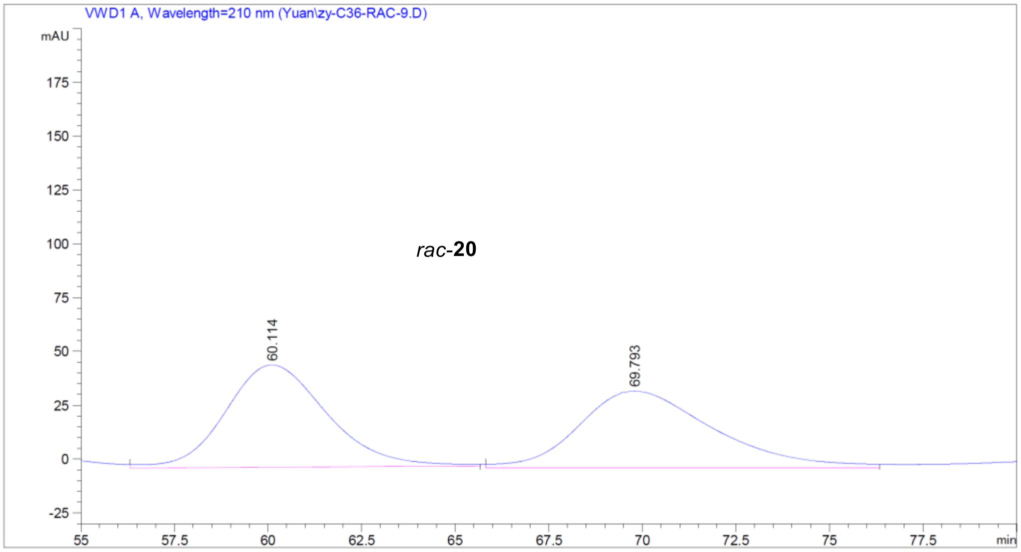

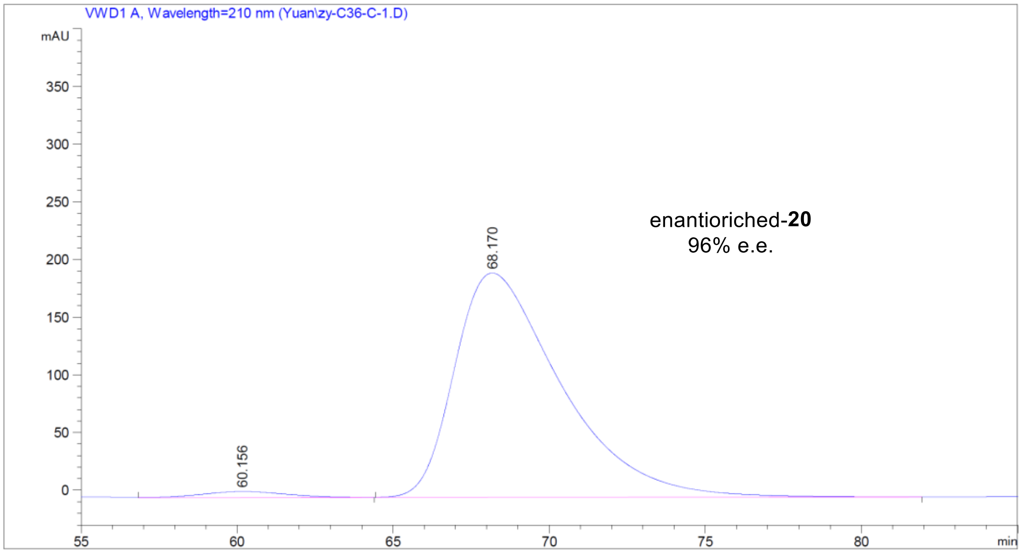

Peak #	RetTime [min]	Type	Width [min]	Area [mAU*s]	Height [mAU]	Area %
1	60.156	MM R	3.1706	1012.06317	5.32005	2.1428
2	68.170	MM R	3.9631	4.62179e4	194.36729	97.8572

Figure S31. HPLC traces of *rac*-**20** and enantioenriched-**20**.

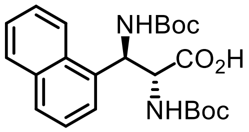

[CHIRALPAK IG, 25 ºC, *i*PrOH/*n*-hexane = 3/97 (v/v) + 0.1% TFA, 1.0 mL/min, 210 nm]

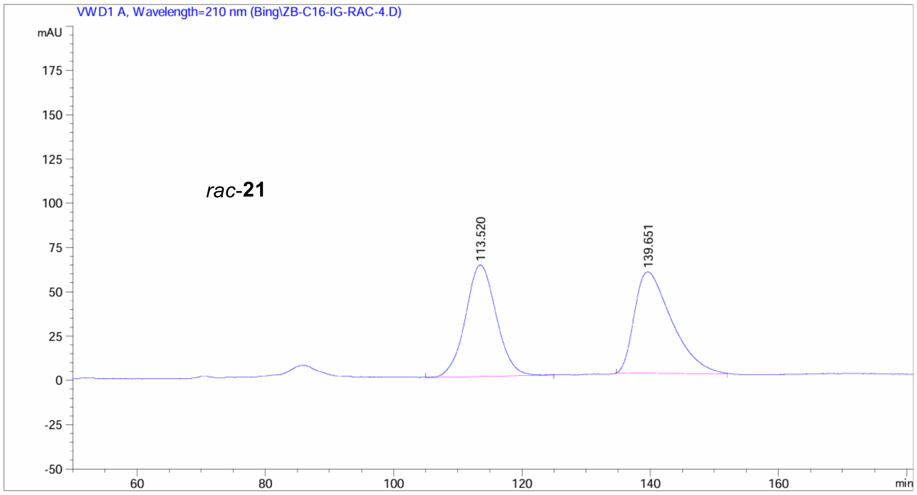

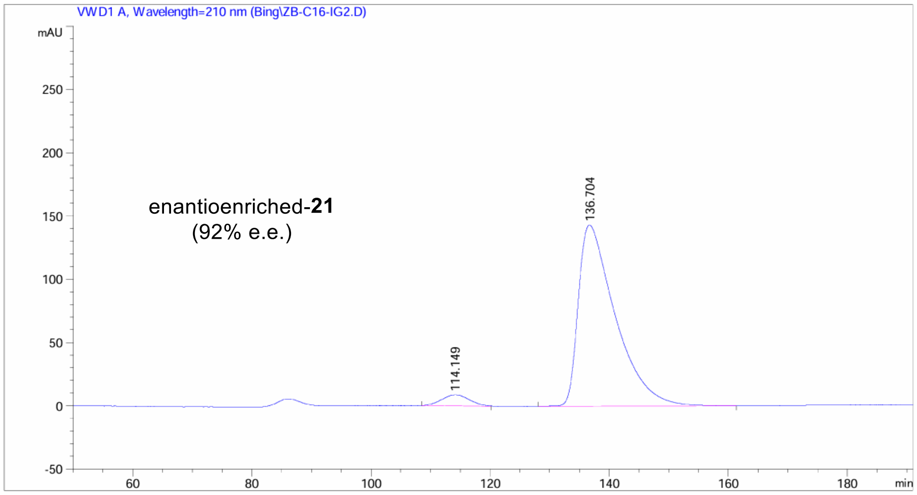

Peak #	RetTime [min]	Type	Width [min]	Area [mAU*s]	Height [mAU]	Area %
1	114.149	MM R	5.2840	2727.24805	8.60220	4.2254
2	136.704	MM R	7.1961	6.18176e4	143.17436	95.7746

Figure S32. HPLC traces of *rac*-**21** and enantioenriched-**21**.

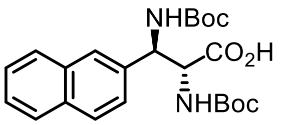

[CHIRALPAK IG, 25 ºC, *i*PrOH/*n*-hexane = 10/90 (v/v) + 0.1% TFA, 0.8 mL/min, 210 nm]

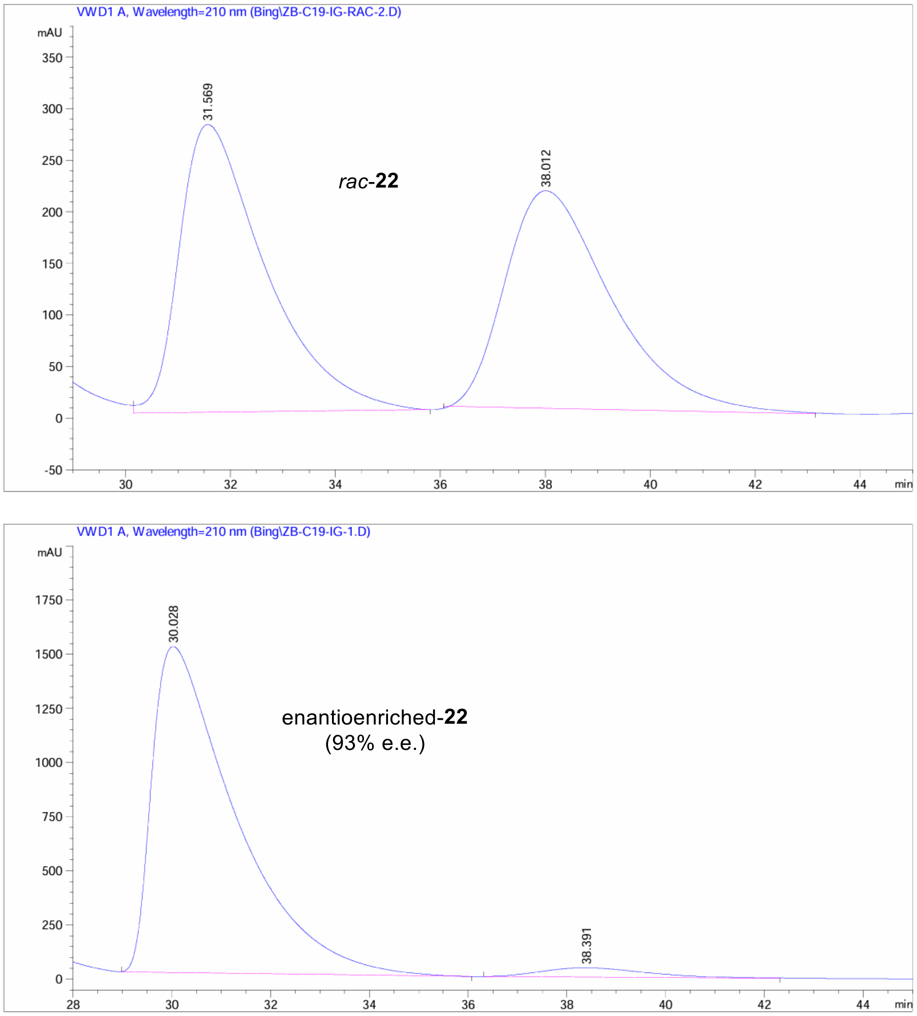

Peak #	RetTime [min]	Type	Width [min]	Area [mAU*s]	Height [mAU]	Area %
1	30.028	MM R	1.9496	1.76084e5	1505.28723	96.5929
2	38.391	MM R	2.4386	6211.01855	42.44874	3.4071

Figure S33. HPLC traces of *rac*-**22** and enantioenriched-**22**.

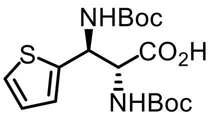

[CHIRALPAK IA, 25 ºC, *i*PrOH/*n*-hexane = 10/90 (v/v) + 0.1% TFA, 1.0 mL/min, 210 nm]

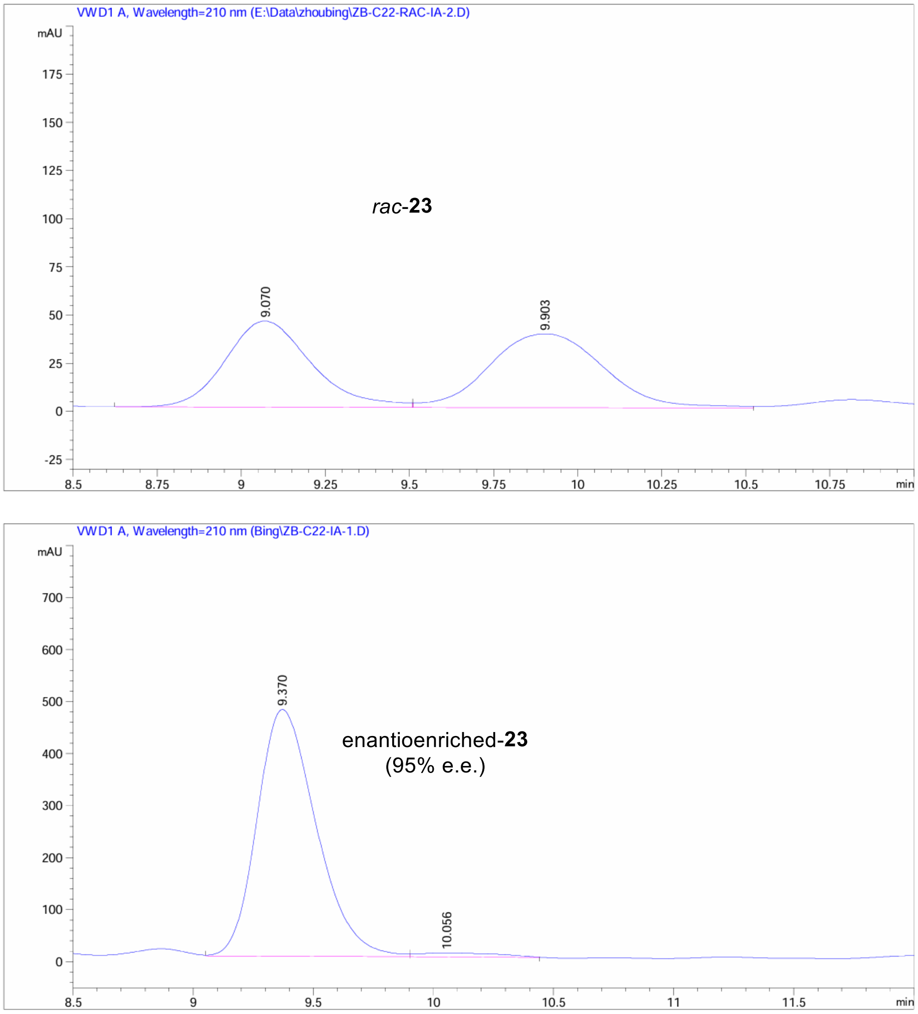

Peak #	RetTime [min]	Type	Width [min]	Area [mAU*s]	Height [mAU]	Area %
1	9.370	MF R	0.2851	8114.33643	474.39420	97.6880
2	10.056	FM R	0.3838	192.04695	8.33959	2.3120

Figure S34. HPLC traces of *rac*-**23** and enantioenriched-**23**.

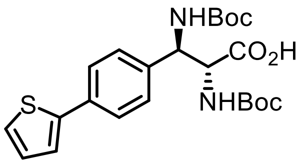

[CHIRALPAK IA, 25 ºC, *i*PrOH/*n*-hexane = 10/90 (v/v) + 0.1% TFA, 1.0 mL/min, 210 nm]

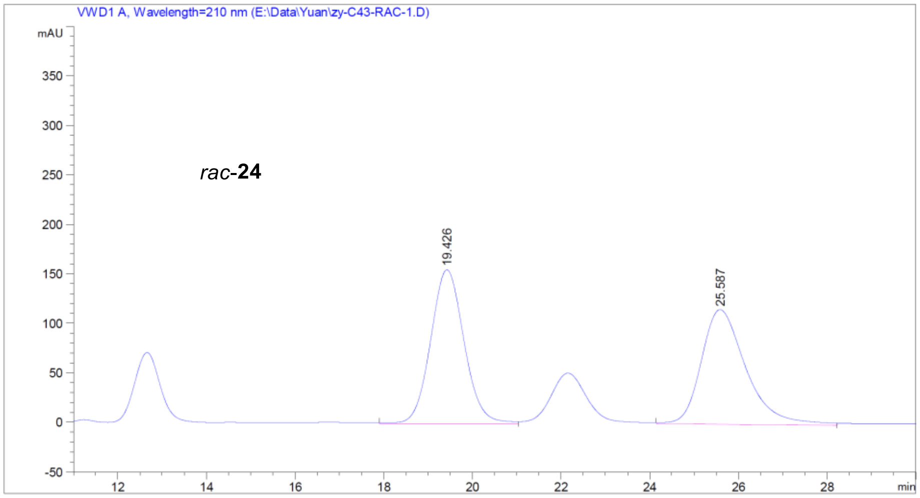

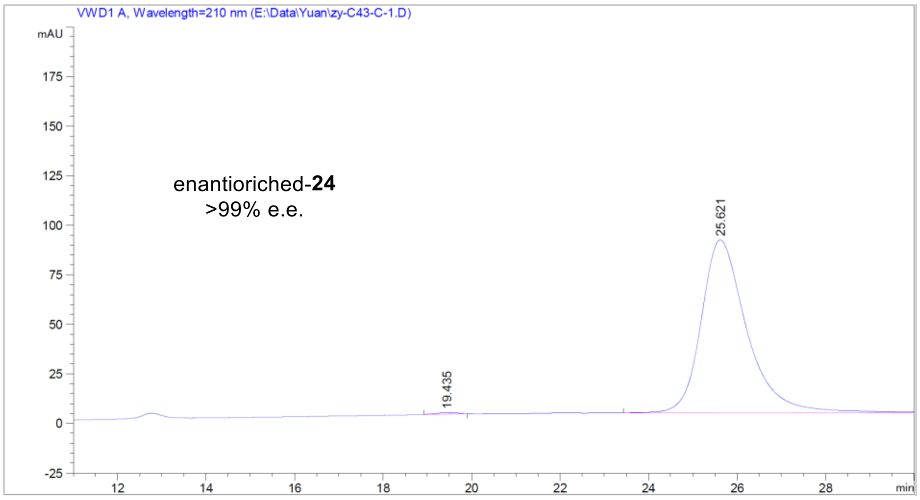

Peak #	RetTime [min]	Type	Width [min]	Area [mAU*s]	Height [mAU]	Area %
1	19.435	MM R	0.5911	25.99729	7.33022e-1	0.4139
2	25.621	BB	1.0769	6255.47314	87.20640	99.5861

Figure S35. HPLC traces of *rac*-**24** and enantioenriched-**24**.

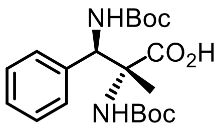

[CHIRALPAK OD-H, 25 ºC, *i*PrOH/*n*-hexane = 5/95 (v/v) + 0.1% TFA, 1.0 mL/min, 210 nm]

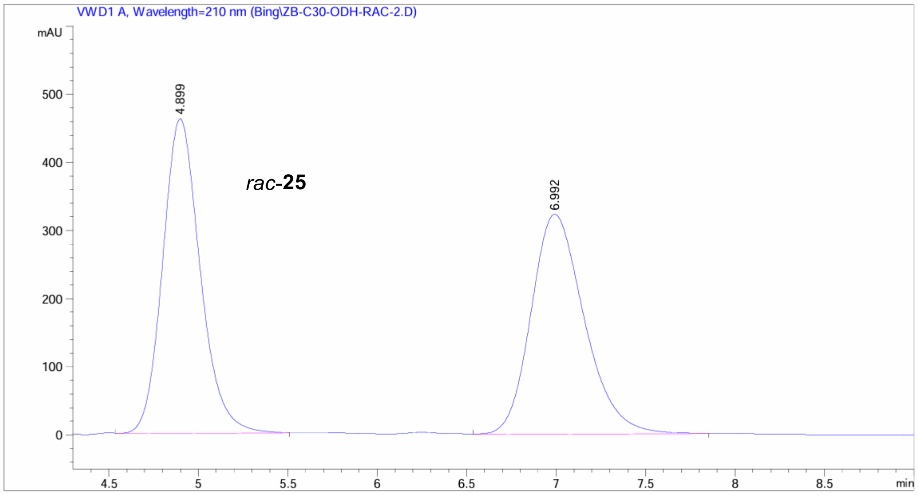

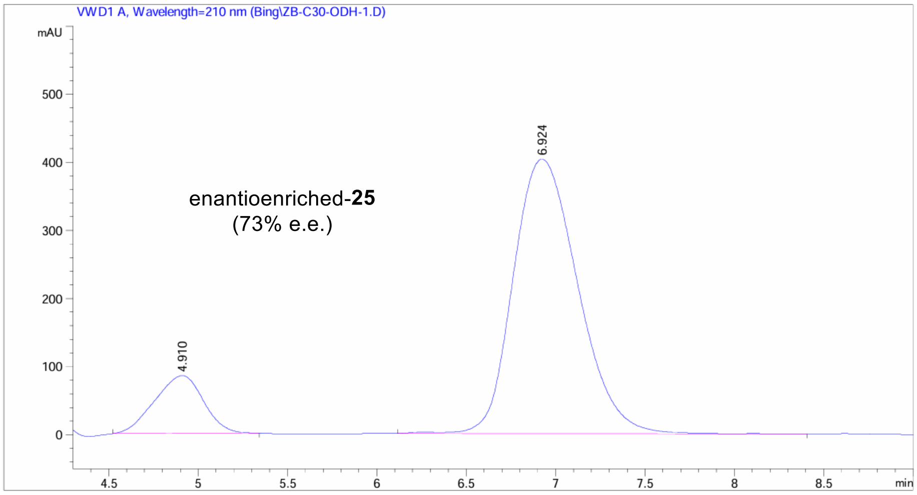

Peak #	RetTime [min]	Type	Width [min]	Area [mAU*s]	Height [mAU]	Area %
1	4.910	MM R	0.3158	1610.98999	85.03419	13.7270
2	6.924	MM R	0.4184	1.01249e4	403.34897	86.2730

Figure S36. HPLC traces of *rac*-**25** and enantioenriched-**25**.

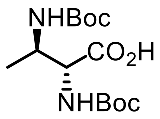

[CHIRALPAK AD-H, 25 ºC, *i*PrOH/*n*-hexane = 5/95 (v/v) + 0.1% TFA, 0.7 mL/min, 210 nm]

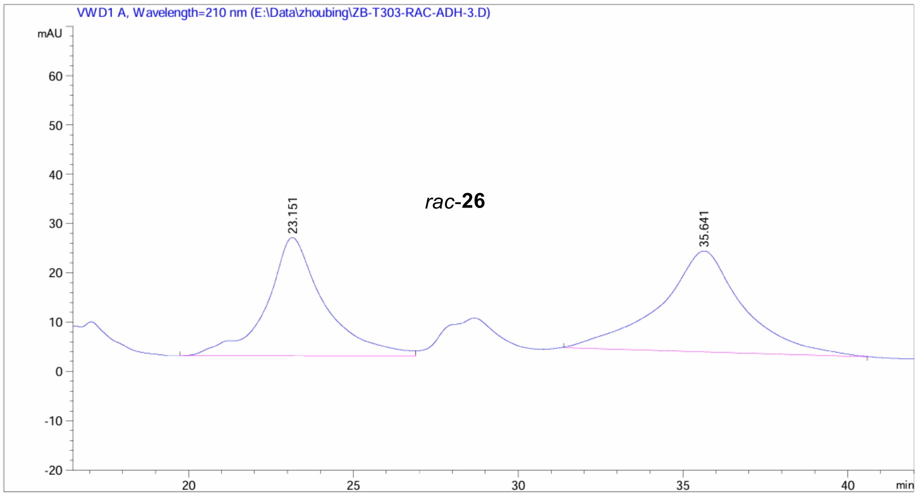

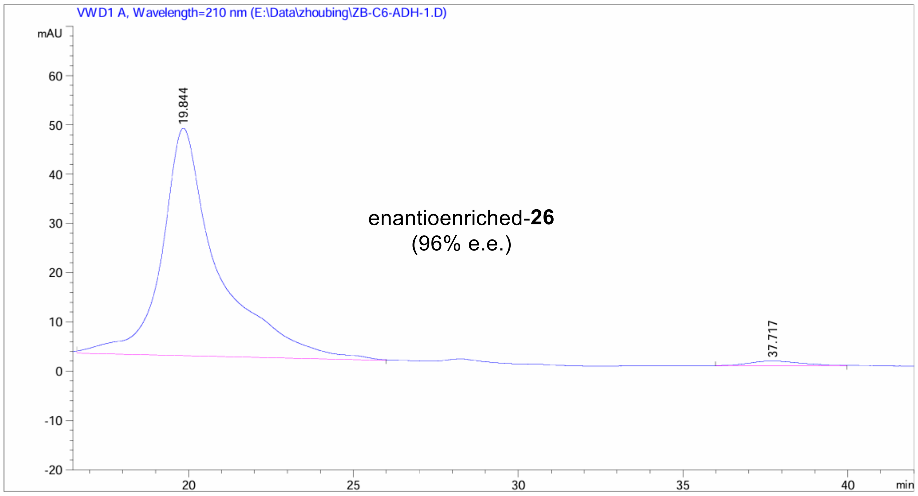

Peak #	RetTime [min]	Type	Width [min]	Area [mAU*s]	Height [mAU]	Area %
1	19.844	MM R	1.9886	5505.40039	46.14198	98.0886
2	37.717	MM R	1.8566	107.28082	9.63036e-1	1.9114

Figure S37. HPLC traces of *rac*-**26** and enantioenriched-**26**.

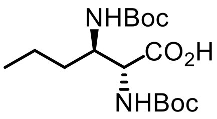

[CHIRALPAK AD-H, 25 ºC, *i*PrOH/*n*-hexane = 5/95 (v/v) + 0.1% TFA, 1.0 mL/min, 210 nm]

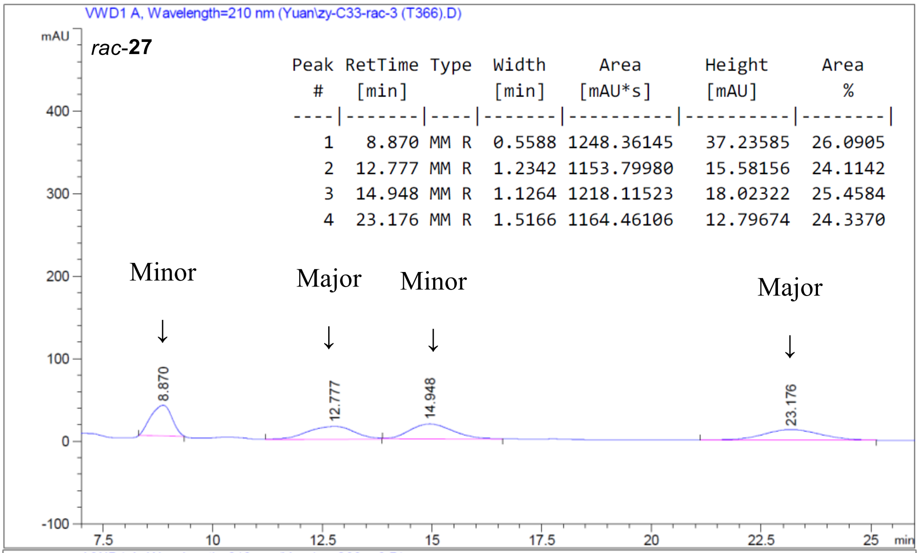

Peak #	RetTime [min]	Type	Width [min]	Area [mAU*s]	Height [mAU]	Area %
1	8.870	MM R	0.5588	1248.36145	37.23585	26.0905
2	12.777	MM R	1.2342	1153.79980	15.58156	24.1142
3	14.948	MM R	1.1264	1218.11523	18.02322	25.4584
4	23.176	MM R	1.5166	1164.46106	12.79674	24.3370

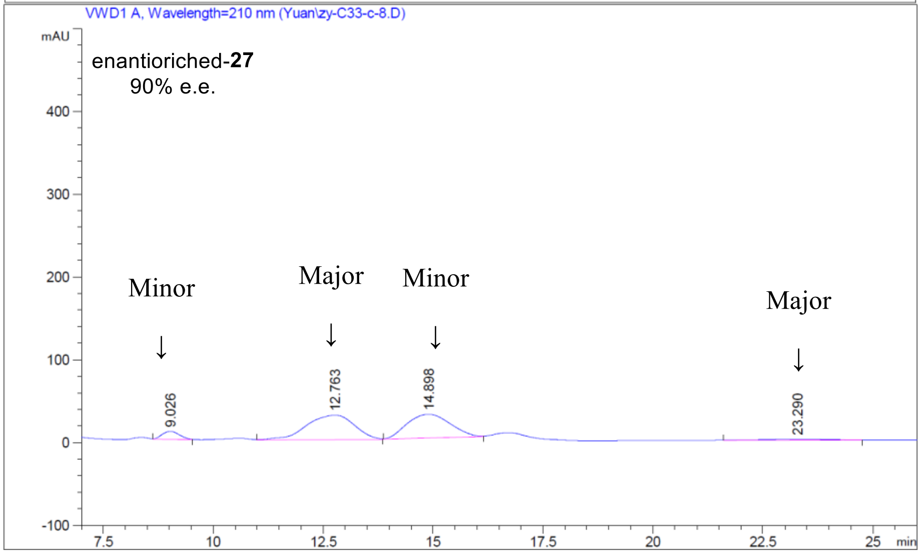

Peak #	RetTime [min]	Type	Width [min]	Area [mAU*s]	Height [mAU]	Area %
1	9.026	MM R	0.4551	261.96350	9.59347	5.6649
2	12.763	MM R	1.2652	2260.98926	29.78402	48.8929
3	14.898	MM R	1.1393	1978.99988	28.95132	42.7950
4	23.290	MM R	1.5136	122.41430	1.34794	2.6472

Figure S38. HPLC traces of *rac*-**27** and enantioenriched-**27**.

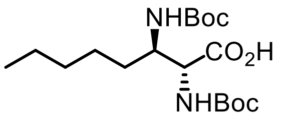

[CHIRALPAK AD-H, 25 ºC, *i*PrOH/*n*-hexane = 5/95 (v/v) + 0.1% TFA, 1.0 mL/min, 210 nm]

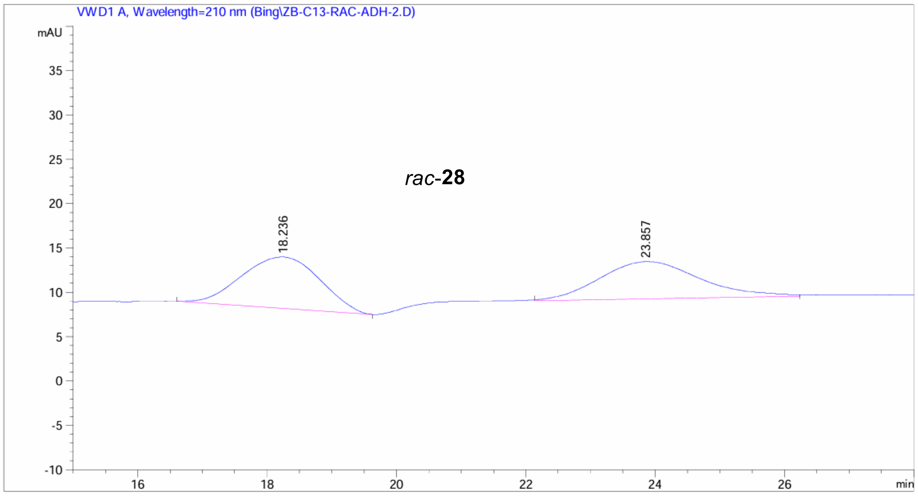

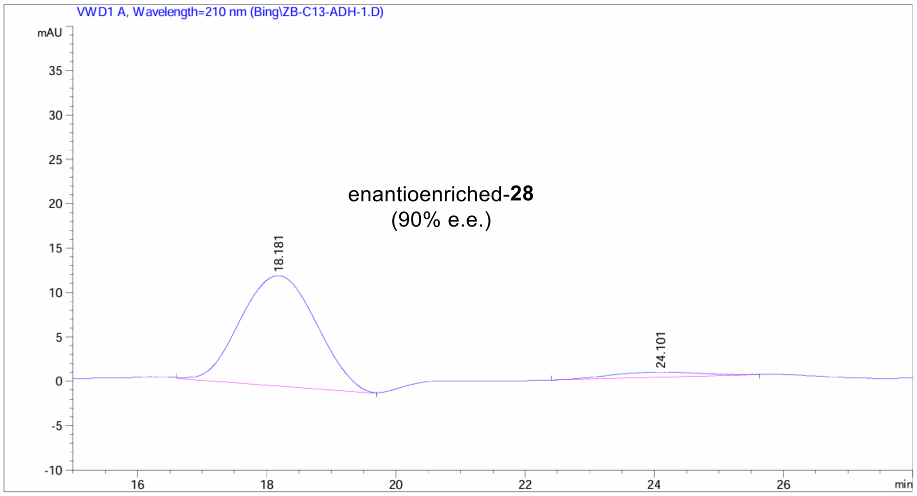

Peak #	RetTime [min]	Type	Width [min]	Area [mAU*s]	Height [mAU]	Area %
1	18.181	MM R	1.3610	1016.02631	12.44208	94.8860
2	24.101	MM R	1.5844	54.76034	5.76025e-1	5.1140

Figure S39. HPLC traces of *rac*-**28** and enantioenriched-**28**.

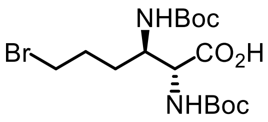

[CHIRALPAK AD-H, 25 ºC, *i*PrOH/*n*-hexane = 5/95 (v/v) + 0.1% TFA, 1.0 mL/min, 210 nm]

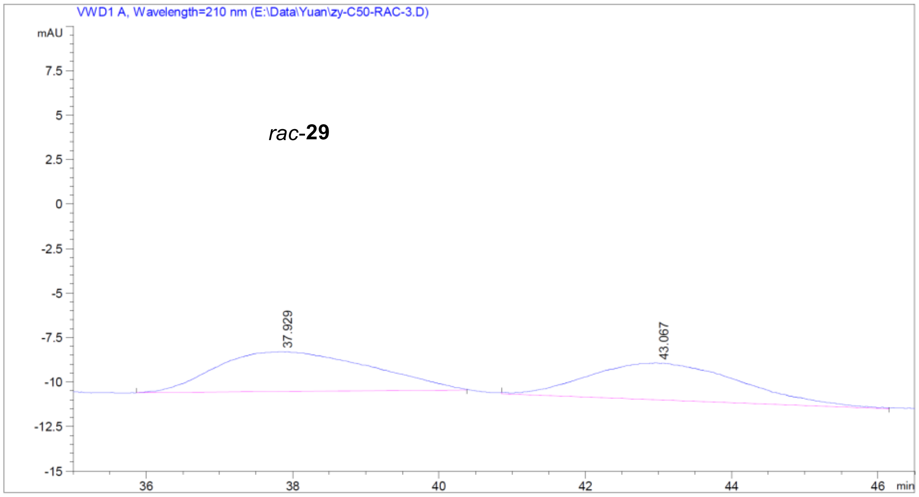

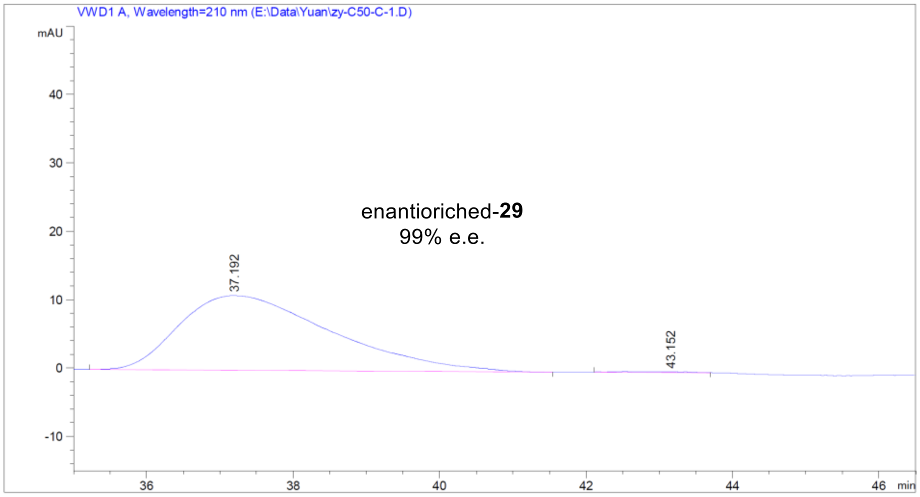

Peak #	RetTime [min]	Type	Width [min]	Area [mAU*s]	Height [mAU]	Area %
1	37.192	BB	1.7287	1580.39868	10.94415	99.4380
2	43.152	MM R	1.1316	8.93182	1.31546e-1	0.5620

Figure S40. HPLC traces of *rac*-**29** and enantioenriched-**29**.

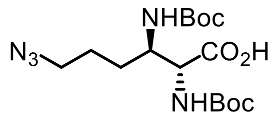

[CHIRALPAK AD-H, 25 ºC, *i*PrOH/*n*-hexane = 15/85 (v/v) + 0.1% TFA, 1.0 mL/min, 254 nm]

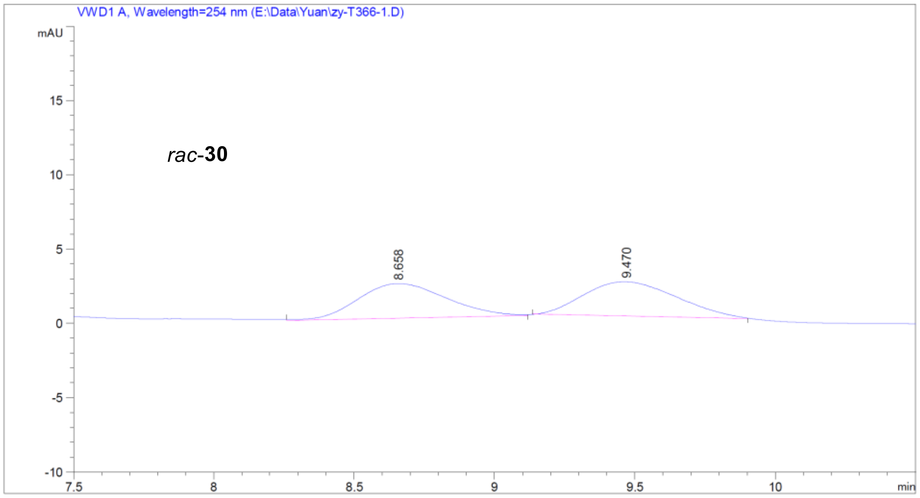

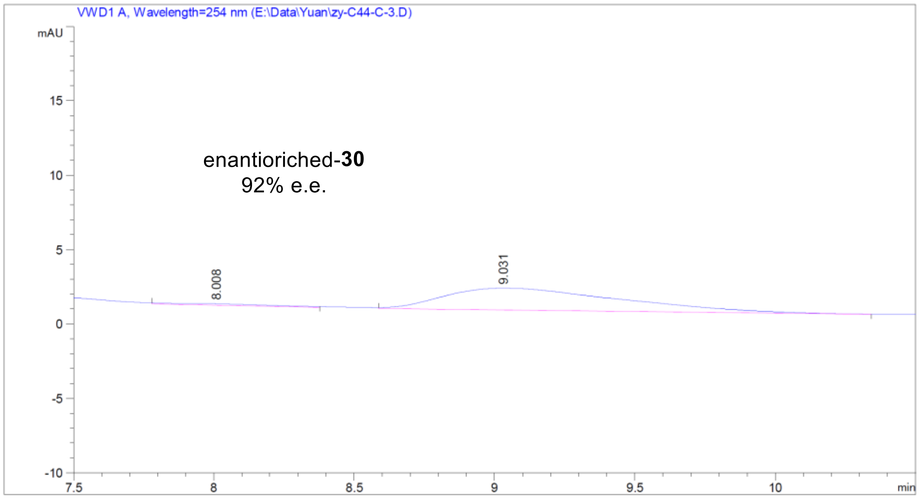

Peak #	RetTime [min]	Type	Width [min]	Area [mAU*s]	Height [mAU]	Area %
1	8.008	MM R	0.4351	2.56078	9.81004e-2	3.7946
2	9.031	VB	0.6404	64.92402	1.46672	96.2054

Figure S41. HPLC traces of *rac*-**30** and enantioenriched-**30**.

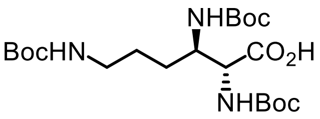

[CHIRALPAK IG, 25 ºC, *i*PrOH/*n*-hexane = 15/85 (v/v) + 0.1% TFA, 1.0 mL/min, 254 nm]

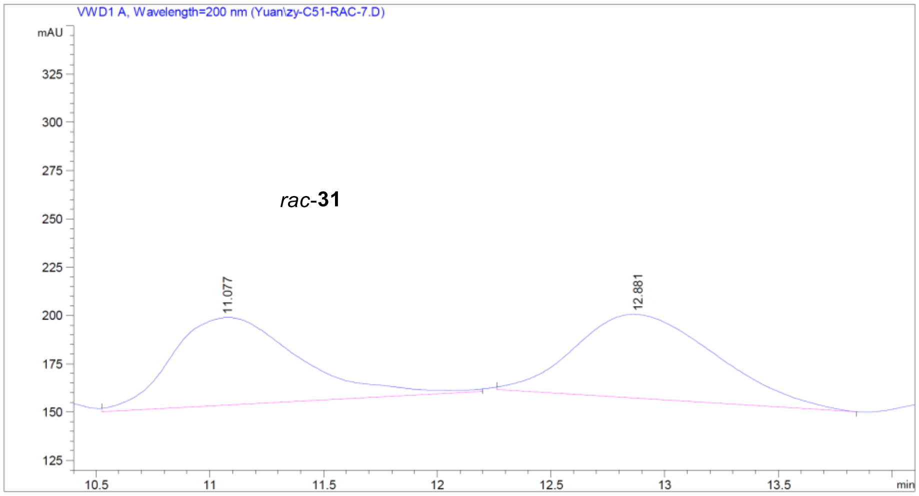

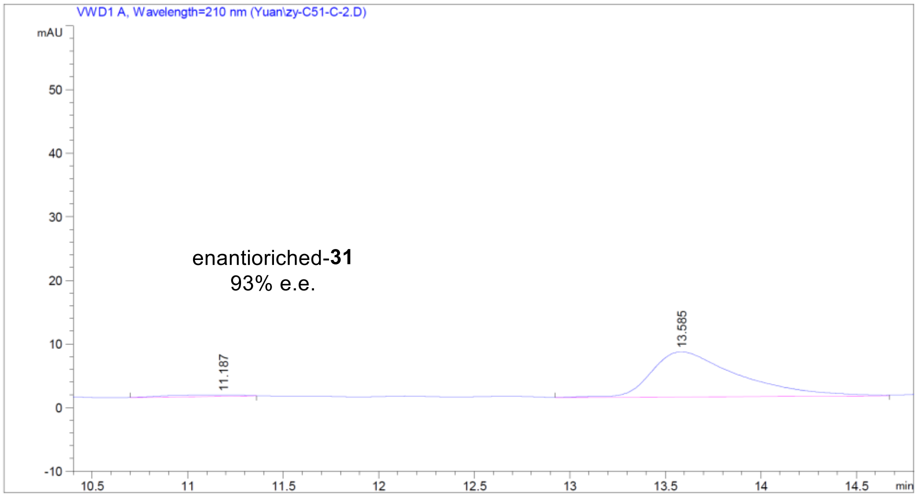

Peak #	RetTime [min]	Type	Width [min]	Area [mAU*s]	Height [mAU]	Area %
1	11.187	MM R	0.4095	7.79875	2.27973e-1	3.3186
2	13.585	MM R	0.5320	227.20378	7.11785	96.6814

Figure S42. HPLC traces of *rac*-**31** and enantioenriched-**31**.

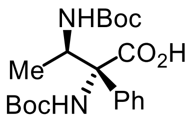

[CHIRALPAK IA, 25 ºC, *i*PrOH/*n*-hexane = 10/90 (v/v) + 0.1% TFA, 1.0 mL/min, 210 nm]

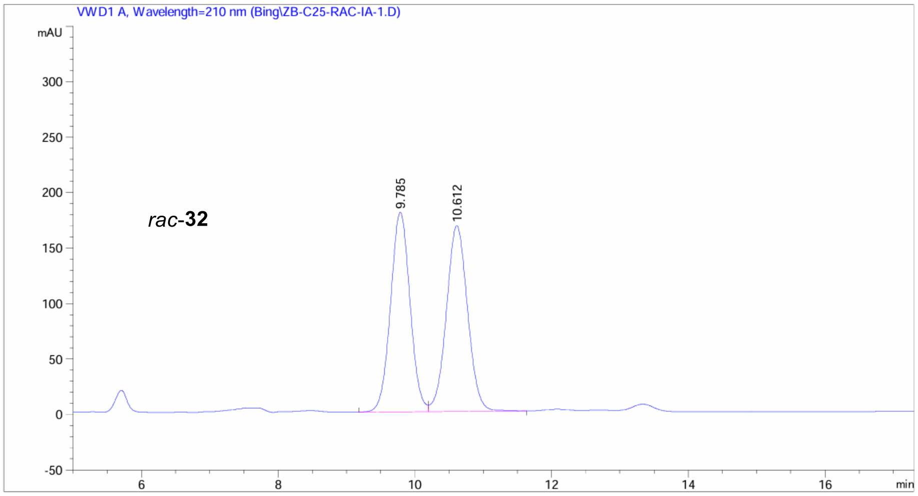

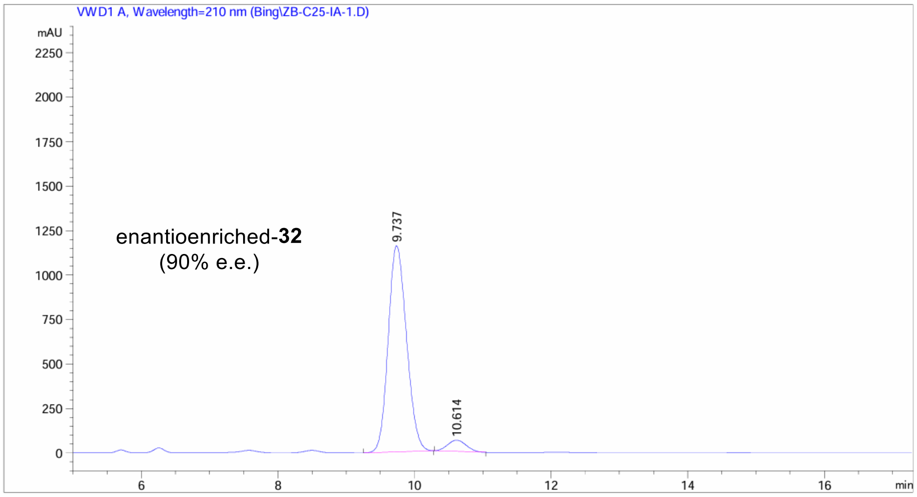

Peak #	RetTime [min]	Type	Width [min]	Area [mAU*s]	Height [mAU]	Area %
1	9.737	MM R	0.3106	2.16227e4	1160.09302	94.7610
2	10.614	MM R	0.3143	1195.43884	63.39920	5.2390

Figure S43. HPLC traces of *rac*-**32** and enantioenriched-**32**.

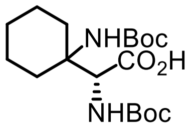

[CHIRALPAK IA, 25 ºC, *i*PrOH/*n*-hexane = 10/90 (v/v) + 0.1% TFA, 1.0 mL/min, 210 nm]

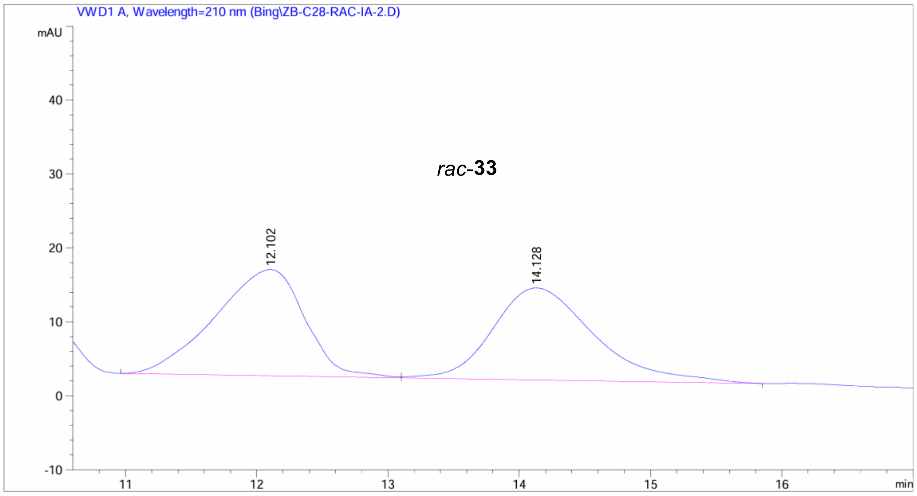

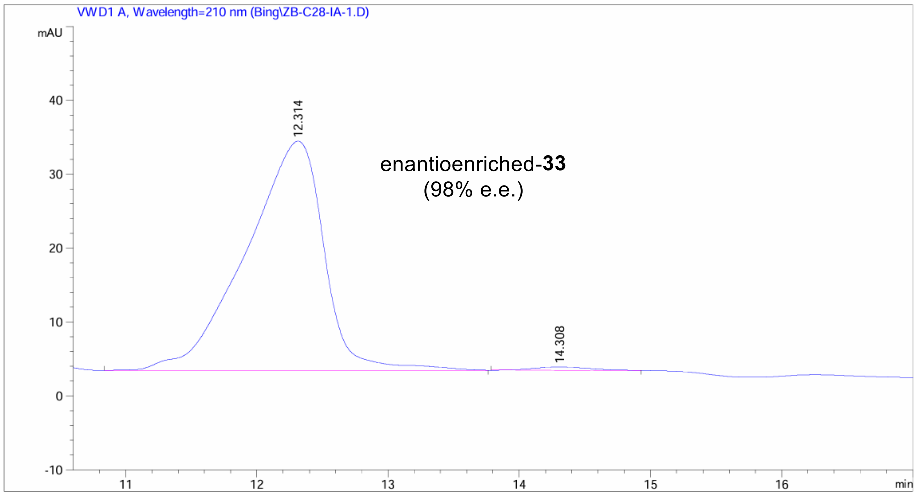

Peak #	RetTime [min]	Type	Width [min]	Area [mAU*s]	Height [mAU]	Area %
1	12.314	BB	0.6080	1279.11169	31.00404	98.9545
2	14.308	MM R	0.4893	13.51408	4.60337e-1	1.0455

Figure S44. HPLC traces of *rac*-**33** and enantioenriched-**33**.

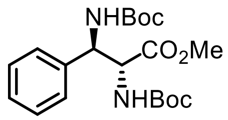

[CHIRALPAK IA, 25 ºC, *i*PrOH/*n*-hexane = 5/95 (v/v), 1.0 mL/min, 210 nm]

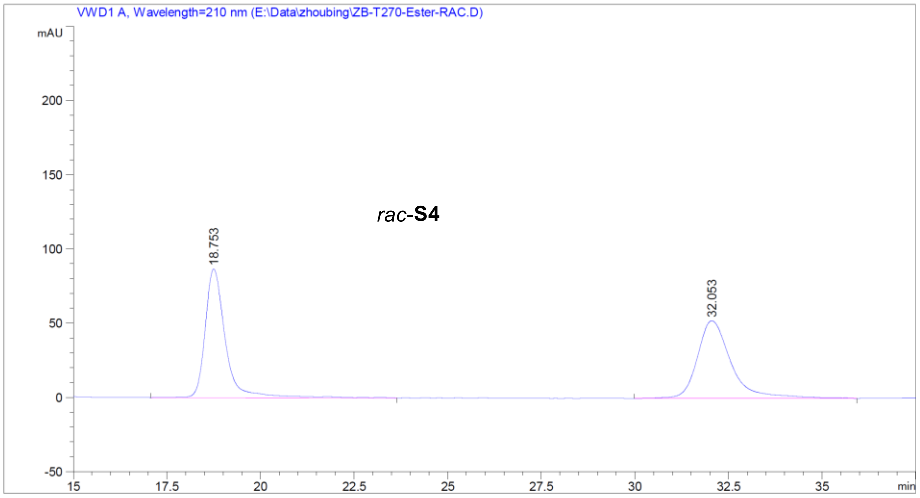

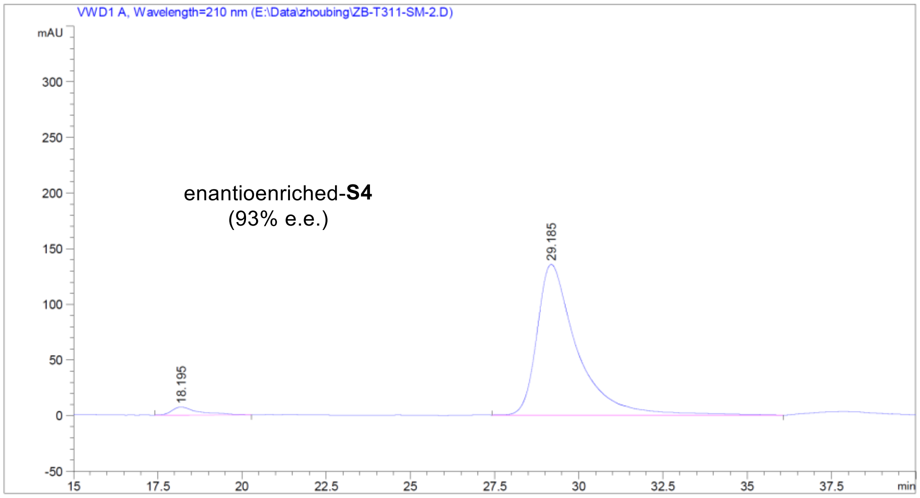

Peak #	RetTime [min]	Type	Width [min]	Area [mAU*s]	Height [mAU]	Area %
1	18.195	MM R	0.9414	406.00708	7.18796	3.4763
2	29.185	MM R	1.3873	1.12734e4	135.43781	96.5237

Figure S45. HPLC traces of *rac*-**S4** and enantioenriched-**S4**.

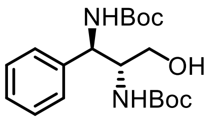

[CHIRALPAK IC, 25 ºC, *i*PrOH/*n*-hexane = 9/91 (v/v), 1.0 mL/min, 210 nm]

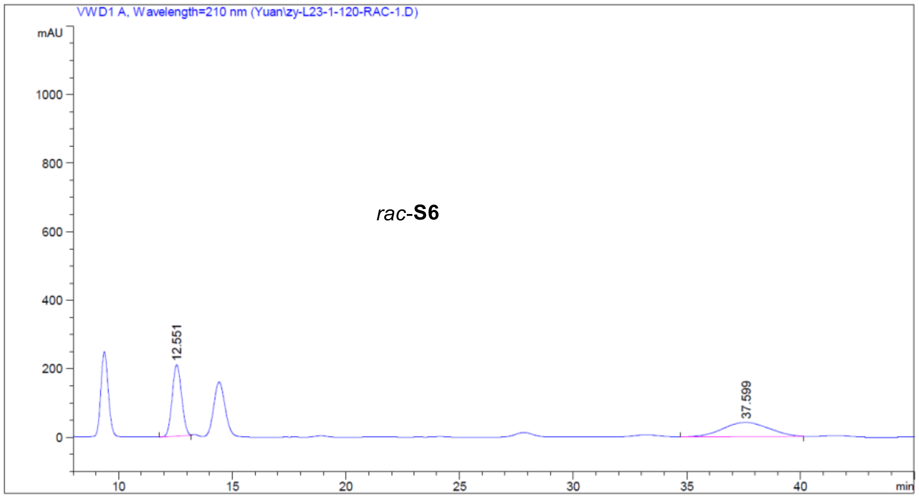

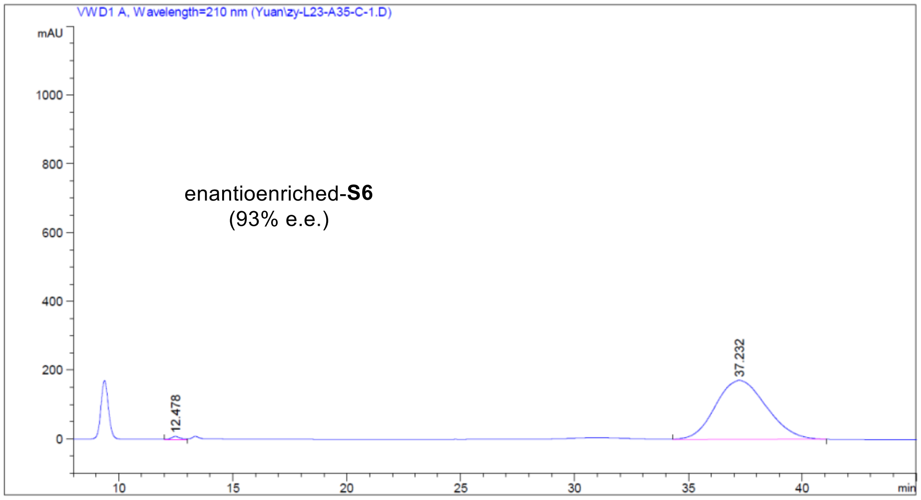

Peak #	RetTime [min]	Type	Width [min]	Area [mAU*s]	Height [mAU]	Area %
1	12.478	MM R	0.4704	254.10257	9.00276	0.9427
2	37.232	MM R	2.5865	2.67002e4	172.04834	99.0573

Figure S46. HPLC traces of *rac*-**S6** and enantioenriched-**S6**.

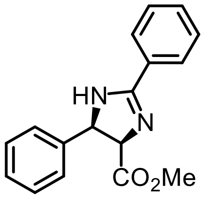

[CHIRALPAK IA, 25 ºC, *i*PrOH/*n*-hexane = 5/95 (v/v), 1.0 mL/min, 210 nm]

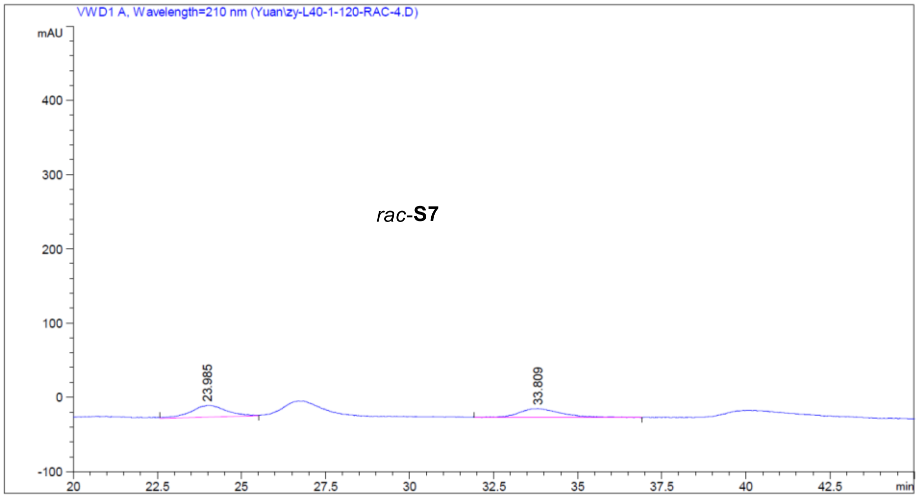

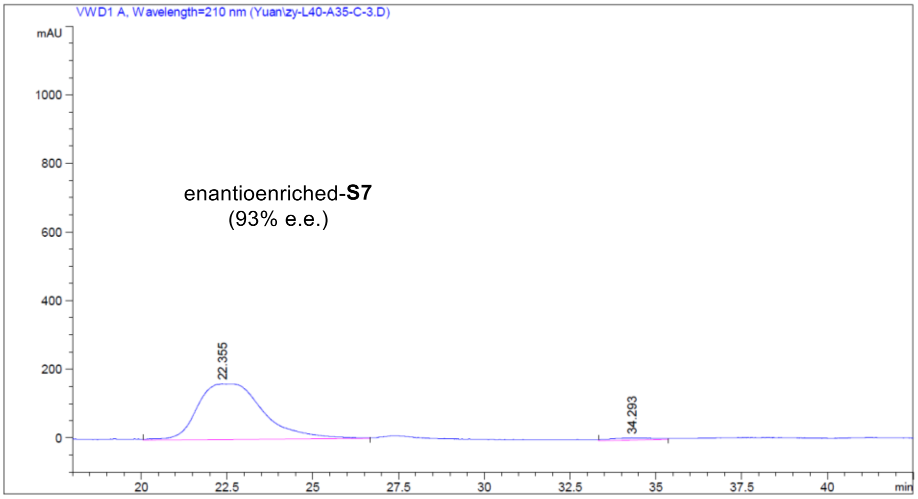

Peak #	RetTime [min]	Type	Width [min]	Area [mAU*s]	Height [mAU]	Area %
1	22.355	MM R	2.2301	2.17640e4	162.65076	97.6504
2	34.293	MM R	1.4136	523.68317	6.17418	2.3496

Figure S47. HPLC traces of *rac*-**S7** and enantioenriched-**S7**.

9. NMR Spectra

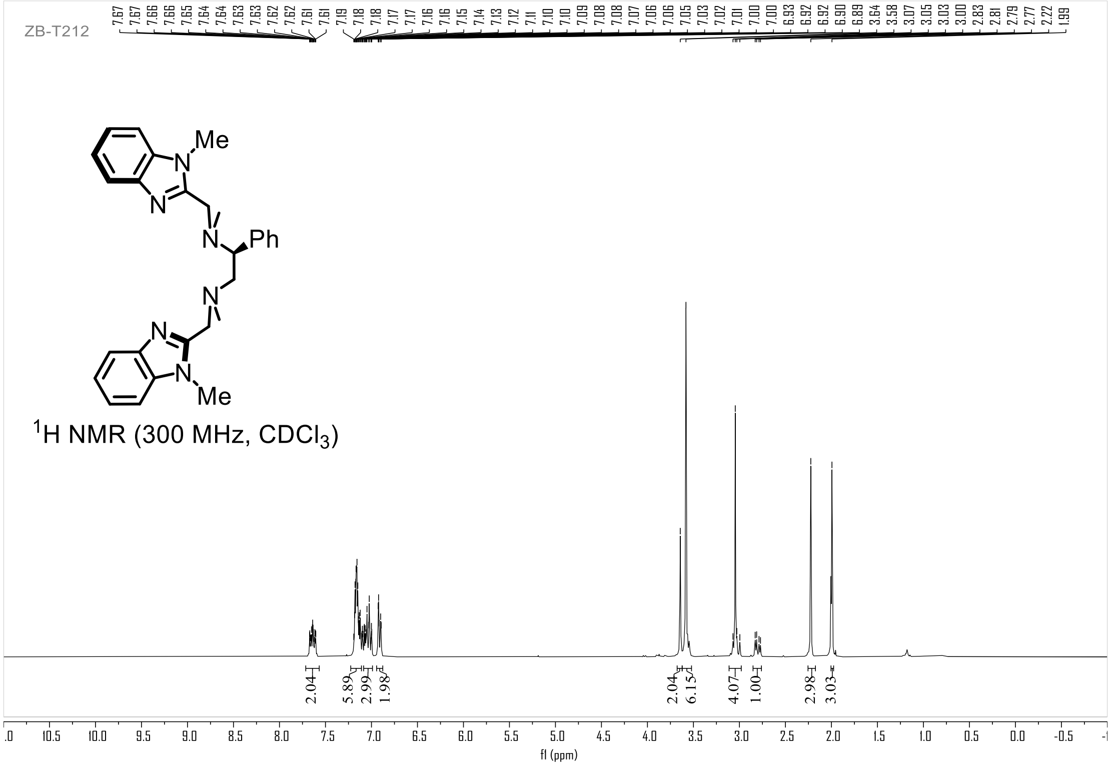

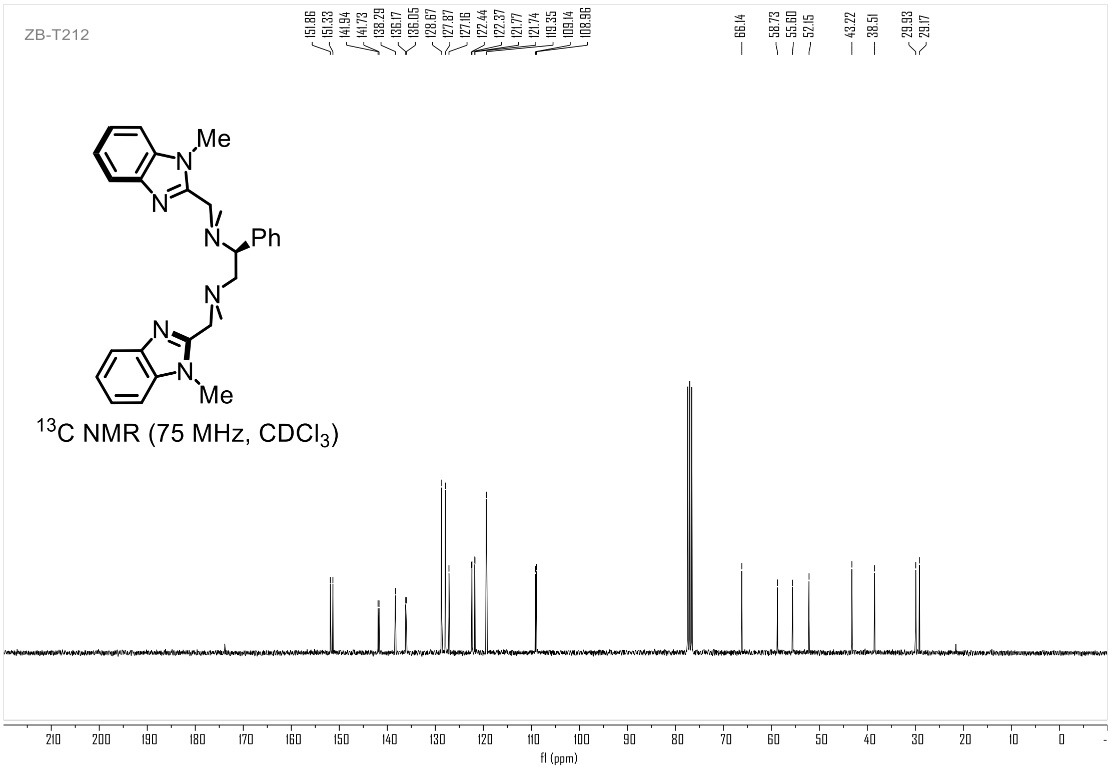

Figure S48. ^{1}H NMR, ^{13}C NMR spectra of **S2-Me**.

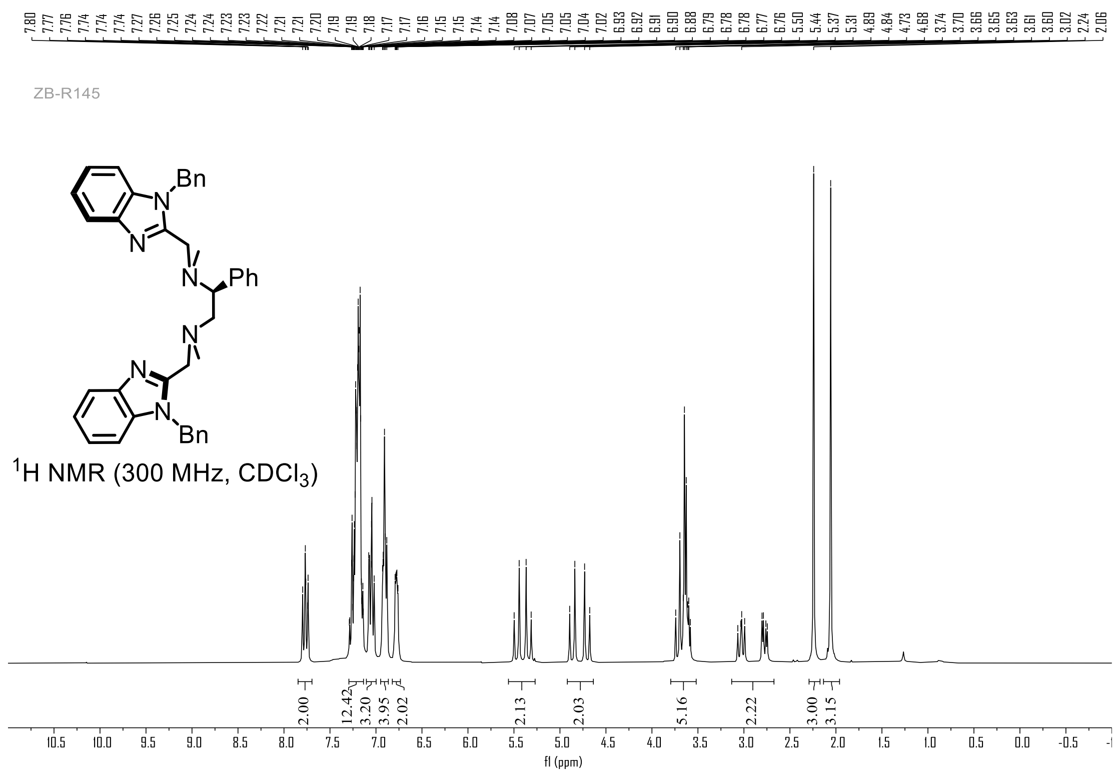

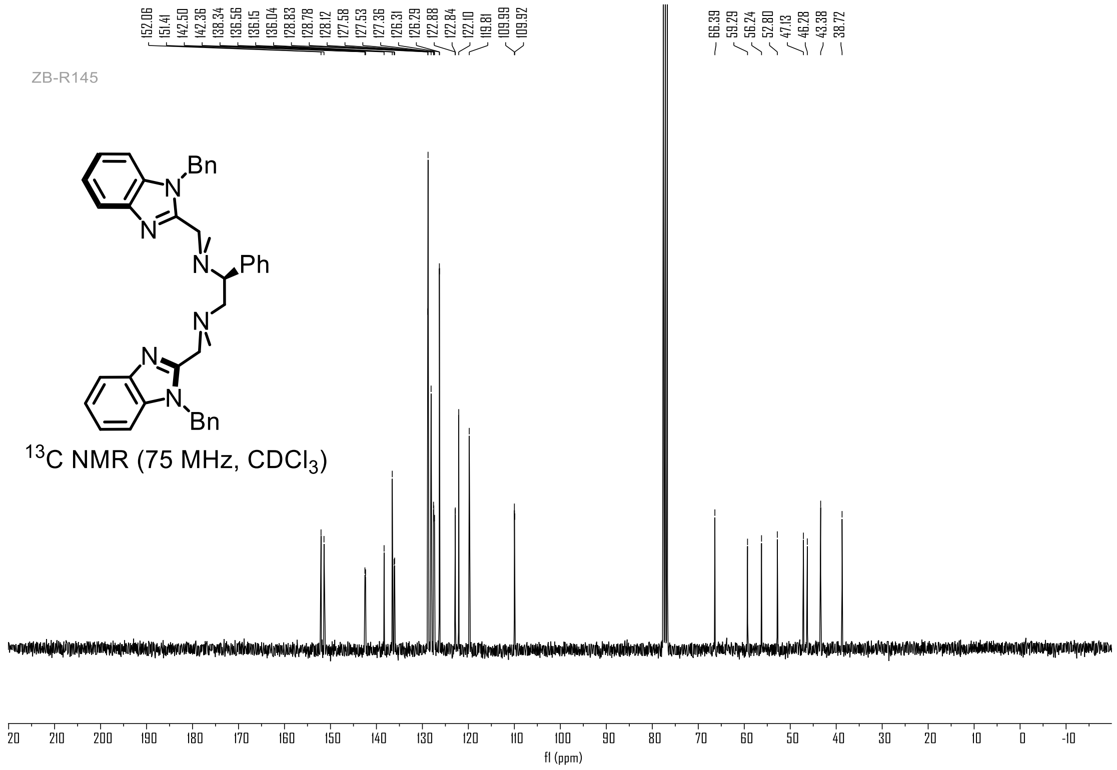

Figure S49. ^{1}H NMR, ^{13}C NMR spectra of **S2-Bn**.

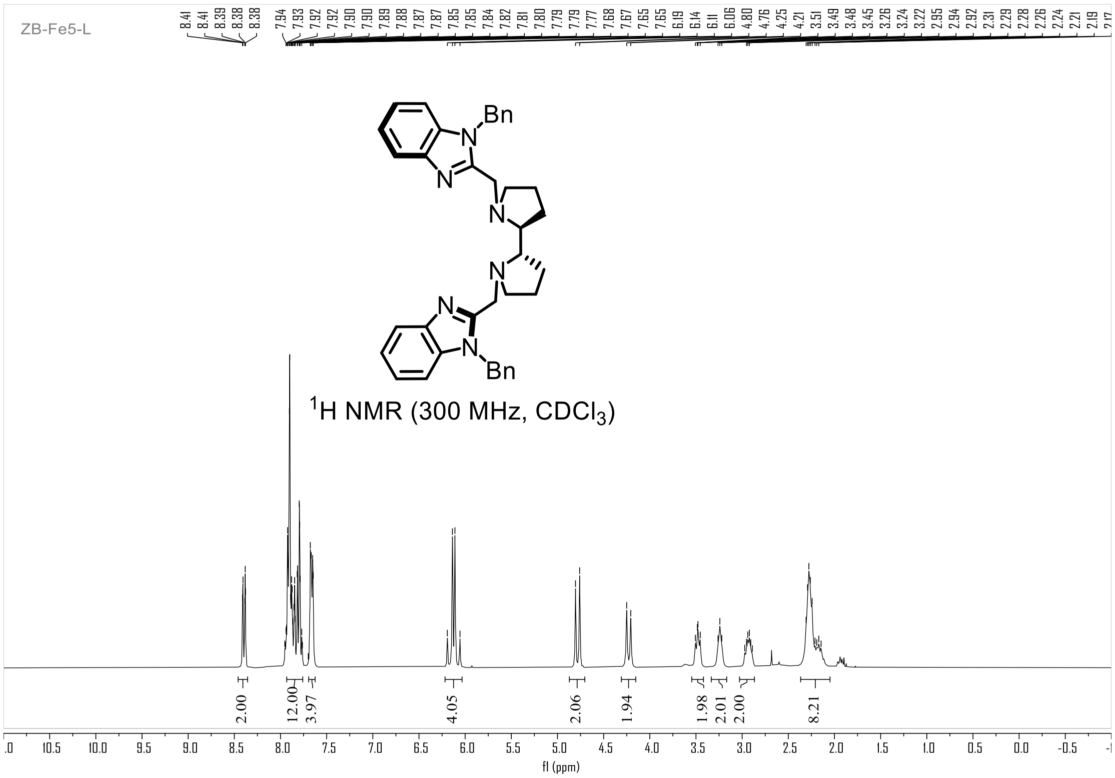

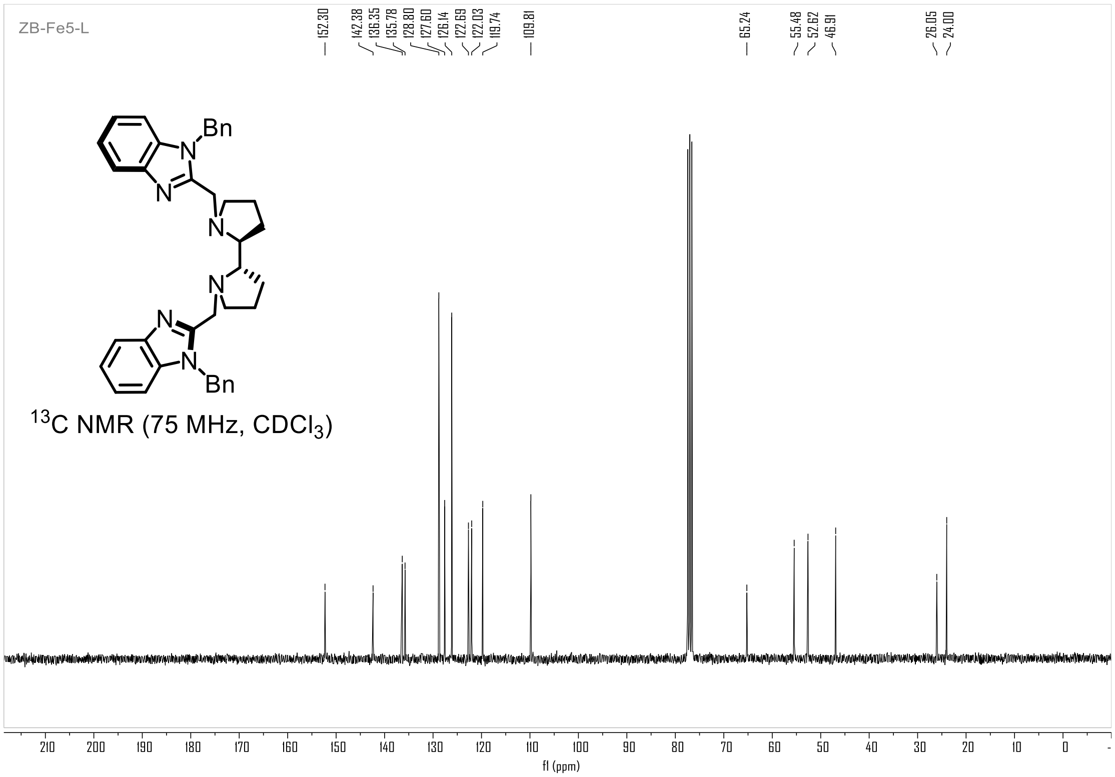

Figure S50. ^{1}H NMR, ^{13}C NMR spectra of **S3**.

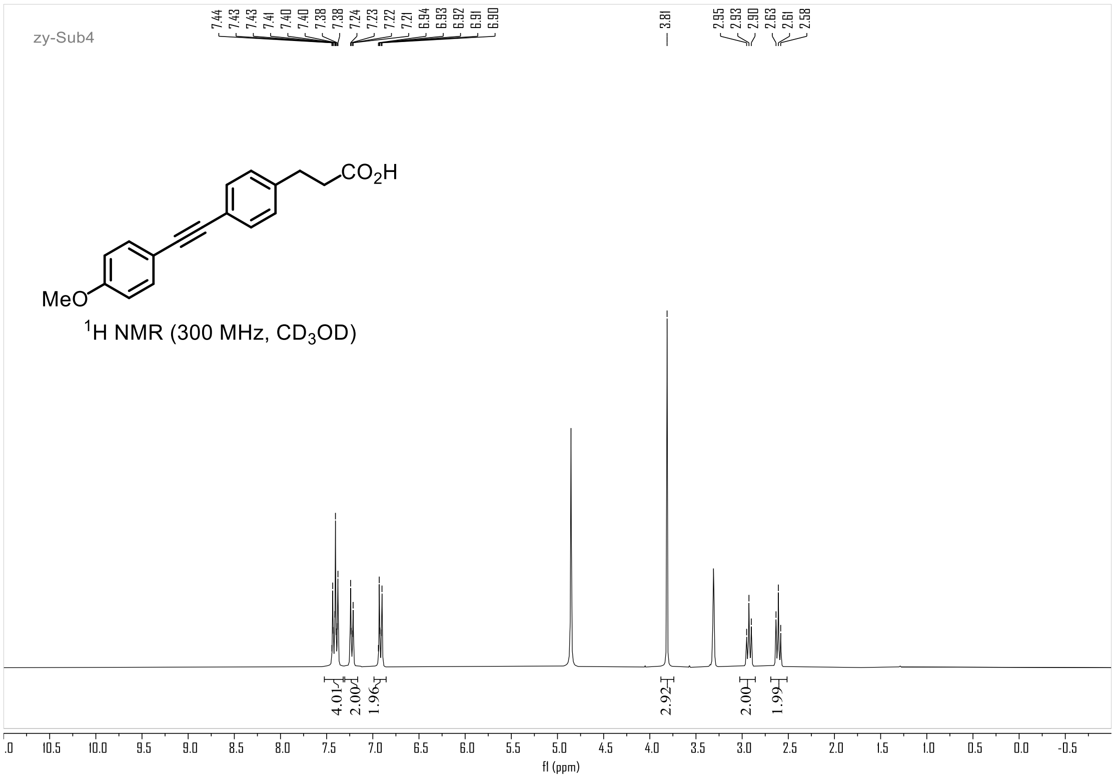

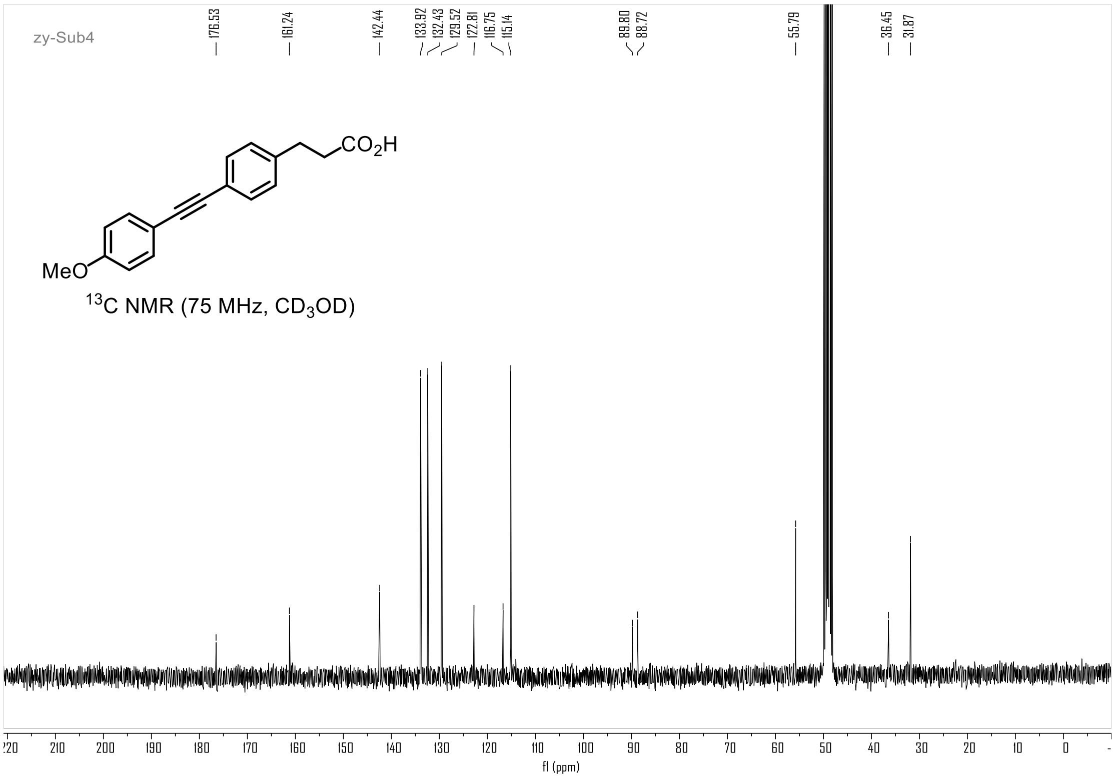

Figure S51. ^{1}H NMR, ^{13}C NMR spectra of **20a**.

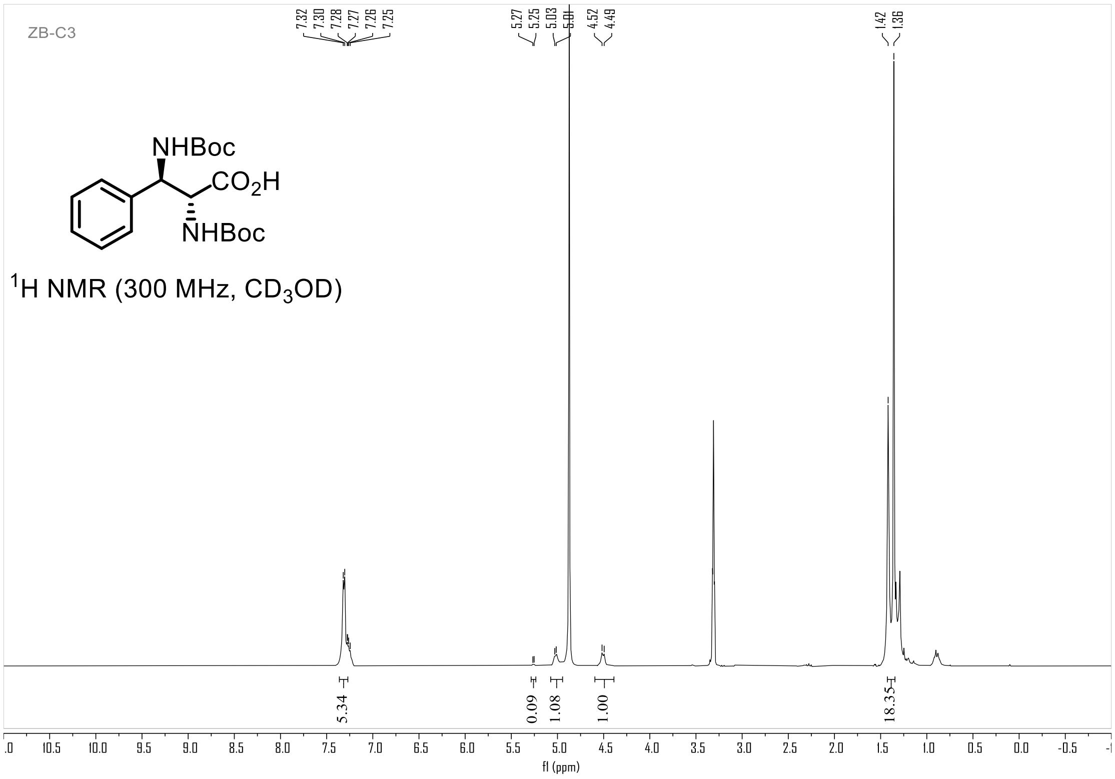

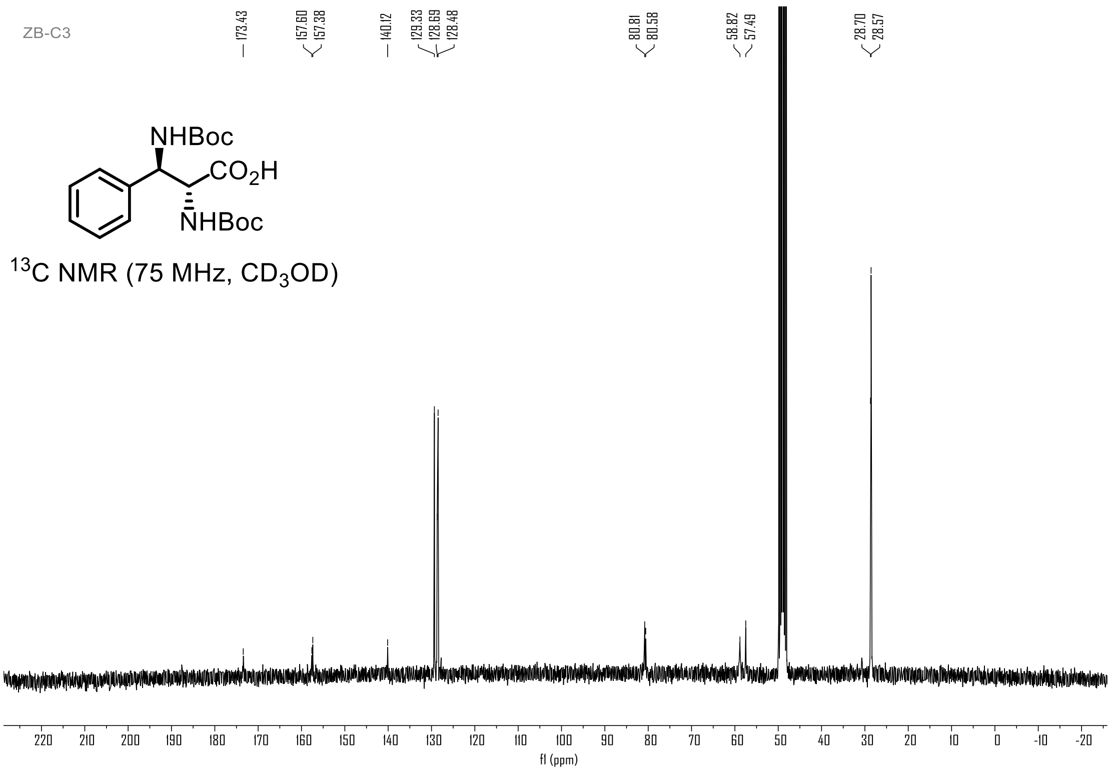

Figure S52. ^{1}H NMR, ^{13}C NMR spectra of **2a**.

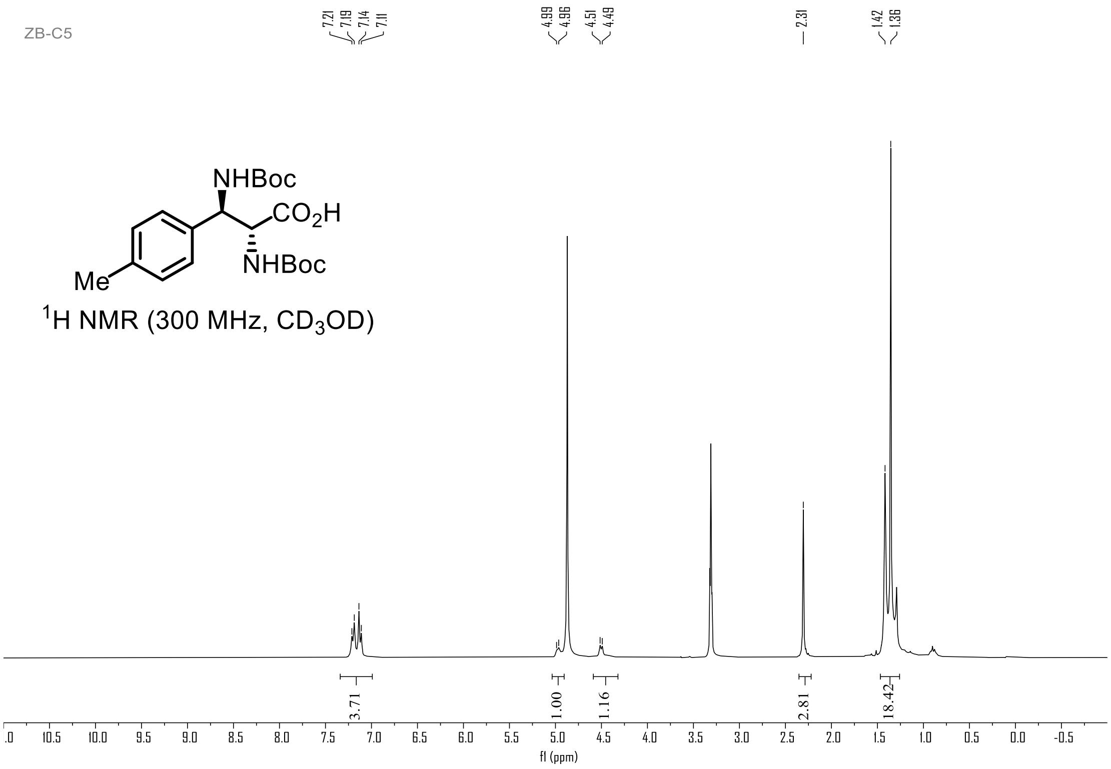

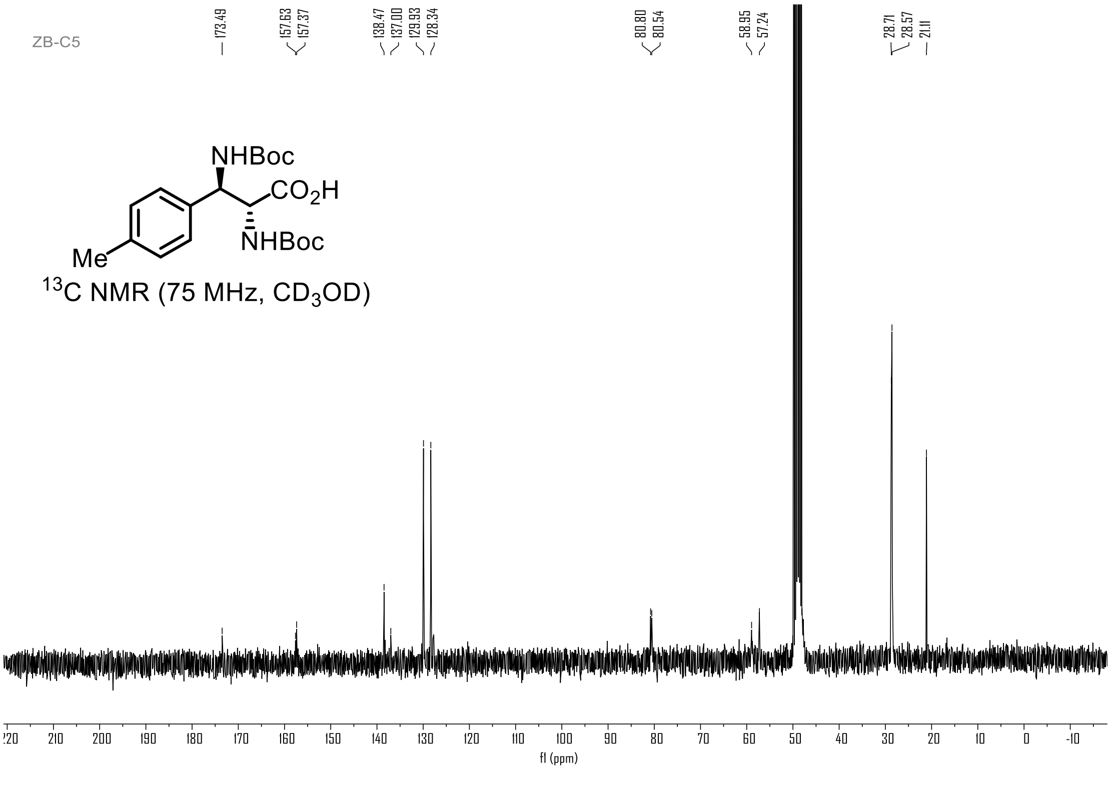

Figure S53. ^{1}H NMR, ^{13}C NMR spectra of **3**.

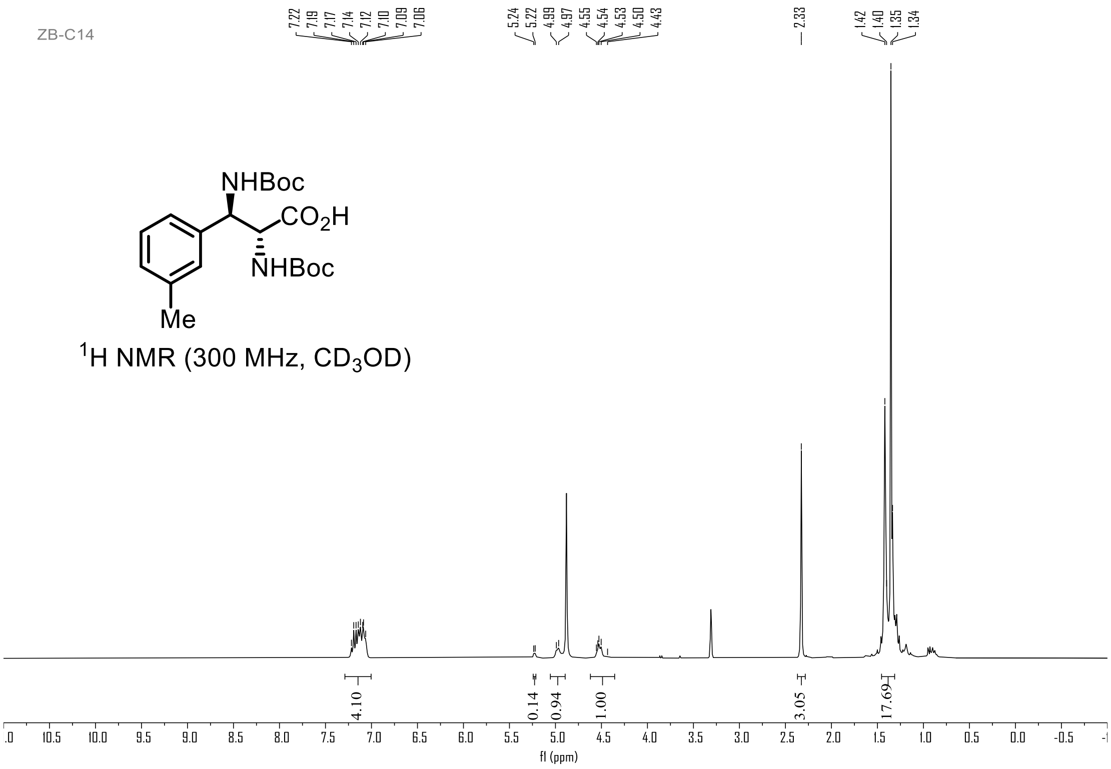

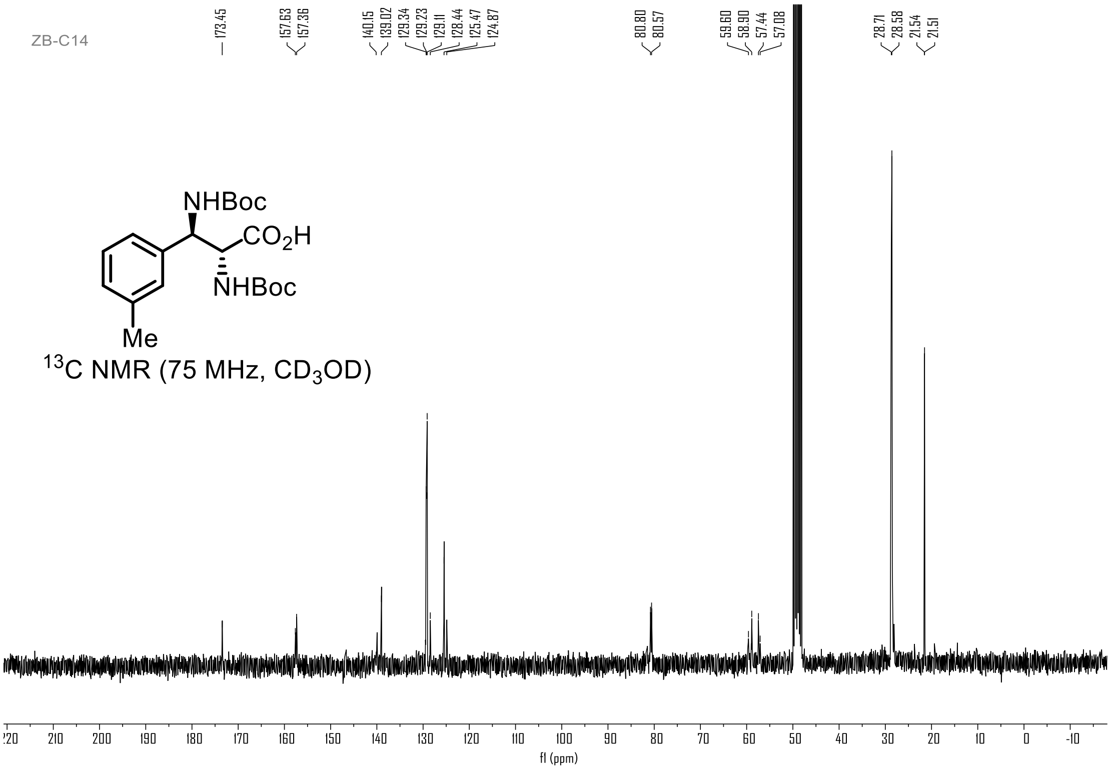

Figure S54. ^{1}H NMR, ^{13}C NMR spectra of **4**.

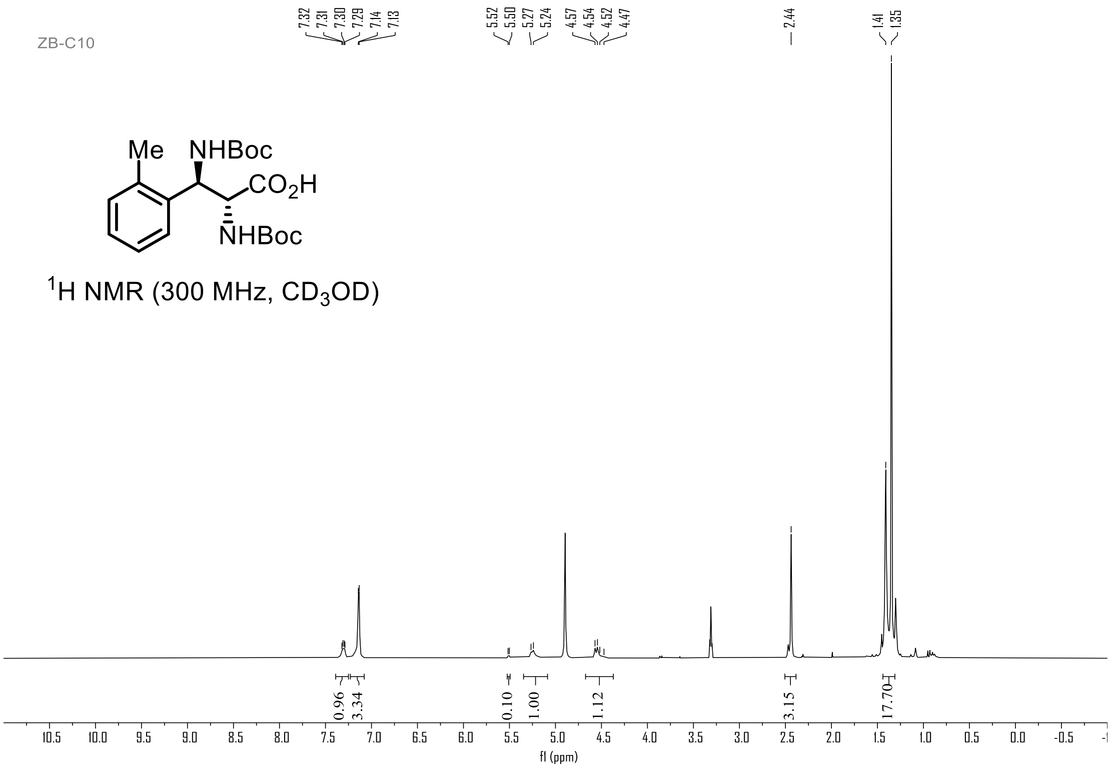

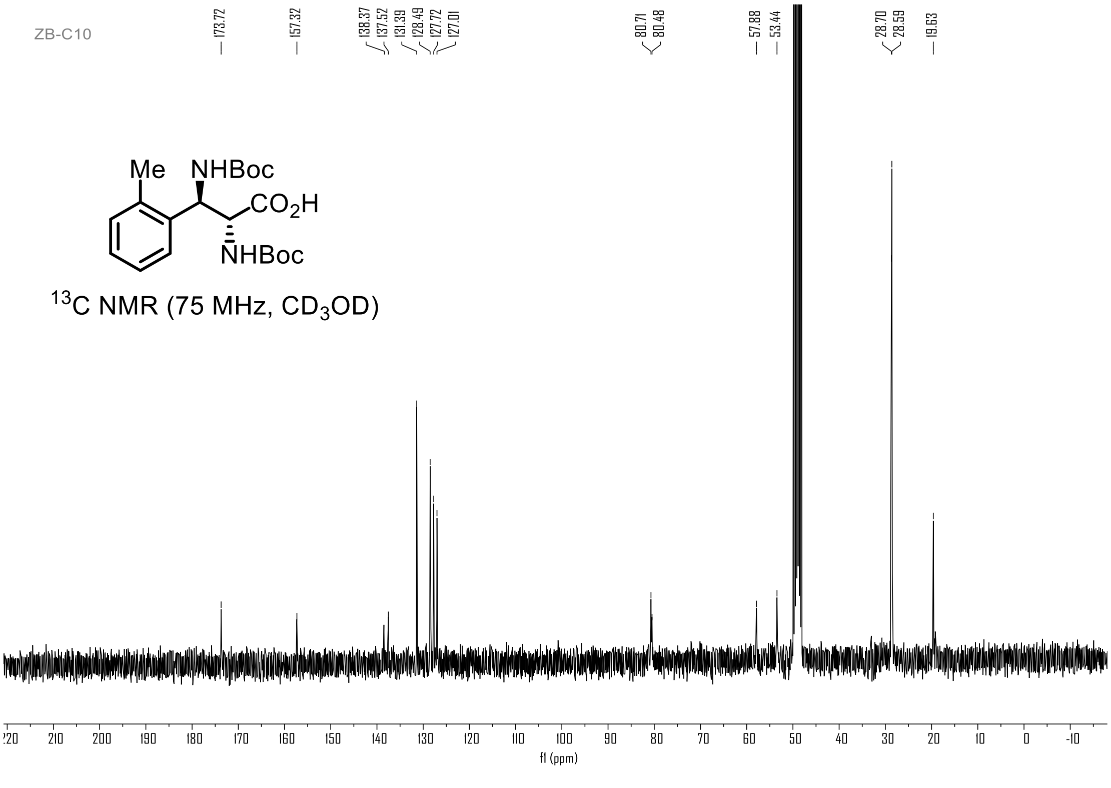

Figure S55. ^{1}H NMR, ^{13}C NMR spectra of **5**.

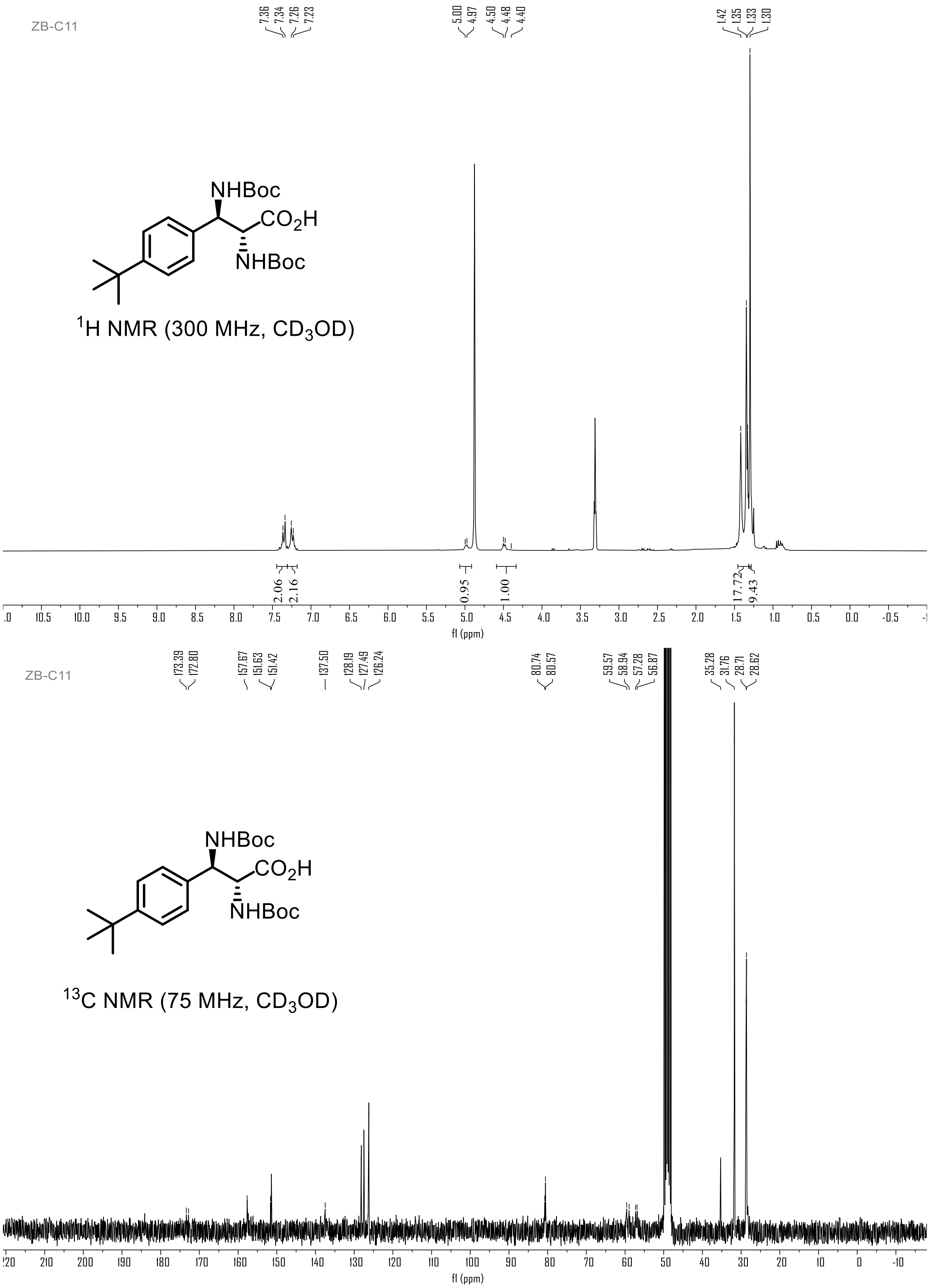

Figure S56. ^{1}H NMR, ^{13}C NMR spectra of **6**.

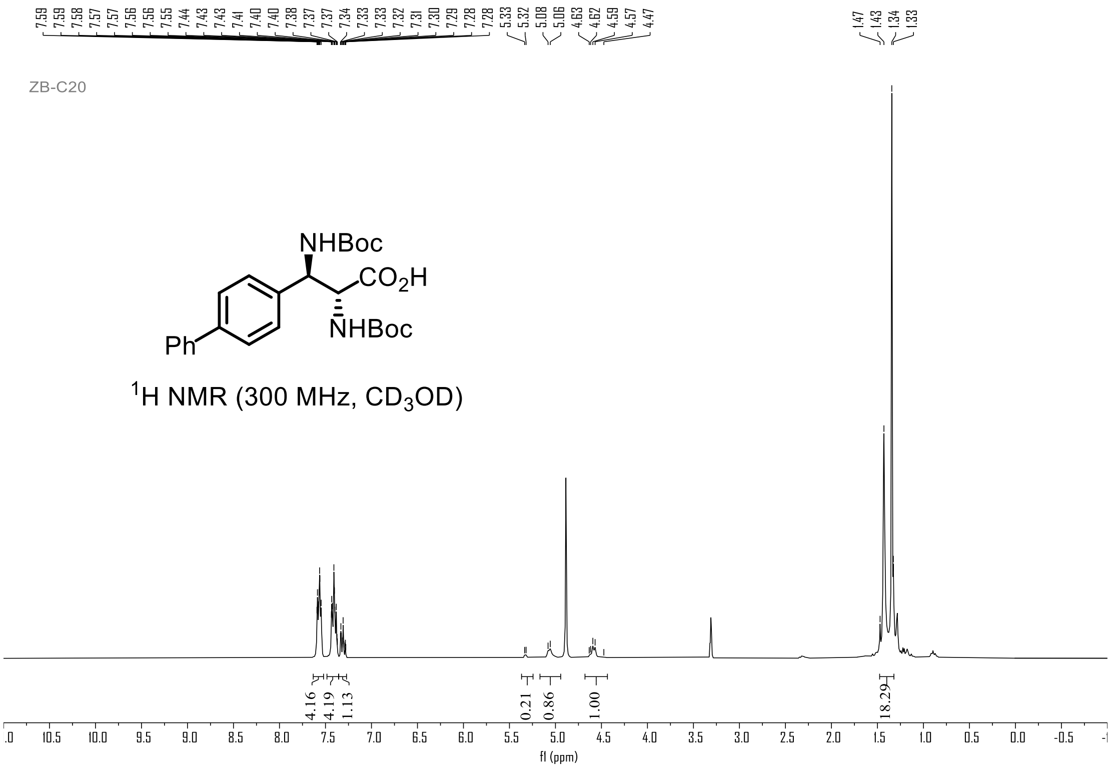

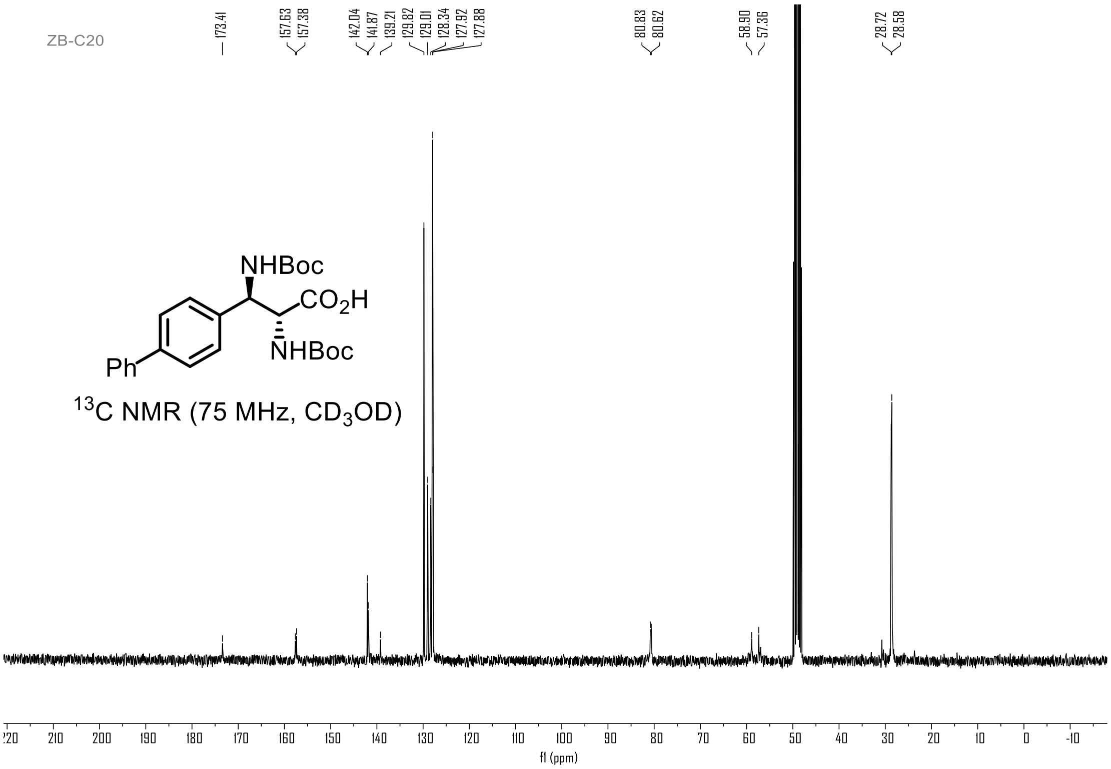

Figure S57. ^{1}H NMR, ^{13}C NMR spectra of **7**.

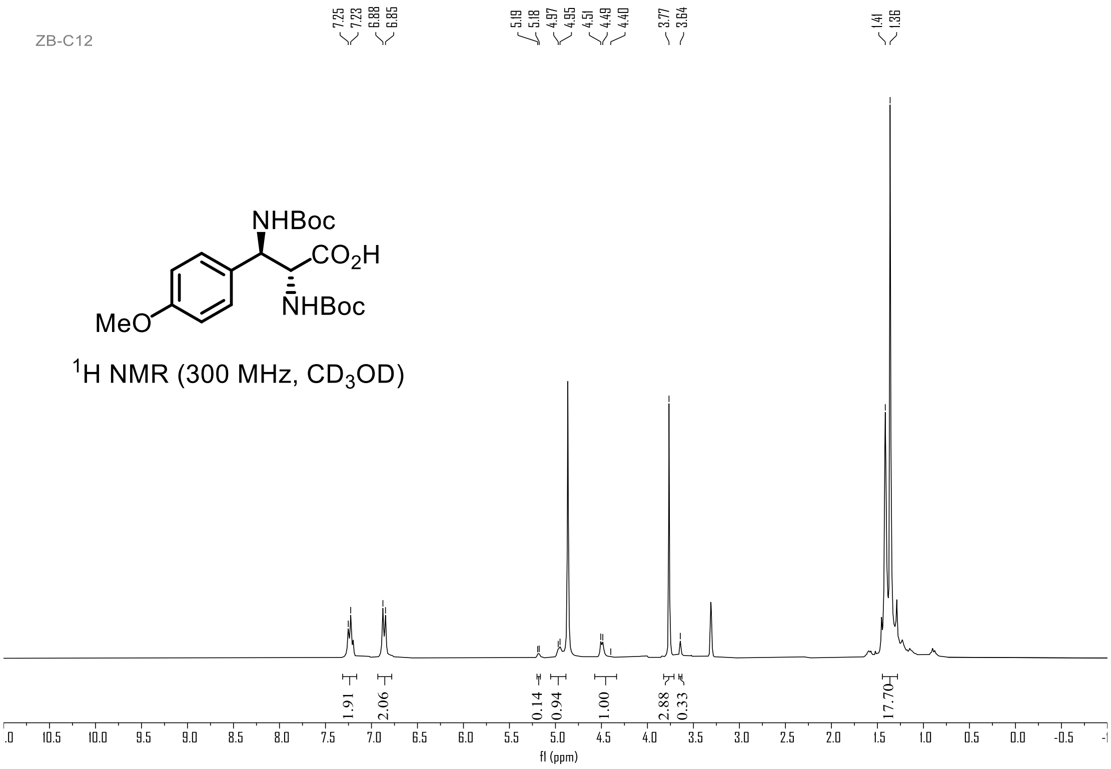

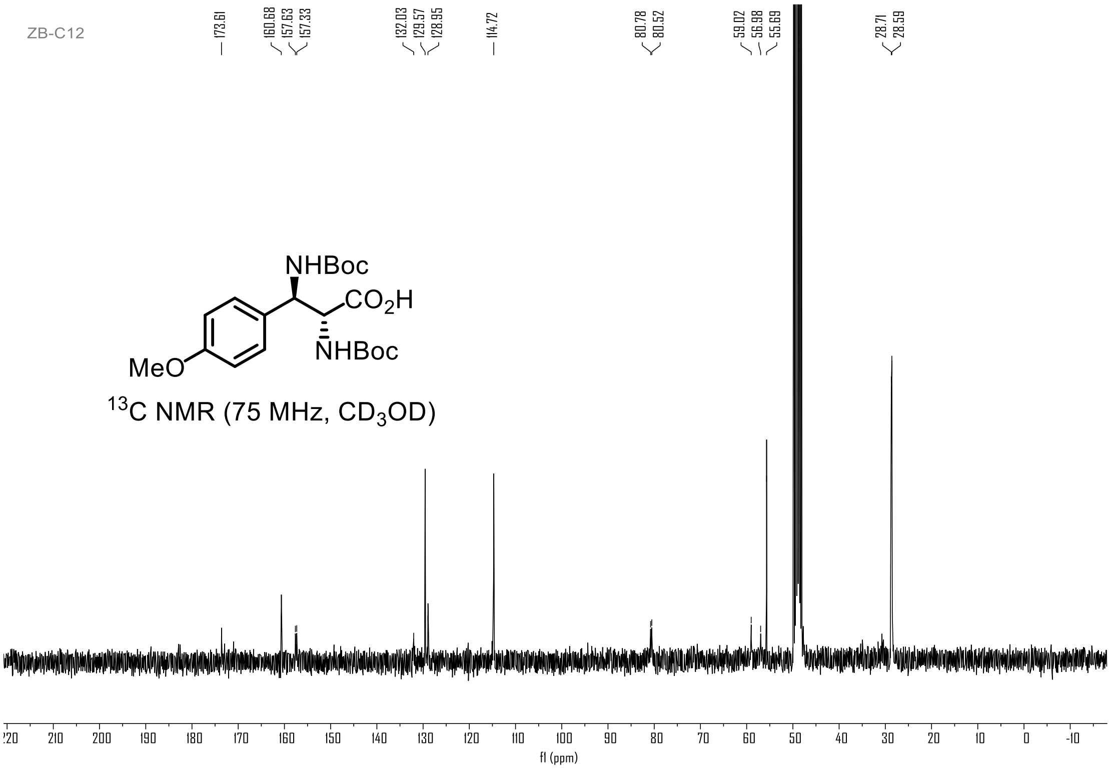

Figure S58. ^1H NMR, ^{13}C NMR spectra of **8**.

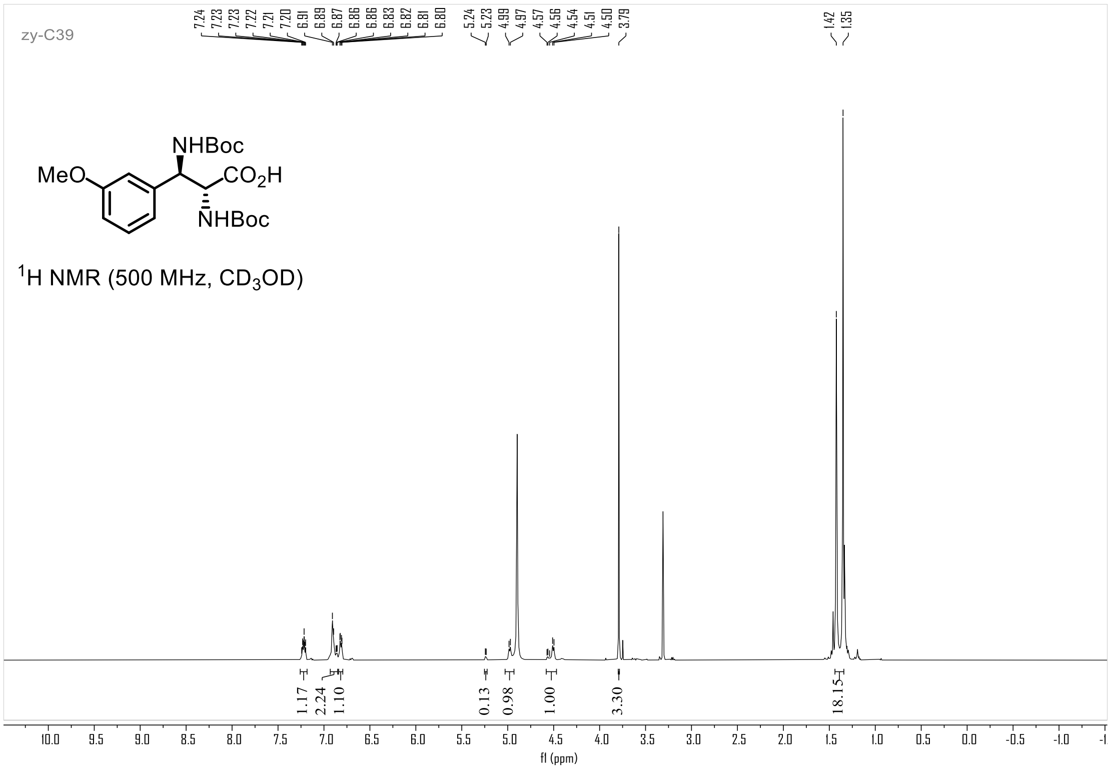

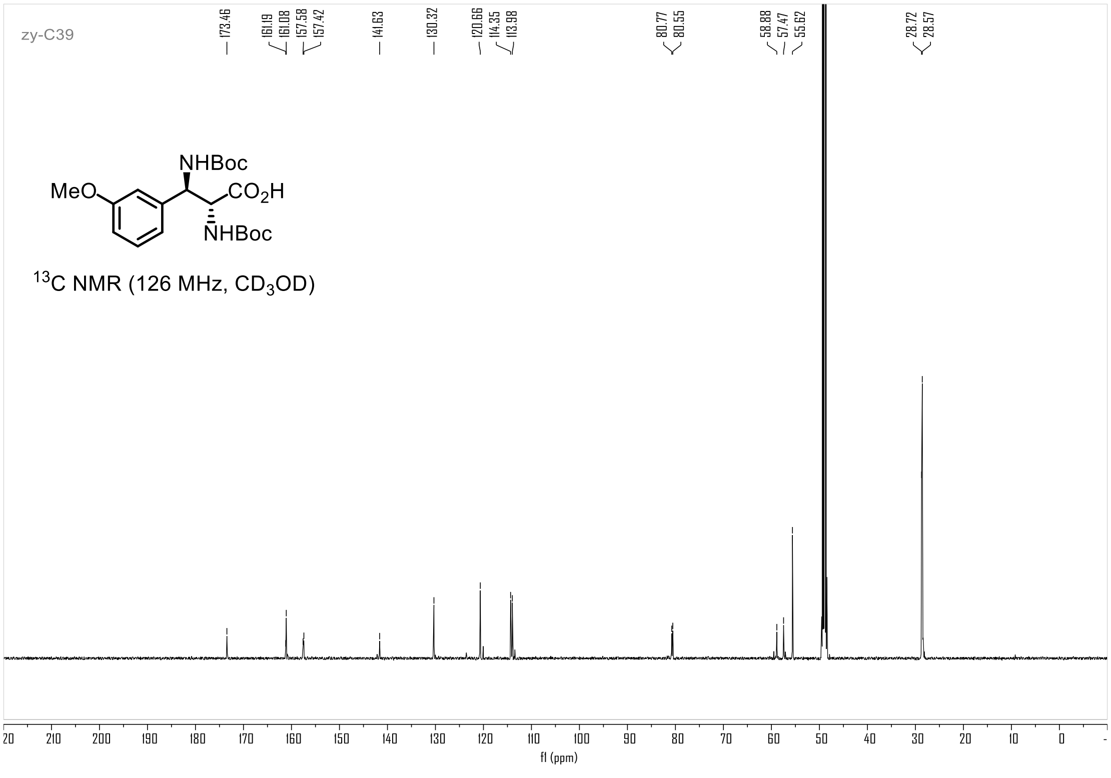

Figure S59. ^1H NMR, ^{13}C NMR spectra of **9**

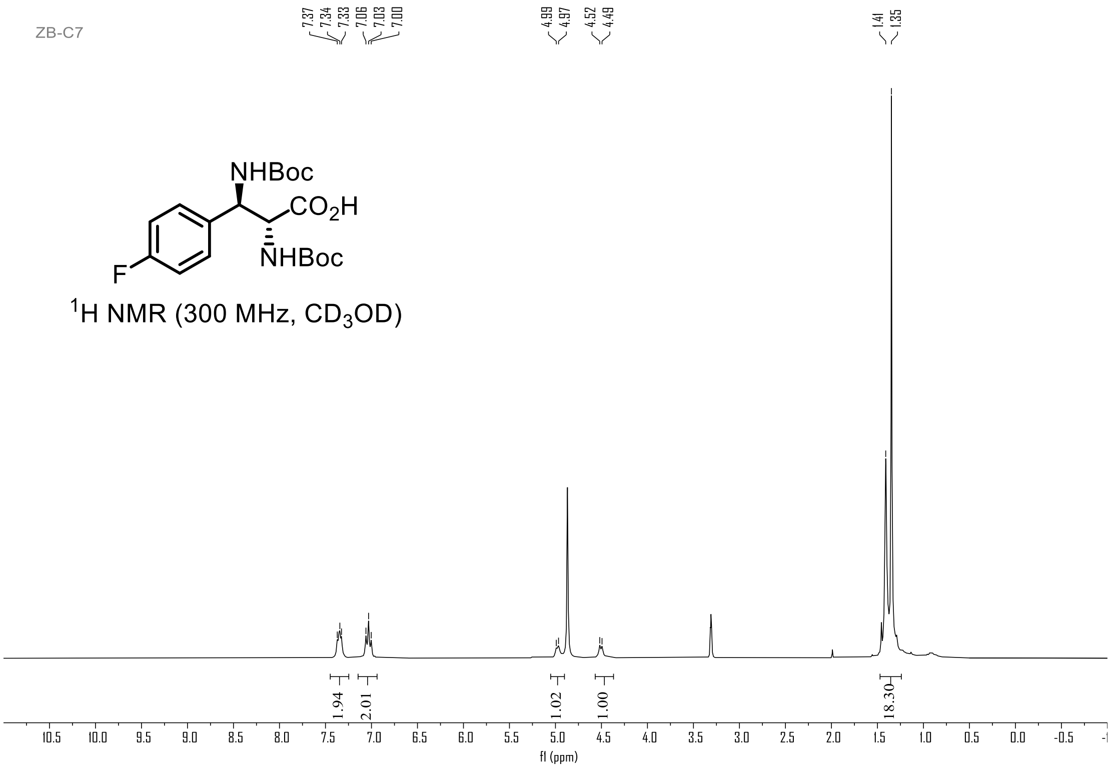
ZB-C7
7.37
7.34
7.33
7.06
7.03
7.00
4.99
4.97
4.52
4.49
1.41
1.35
NHBoc
CO2H
NHBoc
F
1H NMR (300 MHz, CD3OD)
1.94
2.01
1.02
1.00
18.30
f1 (ppm)

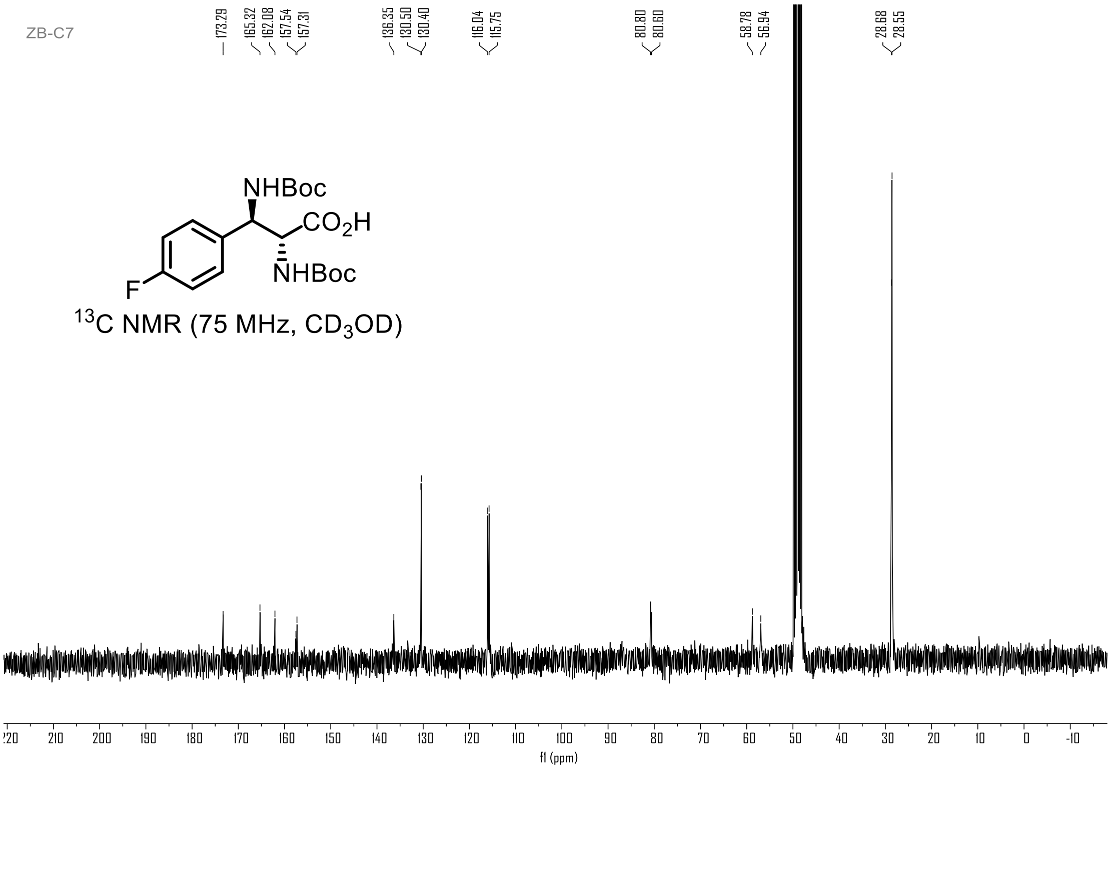
ZB-C7
173.29
165.32
162.08
157.54
157.31
136.35
130.50
130.40
116.04
115.75
80.80
80.60
58.78
56.94
28.68
28.55
NHBoc
CO2H
NHBoc
F
13C NMR (75 MHz, CD3OD)
f1 (ppm)

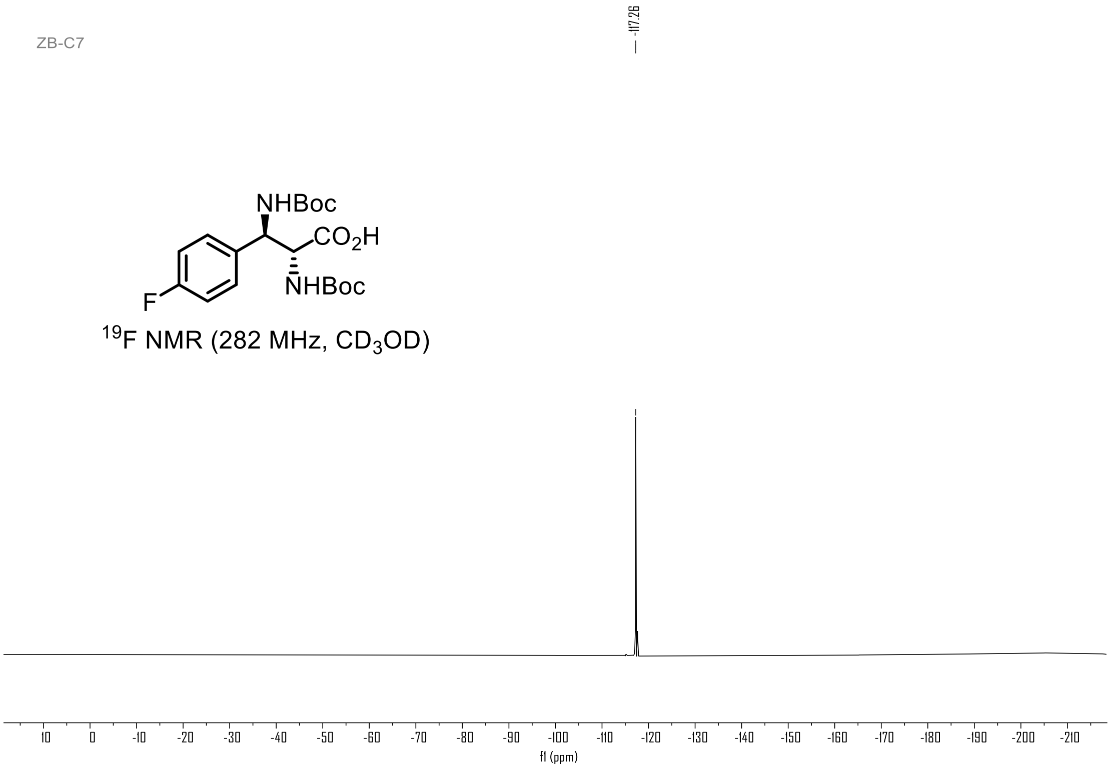

Figure S60. ^{1}H NMR, ^{13}C NMR, ^{19}F NMR spectra of **10**.

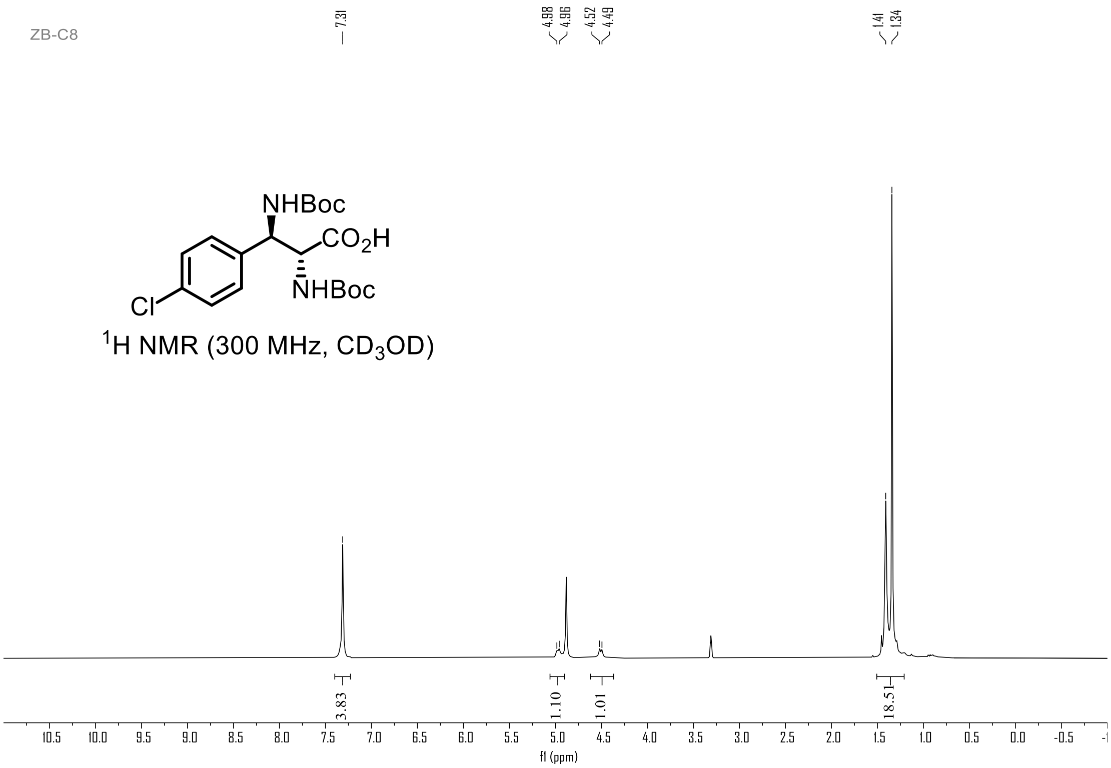

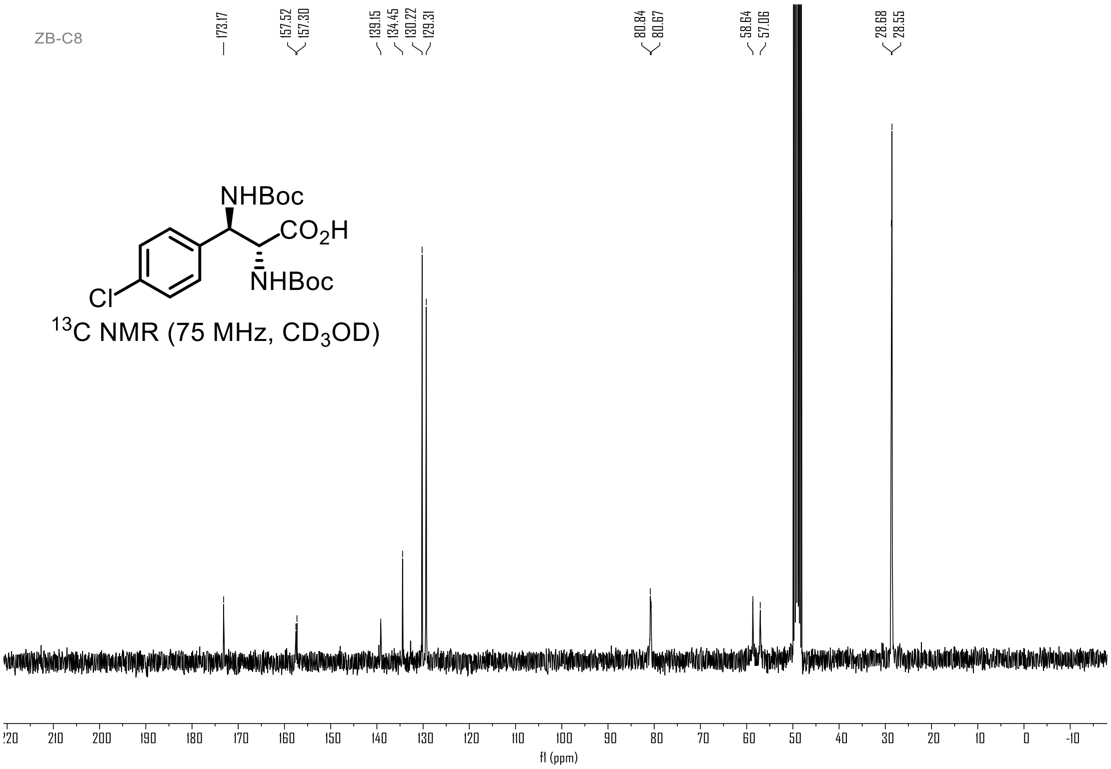

Figure S61. ^{1}H NMR, ^{13}C NMR spectra of **11**.

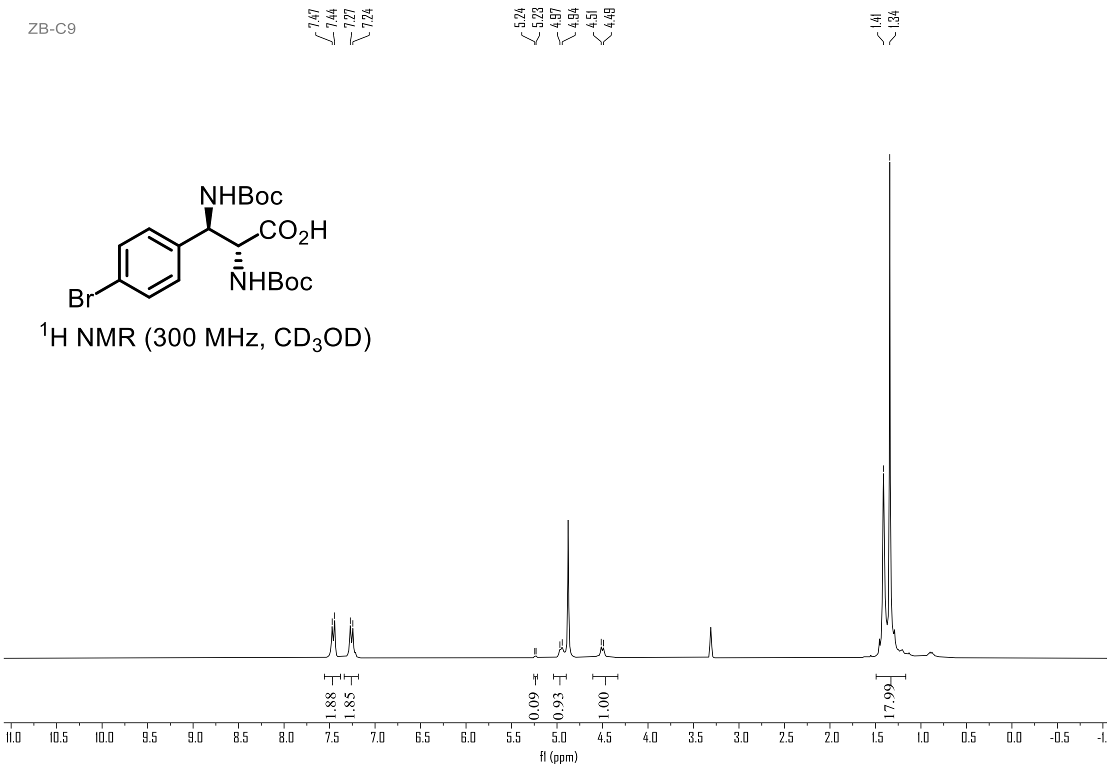

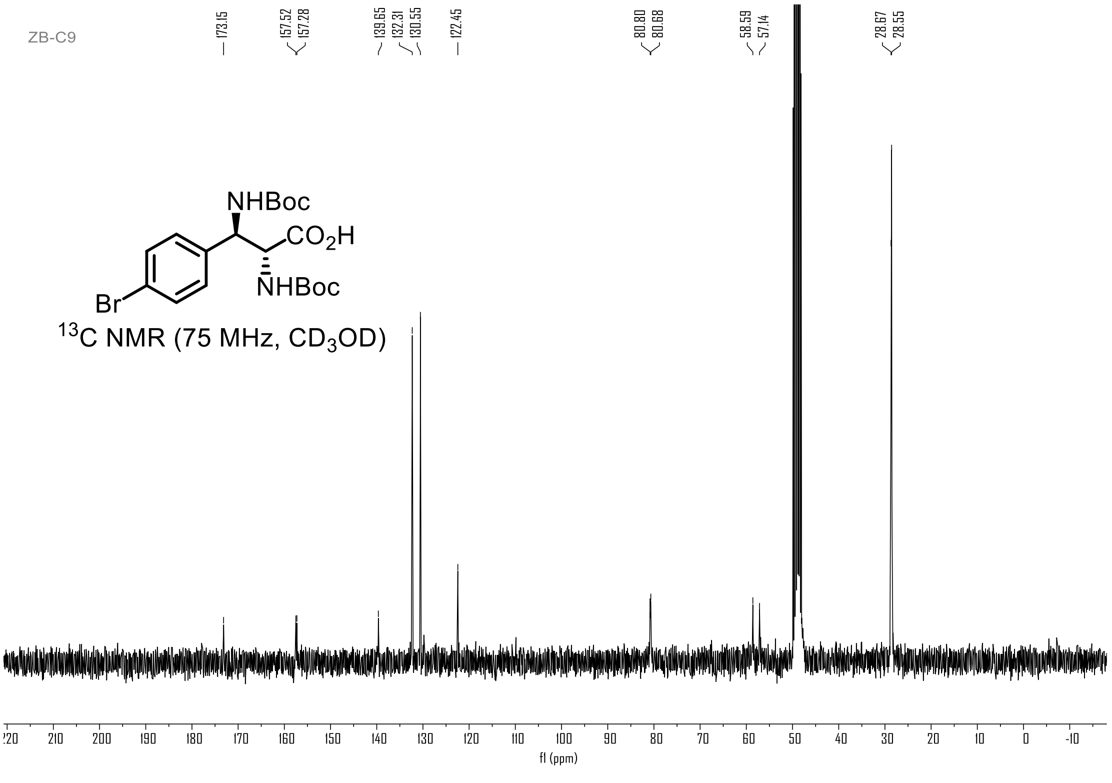

Figure S62. ^1H NMR, ^{13}C NMR spectra of **12**.

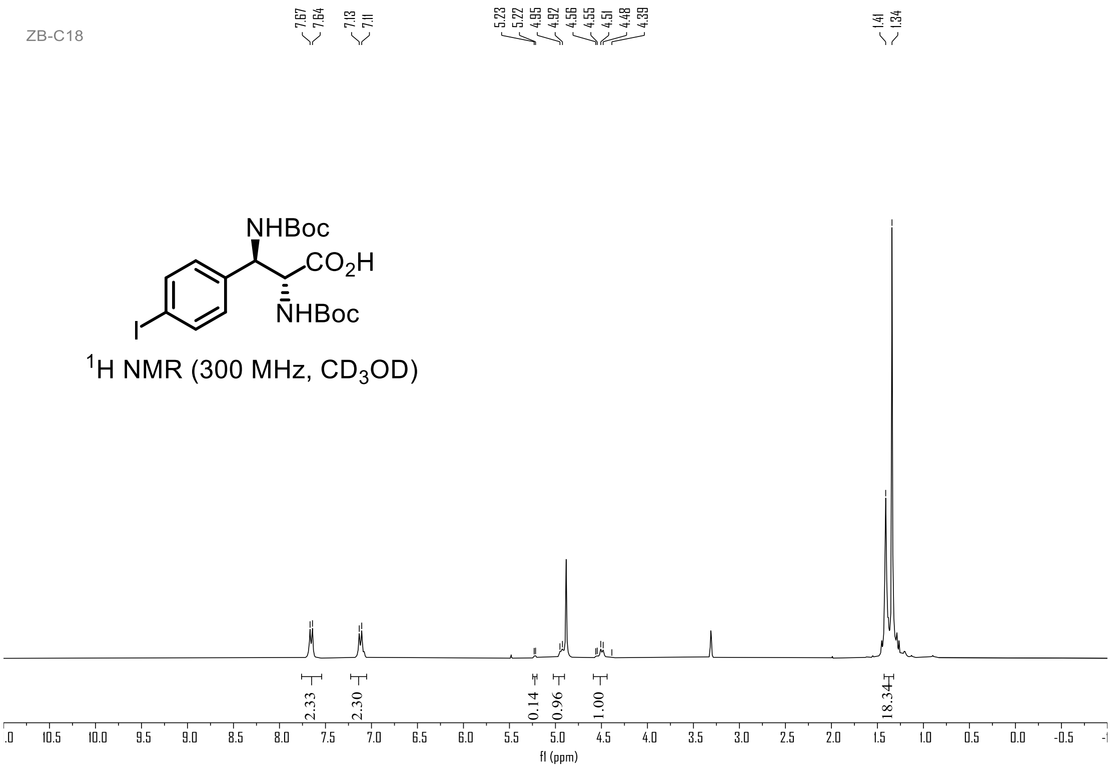

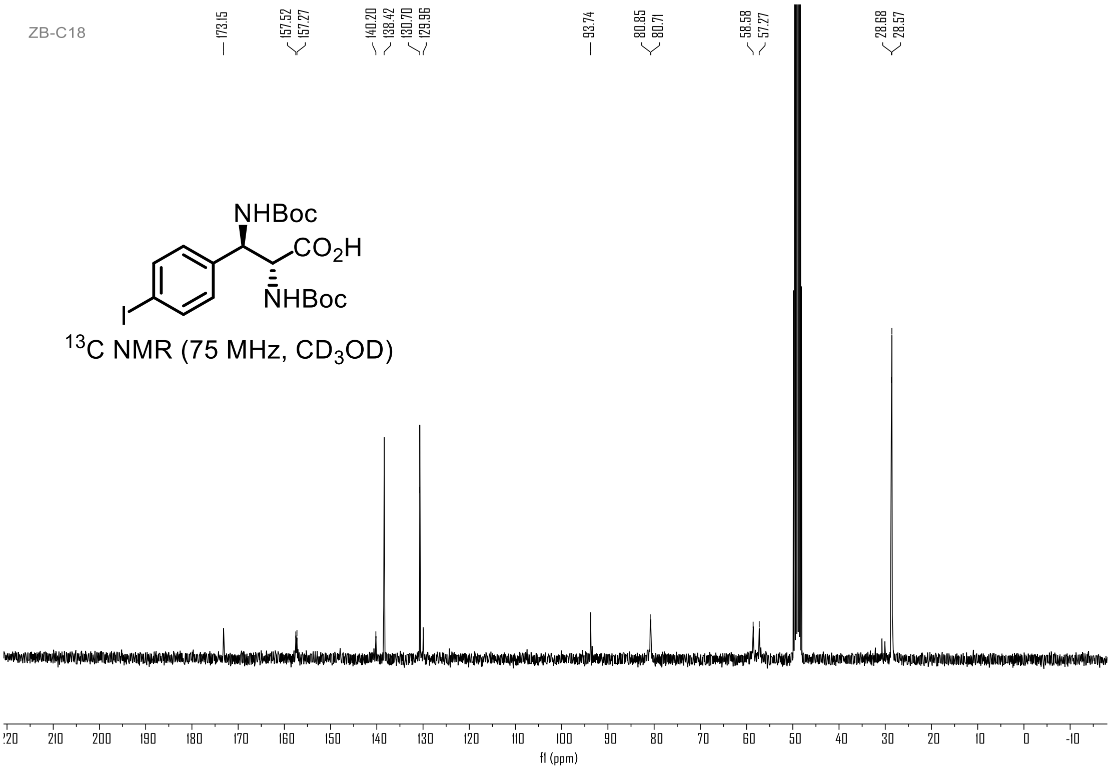

Figure S63. ^{1}H NMR, ^{13}C NMR spectra of **13**.

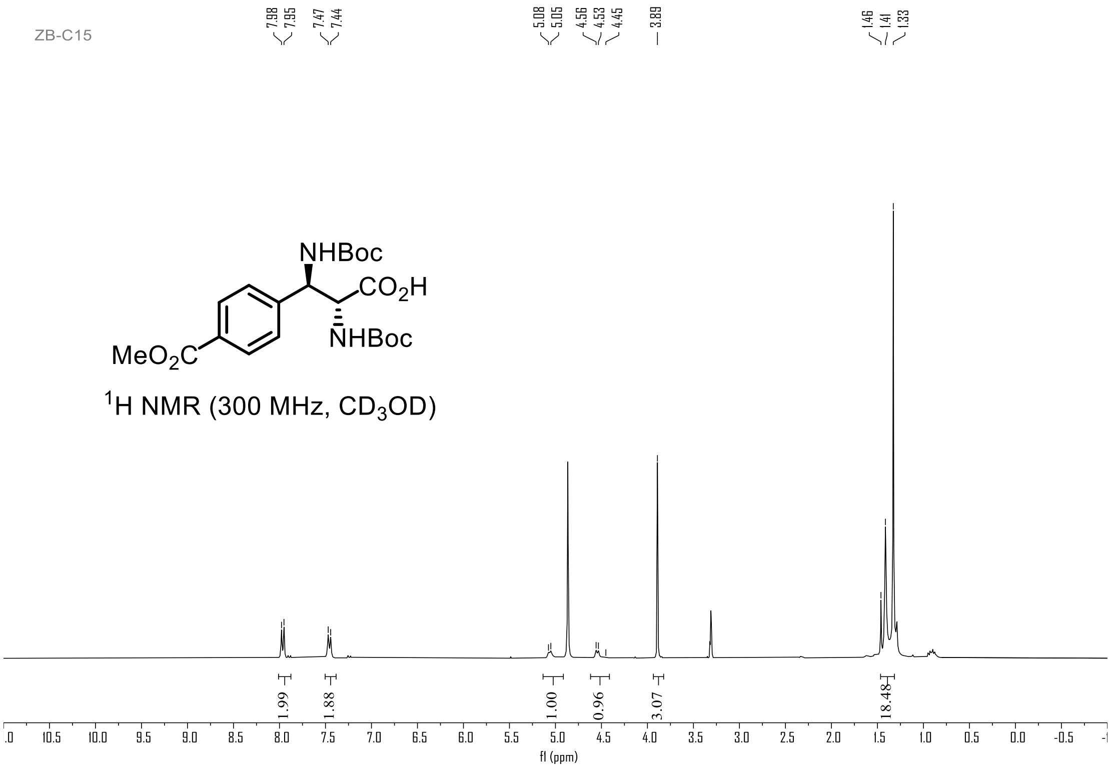

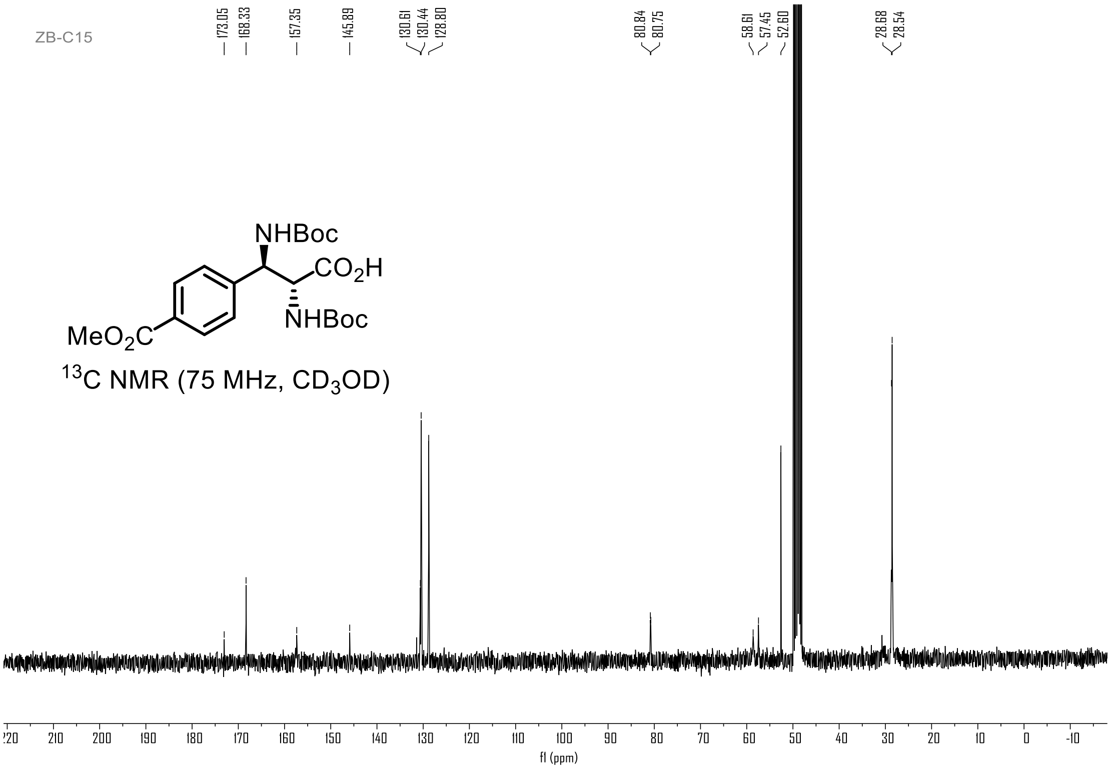

Figure S64. ^{1}H NMR, ^{13}C NMR spectra of **14**.

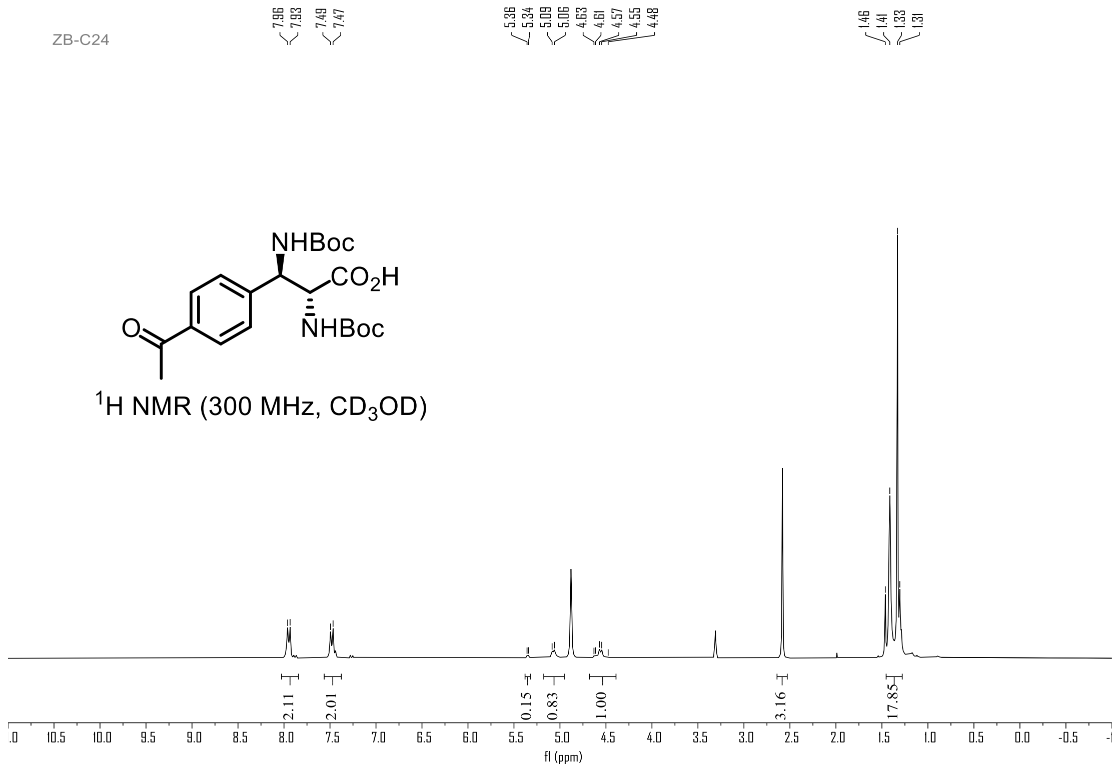

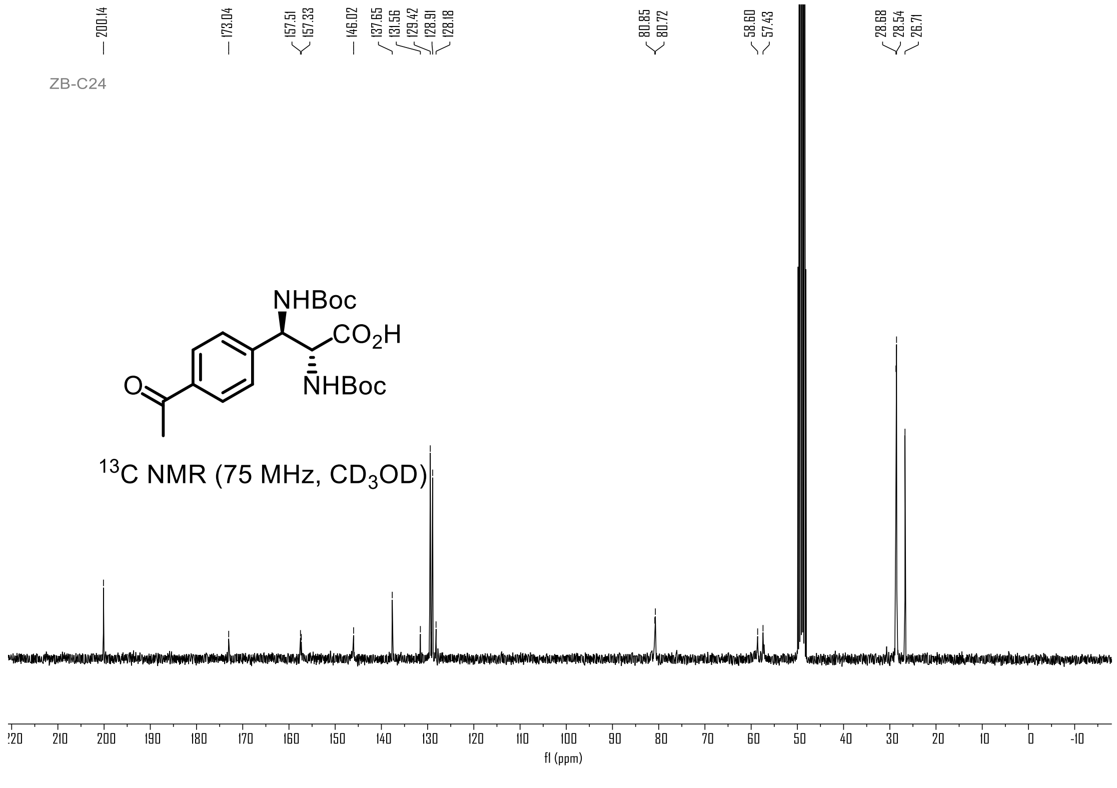

Figure S65. ^{1}H NMR, ^{13}C NMR spectra of **15**.

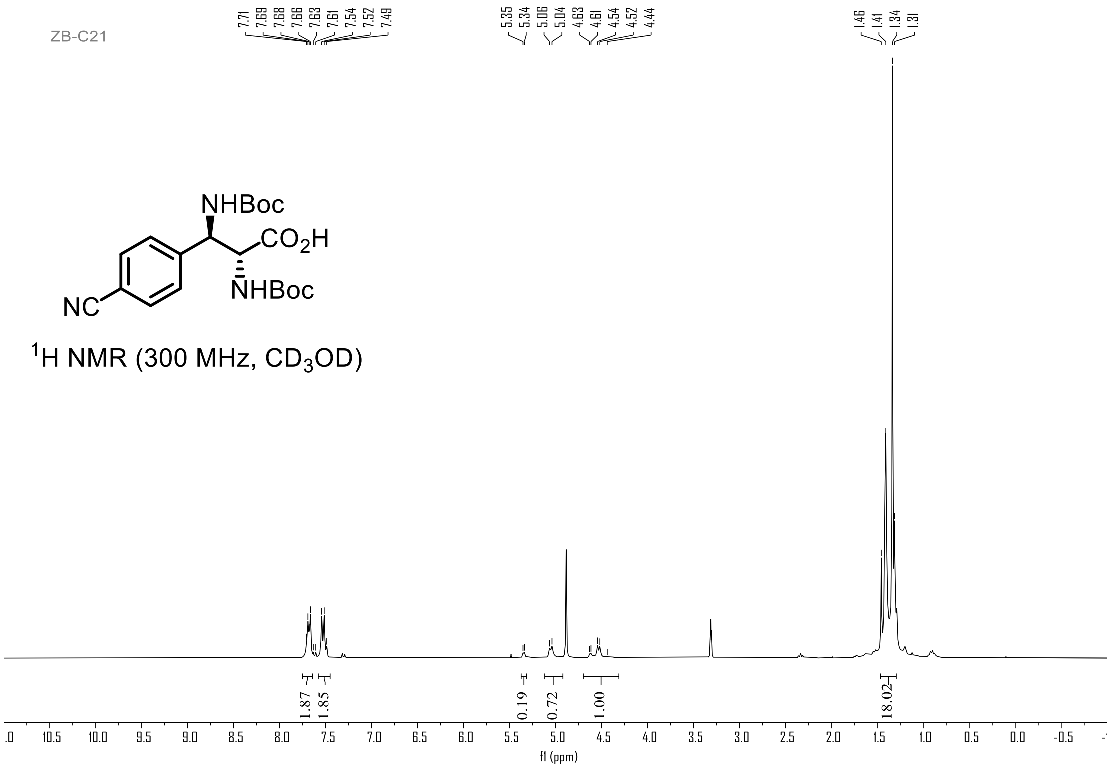

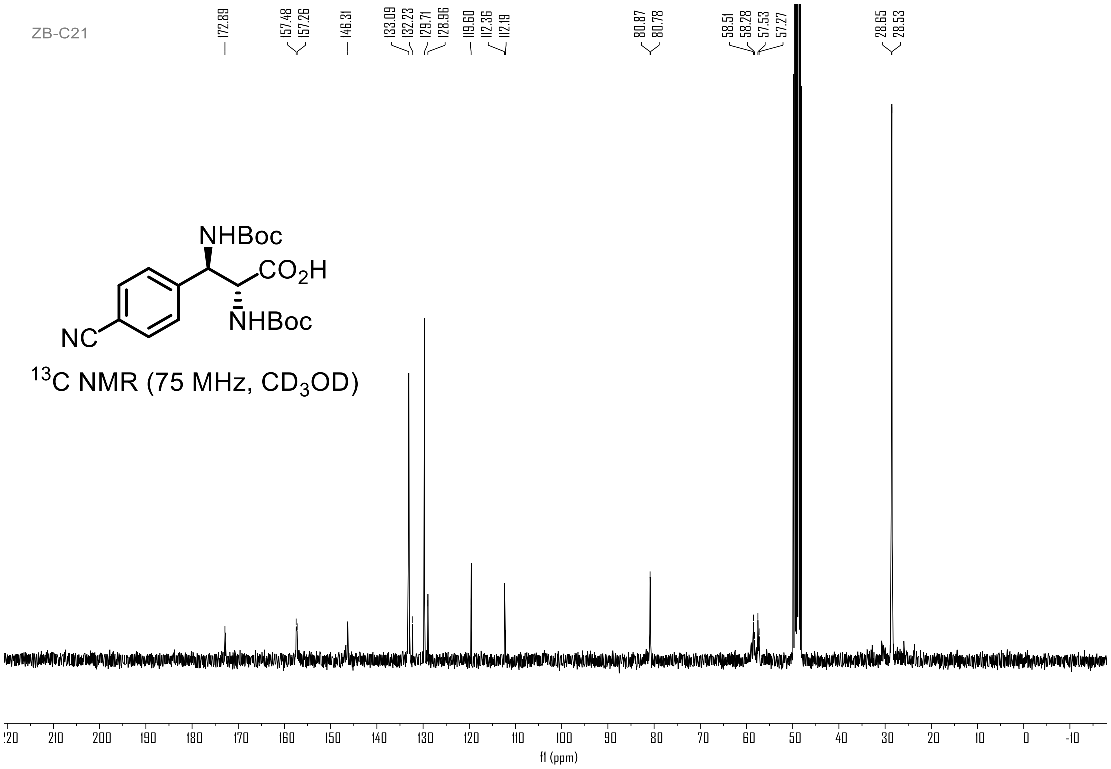

Figure S66. ^{1}H NMR, ^{13}C NMR spectra of **16**.

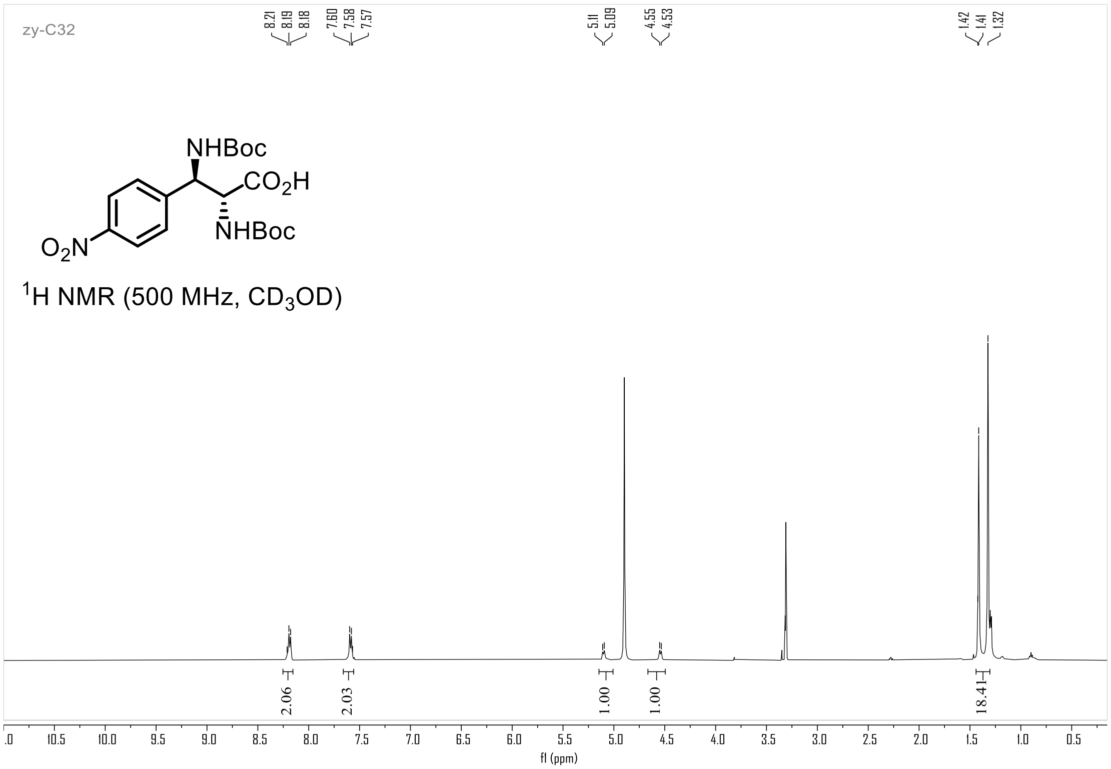

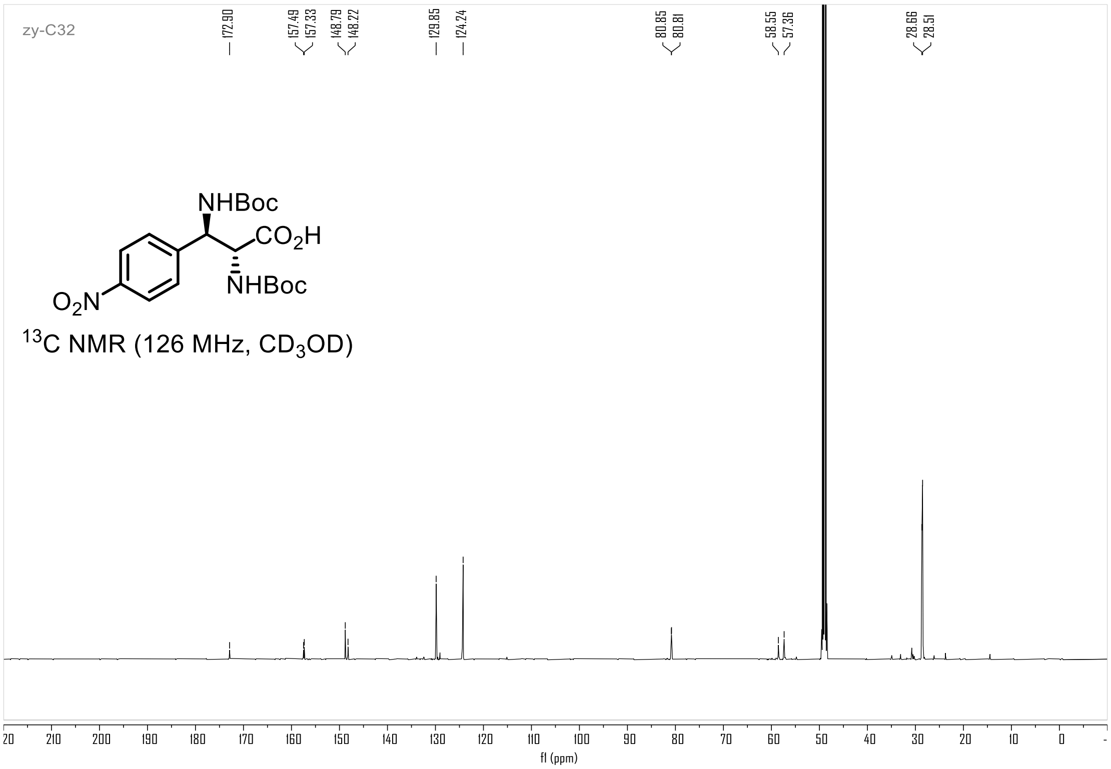

Figure S67. ^{1}H NMR, ^{13}C NMR spectra of **17**.

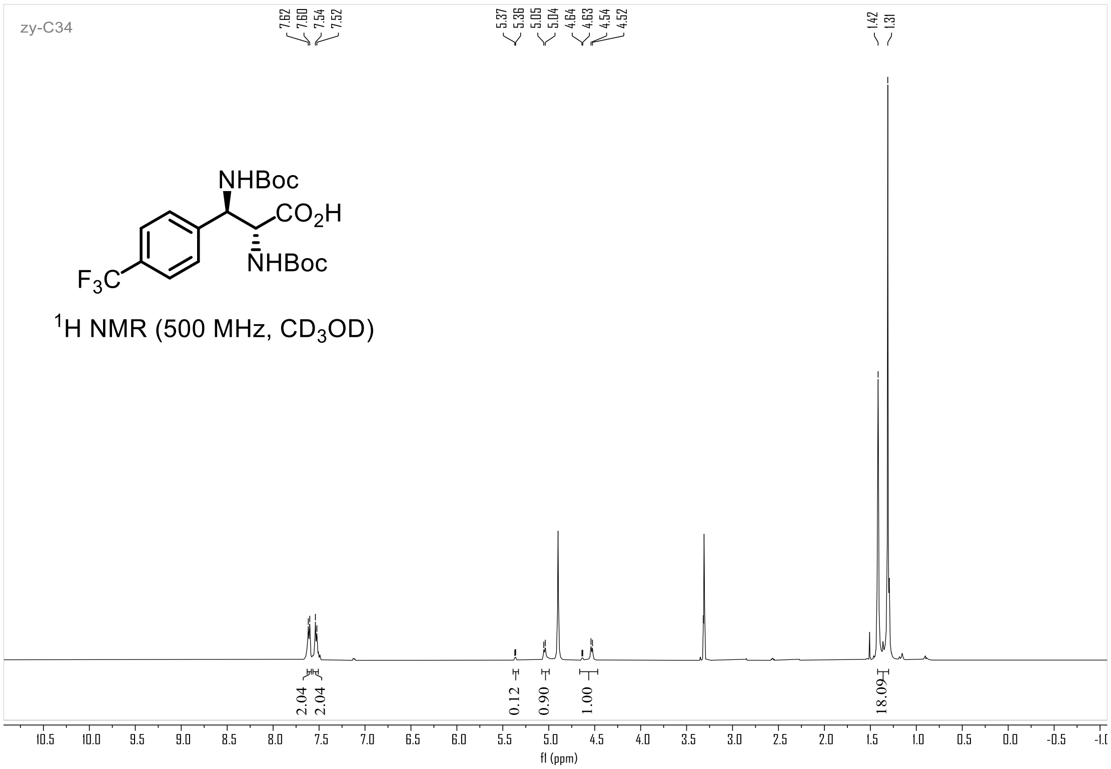
zy-C34
7.62
7.60
7.54
7.52
5.37
5.36
5.05
5.04
4.64
4.63
4.54
4.52
1.42
1.31
NHBoc
CO_2H
NHBoc
F_3C
^{1}H NMR (500 MHz, CD_3OD)
2.04
2.04
0.12
0.90
1.00
18.09
fl (ppm)

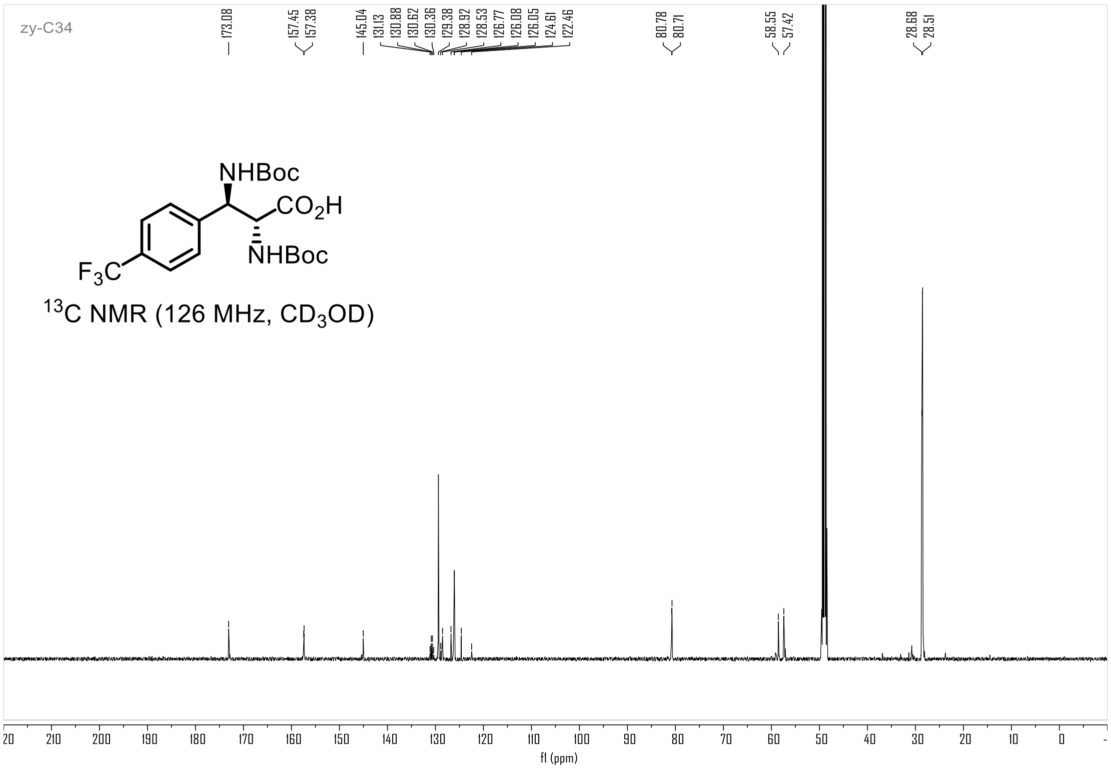
zy-C34
173.08
157.45
157.38
145.04
131.13
130.88
130.62
130.36
129.38
128.92
128.53
126.77
126.08
126.05
124.61
122.46
80.78
80.71
58.55
57.42
28.68
28.51
NHBoc
CO_2H
NHBoc
F_3C
^{13}C NMR (126 MHz, CD_3OD)
fl (ppm)

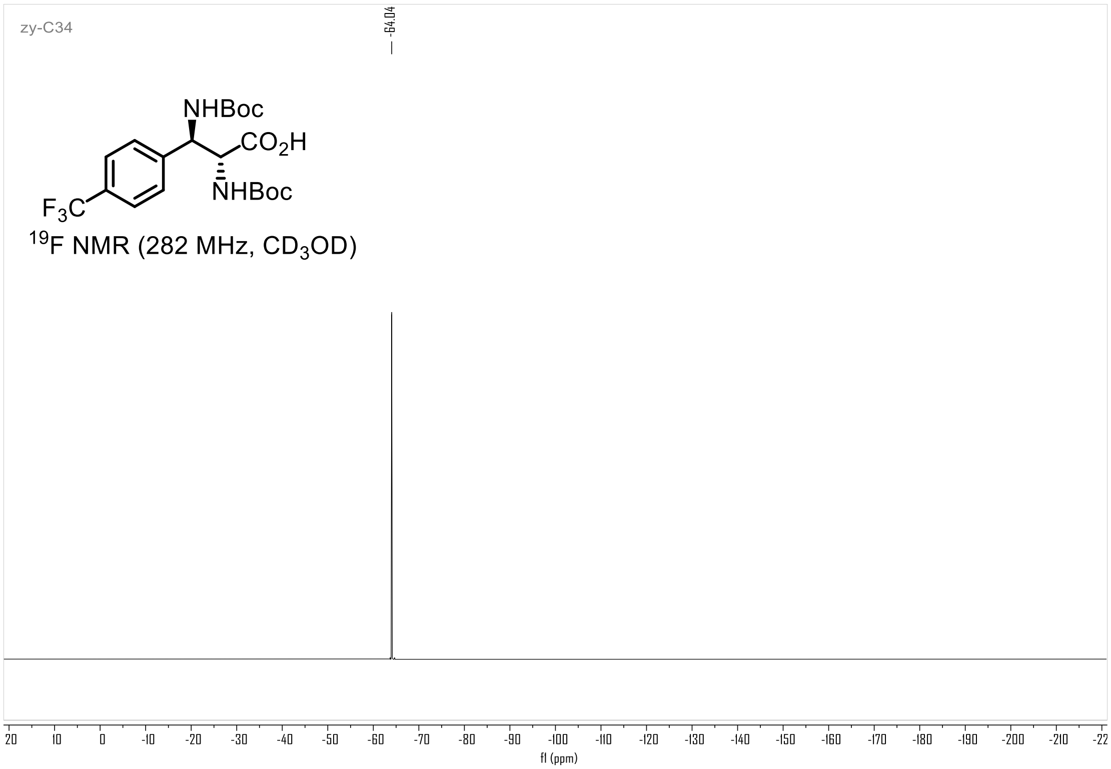

Figure S68. ^{1}H NMR, ^{13}C NMR , ^{19}F NMR spectra of **18**.

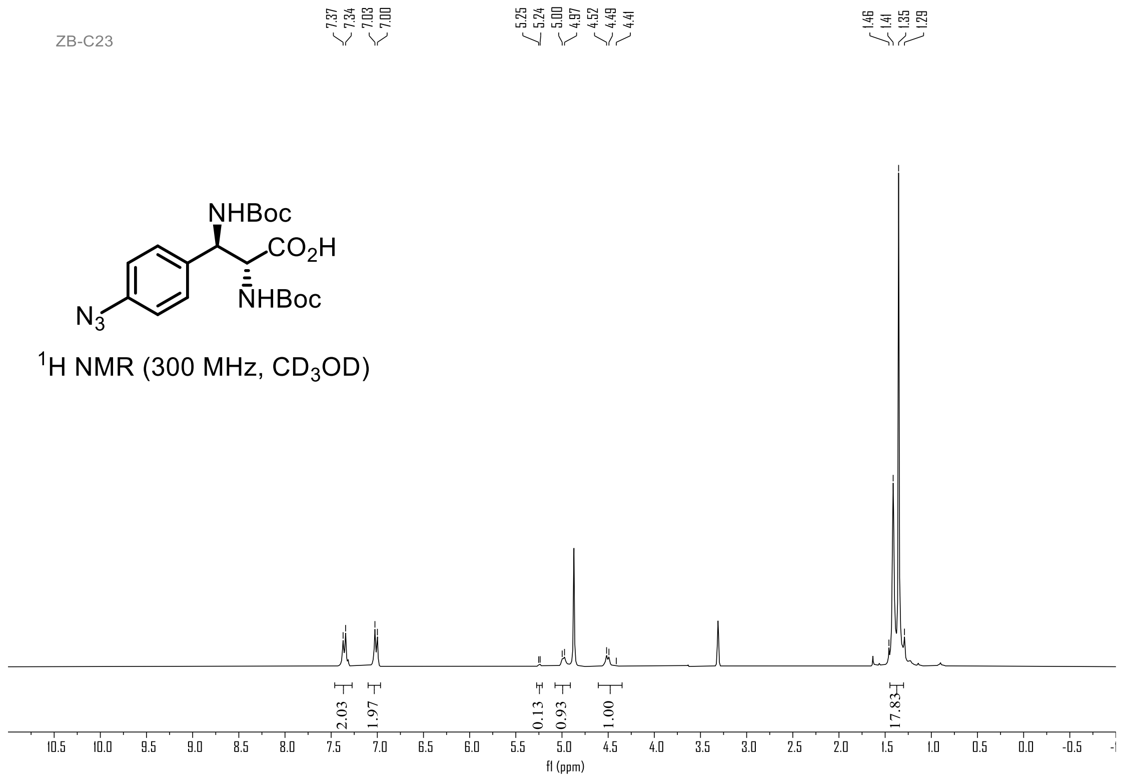

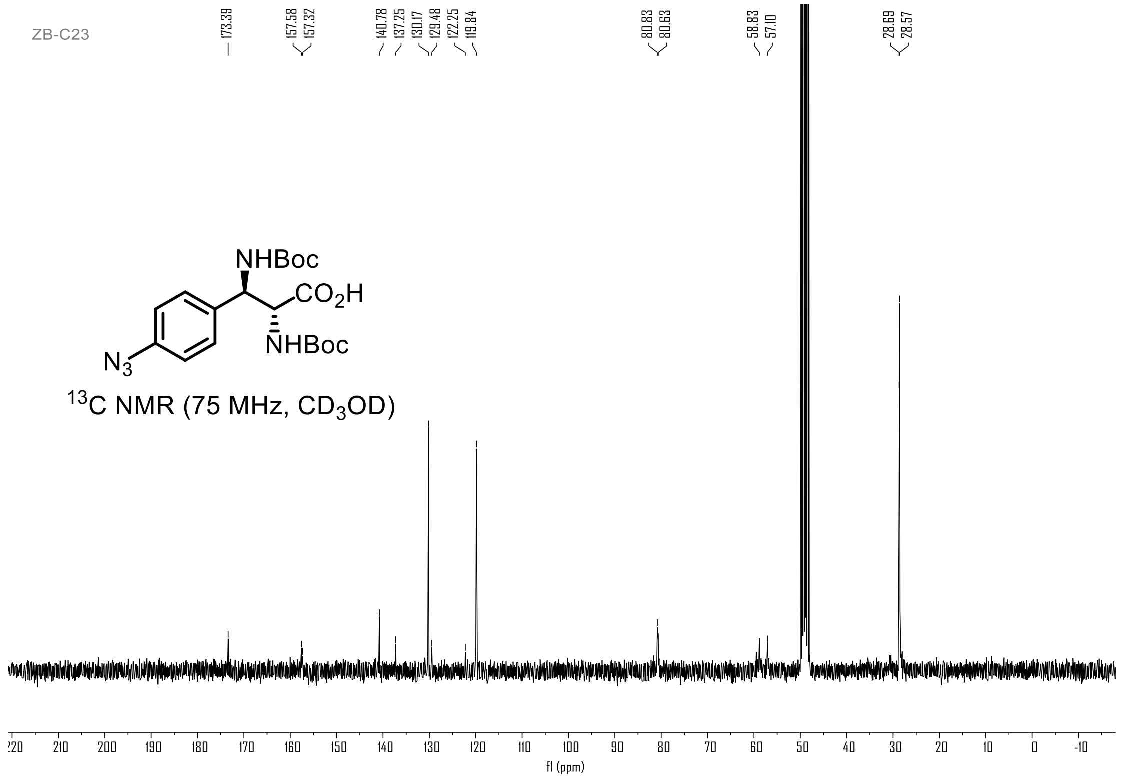

Figure S69. ^{1}H NMR, ^{13}C NMR spectra of **19**.

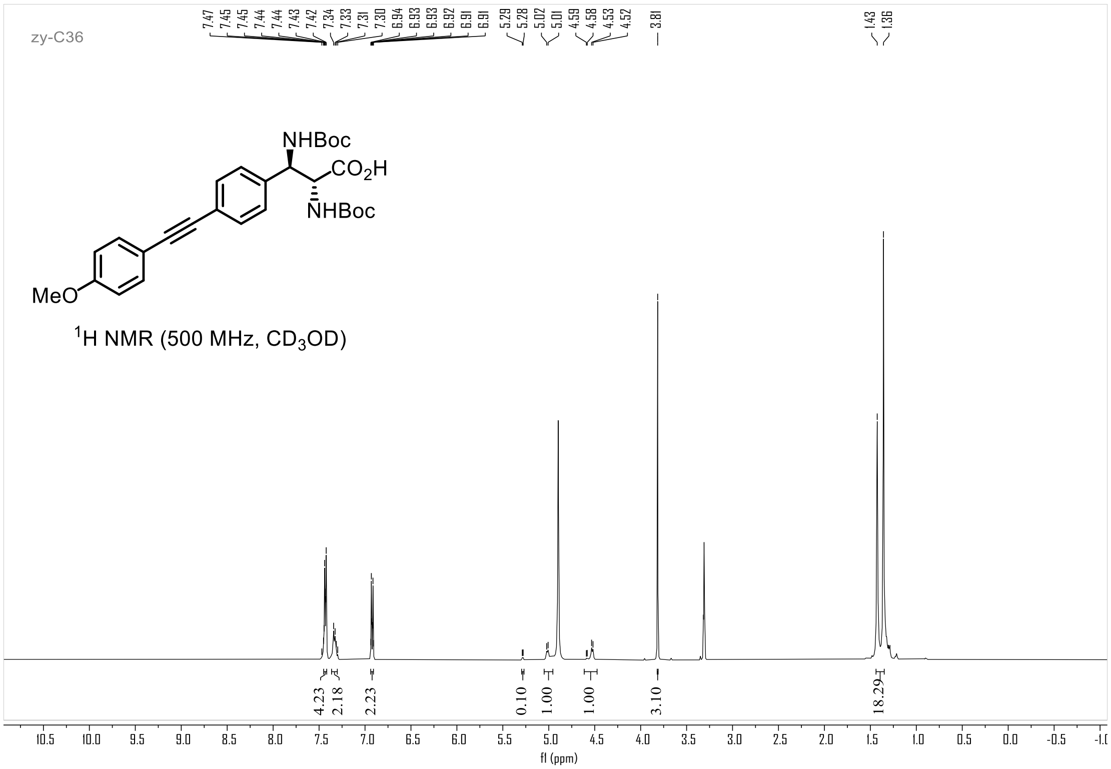

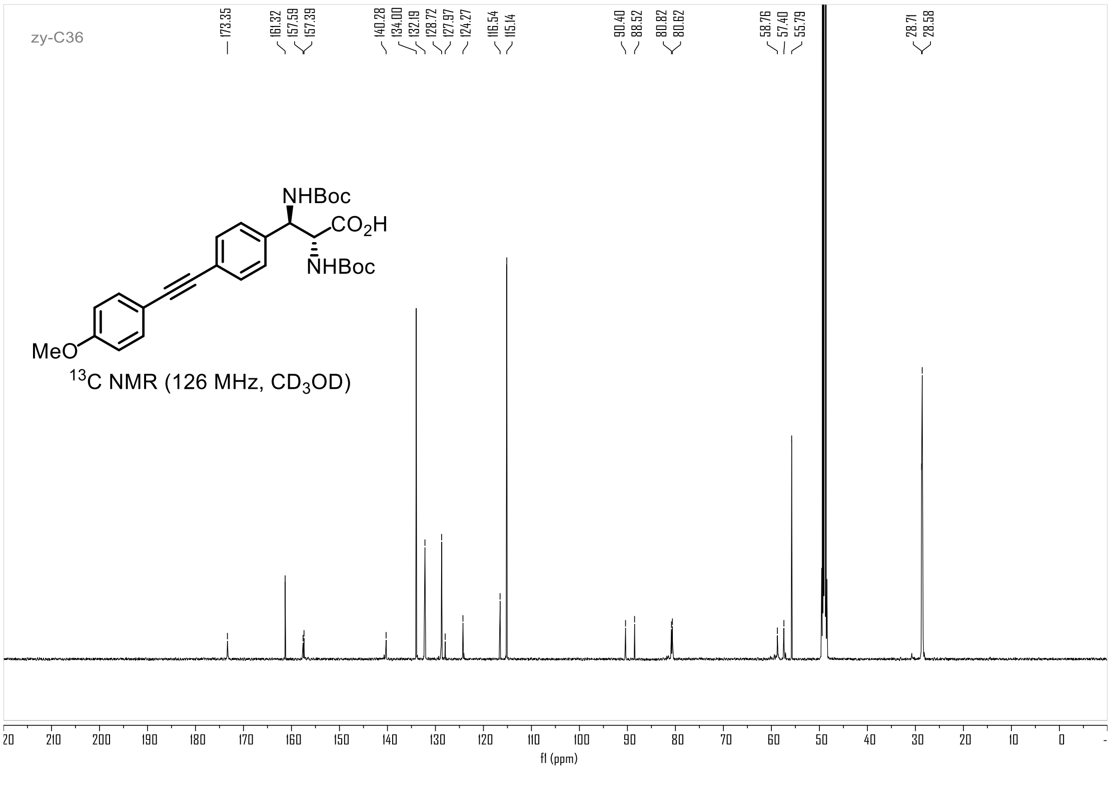

Figure S70. ^{1}H NMR, ^{13}C NMR spectra of **20**.

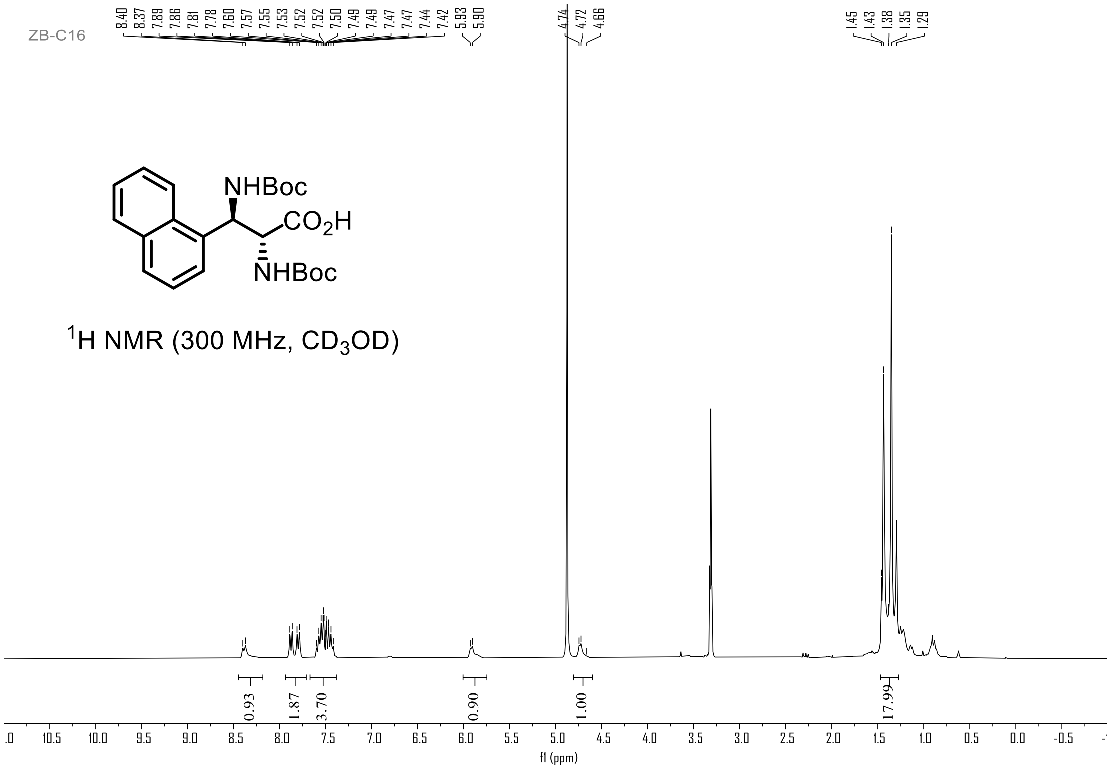

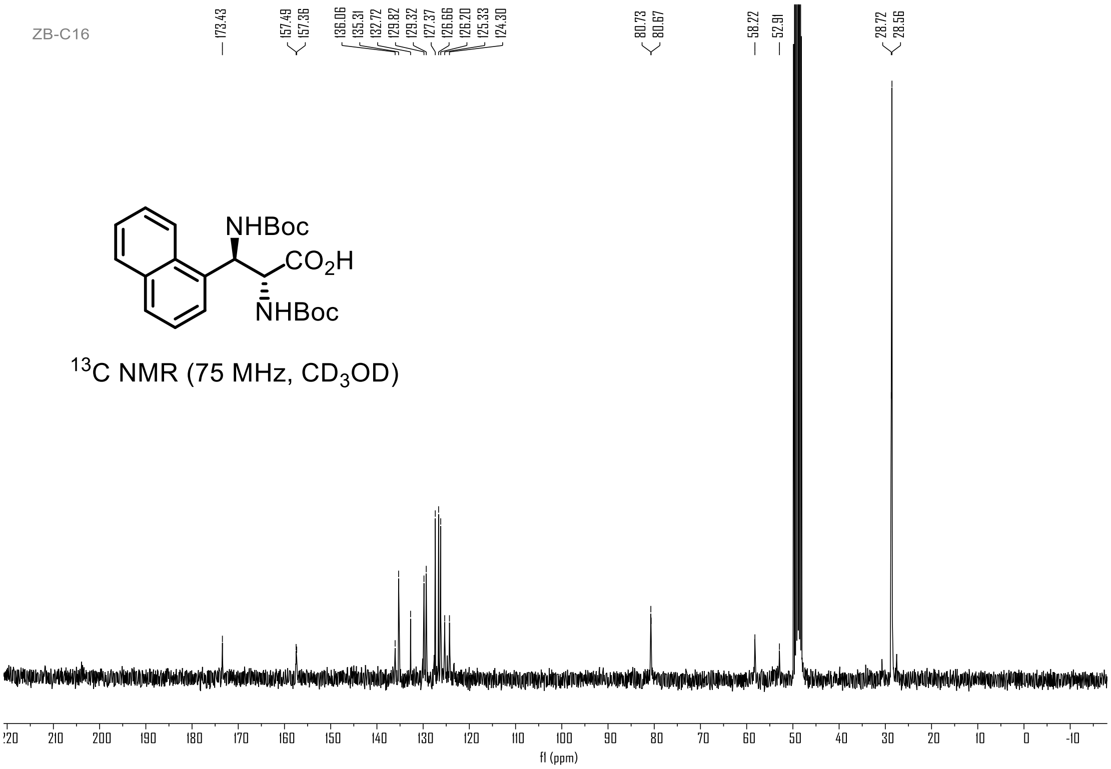

Figure S71. ^{1}H NMR, ^{13}C NMR spectra of **21**.

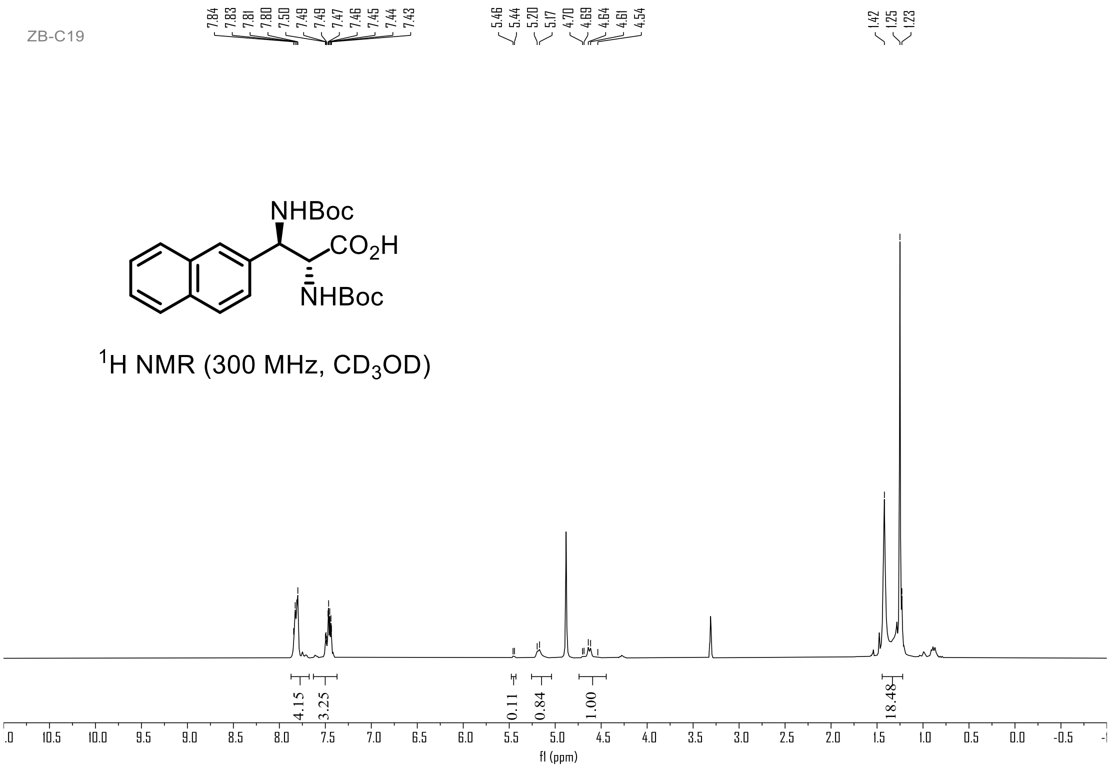

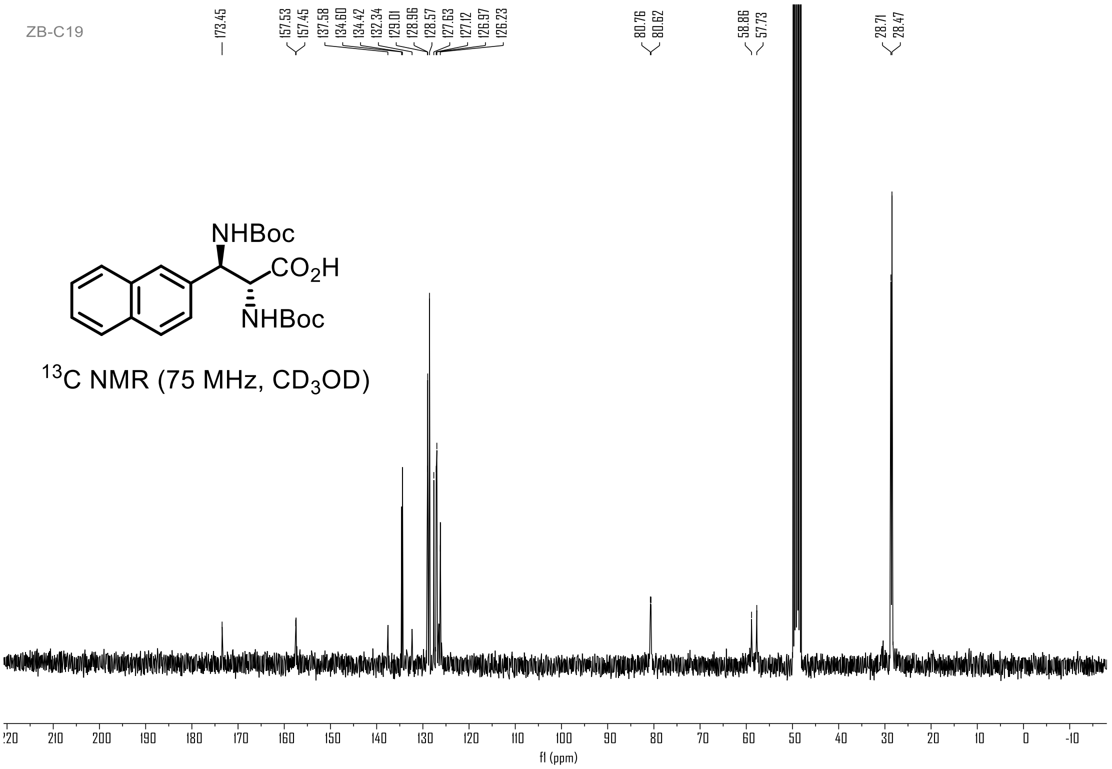

Figure S72. ^{1}H NMR, ^{13}C NMR spectra of **22**.

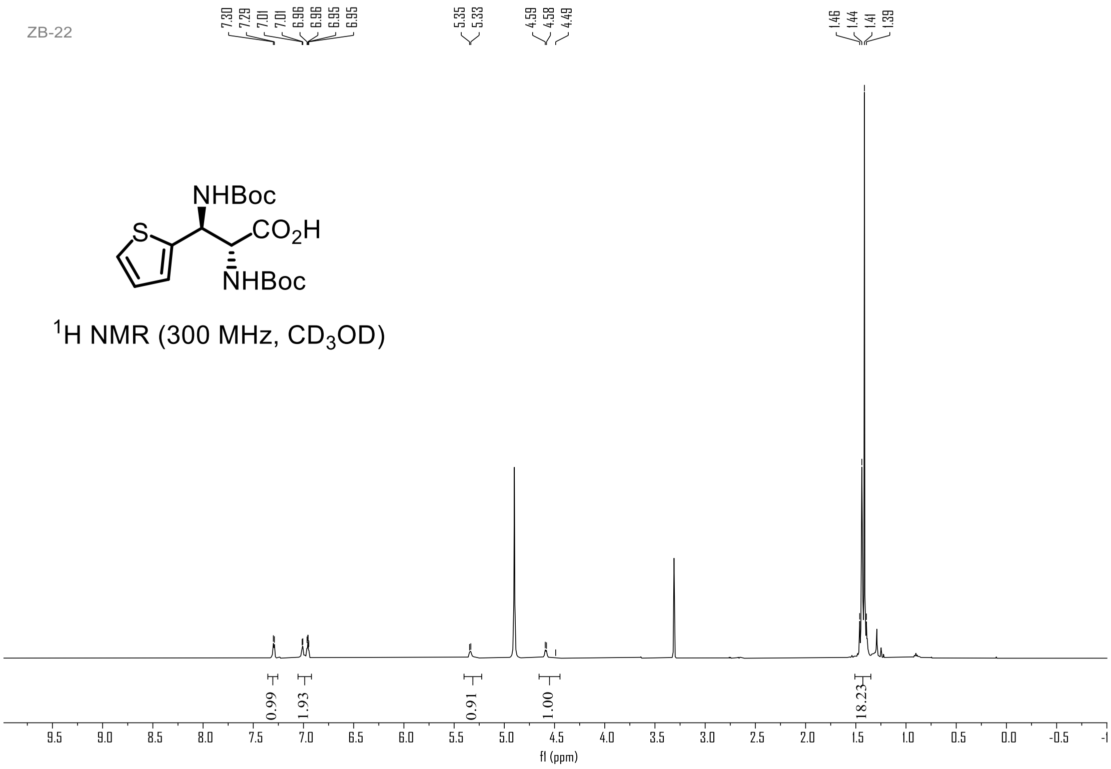

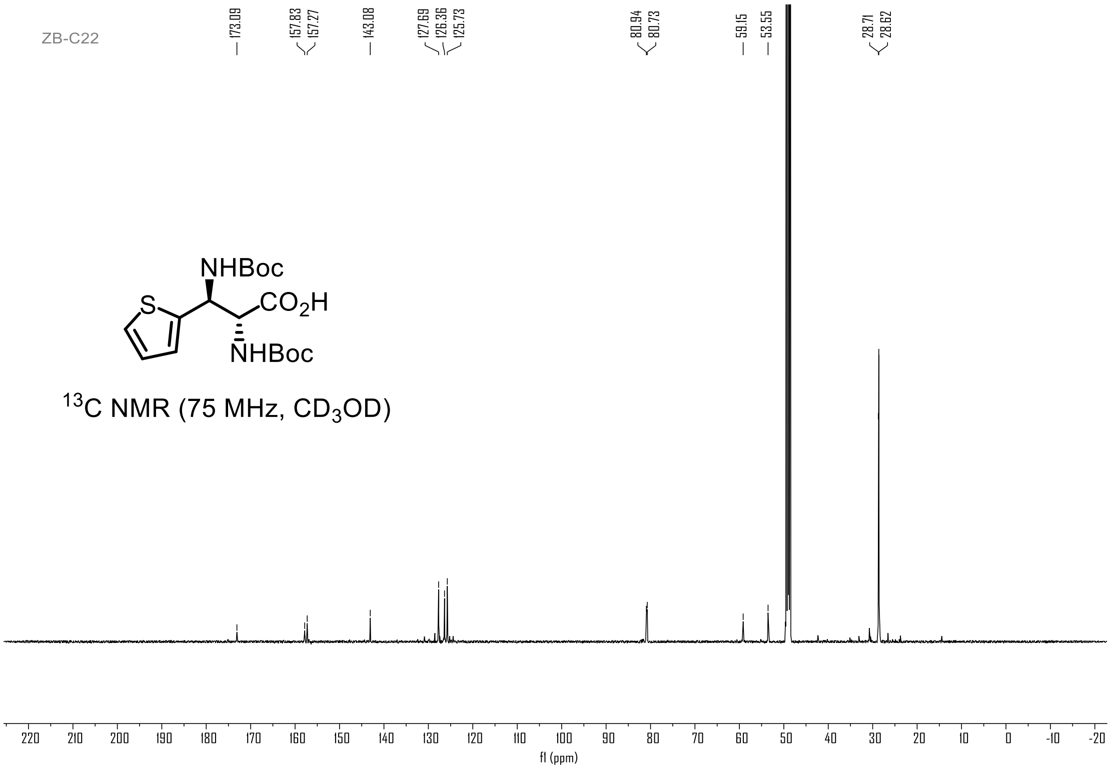

Figure S73. ^{1}H NMR, ^{13}C NMR spectra of **23**.

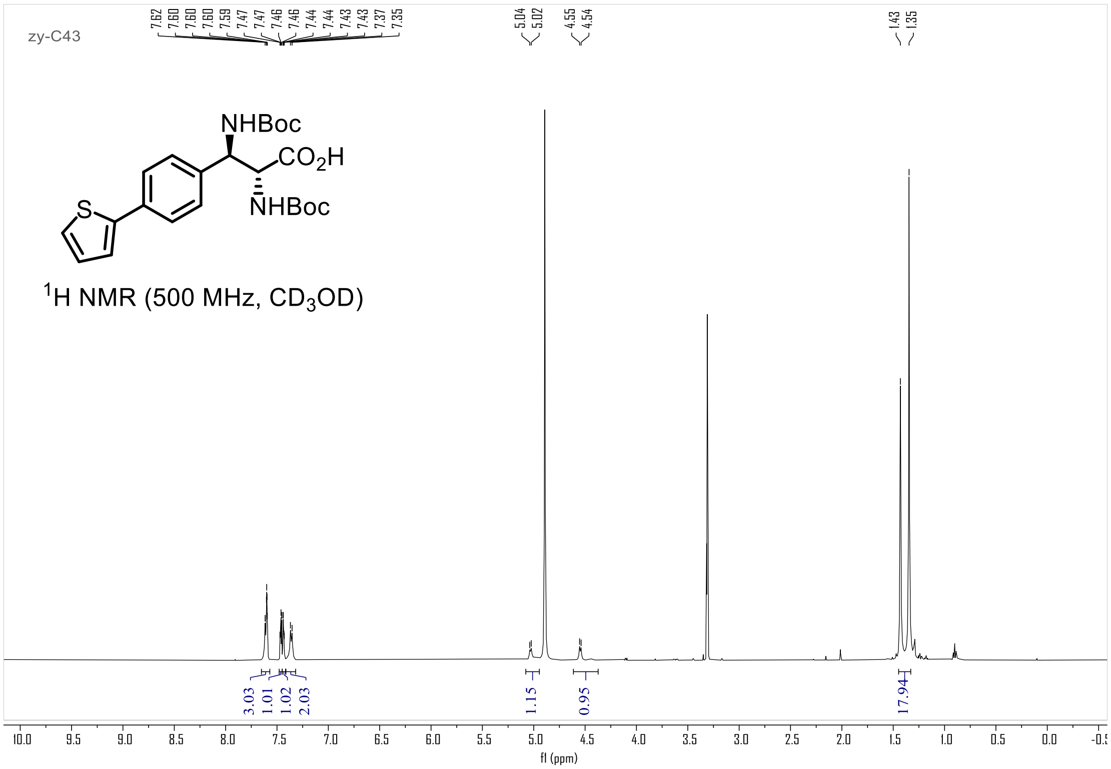

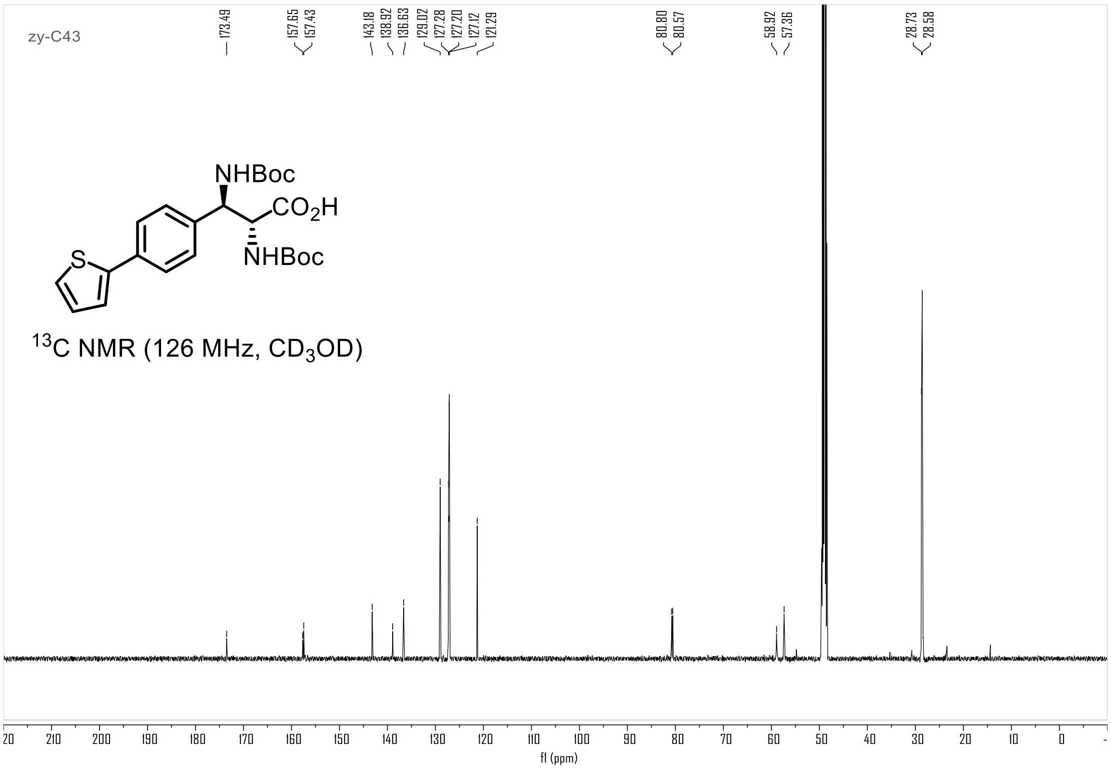

Figure S74. ^{1}H NMR, ^{13}C NMR spectra of **24**.

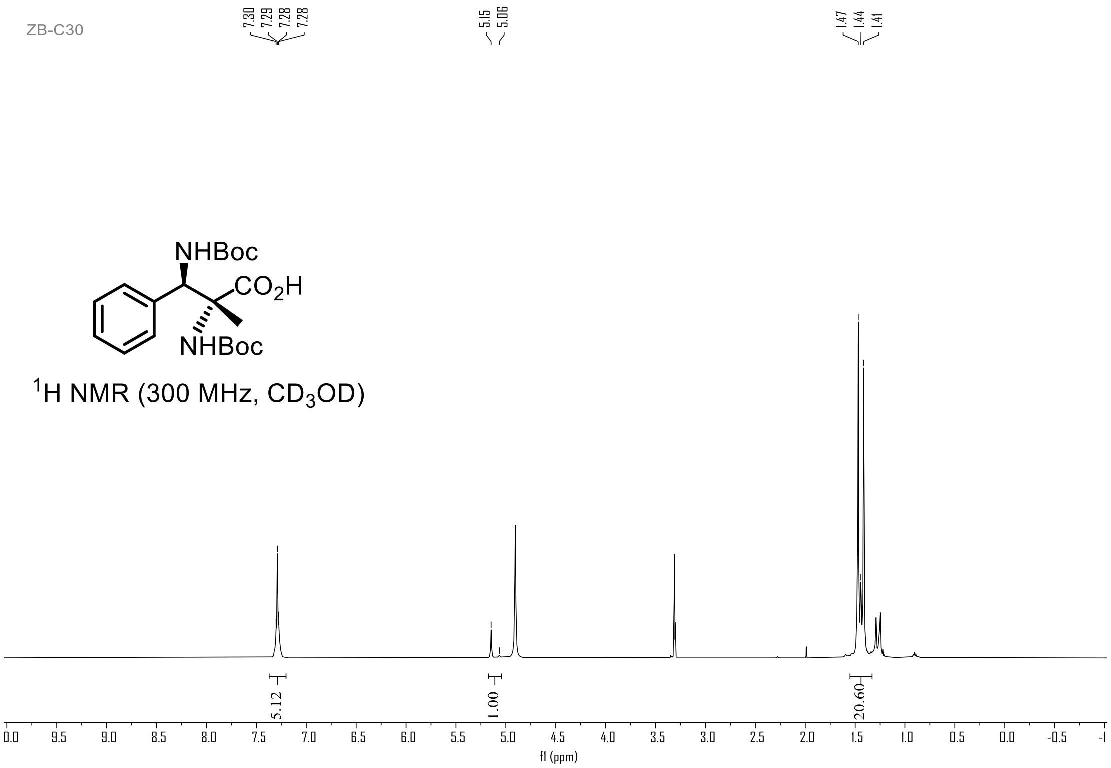

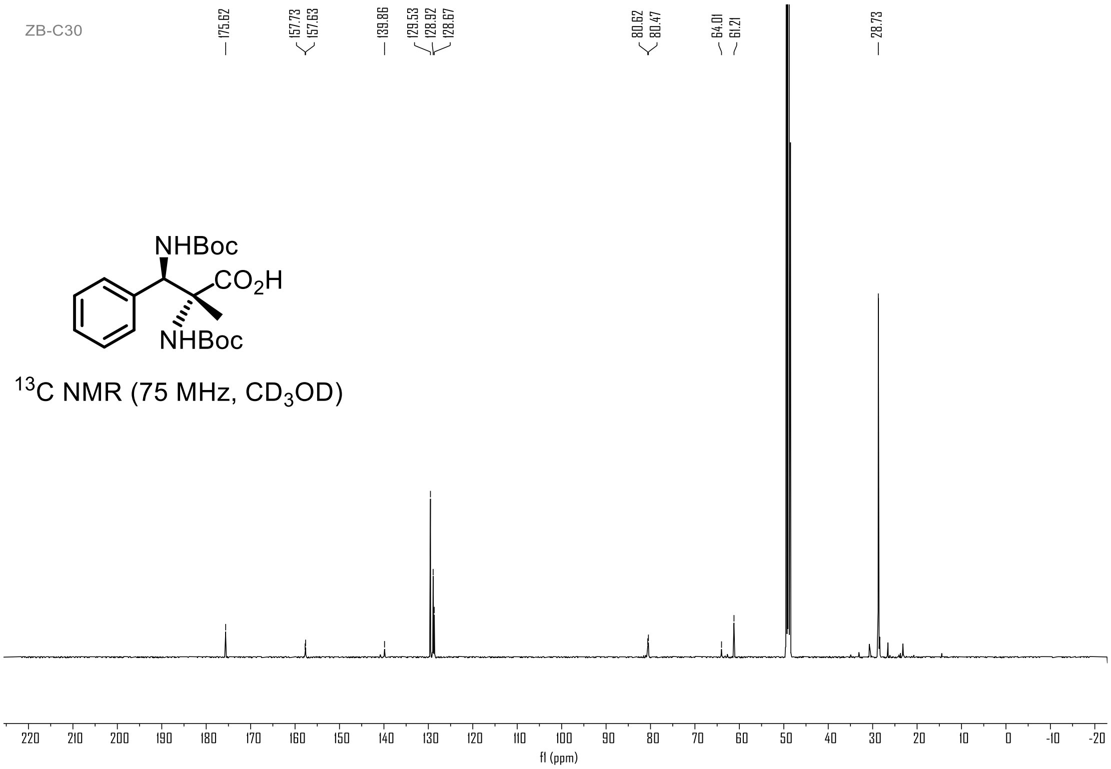

Figure S75. ^{1}H NMR, ^{13}C NMR spectra of **25**.

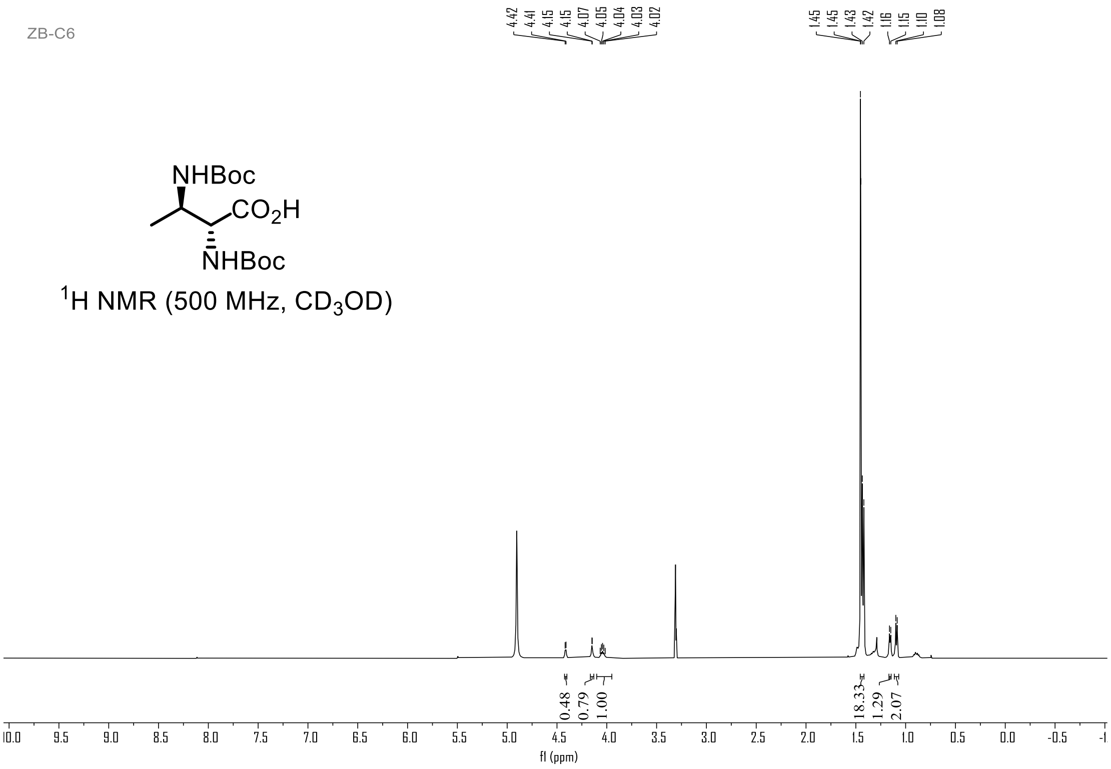

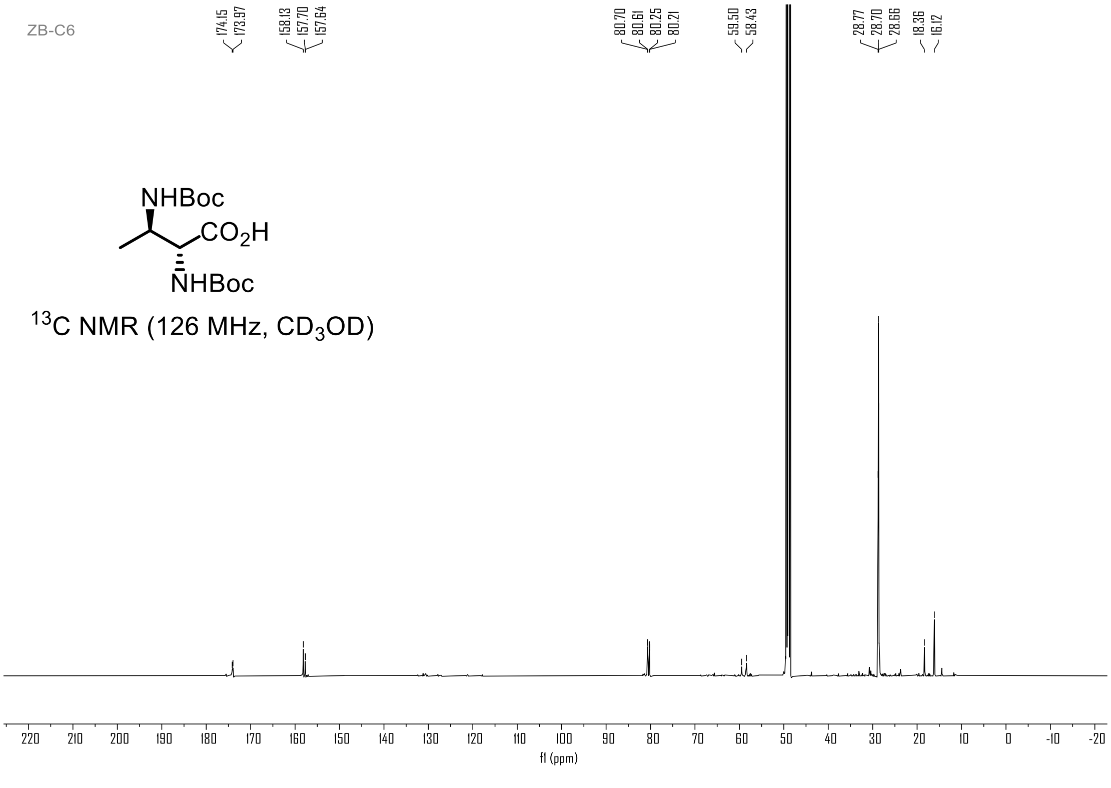

Figure S76. ^{1}H NMR, ^{13}C NMR spectra of **26**.

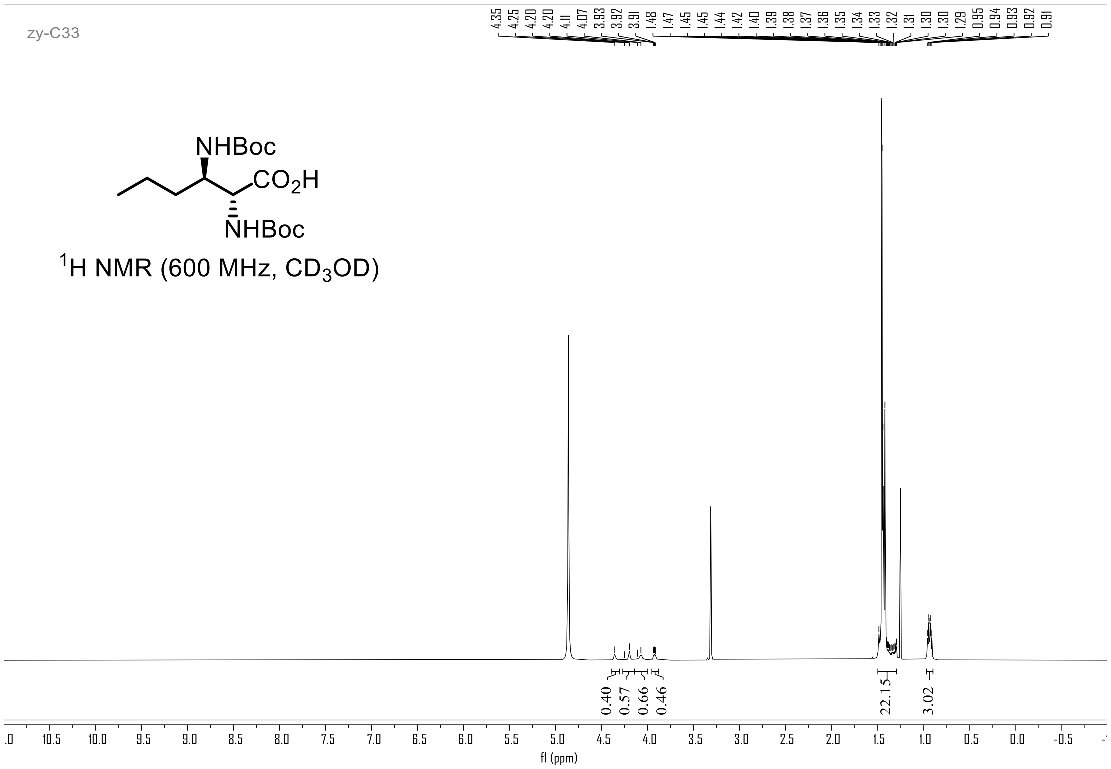

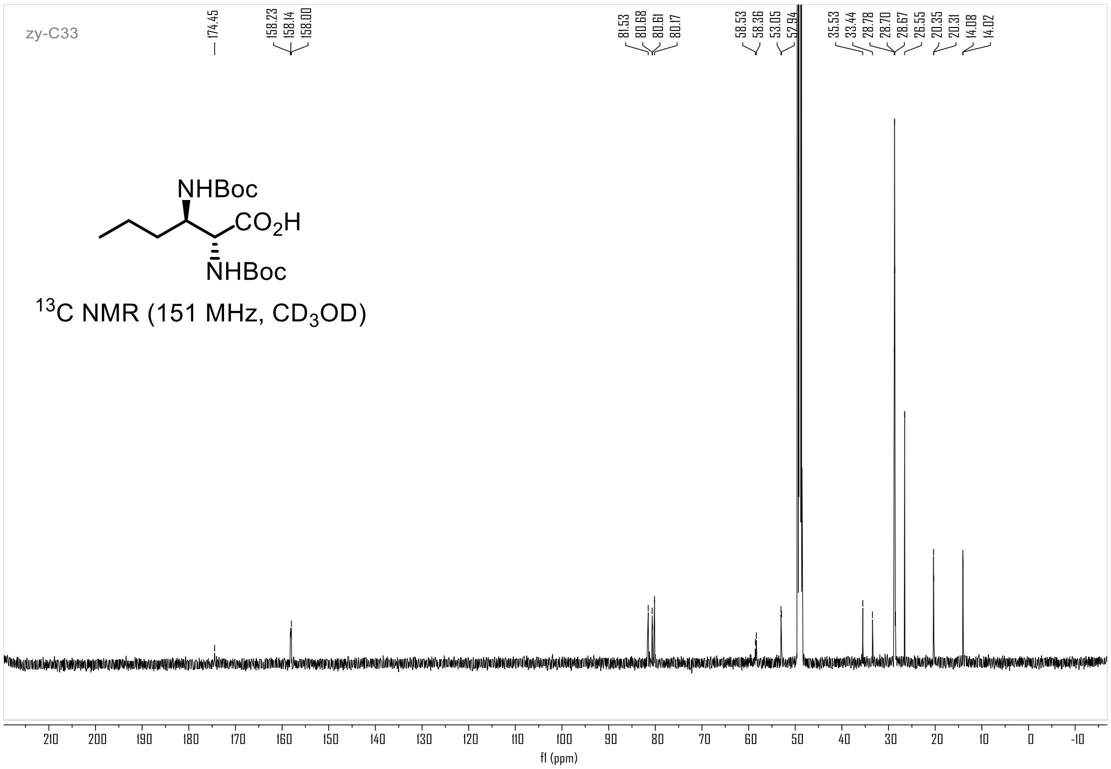

Figure S77. ^{1}H NMR, ^{13}C NMR spectra of **27**.

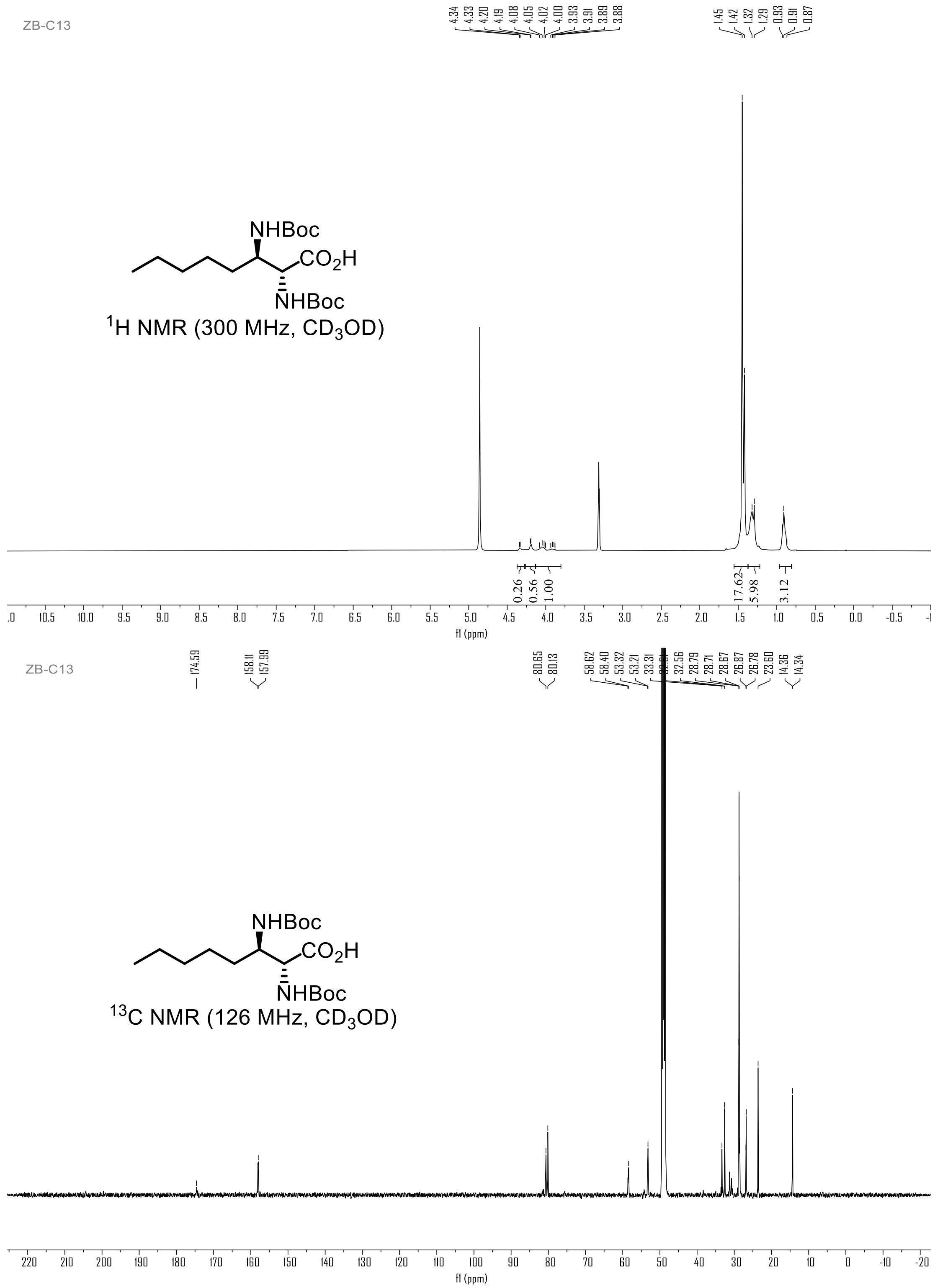

Figure S78. ^{1}H NMR, ^{13}C NMR spectra of **287**.

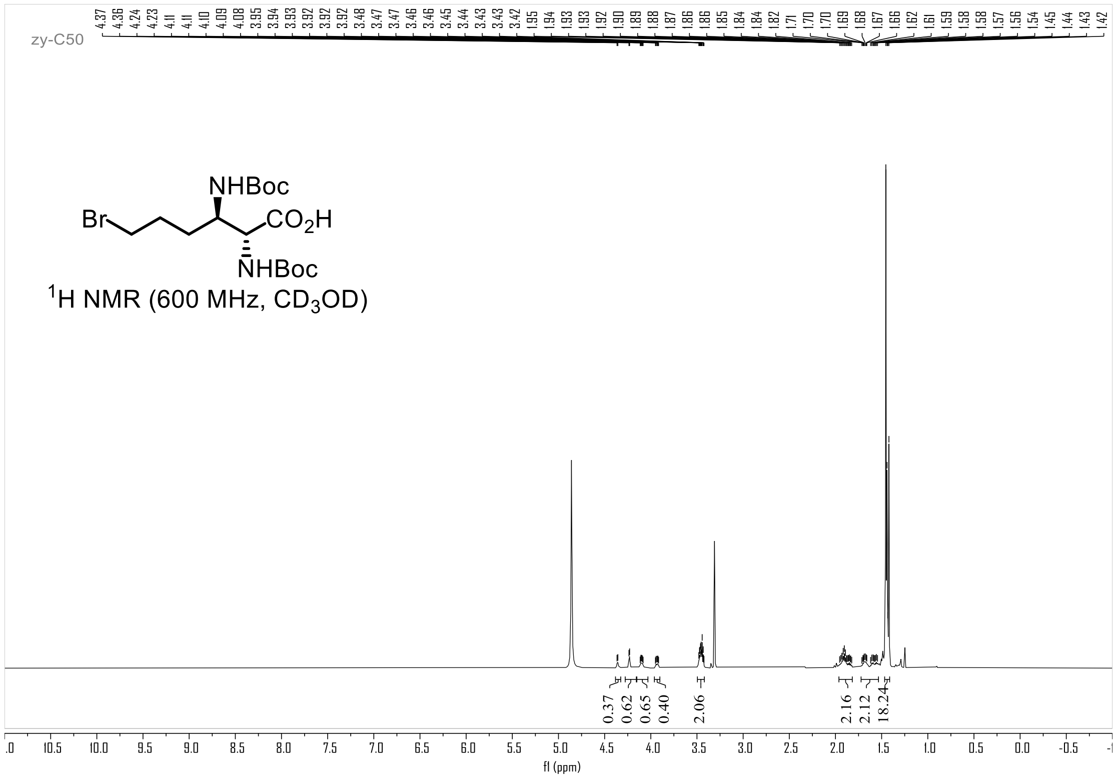

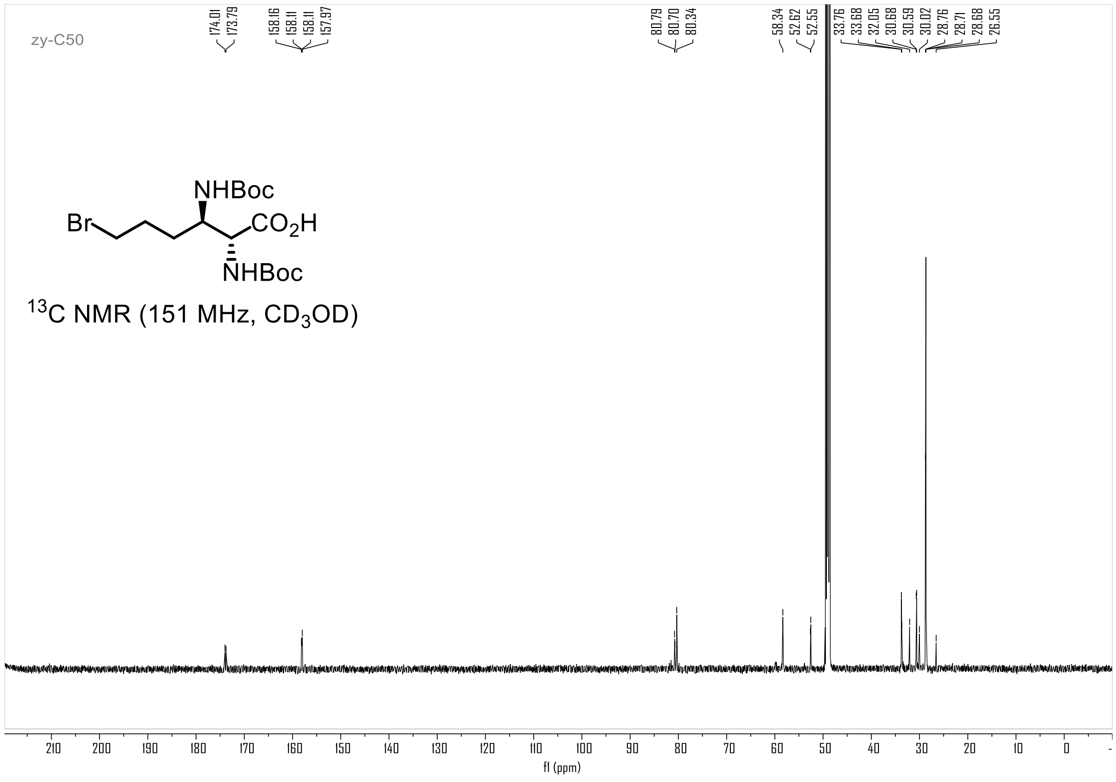

Figure S79. ^{1}H NMR, ^{13}C NMR spectra of **29**.

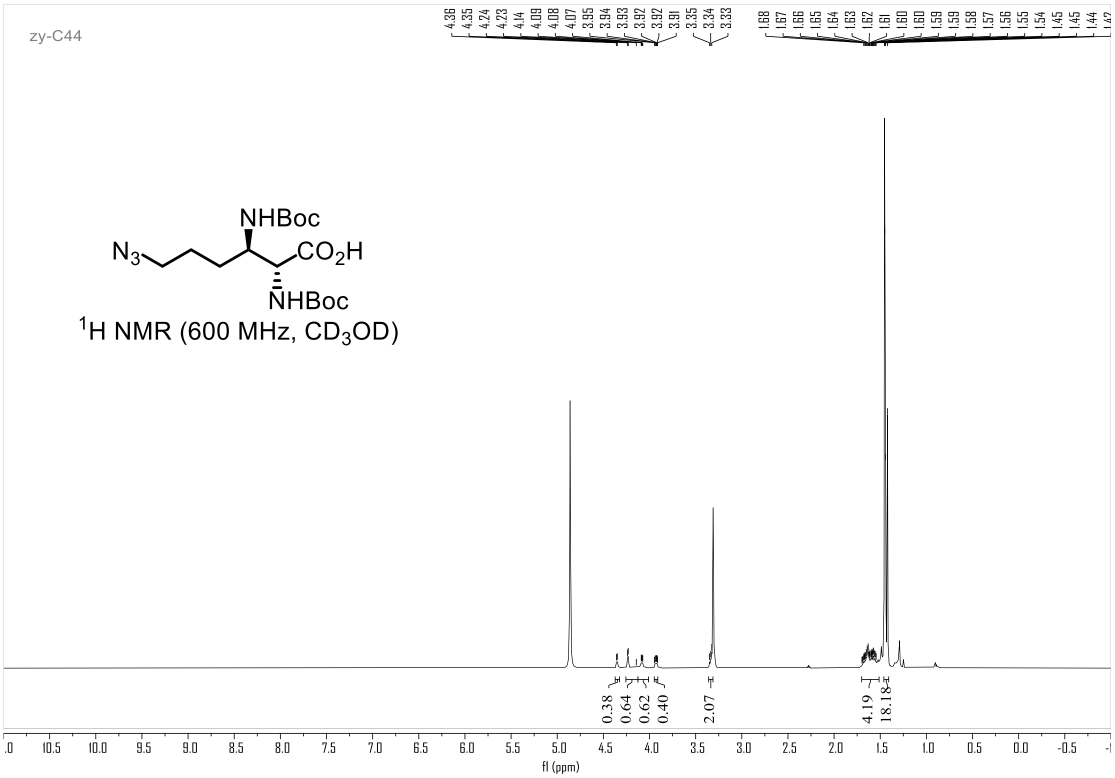

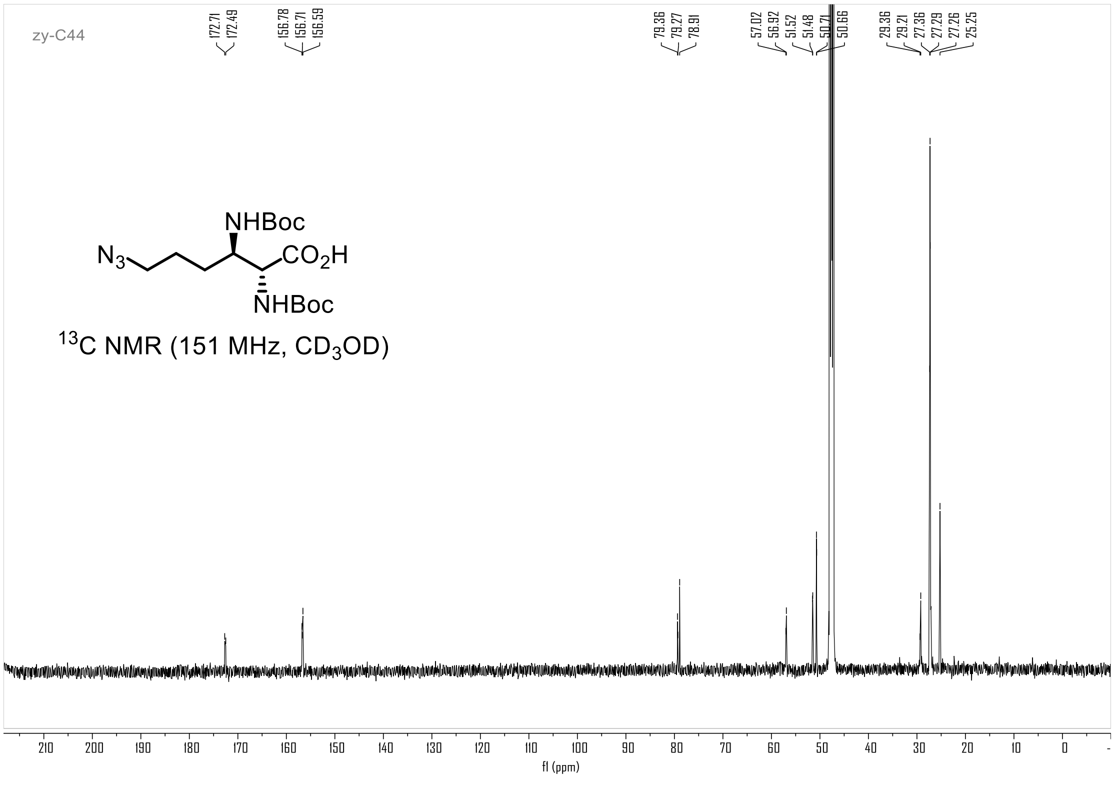

Figure S80. ^{1}H NMR, ^{13}C NMR spectra of **30**.

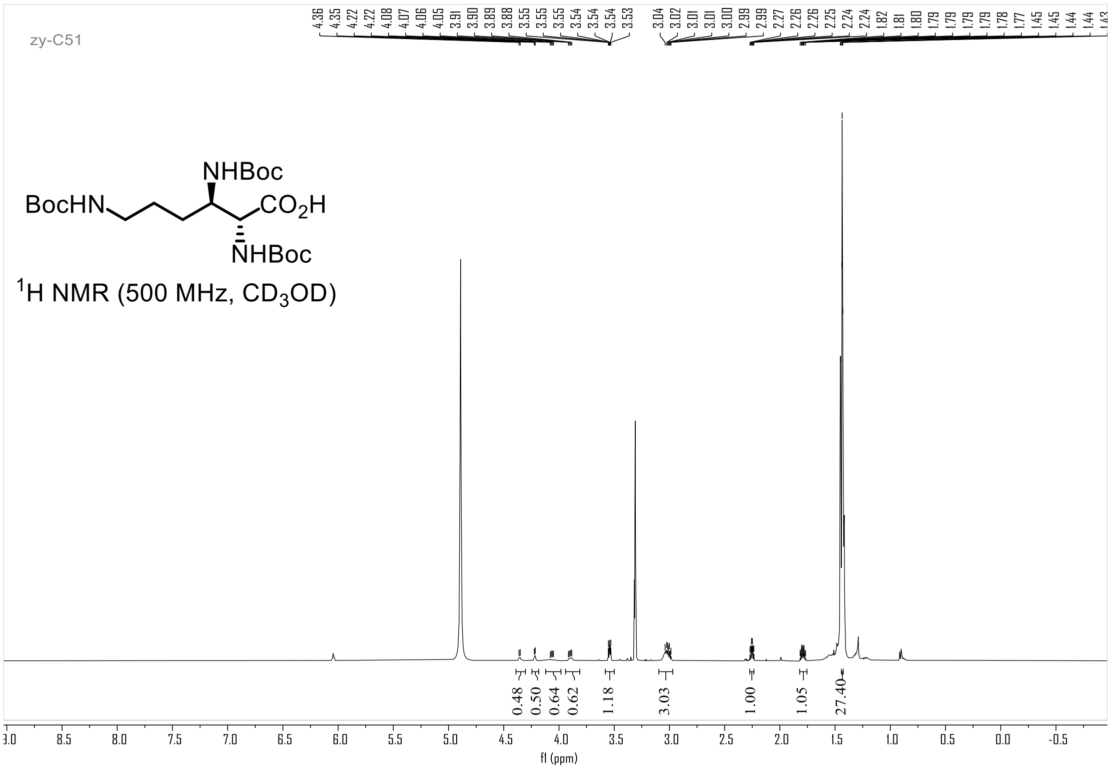

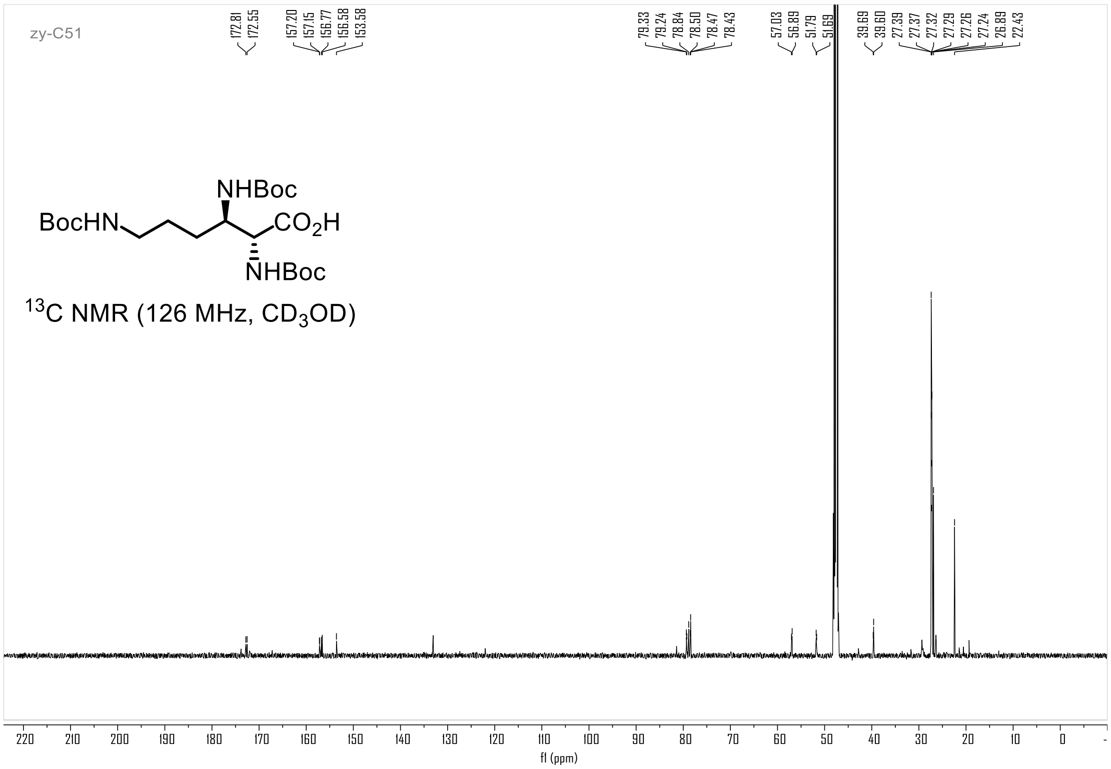

Figure S81. ^{1}H NMR, ^{13}C NMR spectra of **31**.

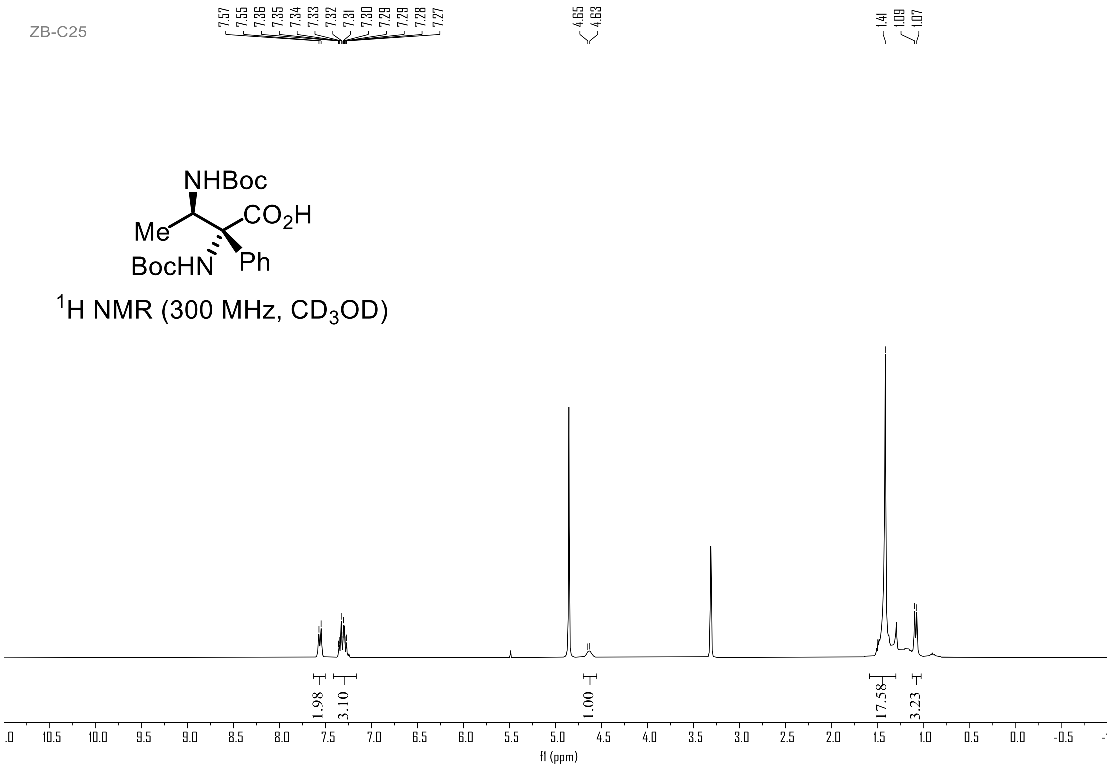

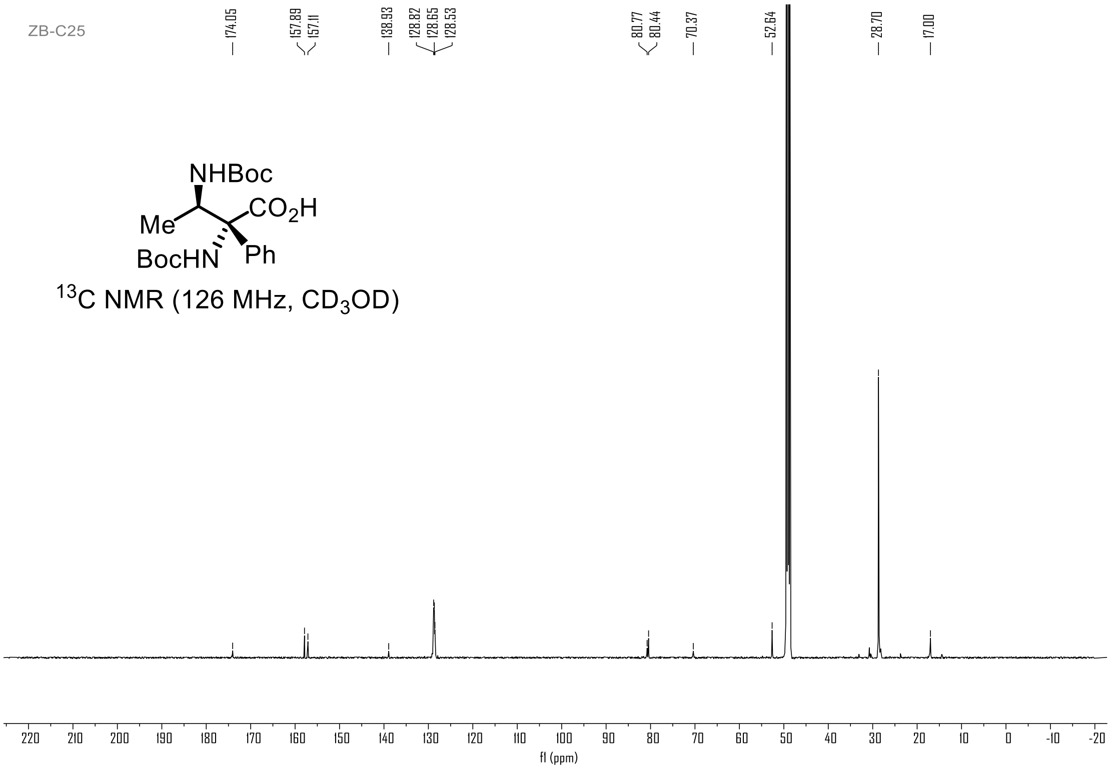

Figure S82. ^{1}H NMR, ^{13}C NMR spectra of **32**.

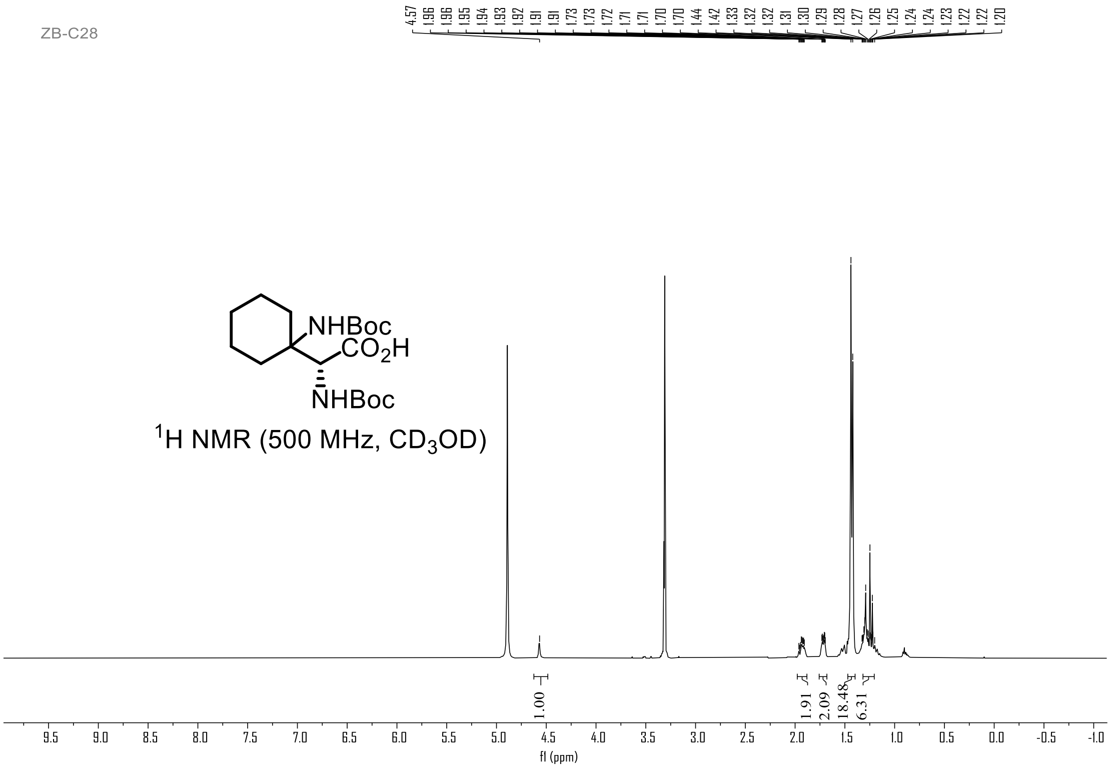

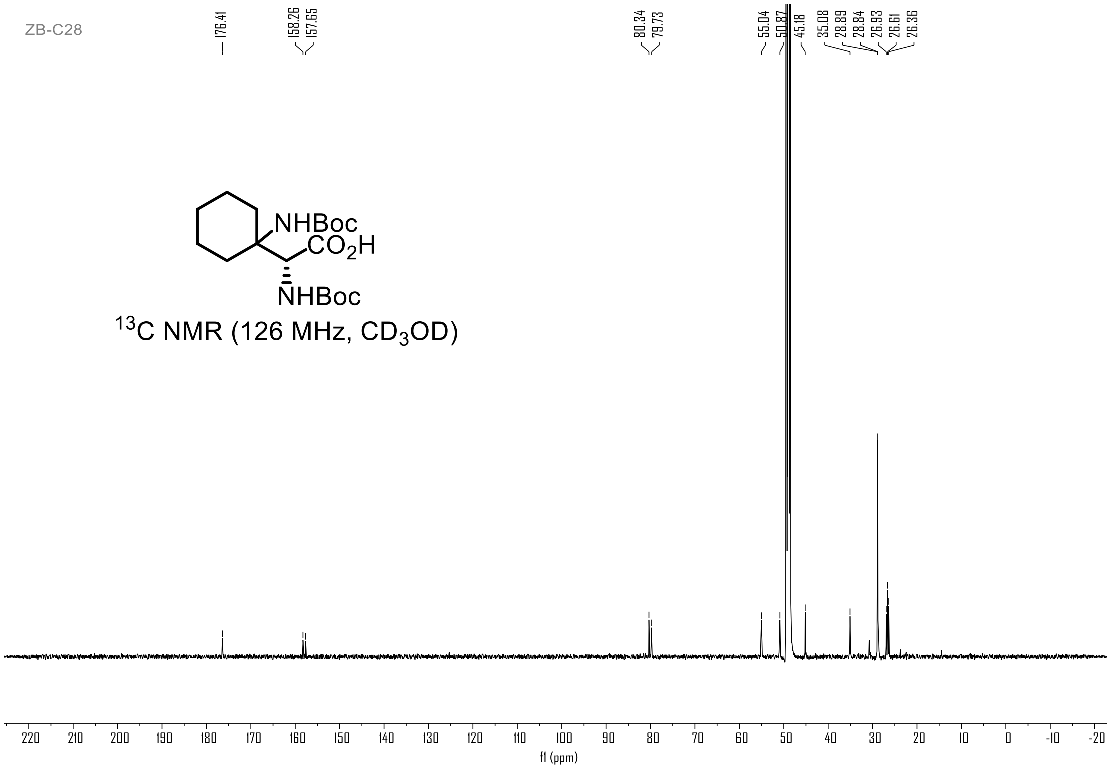

Figure S83. ^1H NMR, ^{13}C NMR spectra of **33**.

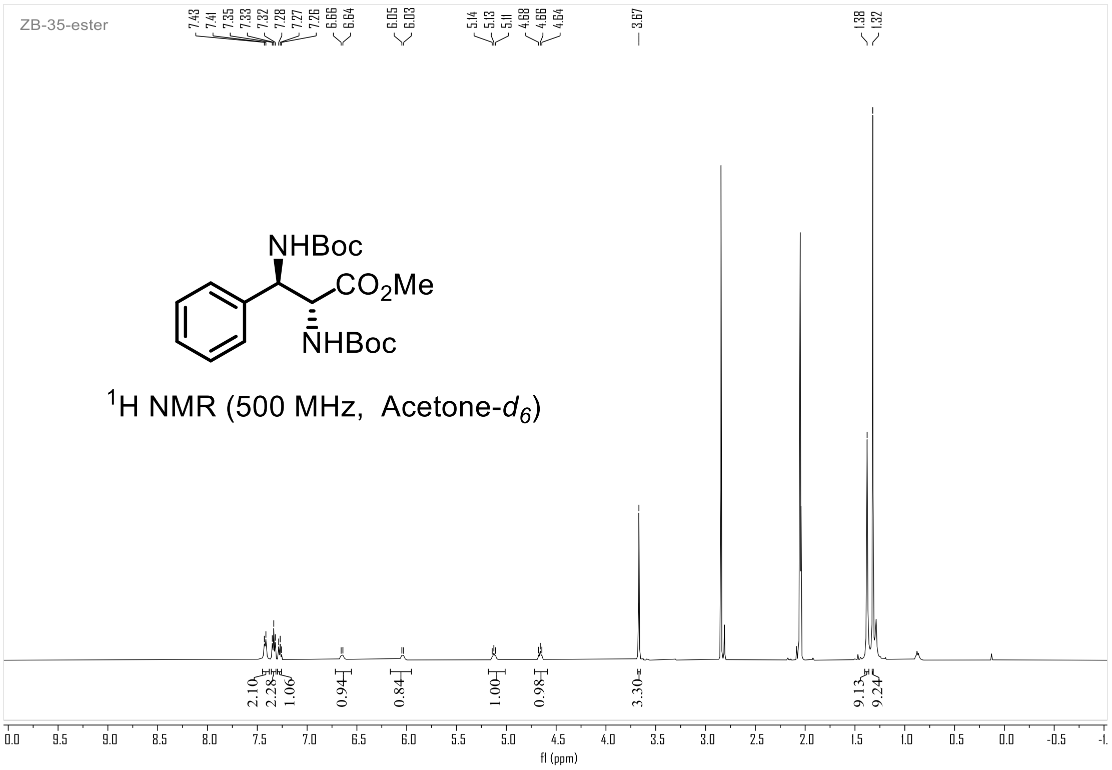

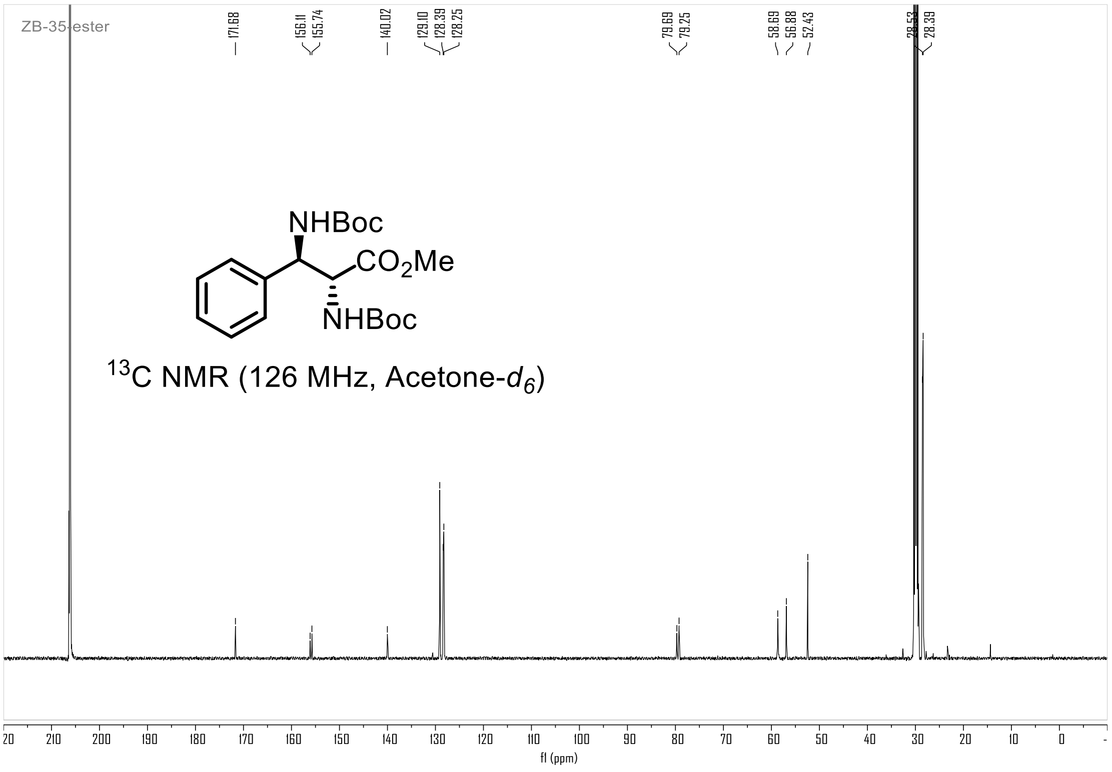

Figure S84. ^{1}H NMR, ^{13}C NMR spectra of **S4**.

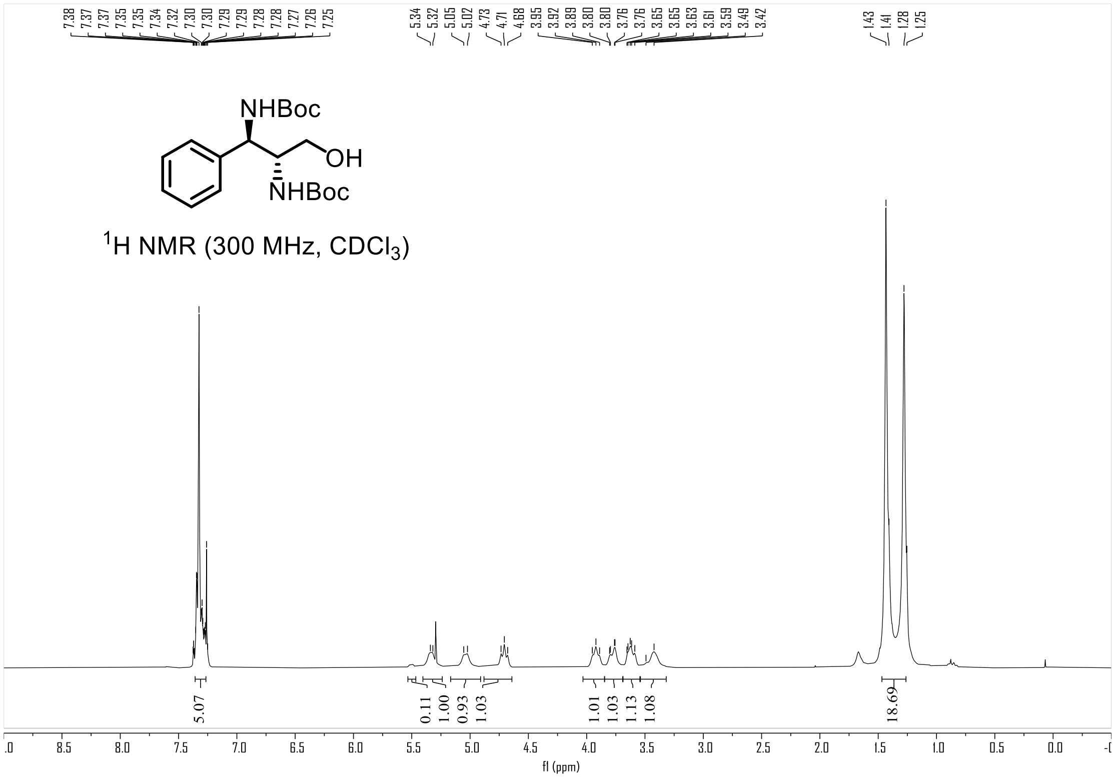

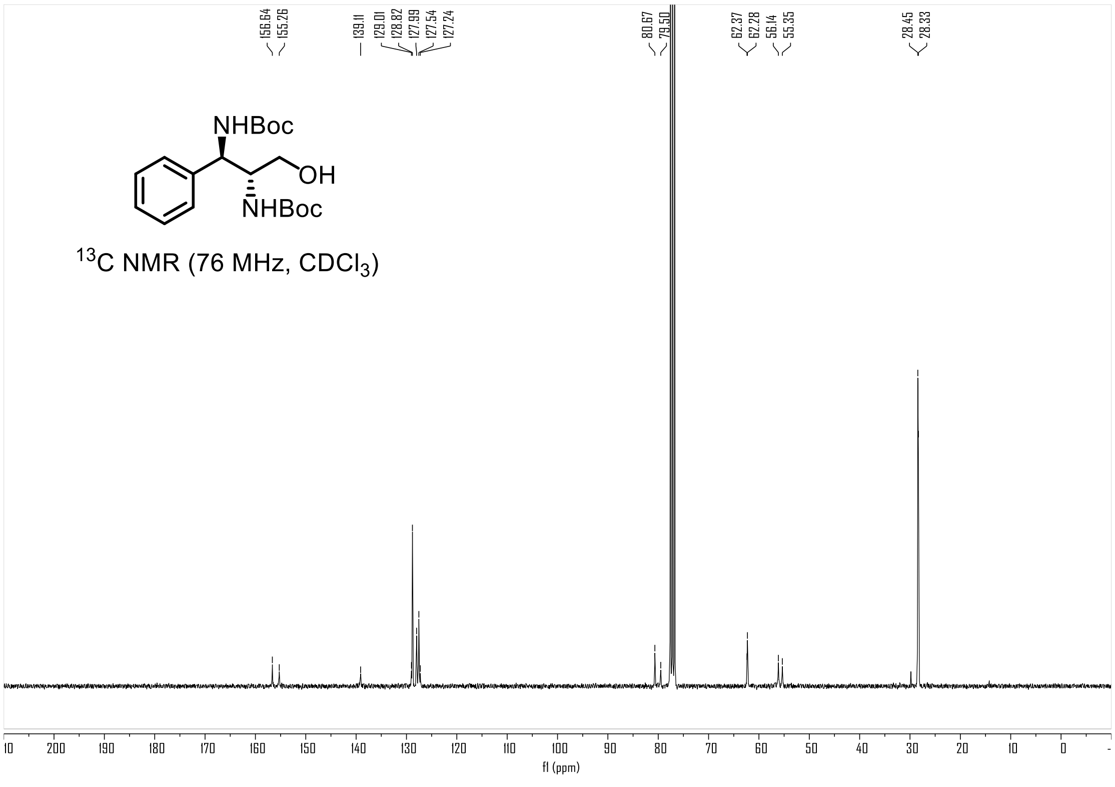

Figure S85. ^{1}H NMR, ^{13}C NMR spectra of **S6**.

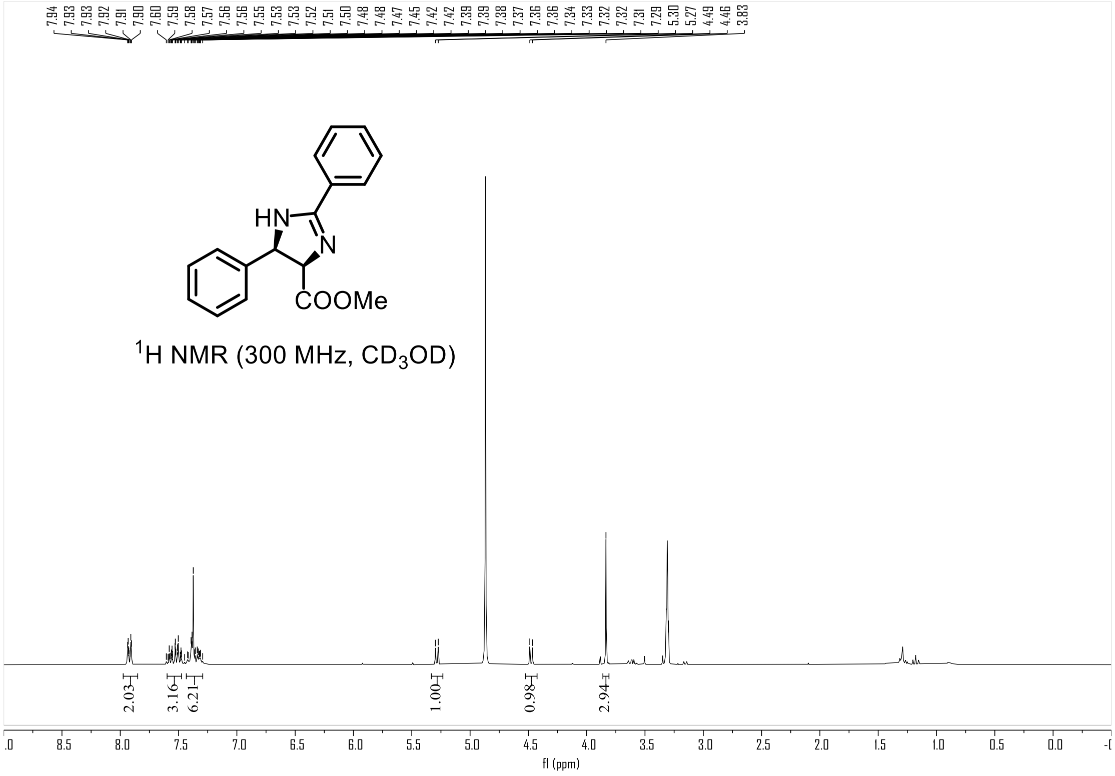

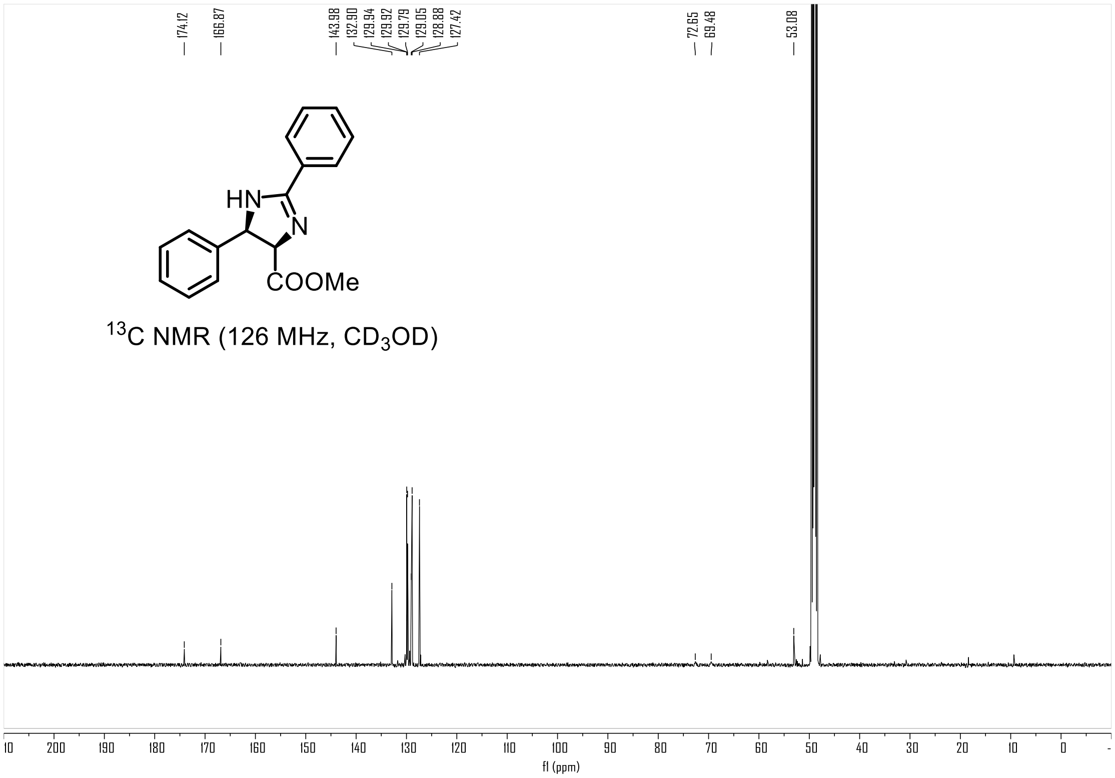

Figure S86. ^{1}H NMR, ^{13}C NMR spectra of **S7**.

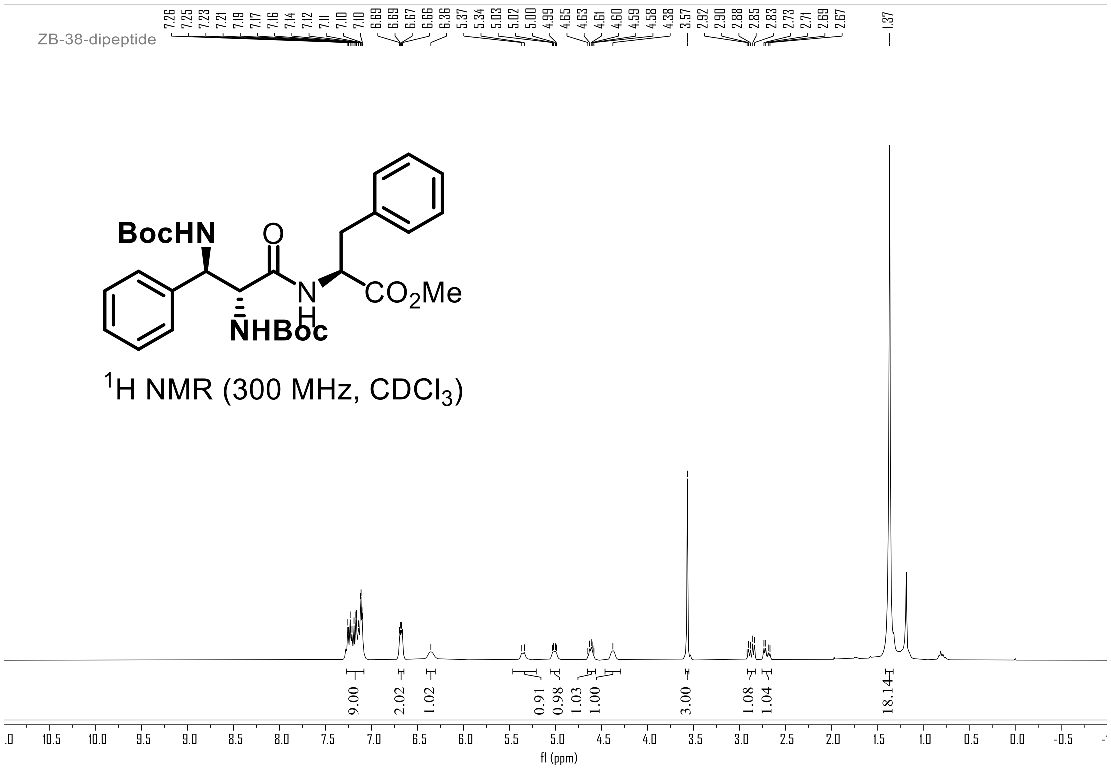

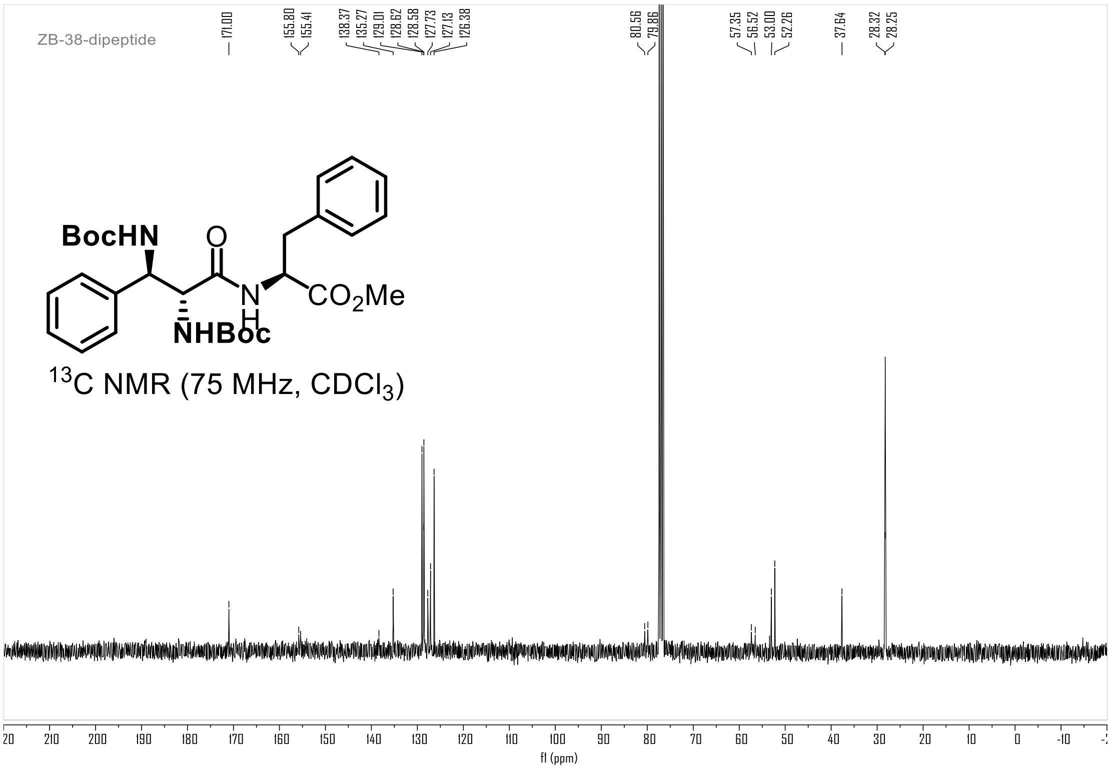

Figure S87. ^{1}H NMR, ^{13}C NMR spectra of **S8**.

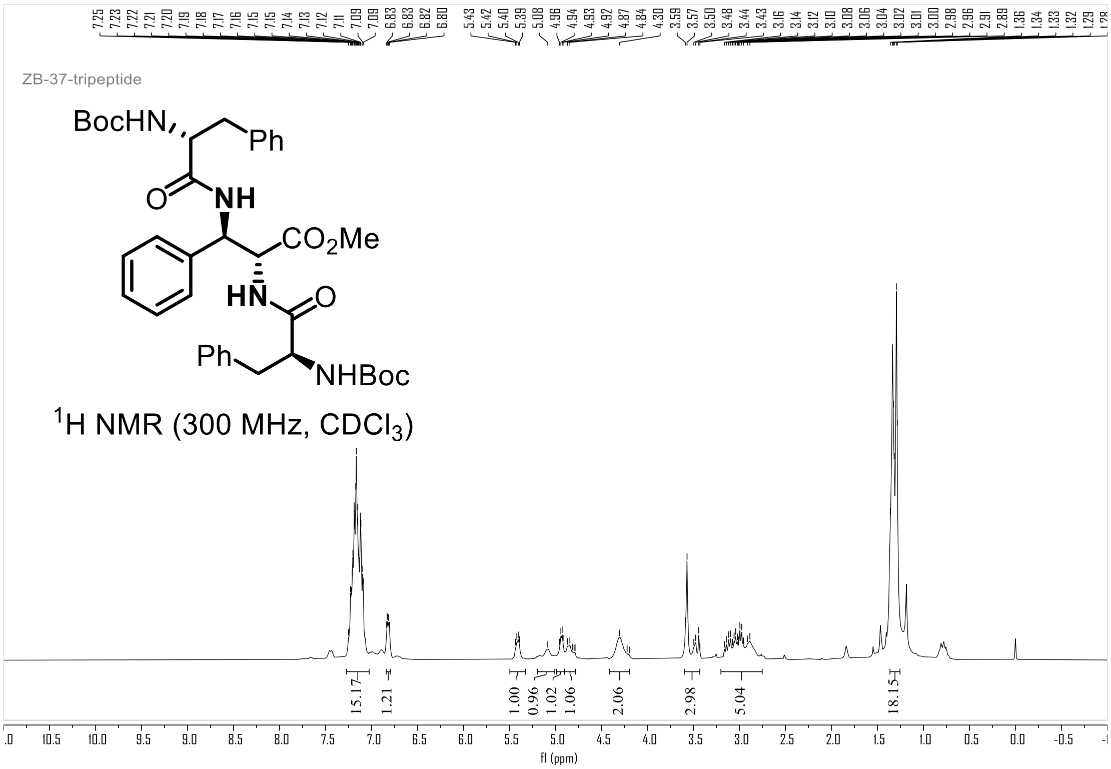

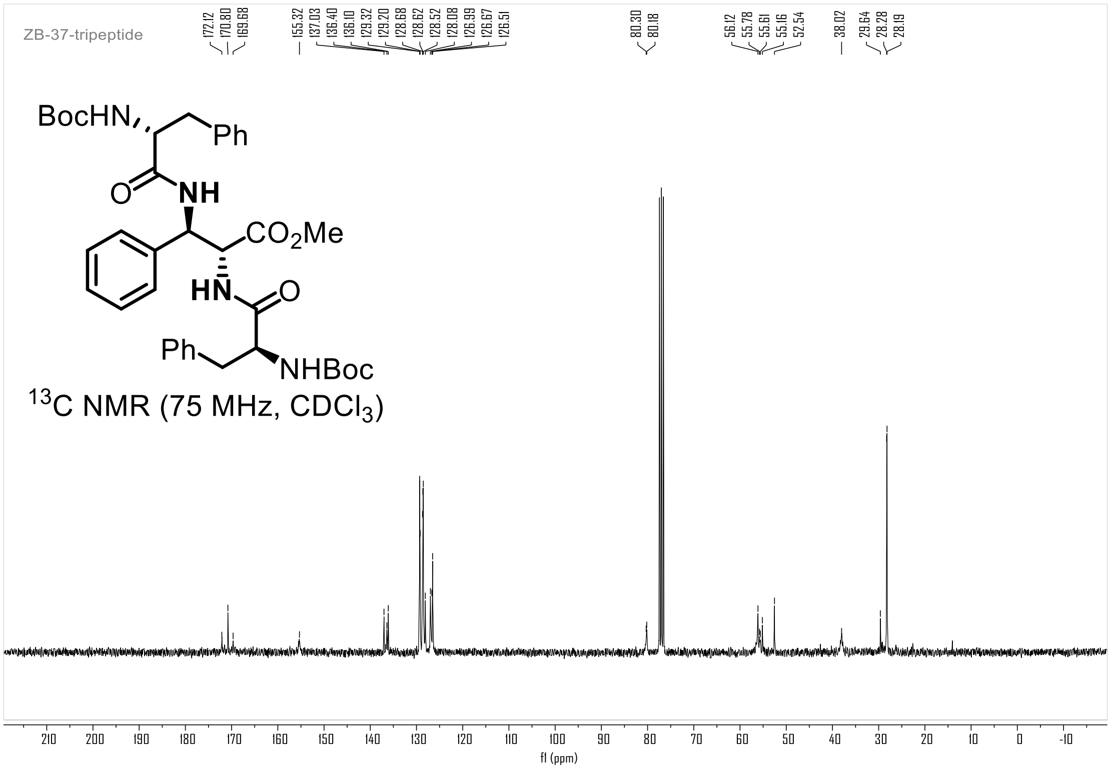

Figure S88. ^{1}H NMR, ^{13}C NMR spectra of **S9**.

Supplementary References

1. C.-X. Ye, X. Shen, S. Chen, E. Meggers, Stereocontrolled 1,3-nitrogen migration to access chiral α-amino acids. *Nat. Chem.* **2022**, *14*, 566–573.

2. P. S. Steinlandt, M. Hemming, X. Xie, S. I. Ivlev, E. Meggers, Trading Symmetry for Stereoinduction in Tetradentate, non-C_2-Symmetric Fe(II)-Complexes for Asymmetric Catalysis. *Chem. Eur. J.* **2023**, *29*, e202300267.

3. M. Chierchia, C. Law, J. P. Morken, Nickel-Catalyzed Enantioselective Conjunctive Cross-Coupling of 9-BBN Borates. *Angew. Chem. In. Ed.* **2017**, *56*, 11870–11874.

4. Y.-N. Wang, *et al.* Discovery of Benzimidazole–Quinolone Hybrids as New Cleaving Agents toward Drug-Resistant Pseudomonas aeruginosa DNA. *ChemMedChem.* **2018**, *13*, 1004–1017.

5. D. F. Evans, The Determination of the Paramagnetic Susceptibility of Substances in Solution by Nuclear Magnetic Resonance. *J. Chem. Soc.* **1959**, 2003–2005.

6. W. C. Dickinson, The Time Average Magnetic Field at the Nucleus in Nuclear Magnetic Resonance Experiments. *Phsy. Rev.* **1951**, *81*, 717–731.

7. D. M. Corsi, C. Platas-Iglesias, H. V. Bekkum, J. A. Peters, Determination of paramagnetic lanthanide(III) concentrations from bulk magnetic susceptibility shifts in NMR spectra. *Magn. Reson. Chem.* **2001**, *39*, 723–726.

8. R. L. Carlin, *Magnetochemistry*, Springer-Verlag: Heidelberg, Germany, **1986**; pp 1–20.

9. B. Weber, F. A. Walker, Solution NMR Studies of Iron(II) Spin-Crossover Complexes. *Inorg. Chem.* **2007**, *46*, 6794–6803.

10. F. H. Köhler, Probing Spin Densities by Use of NMR Spectroscopy, in *Magnetism: Molecules to Materials. Models and Experiments* (Eds.: J. S. Miller, M. Drillon), Wiley-VCH: Weinheim, Germany, **2001**, *4*, 379–430.

11. D. Liße, C. P. Richter, C. Drees, O. Birkholz, C. You, E. Rampazzo, J. Piehler, Monofunctional Stealth Nanoparticle for Unbiased Single Molecule Tracking Inside Living Cells. *Nano Lett.* **2014**, *14*, 2189–2195.

12. P. Zhou, L. Xiang, D. Zhao, J. Ren, Y. Qiu, Y. Li, Synthesis, biological evaluation, and structure activity relationship (SAR) study of pyrrolidine amide derivatives as *N*-acylethanolamine acid amidase (NAAA) inhibitors. *Med. Chem. Commun.* **2019**, *10*, 252–262.

13. D. Fu, *et al.* Design, synthesis and biological evaluation of tyrosinase-targeting PROTACs. *Eur. J. Med. Chem.* **2021**, *2026*, 113850.

14. W. Chen, C. Guo, H. Ding, X. Yang, K. Zhang, Controlled Ring-Opening Polymerization of Macrocyclic Monomers Based on Ring-Opening/Ring-Closing Cascade Reaction. *J. Am. Chem. Soc.* **2023**, *145*, 25022–25030.

15. S. G. Davies, A. W. Mulvaney, A. J. Russell, A. D. Smith, Parallel synthesis of homochiral *β*-amino acids. *Tetrahedron: Asymmetry.* **2007**, *18*, 1554–1566.

16. B. Zhou, C.-X. Ye, E. Meggers, *N*-Boc-Protected *α*-Amino Acids by 1,3-Migratory Nitrene C(sp^3)-H Insertion. *Eur. J. Org. Chem.* **2023**, *26*, e202300296.

17. C.-X. Ye, D. R. Dansby, S. Chen, E. Meggers, Expedited synthesis of α-amino acids by single-step enantioselective a-amination of carboxylic acids. *Nat. Synth.* **2023**, *2*, 645–652.

18. J. Hernández-Toribio, R. G. Arrayás, J. C. Carretero, Direct Mannich Reaction of Glycinate Schiff Bases with N-(8-Quinolyl)sulfonyl Imines: A Catalytic Asymmetric Approach to *anti-α,β*-Diamino Esters. *J. Am. Chem. Soc.* **2008**, *130*, 16150–16151.

19. S.-H. Lee, J. Yoon, S.-H. Chung, Y.-S. Lee, Efficient asymmetric synthesis of 2,3-diamino-3-phenylpropanoic acid derivatives. *Tetrahedron.* **2001**, *57*, 2139–2145.

20. APEX3 V2019.11-2, Bruker AXS Inc., Madison, Wisconsin, USA, **2019**.

21. SADABS, Bruker AXS Inc., Madison, Wisconsin, USA, **2016**.

22. L. Krause, R. Herbst-Irmer, G. M. Sheldrick, D. Stalke, Comparison of silver and molybdenum microfocus X-ray sources for single-crystal structure determination. *J. Appl. Crystallogr.* **2015**, *48*, 3–10.

23. G. M. Sheldrick, SHELXT– Integrated space-group and crystal structure determination. *Acta Crystallogr.* **2015**, *A71*, 3–8.

24. G. M. Sheldrick, Crystal structure refinement with SHELXL. *Acta Crystallogr.* **2015**, *C71*, 3–8.

25 C. B. Hübschle, G. M. Sheldrick, B. Dittrich, *ShelXle*: a Qt graphical user interface for *SHELXL J. Appl. Crystallogr.* **2011**, *44*, 1281–1284.

26. A. L. Spek, *PLATON* SQUEEZE: a tool for the calculation of the disordered solvent contribution to the calculated structure factors. *Acta Crystallogr., Sect. C: Struct. Chem.* **2015**, *71*, 9–18.

27. A. L. Spek, *PLATON - A Multipurpose Crystallographic Tool*, Utrecht University, Utrecht, The Netherlands, **2019**.
